# Metal‐dependent regulated cell death: Molecular architecture and translational frontiers

**DOI:** 10.1002/imt2.70141

**Published:** 2026-07-06

**Authors:** Haoliang Hu, Zhe Chen, Yaqi Li, Jiayi Peng, Jiangang Cao, Hong Zhou, Mengqi Wang, Yuejia Du, Hailin Wu, Huiqin Zhao, Shifang Huang, Dianmei Yu, Meiqing Liu, Olga V. Shevchenko, Natalia Yu. Matveeva, Yiyuan Yang, Kerui Huang, Deguan Lv, Junxia Min, Linxi Chen, Fudi Wang

**Affiliations:** ^1^ The Second Affiliated Hospital, School of Public Health, State Key Laboratory of Experimental Hematology Zhejiang University School of Medicine Hangzhou China; ^2^ The Key Laboratory of Study and Discovery of Small Targeted Molecules of Hunan Province, School of Pharmaceutic Science, Health Science Center Hunan Normal University Changsha China; ^3^ State Key Laboratory of Microbial Technology (Changde R&D Base), Institute of Synthetic Biology Industry Hunan University of Arts and Science Changde China; ^4^ Institute of Pharmacy and Pharmacology, Hunan Province Cooperative Innovation Center for Molecular Target New Drug Study, Hengyang Medical College University of South China Hengyang China; ^5^ The National and Local Joint Engineering Laboratory of Animal Peptide Drug Development, College of Life Sciences Hunan Normal University Changsha China; ^6^ Peptide and Small Molecule Drug R&D Plateform, Furong Laboratory Hunan Normal University Changsha China; ^7^ College of Life Sciences University of Chinese Academy of Sciences Beijing China; ^8^ The Affiliated Nanhua Hospital, Clinical Pharmacy Research Institute, Hengyang Medical School University of South China Hengyang China; ^9^ Department of Radiology, The First Affiliated Hospital, Hengyang Medical School University of South China Hengyang China; ^10^ Department of Pharmacology Yongzhou Vocational Technical College Yongzhou China; ^11^ Hubei key Laboratory of Tumor Microenvironment and Immunotherapy China Three Gorges University Yichang China; ^12^ Laboratory of Cardiovascular Disease of Yunnan Province The Affiliated Yan'an Hospital of Kunming Medical University, Cardiovascular System Disease Clinical Medical Research Center of Yunnan Province, Clinical Medicine Center for Cardiovascular Disease of Yunnan Province Kunming China; ^13^ Joint Scientific and Technological Center Pacific State Medical University Vladivostok Russia; ^14^ Department of Histology, Cytology and Embryology Pacific State Medical University Vladivostok Russia; ^15^ Key Laboratory of Translational Cancer Stem Cell Research, Department of Pathophysiology Hunan Normal University Health Science Center Changsha China; ^16^ Hangzhou Institute of Medicine (HIM) Chinese Academy of Sciences Hangzhou China; ^17^ The First Affiliated Hospital, Institute of Translational Medicine Zhejiang University School of Medicine Hangzhou China; ^18^ Global Innovation Institute of Element Science (GIIES‐JLU) The First Hospital of Jilin University Changchun China

**Keywords:** bioresponsive nanomedicine, cuproptosis, ferroptosis, metalloimmunotherapy, PANoptosis

## Abstract

Intracellular metal dyshomeostasis has emerged as a key regulator of specialized regulated cell death (RCD) programs, challenging classical views that regard necrosis as entirely accidental. This review systematically delineates the molecular architecture and translational trajectories underlying metal‐dependent RCD, including iron‐driven ferroptosis, copper‐mediated cuproptosis, and additional emerging modalities such as calcicoptosis, necrosis by sodium overload (NECSO), and the newly designated zincoptosis, mnoptosis, and coptosis. We examined distinct execution mechanisms, ranging from membrane lipid peroxidation and lipoylation‐targeted proteotoxic stress to organelle‐specific bioenergetic failure, which arise following disruption of compartmentalized metal‐buffering networks. To bridge the persistent knowledge gap between foundational metallobiology and clinical application, we evaluated a bidirectional therapeutic framework: exploiting synthetic lethality and metabolic gating via clinical inducers (e.g., sorafenib, elesclomol) to selectively eliminate therapy‐resistant malignancies while deploying targeted pathway inhibitors and systemic agonists (e.g., dipyridamole, omaveloxolone) to limit pathological tissue degeneration in ischemic and neurodegenerative disorders. Recognizing that off‐target multiorgan toxicity and complex in vivo crosstalk among interconnected death pathways (e.g., disulfidptosis and PANoptosis) represent major translational challenges, we assessed advanced materials‐science strategies designed to overcome these barriers. Specifically, we highlighted the integration of single‐atom catalysts, stimuli‐responsive nanomedicines, and biomimetic carriers engineered to spatiotemporally confine catalytic oxidative flux. Finally, we examined the systemic immunological consequences of targeted metal dysregulation, detailing how metal‐induced immunogenic cell death and cyclic GMP‐AMP synthase (cGAS)‐stimulator of interferon genes (STING) pathway hyperactivation reshape immunosuppressive microenvironments and modulate sterile inflammation, thereby enhancing responsiveness to immune checkpoint blockade, providing a definitive molecular blueprint for next‐generation precision therapeutics.

## Highlights

- Metal dyshomeostasis drives canonical and newly designated different regulated cell death programs, challenging classical views that frame necrosis as purely accidental.
- Bidirectional therapeutic strategies exploit tumor‐specific synthetic lethality or apply targeted inhibitors to preserve vulnerable tissues.
- Bioresponsive nanomedicines and single‐atom catalysts spatiotemporally confine oxidative flux, overcoming systemic toxicity to orchestrate interconnected PANoptosis networks.
- Metal‐induced immunogenic cell death and cGAS–STING hyperactivation cooperate systemically with immune checkpoint blockade.

## Graphical abstract

Intracellular metal dyshomeostasis orchestrates distinct regulated cell death programs, including iron‐driven ferroptosis, copper‐mediated cuproptosis, calcicoptosis, newly designated zincoptosis, mnoptosis, and coptosis. This review systematically delineates their molecular architectures—spanning from Sorafenib‐induced lipid peroxidation and lipoylated proteotoxic stress, to mitochondrial calcium uniporter (MCU)‐gated mitochondrial permeabilization. We further evaluate targeted bidirectional therapeutic strategies, highlighting how catalytic bioresponsive nanomedicines spatiotemporally amplify tumor‐specific oxidative flux. Crucially, we examine how metal‐induced immunogenic cell death and Mn^2+^‐driven cyclic GMP‐AMP synthase (cGAS)‐stimulator of interferon genes (STING) hyperactivation systemically reshape immunosuppressive microenvironments, boosting CD8^+^ T cell infiltration to provide a definitive blueprint for translating next‐generation precision metalloimmunotherapeutics.

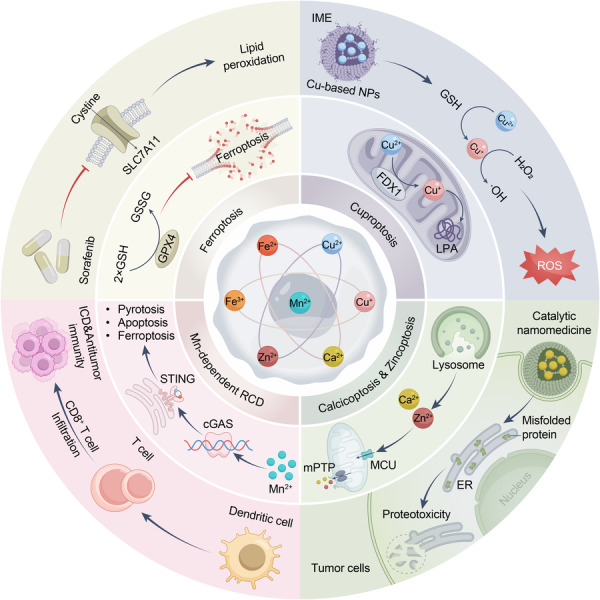

## INTRODUCTION

The understanding of metal‐induced cytotoxicity has undergone a major conceptual shift. Cell death resulting from the intracellular accumulation of transition metals, such as iron, copper, and manganese, or alkaline earth metals, such as calcium and magnesium, was long regarded as accidental necrosis, a passive consequence of acute chemical poisoning or excessive oxidative stress [1, 2]. Over the past decade, this perspective has been superseded by the concept of metal‐dependent regulated cell death (RCD). Within this framework, cell death is understood as a regulated program triggered by disruption of metal homeostasis and amenable to pharmacologic or genetic intervention [3, 4, 5, 6]. Notably, rather than representing uncontrolled cellular failure, metal‐dependent RCD proceeds through identifiable molecular circuits that distinguish it mechanistically from incidental toxicity [7, 8].

**Figure 1:**
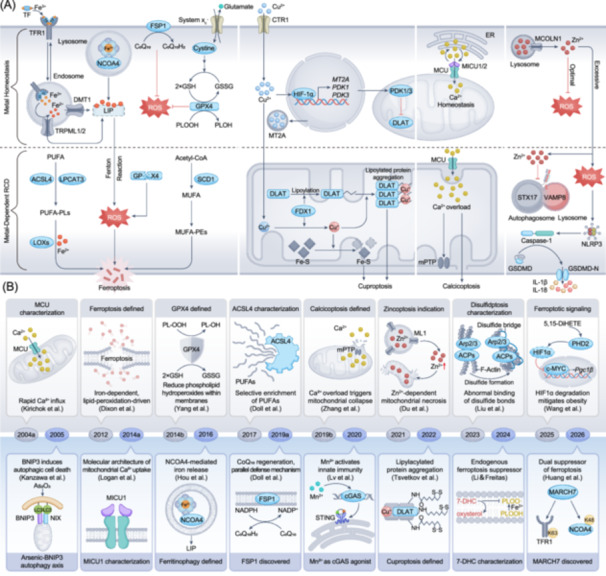
Molecular architecture and conceptual evolution of metal‐dependent regulated cell death (RCD). (A) Mechanistic transition from physiological metal homeostasis to pathological RCD. Under homeostasis, compartmentalized regulatory networks tightly control intracellular metal pools. Glutathione peroxidase 4 (GPX4) and ferroptosis suppressor protein 1 (FSP1) neutralize lipid hydroperoxides to maintain iron‐lipid homeostasis, along with stearoyl‐CoA desaturase 1 (SCD1)‐mediated monounsaturated fatty acid (MUFA) synthesis [26, 27]. Copper is buffered by HIF‐1α‐induced MT2A or mitigated via PDK1/3‐mediated inhibition of DLAT lipoylation [28]. Mitochondrial calcium (Ca^2+^) influx is tightly gated by the MICU1/2‐regulated mitochondrial calcium uniporter (MCU) [11], and optimal lysosomal zinc (Zn^2+^) release via MCOLN1 suppresses reactive oxygen species (ROS) [13]. Exceeding these homeostatic thresholds initiates distinct RCD modalities. Iron accumulation expands the labile iron pool, driving Fenton chemistry and lipoxygenase (LOX)‐catalyzed peroxidation of ACSL4/LPCAT3‐esterified polyunsaturated fatty acids to execute ferroptosis [29, 30]. Copper overload, facilitated by FDX1‐mediated reduction, induces lipoylated DLAT aggregation and destabilizes iron–sulfur (Fe–S) clusters, culminating in cuproptosis [17]. Excessive MCU‐dependent calcium influx triggers mitochondrial permeability transition pore (mPTP) opening, executing calcicoptosis [18, 19]. Unregulated zinc accumulation simultaneously disrupts STX17‐VAMP8‐mediated autophagosome‐lysosome fusion [22] and activates the ROS‐dependent NLRP3/caspase‐1/GSDMD pyroptotic cascade [23]. (B) Timeline detailing the conceptual evolution of structural advancements in metal‐dependent RCD from 2004 to 2026. Early discoveries established the biophysical characterization of MCU [31] and arsenic‐induced BNIP3 autophagy [32]. Subsequent milestones defined the precise molecular mediators and parallel defense networks of ferroptosis (GPX4 [33], NCOA4 [34], ACSL4 [29], FSP1 [27], 7‐DHC [35], and MARCH7 [36]). Recent advancements revealed innate immune activation via the Mn^2+^–cGAS–STING axis [37] along with distinct biochemical definitions of calcicoptosis [38], zincoptosis [24], cuproptosis [17], and disulfidptosis [39], alongside metabolic implications such as HIF1α‐mediated ferroptotic signaling [40], illustrating the continuous characterization of metal‐driven RCD.

The presence of measurable homeostatic thresholds is a key aspect of metal‐dependent RCD. Under physiological conditions, metal ions are part of tightly regulated signaling networks. However, lethal execution is triggered when compartmentalized defense systems, such as the glutathione peroxidase 4 (GPX4)–glutathione (GSH) axis in ferroptosis or MICU1‐regulated gating in mitochondrial calcium handling, are exceeded or functionally impaired [9, 10, 11]. Once buffering systems fail, metal ions no longer function solely as metabolic cofactors but instead act as direct initiators of distinct lethal mechanisms [12, 13] (Figure 1A). For example, ferroptosis exemplifies this principle as an iron‐dependent form of RCD driven by lipid peroxide accumulation within cellular membranes and morphologically characterized by condensed mitochondria with increased membrane density [14, 15, 16]. In contrast, cuproptosis involves copper‐dependent binding to lipoylated tricarboxylic acid (TCA) cycle enzymes, culminating in proteotoxic stress and mitochondrial respiratory collapse [17]. Additional emerging modalities, including calcicoptosis [18, 19, 20, 21] and newly designated zincoptosis‐like cell death [22, 23, 24, 25], associate sustained mitochondrial permeability or autophagy dysfunction with dysregulated calcium and zinc signaling, respectively.

Although the molecular components of metal‐dependent RCD have been defined with increasing precision, clinical translation remains comparatively limited. A principal obstacle arises from a functional dichotomy in therapeutic intent that varies by pathological context. In oncology, the strategy is to exploit the obligate metal reliance of malignant cells, particularly drug‐tolerant persister populations, to activate lethal programs and overcome resistance to apoptosis‐targeted therapies [41, 42, 43]. In contrast, in nonmalignant diseases, such as neurodegeneration or ischemia‐reperfusion injury (IRI), the therapeutic objective is to reinforce buffering capacity and suppress metal‐driven necrotic signaling [44, 45]. The practical implementation of these opposing aims is constrained by the narrow therapeutic index of broad‐spectrum ionophores and the potential for unintended systemic toxicity.

Thus, this review aims to integrate mechanistic insights into metal homeostasis and metabolic dysfunction with therapeutic design. First, we examine the molecular architecture of the principal metal‐dependent modalities, including ferroptosis, cuproptosis, and calcicoptosis, to define tractable intervention points. Second, we evaluate strategies for translating these mechanisms into precision therapeutics, with emphasis placed on approaches that improve selectivity, including stimuli‐responsive nanomedicine [46, 47] and metabolic sensitization [26, 28, 48, 49]. Finally, we consider the systemic implications of metal‐dependent cell death, specifically the induction of immunogenic cell death (ICD) and its capacity to reshape the immune microenvironment. By aligning molecular definition with therapeutic strategy, this review proposes a framework for developing metal‐dependent interventions that balance efficacy with controlled toxicity.

## CONCEPTUAL EVOLUTION OF METAL‐DEPENDENT RCD

The evolution of this field has been shaped by a series of conceptual advances that have clarified the molecular basis of metal‐dependent cell death. Over time, the framework has shifted from viewing intracellular metal accumulation as passive toxicity to recognizing it as a regulated signaling process. A chronological overview of these milestone discoveries across different metal species and cell death modalities is summarized in Figure 1B. In 2004, the biophysical characterization of the mitochondrial calcium uniporter (MCU) by Kirichok and colleagues established the channel as a highly selective ion conduit rather than a carrier, providing a mechanistic foundation for rapid mitochondrial calcium influx [31]. In 2011, identification of the *MCU* gene and its regulatory subunit MICU1 elucidated the molecular architecture of mitochondrial calcium uptake, linking defined genetic components to mitochondrial metal‐induced toxicity [10, 50]. Simultaneously, studies investigating arsenic trioxide have shifted the perception of this agent from a mere environmental poison to a regulated therapeutic, demonstrating that it can induce caspase‐independent autophagic cell death through BNIP3 [32, 51, 52].

In 2012, Dixon et al. introduced the term “ferroptosis” to describe an erastin‐induced, iron‐dependent, nonapoptotic form of RCD [14]. This form of cell death was distinct from apoptosis and necrosis owing to its biochemical dependence on lipid peroxidation and characteristic mitochondrial condensation. In 2014, GPX4 was identified as the central enzymatic suppressor of ferroptosis, responsible for reducing phospholipid hydroperoxides within membranes [33, 53]. Simultaneously, the discovery of NCOA4‐mediated ferritinophagy defined a selective autophagic pathway that mobilizes ferritin‐bound iron to expand the labile iron pool (LIP) required for Fenton chemistry [34, 54]. In 2017, acyl‐CoA synthetase long‐chain family member 4 (ACSL4) characterization further refined the model by demonstrating that the selective enrichment of polyunsaturated fatty acids (PUFAs) within phospholipids dictates ferroptosis sensitivity through substrate availability [29, 30, 55].

Between 2019 and 2020, the conceptual scope of the field broadened beyond iron overload to incorporate novel ion‐driven death mechanisms, immune signaling, and layered antioxidant defenses. Expanding this non‐iron paradigm, calcicoptosis was defined in 2019 as a distinct modality driven by calcium‐induced mitochondrial collapse [18, 21, 38]. Parallel to these insights into metal toxicity, the discovery of ferroptosis suppressor protein 1 (FSP1) during the same year revised the GPX4‐centric model by revealing a glutathione‐independent defense pathway. FSP1 regenerates reduced coenzyme Q_10_ (CoQ_10_) at the plasma membrane, interrupting lipid radical propagation and establishing a parallel protective axis [27, 56]. Subsequent identification of additional compartment‐specific systems, including the GCH1/BH4 [57] and mitochondrial dihydroorotate dehydrogenase (DHODH) pathways [58], demonstrated that resistance to metal‐dependent death reflects distributed and context‐specific redox control rather than reliance on a single node. Building on the discovery that Mn^2+^ sensitizes cyclic GMP‐AMP synthase (cGAS)‐stimulator of interferon genes (STING) signaling, a 2020 study showed that Mn^2+^ is essential for cGAS–STING‐dependent antitumor immunity [37, 59].

The definition of metal‐dependent death has expanded to include additional transition metals and metabolic constraints beginning in 2021. In 2021, Du and colleagues reported that excessive activation of lysosomal ML1/TRPML1 triggers lysosomal Zn^2+^ release, mitochondrial swelling/dysfunction, ATP depletion, and rapid nonapoptotic necrotic death in metastatic melanoma cells, a process they termed “lysozincrosis [60].” This ML1‐dependent, zinc‐driven mitochondrial necrosis provides a mechanistic basis for proposing “zincoptosis” as a zinc‐associated regulated cell death modality [61, 62]. Subsequently, in 2022, Tsvetkov and colleagues defined “cuproptosis” as a copper‐dependent RCD that is mechanistically different from ferroptosis. Rather than lipid peroxidation, cuproptosis is initiated by copper binding to lipoylated TCA cycle enzymes in a ferredoxin 1 (FDX1)‐dependent manner, resulting in proteotoxic stress and mitochondrial dysfunction [17, 63]. In 2023, disulfidptosis was described in cells with high SLC7A11 expression subjected to glucose deprivation, where NADPH depletion drives aberrant disulfide bonding within the actin cytoskeleton [39, 64, 65]. In 2024, 7‐dehydrocholesterol (7‐DHC) was identified as a potent endogenous suppressor, which clarified how cholesterol metabolism modulates membrane susceptibility to peroxidation [35, 66]. Following this, in 2025, 5,15‐DiHETE‐induced ferroptotic signaling was shown to mitigate obesity via HIF1α degradation [40]. In 2026, MARCH7 emerged as a dual ferroptosis suppressor by restricting TFR1 and degrading NCOA4 [36]. Overall, this trajectory reflects the rapid diversification of the field and the delineation of mechanistically distinct and therapeutically actionable targets across metal species.

## FERROPTOSIS: IRON, LIPIDS, AND REDOX

### Overview

**Figure 2:**
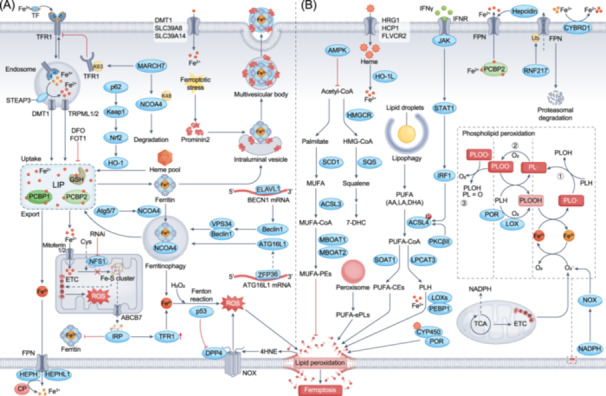
Iron metabolism and lipid peroxidation regulatory networks in ferroptosis. (A) Iron metabolism and labile iron pool (LIP). Extracellular iron is internalized via transferrin receptor 1 (TFR1)‐mediated endocytosis (involving STEAP3 and TRPML1/2) and solute carriers (DMT1, SLC39A8, SLC39A14), increasing cytosolic LIP [104, 105], which is sequestered by DFO and FOT1 [106]. Heme is imported via HRG1, HCP1, and FLVCR2, and intracellular heme is degraded by heme oxygenase‐1 (HO‐1) (regulated via the p62‐Keap1‐Nrf2 axis) to release Fe^2+^ [107, 108]. Intracellular iron homeostasis is maintained by the IRP/IRE system, PCBP1/2 chaperones, and ferritin [109, 110], while NFS1 inhibition disrupts Fe‐S clusters to expand the LIP [111]. NCOA4‐mediated ferritinophagy, facilitated by Atg5/7, and ELAVL1/*BECN1*‐ [112] and ZFP36/*ATG16L1*‐ [113] mediated RNA regulation, further increase the LIP [34, 54], whereas prominin‐2 mediates exosomal ferritin export [84]. Concurrently, MARCH7 restrains iron overload by promoting NCOA4 degradation and restricting TFR1 membrane translocation [36]. Iron efflux is mediated via ferroportin (FPN), coupled with HEPH, HEPHL1, CP, and CYBRD1, and is restricted by hepcidin and RNF217‐dependent ubiquitination [114, 115]. The elevated LIP promotes the nonenzymatic Fenton reaction, generating reactive oxygen species (ROS) [14]. This process, augmented by ROS from the mitochondrial electron transport chain (ETC) fueled via mitoferrin and p53‐DPP4‐regulated NOX, leads to ferroptosis [67, 92]. (B) Membrane remodeling and lipid peroxidation. Concurrently, membrane lipid remodeling regulates ferroptosis sensitivity [29]. Polyunsaturated fatty acids (PUFAs), mobilized via lipophagy, are activated by PKCβII‐phosphorylated or IFNγ‐STAT1‐IRF1‐induced [68] ACSL4 and esterified either into membrane phospholipids (PLH) by LPCAT3, or PUFA‐cholesteryl esters (PUFA‐CEs) by SOAT1 [30, 69]. Peroxisomes synthesize oxidizable PUFA‐ether phospholipids (PUFA‐ePLs) [116]. Conversely, SCD1‐derived monounsaturated fatty acids (MUFAs) are esterified by ACSL3 and MBOAT1/2 into MUFA‐PEs to inhibit lipid peroxidation [26, 70], alongside AMPK‐restricted PUFA supply and HMGCR‐SQS‐derived 7‐DHC. Ferroptosis execution involves PUFA‐phospholipid oxidation into lipid hydroperoxides (PLOOH), catalyzed enzymatically by lipoxygenases (LOXs) in complex with PEBP1, POR, and CYP450 [117, 118].

**Figure 3:**
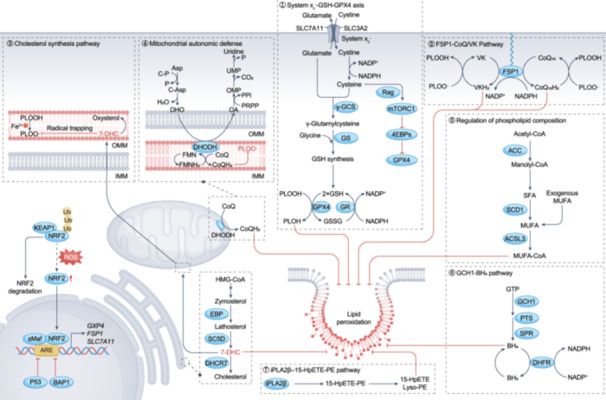
Compartmentalized redox defense network against ferroptosis. Ferroptosis sensitivity is governed by spatially distinct antioxidant axes. In the cytosol, the system x_c_/GSH/GPX4 axis reduces phospholipid hydroperoxides (PLOOH) to alcohols (PLOH) [14, 33]. GPX4 translation is strictly coupled to cystine availability via mTORC1, which phosphorylates 4EBP to relieve translational repression [119]. At the plasma membrane, FSP1 acts as an oxidoreductase, consuming NADPH to regenerate the potent radical‐trapping antioxidants ubiquinol (CoQ_10_H_2_) [56] and vitamin K hydroquinone (VKH_2_) [120], thereby neutralizing lipid peroxyl radicals (PLOO·). In the mitochondria, DHODH provides autonomous protection by reducing ubiquinone to ubiquinol at the inner mitochondrial membrane [58]. Parallel metabolic modules further mitigate lipid peroxidation: the GCH1‐BH_4_ pathway biosynthesizes tetrahydrobiopterin (BH_4_) for direct radical scavenging, sustained by DHFR‐mediated regeneration [121], while iPLA2β repairs membranes by cleaving peroxidized acyl chains (15‐HpETE‐PE) [122]. Furthermore, lipid remodeling restricts oxidizable substrates via the incorporation of monounsaturated fatty acid, synthesized downstream of malonyl‐CoA by ACC, SCD1, and ACSL3 [26], alongside the intrinsic radical‐trapping activity of 7‐DHC [35]. Transcriptionally, NRF2 upregulates core defense genes (*SLC7A11*, *FSP1*, and *GPX4*) [123], whereas the tumor suppressors p53 and BAP1 specifically repress *SLC7A11* transcription [71, 72].

**Figure 4:**
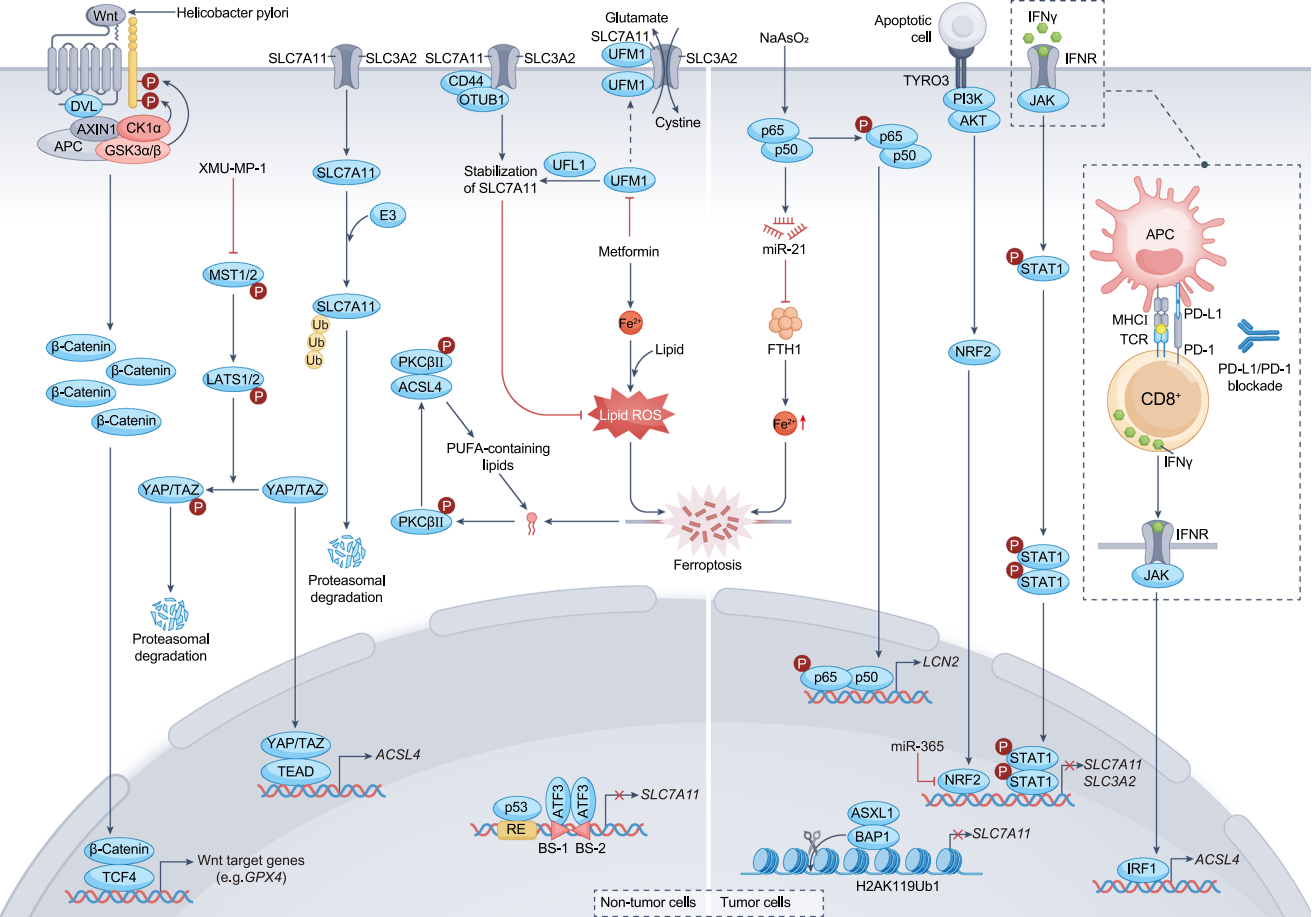
Hierarchical regulation of ferroptotic thresholds via epigenetic and posttranslational modulation. Ferroptosis sensitivity is governed by a multilayered regulatory network. Epigenetically, the BAP1‐ASXL1 complex removes monoubiquitin from histone H2A (H2AK119Ub1) to silence *SLC7A11* transcription, thereby promoting ferroptosis‐mediated tumor suppression. Transcriptionally, p53, which binds to specific p53‐responsive elements, and stress‐induced ATF3 directly repress *SLC7A11*. Conversely, Wnt/β‐catenin signaling upregulates *GPX4* via TCF4, while the TYRO3–PI3K–AKT axis activates NRF2 to promote antioxidant transcription, a process attenuated by miR‐365. Furthermore, YAP/TAZ, activated upon MST1/2 inhibition by XMU‐MP‐1, and IFN‐γ‐induced IRF1 transactivate *ACSL4*. Additionally, NaAsO_2_ activates NF‐κB, which induces *LCN2* and *miR‐21*; the latter represses *FTH1*, expanding the labile iron pool. Post‐translationally, the cystine transporter SLC7A11 is protected from proteasomal degradation via CD44‐OTUB1 interaction and UFMylation. Metformin disrupts this UFMylation, triggering SLC7A11 degradation. Concurrently, PKCβII phosphorylates ACSL4, establishing a positive feedback loop that amplifies lipid peroxidation. Intercellularly, CD8^+^ T‐cell‐secreted IFN‐γ activates the JAK‐STAT1 cascade, synergistically repressing *SLC7A11* and upregulating *ACSL4* to trigger immunogenic tumor ferroptosis.

Beyond its traditional characterization as a terminal execution pathway, ferroptosis functions as a highly integrated metabolic and oxidative hub. This regulated process exhibits a striking pathophysiological duality—it facilitates the physiological clearance of damaged cells while acting as a pathogenic driver in degenerative disorders and a therapeutically exploitable vulnerability in cancer [1, 67]. As systematically delineated in Table 1, ferroptosis is strictly defined as an iron‐dependent, lipid peroxidation‐driven RCD, distinguishing its mechanistic identity from other metal‐induced modalities through its exclusive execution via radical chain propagation within cellular membranes. Mechanistically, the molecular architecture of ferroptotic cell death is organized around a tightly coordinated tripartite system—catalytic expansion of the intracellular LIP, ACSL4‐driven remodeling of membrane lipid bilayers that enrich peroxidation‐susceptible PUFAs [29, 68, 69], and the progressive collapse of spatially compartmentalized antioxidant defense networks [27, 33, 58, 70]. This chapter first examines cellular control of iron homeostasis with the catalytic dynamics governing the LIP to systematically dissect this interconnected regulatory landscape. It subsequently evaluates enzymatic pathways responsible for lipid remodeling and the production of essential peroxidation substrates before analyzing multilayered organelle‐specific redox defense architectures. Finally, this chapter explores epigenetic, transcriptional, and posttranslational regulatory systems that dynamically determine the cellular threshold for ferroptotic execution [71, 72] (Figures 2, 3, 4).

**Table 1:** Comparative molecular architectures and distinctive boundaries across metal‐dependent RCD modalities.

Feature	Ferroptosis	Cuproptosis	Calcicoptosis	NECSO	Zincoptosis^a^	Mnoptosis^a^
Definition	Iron‐dependent, lipid peroxidation‐driven RCD [14]	Copper‐dependent, proteotoxic stress‐driven RCD [17]	Calcium‐dependent, mPTP megachannel opening‐driven RCD [73]	Sodium‐dependent, osmolar collapse‐driven RCD [74]	Zinc‐dependent, mitochondria‐mediated RCD [22, 24]	Manganese‐dependent, enzymatic mismetallation‐driven RCD [75]
Primary Triggering Metal	Iron (Fe^2+^/Fe^3+^) overload	Copper (Cu^+^/Cu^2+^) accumulation	Calcium (Ca^2+^) matrix influx	Sodium (Na^+^) intracellular overload	Zinc (Zn^2+^) cytosolic/mitochondrial surge	Manganese (Mn^2+^) accumulation
Primary Subcellular Target	Plasma membrane; ER; Mitochondria	Mitochondrial matrix	Inner mitochondrial membrane	Plasma membrane; Mitochondria; Lysosomes	Lysosome‐mitochondrial axis	Inner mitochondrial membrane; Cytosol
Core Execution Mechanism	PUFA‐PL peroxidation via ALOXs and POR/CYB5R1; Radical chain propagation [14, 76]	Lipoylated TCA enzyme aggregation; Fe–S cluster destabilization [17]	F‐ATP synthase conformational transition; mPTP megachannel opening; MIMP [73, 77]	Osmolar collapse; Na^+^/K^+^‐ATPase overdrive; Reverse NCX‐mediated secondary Ca^2+^ overload [78, 79]	FDX2/LIAS suppression; STX17‐VAMP8 decoupling (autophagosome‐lysosome fusion block) [22, 24]	Enzymatic mismetallation (Fe displacement in Coq_7_); CoQ biosynthesis arrest [75]
Key Molecular Regulators	GPX4, ACSL4, FSP1, SLC7A11, DHODH, GCH1, 7‐DHC [35]	FDX1, LIAS, DLAT, LIPT1, SIRT2, METTL16 [80]	CypD, F‐ATP synthase (c‐subunit), BAX/BAK, TMEM65 [81]	TRPM4, SEC. 62, NHE1, NEK8, CTSB, NLRP3, GSDMD [82, 83]	FDX2, LIAS, TRPML1, Akt/mTOR, STX17 [22, 24]	Coq_7_, cGAS, STING [37, 75]
Metal Homeostasis Gatekeepers	TFR1, FPN, FTH1/FTL, NCOA4, Prominin2 [84]	SLC31 A1 (CTR1), ATP7A/B, MT1E/X, Atox1	MCU complex, NCLX, RyR, IP3R, MICU1/2 [85, 86]	Na^+^/K^+^‐ATPase, TRPM4, Nav1.5, NHE1, NCX, SGLT2	SLC30A1 (ZnT1), SLC39A7/8/10/14, MCOLN1 [22, 87]	SLC39A8, SLC30A10, MCU [88]
Key Metabolic Dependency	Cystine deprivation; PUFA synthesis; Ether lipid synthesis	Strict TCA cycle/OXPHOS reliance; Glycolysis reprogramming confers resistance [28]	Rapid ATP depletion; NAD^+^ exhaustion	Severe ATP depletion; OXPHOS and glycolysis failure [89]	Glycolysis inhibition (HK2/LDHA); Akt/mTOR suppression [24]	CoQ depletion; Bioenergetic failure
ROS and Redox Context	GSH depletion; Massive lipid ROS burst	Execution is largely ROS‐independent; GSH acts as a Cu^+^ chelator	Localized mtROS burst; ROS‐induced ROS release	ROS‐induced channel hyperactivation; Secondary massive ROS storm	Lysosomal ROS; mtROS surge; GSH depletion	Fenton‐like ROS burst; GSH oxidation
Mitochondrial Impact	Shrunken mitochondria; Increased membrane density; Reduced cristae	Proteotoxic stress; TCA cycle arrest; Membrane potential loss; Vacuolization	Massive matrix swelling; MIMP; Outer membrane rupture	Massive matrix swelling; Outer membrane rupture; mPTP opening; ETC destruction [90]	Drp‐1‐mediated fission [91]; Fragmented networks; ETC inhibition	Respiratory chain failure; Coq_7_ degradation [75]; Cristae disruption
Biochemical Markers	MDA/4‐HNE elevation; PLOOH accumulation [30]; PC‐PUFA_2s_ accumulation [92]	DLAT oligomerization; Fe–S protein loss [17]	mPTP opening [19]; Cytosolic/Matrix Ca^2+^ spike [38]	Cytosolic Na^+^ surge; Severe ATP exhaustion; Cytosolic CTSB leakage [83, 93]	Cytosolic Zn^2+^ surge [22]; FDX2/LIAS depletion [24]	DMQ (demethoxy‐ubiquinone) accumulation [75]; cGAMP production [37]
Morphological Features	Plasma membrane nanopores; Normal nucleus	Mitochondrial membrane rupture; Cell shrinkage	Severe organelle swelling; Membrane rupture	Profound cellular edema; Severe organelle swelling; Necrotic plasma membrane rupture [78]	Lysosomal permeabilization; Mitochondrial fragmentation	Mitochondrial cristae disruption; Intact plasma membrane initially
Pharmacological Inducers	Erastin [14], RSL3 [33], FIN56 [94], IKE [95]	Elesclomol [17], Disulfiram/Cu [96]	Thapsigargin, Ionomycin, Ca^2+^ NPs [38]	Necrocide 1 (NC1) [78], Cinobufagin [82], (‐)‐Englerin A [93], Sodium nanocarriers [97]	ZnO NPs [98], TRPML1 agonists (ML‐SA5) [22], Pyrithione [25]	MnO_2_ nanozymes, High‐valence Mn oxides [75]
Pharmacological Inhibitors	Fer‐1, Lip‐1 [3], DFO [33], Vitamin E [99], 7‐DHC [35]	TTM, Specific Cu chelators, GSH	CsA, NIM811 [85], Ru360	Clotrimazole [78], Cariporide [100], Ranolazine [79], 9‐phenanthrol, Dapagliflozin [83]	CaEDTA, Riluzole	2,4‐diHB [75], CoQ_10_ supplementation [101], PAS‐Na [102]
Immunogenic Features (ICD)	Context‐dependent DAMPs release; CD8^+^ T cell synergy via IFN‐γ [103]	Robust DAMPs emission; mtDNA leakage activates cGAS‐STING	Necrotic pro‐inflammatory DAMP efflux; Macrophage recruitment	Robust DAMPs emission (HMGB1, ATP); NLRP3 inflammasome activation; GSDMD‐dependent pyroptosis	mtDNA release; IFN‐I secretion; NLRP3/Caspase‐1 pyroptotic crosstalk	Direct cGAS‐STING allosteric agonism [37]; Robust IFN‐I burst
Pathway Crosstalk	Autophagy (Ferritinophagy); Pyroptosis; Crosstalk with Disulfidptosis [39]	Disulfidptosis; Ferroptosis	Apoptosis; Necrosis; Ferroptosis	Calcicoptosis (via reverse NCX); Pyroptosis; Apoptosis; Ferroptosis	Pyroptosis; Ferroptosis	Apoptosis; Ferroptosis
Clinical Translational Relevance	Precision oncology targeting therapy‐resistant cells; Mitigation of IRI	Overcoming drug resistance in OXPHOS‐dependent tumors; Wilson's disease pathogenesis	Preserving tissue viability in myocardial infarction and neurodegeneration	Mitigating ischemia‐reperfusion injury and cardiotoxicity; Overcoming chemoresistance in solid tumors	Antibacterial precision therapy; Targeting high‐autophagy metastatic tumors	Metalloimmunotherapy in solid tumors; Mitigating neurotoxicity (Manganism)

Abbreviations: 4‐HNE, 4‐hydroxynonenal; 7‐DHC, 7‐dehydrocholesterol; ACSL4, acyl‐CoA synthetase long‐chain family member 4; AhR, aryl hydrocarbon receptor; AKI, acute kidney injury; ARID1A, AT‐rich interaction domain 1A; ATP, adenosine triphosphate; ATP7B, ATPase copper transporting beta; BAs, bile acids; BrCa, breast cancer; CAR‐T, chimeric antigen receptor T cells; CD8^+^, cluster of differentiation 8; CNS, central nervous system; CoQ_10_, coenzyme Q_10_; Cp, ceruloplasmin; CRC, colorectal cancer; CSF, cerebrospinal fluid; CTLs, cytotoxic T lymphocytes; CTSB, cathepsin B; Cu^2+^, cupric ion; DHCR7, 7‐dehydrocholesterol reductase; DLAT, dihydrolipoamide S‐acetyltransferase; ETC, electron transport chain; FDX1, ferredoxin 1; Fer‐1, ferrostatin‐1; FSP1, ferroptosis suppressor protein 1; FTO, fat mass and obesity‐associated protein (m^6^A RNA demethylase); GCa, gastric cancer; GPX4, glutathione peroxidase 4; GSDMD, gasdermin D; HCC, hepatocellular carcinoma; HDACs, histone deacetylases; HMGB1, high mobility group box 1; ICA, indole‐3‐carboxaldehyde; ICB, immune checkpoint blockade; ICD, immunogenic cell death; IFN‐γ, interferon gamma; IRI, ischemia‐reperfusion injury; Kla, lysine lactylation; LDHA, lactate dehydrogenase A; LuCa, lung cancer; MCT4, monocarboxylate transporter 4; MCU, mitochondrial calcium uniporter; MDA, malondialdehyde; MDM2, mouse double minute 2 homolog; MICU1, mitochondrial calcium uptake 1; MT2A, metallothionein 2A; mtDNA, mitochondrial DNA; m^6^A, N6‐methyladenosine; Nav1.5, voltage‐gated sodium channel 1.5; NC1, necrocide 1; NCX, Na^+^/Ca^2+^ exchanger; NECSO, necrosis by sodium overload; NEK8, Never‐in‐mitosis A‐related kinase 8; NF2, neurofibromin 2; NHE1, Na^+^/H^+^ exchanger 1; NRF2, nuclear factor erythroid 2‐related factor 2; NSCLC, non‐small cell lung cancer; OTUB1, OTU deubiquitinase 1; OvCa, ovarian cancer; OXPHOS, oxidative phosphorylation; p53, tumor protein p53; PANoptosis, synchronized pyroptosis, apoptosis, and necroptosis; PC‐PUFA_2s_, phosphatidylcholine containing two polyunsaturated fatty acid chains; PCa, prostate cancer; PDAC, pancreatic ductal adenocarcinoma; PUFA‐ePLs, polyunsaturated ether phospholipids; PUFAs, polyunsaturated fatty acids; RCD, regulated cell death; ROS, reactive oxygen species; RTA, radical‐trapping antioxidant; SCFAs, short‐chain fatty acids; SEC. 62, SEC. 62 homolog, preprotein translocation factor; SGLT2, sodium‐glucose cotransporter‐2; SLC7A11, solute carrier family 7 member 11; STING, stimulator of interferon genes; TAMs, tumor‐associated macrophages; TCA, tricarboxylic acid cycle; TFRC, transferrin receptor; TLR3, Toll‐like receptor 3; TMAO, trimethylamine N‐oxide; TNBC, triple‐negative breast cancer; Tregs, regulatory T cells; TRPM4, transient receptor potential melastatin 4; YAP, Yes‐associated protein; ZIF‐67, zeolitic imidazolate framework‐67; Zn^2+^, zinc ion; ZnT3, zinc transporter 3; ΔΨm, mitochondrial membrane potential.

a Putative RCD modalities are systematically defined in this work.

### Iron metabolism and LIP

Ferroptosis execution is defined by its dependence on LIP, a transient and redox‐active pool of intracellular iron that exists mainly in the ferrous state (Fe^2+^). In contrast to iron stably incorporated into heme or Fe–S clusters, LIP functions not only as a metabolic cofactor but also as a catalytic engine of lipid peroxidation [1, 4]. The shift from cellular survival to ferroptotic death is tightly shaped by the homeostatic control of redox‐active labile iron. When iron handling and lipid peroxide detoxification are overwhelmed, iron‐dependent lipid hydroperoxides accumulate to lethal levels, initiating a ferroptotic cascade that is mechanistically distinct from caspase‐mediated apoptosis [1, 14, 124]. Accordingly, the disruption of the coordinated network controlling iron uptake, storage, utilization, and export constitutes the principal upstream driver of ferroptosis [125, 126] (Figure 2A). Our group and other researchers have systematically elucidated these iron‐regulatory networks in human diseases [101, 127].

The cytotoxic potential of LIP arises from its capacity to generate reactive oxygen species (ROS) through two convergent mechanisms. First, Fe^2+^ catalyzes the nonenzymatic Fenton reaction (Fe^2+^ + H_2_O_2_ → Fe^3+^ + OH· + OH^−^), producing highly reactive hydroxyl radicals that abstract hydrogen atoms from PUFAs and initiate chain‐propagating membrane oxidation [92, 116, 128, 129]. This oxidative process is further augmented by ROS from the mitochondrial electron transport chain (ETC) and meticulously modulated by tumor suppressors such as p53, which can sequester DPP4 in a nuclear complex, thereby preventing NOX‐dependent lipid peroxidation at the plasma membrane [72, 125, 130, 131]. Reactivity has motivated the development of single‐atom iron catalysts [132] and iron‐based metal‐organic frameworks [133] to recapitulate this oxidative burst for tumor ablation [134]. Second, the LIP serves as an obligate cofactor for iron‐dependent enzymes, including arachidonate lipoxygenases (ALOXs) and cytochrome P450 oxidoreductase (POR), which catalyze stereoselective phospholipid peroxidation [76, 117]. Notably, POR functions as an essential electron donor to aggressively drive this lipid peroxidation process [118]. The requirement for the LIP is demonstrated by iron chelators, such as deferoxamine (DFO) and ferroterminator1 (FOT1), which suppress ferroptosis across experimental systems by sequestering this catalytic fraction [33, 106, 135].

LIP expansion often reflects dysregulated iron import. The canonical entry route involves transferrin receptor 1 (TFR1), which internalizes transferrin‐bound iron via endocytosis. Within the endosome, the metalloreductase STEAP3 reduces intra‐endosomal ferric iron (Fe^3+^) to ferrous iron (Fe^2+^), which is subsequently released into the cytosolic LIP via DMT1 and the endolysosomal channels TRPML1/2 [104, 136]. Oncogenic activation, specifically *RAS* mutations, commonly elevates *TFR* expression, establishing iron dependence that sensitizes malignant cells to ferroptosis [95]. A critical regulatory node within this network is the Fe–S cluster biosynthetic machinery, notably NFS1. NFS1 inhibition induces an iron starvation response mediated by the IRP1/ACO1 switch, paradoxically increasing TFR1 expression while suppressing ferritin synthesis, thus expanding the cytosolic labile iron fraction [111, 137]. Beyond TFR1, alternative uptake pathways contribute in tissue‐specific contexts. At the plasma membrane, solute carriers such as SLC39A8 and DMT1 function as direct cellular Fe^2+^ importers [126, 138], while extracellular heme is internalized through distinct transport pathways mediated by HRG1, HCP1, and FLVCR2. In hepatocytes, SLC39A14 (ZIP14) mediates nontransferrin‐bound iron uptake, promoting ferroptosis in transferrin‐deficient states [105]. In lung carcinoma, erianin‐induced Ca^2+^/CaM activation increases intracellular Ca^2+^ and Fe^2+^ levels, consistent with LVDCC‐associated iron uptake and ferroptosis induction [139]. In mesenchymal tumor states, CD44 mediates a noncanonical iron‐bound hyaluronan endocytosis pathway, linking EMT‐associated metabolic and epigenetic plasticity to iron‐dependent ferroptotic vulnerability [140, 141].

Once internalized, intracellular iron chaperones meticulously manage this pool; specifically, PCBP1 delivers Fe^2+^ to the ferritin and heme pools for storage and utilization, while PCBP2 routes Fe^2+^ to ferroportin (FPN) for cellular efflux [142, 143]. Concurrently, LIP dynamics are governed by ferritinophagy, a regulated storage‐release mechanism [104, 144]. Ferritin (FTH1/FTL) normally confines excess iron in a redox‐inert form [109]; however, the cargo receptor NCOA4, structurally facilitated by core autophagy machinery proteins such as Atg5 and Atg7 [34, 124], selectively binds ferritin and directs it to lysosomes for autophagic degradation, rapidly mobilizing iron into the LIP [34, 54, 124, 130]. This axis is subject to multilayered control. Posttranslationally, *O*‐GlcNAcylation of FTH1 sterically restricts NCOA4 interaction, and its removal permits accelerated ferritinophagy [145]. Recently, our group demonstrated that the E3 ligase MARCH7 suppresses ferroptosis by ubiquitylating NCOA4 for proteasomal degradation and limiting TFR1 membrane translocation, dually restricting intracellular iron accumulation [36]. At the RNA level, ELAVL1/HuR promotes BECN1‐dependent autophagy by stabilizing or enhancing the translation of *BECN1* mRNA [112], whereas ZFP36/TTP suppresses autophagy by promoting *ATG16L1* mRNA decay [113]. Moreover, environmental cues further modulate this circuit; hypoxia suppresses NCOA4 to enhance resistance [113, 146]. Conversely, arsenic or copper exposure induces NCOA4 expression and promotes iron‐dependent toxicity [110, 147]. Additionally, salinomycin sequesters iron within lysosomes, generating a cytosolic iron‐deficiency signal that paradoxically accelerates ferritin degradation, culminating in lysosomal iron overload and cell death [148].

Beyond ferritin turnover, heme catabolism provides a substantial source of labile iron. The transcription of *heme oxygenase‐1 (HO‐1)* is tightly orchestrated by the p62‐Keap1‐Nrf2 regulatory cascade, where p62‐mediated inhibition of Keap1 leads to Nrf2 stabilization [107, 149, 150]. Heme oxygenase‐1 (HO‐1) degrades heme to yield Fe^2+^, carbon monoxide, and biliverdin. Although HO‐1 is frequently considered cytoprotective, excessive activation, for example, following doxorubicin or withaferin A exposure, can exceed the buffering capacity and liberate sufficient iron to drive heme‐associated ferroptosis [108, 131, 149]. This mechanism is particularly relevant in cardiomyopathy, where NRF2‐dependent HO‐1 induction promotes mitochondrial iron accumulation [151]. Unlike iron mobilization, cells deploy export pathways to reduce LIP. FPN mediates iron efflux, operating in tandem with membrane ferroxidases, such as hephaestin (HEPH), HEPHL1, and ceruloplasmin (CP), to oxidize exported Fe^2+^ back to Fe^3+^, while the ferrireductase CYBRD1 dynamically modulates the iron redox state. Conversely, this export axis is strictly restricted by hepcidin and RNF217‐dependent ubiquitination, which target FPN for proteasomal degradation [114, 115]. The loss of FPN contributes to neurodegenerative phenotypes such as Alzheimer's disease [114]. Prominin‐2 supports ferritin export via exosomal pathways [84], and the NUPR1–LCN2 axis limits intracellular iron availability to suppress ferroptosis in pancreatic and hepatic malignancies [152, 153].

LIP regulation also extends to mitochondrial and nuclear compartments. Mitochondria represent the central hubs of iron utilization, and perturbation of proteins, such as CISD1 (mitoNEET) or increased import via mitoferrin destabilizes mitochondrial iron homeostasis, thereby coupling metabolic dysfunction to ferroptotic sensitivity [154]. This vulnerability is often amplified by mitochondrial Ca^2+^ overload through the MCU complex, creating a cooperative toxicity axis [155, 156]. At the transcriptional level, NRF2 promotes ferroptosis resistance by inducing iron‐storage and iron‐efflux genes, including FTH1/FTL and FPN1/SLC40A1 [150, 157]. However, dysregulation of the NRF2‐dependent HERC2–VAMP8 axis can impair ferritinophagy/autophagic flux, resulting in ferritin or apoferritin accumulation [158]. Epigenetic regulators such as HDAC3, along with metal‐regulatory proteins like COMMD10, further refine systemic iron control through the modulation of hepcidin and ceruloplasmin expression, respectively [115, 159]. Collectively, LIP functions as a central rheostat for ferroptosis, determined by the integrated balance of uptake, mobilization, and export.

### Membrane remodeling and lipid peroxidation

Although the LIP provides the catalytic impetus, ferroptosis is ultimately governed by the availability and molecular architecture of specific lipid substrates. Evidence indicates that ferroptosis does not occur from indiscriminate oxidative stress or free fatty acid oxidation but rather from the selective peroxidation of PUFA‐containing phospholipids embedded within membranes [29, 136]. To sustain this peroxidizable substrate pool, free PUFAs, such as arachidonic acid (AA), linoleic acid (LA), and docosahexaenoic acid (DHA), are buffered within lipid droplets and can be mobilized via lipophagy under specific conditions [125, 160]. The vulnerability of these lipids is determined by bis‐allylic hydrogen atoms positioned between double bonds, which possess low bond dissociation energy [117] and are readily abstracted. Oxidized phosphatidylethanolamines (PEs) have emerged as principal death signals, specifically species esterified with AA (C20:4) or adrenic acid (C22:4) at the *sn*−2 position [29, 30]. Lipidomic analyses further identify di‐PUFA phospholipids, including PC(20:4/20:4), as potent ferroptotic lipid substrates that promote mitochondrial ROS generation and lipid peroxidation through interactions with the mitochondrial ETC [92]. Additionally, PUFA‐ePLs, synthesized via AGPS and FAR1, represent an independent substrate reservoir that enhances ferroptosis susceptibility, particularly in neuronal tissues and renal carcinomas [116]. The organelle‐specific biology and multifaceted mitochondrial dynamics of ferroptosis are systematically evaluated elsewhere [161, 162].

Accordingly, cellular sensitivity to ferroptosis reflects membrane lipid composition, which is actively shaped by defined enzymatic pathways. The intricate metabolic routing of these lipids, from biogenesis and membrane incorporation to enzymatic and nonenzymatic peroxidation, is depicted in Figure 2B. ACSL4 serves as a key regulatory node by preferentially activating long‐chain PUFAs to their CoA derivatives [125, 163]. Lysophosphatidylcholine acyltransferase 3 (LPCAT3) subsequently incorporates these activated acyl chains into phospholipids [30]. In parallel with PUFA incorporation into phospholipids, sterol O‐acyltransferase 1 (SOAT1) provides an alternative esterification route by channeling PUFA‐CoAs into PUFA‐CEs, thereby broadening the pool of oxidizable lipids [164, 165]. Genetic deletion of ACSL4 or LPCAT3 markedly reduces oxidizable membrane substrates and confers resistance to GPX4 inhibition despite preserved iron availability [55]. ACSL4 activity is further modulated by context‐dependent signaling inputs. PKCβII phosphorylates ACSL4 to enhance its catalytic function within a reinforcing loop [69], whereas the NF2‐YAP pathway regulates its transcription in response to cell density [166]. Immune‐derived signals via the interferon gamma (IFN‐γ)‐driven STAT1‐IRF1 axis increase ACSL4 expression, thereby linking adaptive immune signaling to ferroptotic susceptibility in tumor cells [68].

Once a peroxidation‐prone lipid environment is established, oxidation initiation proceeds through coordinated enzymatic and nonenzymatic mechanisms. Iron‐dependent lipoxygenases, specifically ALOX15 in complex with PEBP1, catalyze the oxygenation of PUFA‐PEs to generate 15‐HpETE‐PE [167], a defined prodeath species [168]. In environments with limited lipoxygenase activity, POR and CYB5R1 provide alternative initiation routes by facilitating electron transfer reactions that generate reactive intermediates targeting membrane lipids, independent of their canonical electron‐accepting partners, such as cytochrome P450 (CYP450) enzymes [76, 118]. Concurrently, the ROS pool required to drive lipid peroxidation can be modulated at the plasma membrane by NADPH oxidase (NOX) complexes, which are under tight negative regulation by the p53‐DPP4 axis acting as a brake on lipid peroxidation [67, 169]. Regardless of the initiating enzyme, execution is predominantly propagated through iron‐catalyzed radical chain reactions within the bilayer (Figure 2B, for the detailed kinetic cycle mapping the phospholipid peroxidation cascade, explicitly highlighting the nonenzymatic Fenton‐mediated Fe^2+^/Fe^3+^ cycling at this stage) [99, 170, 171]. Radical‐trapping antioxidants (RTAs), including ferrostatin‐1 (Fer‐1), suppress ferroptosis primarily by intercepting lipid‐radical chain propagation rather than by directly blocking upstream lipoxygenase activity [170, 171, 172]. Although the endoplasmic reticulum (ER) represents the principal site of lipid remodeling and oxidation [173], mitochondrial phospholipids, especially cardiolipin, constitute critical execution substrates in pathologies such as cardiomyopathy [108, 174].

To limit this vulnerability, cells remodel membrane composition toward a resistant phenotype. Monounsaturated fatty acids (MUFAs), exemplified by oleic acid, competitively displace PUFAs from phospholipids and thereby reduce oxidizable content [175]. This adaptive remodeling is mediated by stearoyl‐CoA desaturase 1 (SCD1) and acyltransferases, including ACSL3 and MBOAT1/2 [70, 176]. Inhibition of SCD1 disrupts this MUFA‐enriched state and restores ferroptosis sensitivity in several tumor models [26, 177]. These lipidic defense systems are intricately linked to the upstream mevalonate and cholesterol biosynthesis pathways, where enzymes, such as HMG‐CoA reductase (HMGCR) and squalene synthase (SQS), orchestrate the production of squalene, an endogenous lipophilic antioxidant [3, 94]. Recently, 7‐DHC has been identified as a potent endogenous suppressor. As a cholesterol precursor, 7‐DHC integrates into membranes and preferentially undergoes oxidation, thereby intercepting peroxyl radicals and protecting adjacent phospholipids [35, 66]. Simultaneously, the phospholipase iPLA2β contributes to membrane repair by hydrolyzing peroxidized acyl chains, thereby removing oxidized moieties and elevating the death threshold [122]. If these repair mechanisms are overwhelmed, accumulating lipid peroxides decompose into highly reactive and toxic aldehydes, most notably 4‐hydroxynonenal (4‐HNE) and malondialdehyde (MDA), which act as terminal downstream executioners of cell death [106, 147, 160, 178].

The availability of substrates for lipid peroxidation is also tightly linked to the cellular metabolic state, coupling energy metabolism with ferroptotic competence. During glucose deprivation, AMPK activation suppresses acetyl‐CoA carboxylase, reducing de novo fatty acid synthesis and limiting PUFA supply required for ferroptosis [94, 179]. Conversely, hypoxia‐inducible factor 2α (HIF‐2α) induces HILPDA expression, promoting selective enrichment of PUFA‐containing phospholipids and generating a hypersensitive phenotype in clear cell renal cell carcinoma [180]. Acidic tumor microenvironments (TME) further influence lipid trafficking and can promote noncanonical PUFA peroxidation [160]. Thus, ferroptotic lipid remodeling is an active enzymatic–metabolic program: ACSL4 and LPCAT3 shape the pool of peroxidizable PUFA‐phospholipids [29, 181], whereas AMPK–ACC and PI3K–AKT–mTOR–SREBP/SCD1 signaling tune the balance between PUFA‐driven lethality and MUFA‐mediated protection [177, 179].

### Compartmentalized redox defense network

Cellular susceptibility to ferroptosis is governed by a multilayered antioxidant defense architecture that monitors and neutralizes lipid peroxides. Although early work positioned GPX4 as the singular gatekeeper of ferroptosis, subsequent studies have defined a compartmentalized network composed of spatially distinct yet partially redundant antioxidant axes operating in the cytosol, plasma membrane, and mitochondria [3, 27, 56, 58]. These axes are coordinated through transcriptional and posttranslational regulation, enabling adaptation to changing metabolic conditions. The integrated molecular architecture of these spatially distinct antioxidant modules is comprehensively mapped in Figure 3.

The primary cytosolic defense is the canonical system x_c_^−^/GSH/GPX4 axis, which reduces phospholipid hydroperoxides to phospholipid alcohols (PLOH) [14, 33]. GPX4 is unique among mammalian glutathione peroxidases in directly reducing membrane‐embedded phospholipid and cholesterol hydroperoxides, and its full catalytic efficiency relies on the active‐site selenocysteine (Sec) residue [182]. Substitution of Sec with cysteine predisposes the enzyme to irreversible overoxidation to sulfonic acid under peroxide stress, leading to loss‐of‐function and ferroptotic cell death, a vulnerability particularly evident in metabolically active parvalbumin‐positive interneurons [182]. GPX4 activity is tightly linked to intracellular GSH levels and to cystine import through the system x_c_^−^ antiporter (SLC7A11/SLC3A2) [183, 184, 185]. This pathway is tightly regulated; for example, GPX4 synthesis is coupled to cystine availability through the mTORC1–4EBP axis, such that nutrient starvation suppresses translation independently of GSH concentration [119]. Additionally, protein interactions and degradation pathways modulate stability. BECN1 binds SLC7A11 to inhibit cystine transport [186], whereas excess Cu^2+^ can associate with GPX4, promoting aggregation and TAX1BP1‐mediated autophagic degradation [187]. Comprehensive literature provides further mechanistic depth regarding GPX4 regulation and related metabolic vulnerabilities [183, 188].

Notably, the FSP1 pathway provides plasma membrane‐localized protection [27, 56]. FSP1 functions as an NADPH‐dependent oxidoreductase that regenerates reduced coenzyme Q_10_ (CoQ_10_/ubiquinol), which acts as a lipophilic radical‐trapping antioxidant within the membrane to interrupt lipid peroxidation propagation [27, 56]. Proper activity depends on subcellular localization; that is, N‐terminal myristoylation anchors FSP1 to the plasma membrane, and emerging evidence indicates that phase separation into biomolecular condensates further regulates its function [27, 189]. FSP1 also supports a noncanonical vitamin K cycle by reducing vitamin K to its hydroquinone form (VKH_2_), providing an additional radical‐trapping mechanism independent of CoQ_10_ [120]. This compensatory axis is particularly relevant in KEAP1‐deficient lung cancers, where NRF2‐driven FSP1 upregulation offsets GPX4 inhibition [123].

Mitochondria maintain dedicated ferroptosis defense mechanisms considering their central role in ROS generation and iron metabolism. The double‐membrane architecture of mitochondria limits the access of cytosolic GPX4/FSP1 to mitochondrial inner‐membrane lipid peroxides, necessitating mitochondria‐localized ferroptosis‐defense systems such as GPX4^mito^ and DHODH [58, 190, 191]. DHODH, localized to the outer surface of the inner mitochondrial membrane, links pyrimidine synthesis to antioxidant defense by reducing mitochondrial ubiquinone to ubiquinol, thereby neutralizing lipid peroxides within the organelle. Loss of DHODH sensitizes cells to ferroptosis even when cytosolic defenses remain intact [58]. A mitochondrial GPX4 isoform containing a targeting sequence further protects lipids, such as cardiolipin [53], and its transcription can be enhanced by selenium supplementation through TFAP2c and Sp1 to confer neuroprotection under ischemic conditions [192]. Furthermore, the mitochondrial thioredoxin (Trx2) system functions as an independent redox node, with certain metal ions, including arsenic, preferentially oxidizing the mitochondrial thioredoxin pathway rather than the cytosolic GSH pool [193].

Beyond enzymatic detoxification, cells use complementary metabolic and structural strategies. The GTP cyclohydrolase 1 (GCH1) pathway controls the synthesis of tetrahydrobiopterin (BH_4_), an endogenous radical‐trapping antioxidant that protects phospholipids containing two polyunsaturated fatty acid chains [57, 121]. This pathway operates independently of GPX4 and FSP1, and its inhibition synergizes with GSH depletion to promote lipid peroxidation [194]. The phospholipase iPLA2β contributes to membrane repair by cleaving oxidized acyl chains from phospholipids, thereby removing damaged species and increasing the death threshold [122]. These defense modules are primarily coordinated by NRF2‐dependent transcriptional programs that regulate *SLC7A11*, *GPX4*, *FSP1* [123, 150], and related genes. Furthermore, hormonal signaling further modulates susceptibility; androgen and estrogen receptors upregulate O‐acyltransferases (MBOAT1/2), promoting the incorporation of monounsaturated fatty acids and dilution of oxidizable substrates [70]. Accordingly, effective ferroptosis induction in resistant cells often requires simultaneous targeting of multiple nodes within this adaptive network.

### Hierarchical regulation of ferroptotic thresholds

Ferroptosis susceptibility is not simply a passive consequence of substrate availability but is tightly controlled by a hierarchical regulatory network. This regulatory architecture extends from chromatin remodeling and transcriptional control to posttranscriptional RNA processing and rapid post‐translational modifications (PTMs) of core effector proteins [195, 196]. Collectively, these layers modulate the three principal components of ferroptosis, the cystine/glutamate antiporter (system x_c_^−^), lipid remodeling enzymes, and iron homeostasis machinery, and determine the threshold at which cellular stress progresses to irreversible death. Other comprehensive reviews elegantly summarize the intricate molecular machinery governing these ferroptotic thresholds [3, 15, 136]. The integrated map of these epigenetic, transcriptional, and post‐translational regulatory nodes is illustrated in Figure 4.

Epigenetic regulation, including histone modification and DNA methylation, establishes basal ferroptosis sensitivity by controlling antioxidant and metabolic gene accessibility. The tumor suppressor BRCA1‐associated protein 1 (BAP1) functions within the polycomb repressive deubiquitinase complex as a key epigenetic regulator. BAP1 removes monoubiquitin from histone H2A (H2Aub) at the *SLC7A11* promoter; notably, this deubiquitination represses *SLC7A11* expression, thereby limiting cystine uptake and enhancing ferroptosis sensitivity [71, 130]. Similarly, p53 regulates this threshold in a manner dependent on its acetylation state. The acetylation‐defective p53^3^KR mutant retains the ability to repress SLC7A11 transcription despite loss of apoptotic activity, indicating that distinct posttranslational configurations of p53 determine its ferroptosis‐related output [72, 131, 197]. DNA methylation further contributes to gene silencing. In hepatocellular carcinoma (HCC), hypermethylation of the *LIFR* promoter reduces its expression, leading to increased levels of the iron‐sequestering protein lipocalin‐2 (LCN2) and consequent resistance through iron depletion [152]. Additionally, chronic exposure to toxins such as arsenic may induce irreversible ferroptotic damage, potentially driven by mechanisms such as the promoter methylation of *GPX4*, which prevents its functional rescue [198]. Histone deacetylase HDAC3 also influences systemic iron regulation by maintaining Hippo pathway activity to prevent YAP‐mediated repression of hepcidin transcription [115].

Downstream of chromatin remodeling, multiple signaling pathways converge on transcription factors to adjust the ferroptosis machinery in response to stress [199]. In addition to p53 and BAP1, ATF3 functions as a stress‐inducible repressor that binds the SLC7A11 promoter to inhibit system x_c_^−^ activity [200]. Under therapeutic stress, ATM and IFN‐γ‐activated STAT1 cooperatively suppress SLC7A11, creating synthetic lethal vulnerability [201]. The expression of ACSL4, which governs the incorporation of oxidizable polyunsaturated fatty acids into membranes, is also tightly regulated. Activation of YAP following low cell density or NF2 loss increases transcription of *ACSL4* and *TFRC* [166]. Similarly, CD8^+^ T‐cell‐derived IFN‐γ activates the JAK1–STAT1–IRF1 axis, and IRF1 directly binds the ACSL4 promoter to enhance lipid peroxidation capacity [68]. Conversely, resistance commonly involves induction of antioxidant defenses. The Wnt/β‐catenin pathway promotes resistance through TCF4‐mediated upregulation of GPX4 [202], whereas TYRO3 activates PI3K/AKT signaling to enhance NRF2‐driven transcription of SLC7A11 and FTH1 [203].

Posttranscriptional control provides an additional rapid layer of regulation through modulation of mRNA stability. The RNA‐binding protein ZFP36 (Tristetraprolin) limits ferroptosis by destabilizing ATG16L1 mRNA, thereby restraining ferritinophagy and preventing iron overload [113]. Conversely, ELAVL1 (HuR) stabilizes BECN1 transcripts and promotes autophagy‐dependent ferroptosis [112]. Additionally, EGFR‐driven ALKBH5 nuclear retention prevents m^6^A‐dependent GCLM mRNA decay, thereby sustaining GSH synthesis and suppressing ferroptosis [204]. Non‐coding RNAs further refine this network. *miR‐21* enhances ferroptosis by repressing FTH1 [205], whereas cancer‐associated fibroblasts release exosomes containing *miR‐522* to suppress ALOX15 translation in tumor cells [206]. The long non‐coding RNA *MT1DP* increases oxidative stress by stabilizing miR‐365, which represses the translation of the antioxidant regulator NRF2 [207].

The most immediate regulatory layer involves PTMs that alter protein stability, localization, or catalytic activity. Ubiquitination is central to proteostatic control [110]. The deubiquitinase OTUB1 stabilizes SLC7A11 by preventing proteasomal degradation [208], while UFMylation has been identified as a PTM required for SLC7A11 maintenance [209]. In contrast, GPX4 can be targeted for degradation by small molecules such as DMOCPTL through ubiquitin‐dependent pathways [210] or by Cu^2+^‐induced aggregation followed by TAX1BP1‐mediated autophagy [187]. Iron metabolism is similarly regulated; the E3 ligase HERC2 controls NCOA4 abundance and ferritinophagy [158], and NEDD4 ubiquitylates VDAC2/3 to constrain mitochondrial ferroptosis signaling [211].

Beyond protein turnover, phosphorylation and related PTMs function as activity switches. PKCβII phosphorylates ACSL4 at Thr^328^, promoting dimerization and enhancing enzymatic activity within a reinforcing loop that augments lipid peroxidation [69]. Conversely, AMPK‐mediated phosphorylation of BECN1 promotes formation of a complex with SLC7A11 that directly suppresses cystine transport [186]. Additional modifications include O‐GlcNAcylation of ferritin heavy chain at Ser^179^, whose removal is required for NCOA4 binding and ferritin degradation [145], and lactylation of NUDT21, which alters FDX1 mRNA processing and confers resistance in lactate‐enriched environments [212]. Collectively, these epigenetic and post‐translational controls define the plasticity of the ferroptotic threshold and identify multiple intervention points for therapeutic modulation.

### Section summary

Rather than operating as a simple linear biochemical cascade, ferroptosis is now understood as a highly orchestrated and spatiotemporally organized redox network [14]. The molecular architecture of this lethal execution program is organized around a tightly coordinated tripartite system: catalytic expansion of the intracellular LIP, enzymatic incorporation of peroxidizable PUFAs into membrane phospholipids [29], and progressive collapse of spatially segregated antioxidant defenses spanning the cytosol, mitochondria, and plasma membrane. Conceptually, this tripartite convergence constitutes the “FerroLipid,” shattering the classical paradigm that iron and lipid metabolism operate merely as parallel cascades. Instead, it defines them as an integrated core co‐regulatory unit where the pathological collision of an unstable labile iron pool, vulnerable PUFA‐PLs, and collapsed antioxidant defenses synergistically detonates an irreversible “ferroptotic explosion” [213]. Translating this mechanistic triad into precision oncology, however, is strongly constrained by the structural and metabolic plasticity of malignant cells. Tumors rarely rely on a single canonical GPX4‐dependent defense node [33]. Instead, they evade lethal lipid peroxidation by upregulating redundant, compartment‐specific radical‐trapping systems, including plasma membrane‐associated FSP1 [27] or mitochondrial DHODH [58], and by remodeling lipid bilayers to enrich oxidation‐resistant species such as MUFAs and 7‐DHC [35]. This adaptive flexibility exposes a major translational bottleneck: blunt pharmacological inhibition of essential housekeeping enzymes such as GPX4 frequently fails to eliminate resilient tumors and can consistently produce severe, dose‐limiting toxicities, including acute kidney injury (AKI) [53] and irreversible neurodegeneration [182].

To overcome this clinical barrier, future precision strategies must move beyond broad monotherapies toward context‐dependent synthetic lethality and coordinated epigenetic modulation. Because the cellular ferroptotic threshold is governed by hierarchical chromatin remodeling [71], complex transcriptional circuitry, and dynamic PTMs, therapeutic approaches must strategically exploit these multilayered regulatory networks together with intrinsic oncogenic liabilities. By coupling tumor‐specific susceptibilities, such as metabolic iron hyperdependence driven by oncogenic RAS mutations [14] or pronounced redox addiction associated with SLC7A11 overexpression, with immunotherapy‐based combinations targeting system x_c_^‐^ or cystine metabolism [103], emerging therapies may selectively dismantle the tumor's antioxidant defenses. Ultimately, this integrated mechanistic strategy aims to breach the elevated ferroptotic threshold of malignant cells while preserving physiological homeostasis in healthy tissues.

## CUPROPTOSIS: BIOENERGETIC COLLAPSE AND PROTEOTOXICITY

### Overview

Cuproptosis represents a distinct RCD modality triggered by intracellular copper overload, conceptually diverging from the lipid‐centered framework characteristic of ferroptosis [14] and the caspase‐dependent cascades underlying apoptosis. Rather than merely producing indiscriminate oxidative injury, cuproptosis represents a respiration‐dependent metabolic vulnerability in which copper binds selectively to lipoylated mitochondrial TCA‐cycle proteins, promoting their aggregation, Fe–S cluster protein loss, and lethal proteotoxic stress [17, 214]. Mechanistically, this susceptibility requires active mitochondrial respiration and proceeds through the FDX1‐mediated reduction of Cu^2+^ to Cu^+^. The resulting highly reactive cuprous ions directly bind to lipoylated TCA cycle enzymes, specifically dihydrolipoamide *S*‐acetyltransferase (DLAT), thereby inducing aberrant protein oligomerization, widespread destabilization of Fe–S cluster proteins, and eventual mitochondrial collapse [17]. As this pathway is tightly constrained by cellular metabolic flux and substrate availability, this chapter first outlines the core molecular execution machinery centered on the FDX1‐lipoylation axis. It then examines metabolic gating processes, particularly glycolytic reprogramming [215], along with microenvironmental checkpoints such as hypoxia that determine cellular resistance [28]. Finally, the chapter evaluates mechanistic crosstalk linking cuproptosis, ferroptosis, and iron/heme metabolism [216], highlighting shared biochemical vulnerabilities, most prominently glutathione depletion [96]. These align with the intricate copper homeostatic networks our group has comprehensively mapped recently [63, 217, 218].

### The FDX1‐lipoylation axis

**Figure 5:**
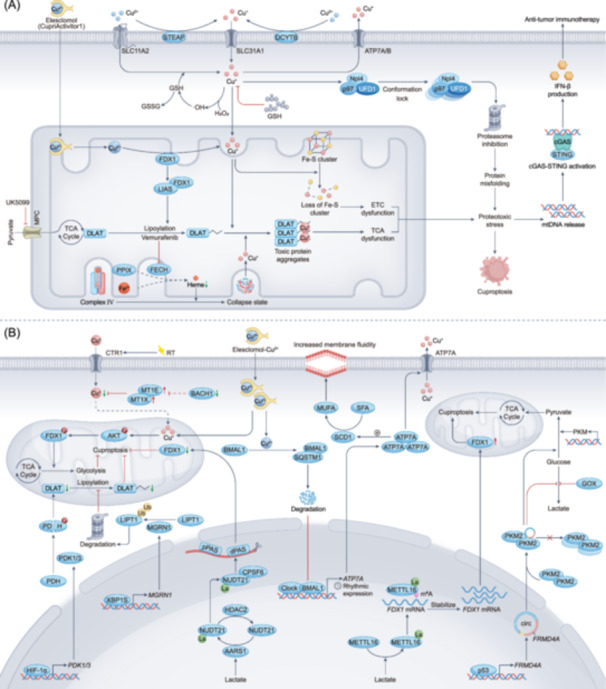
Molecular architecture and metabolic gating of cuproptosis. (A) Extracellular Cu^2+^ is reduced by STEAP/DCYTB and imported via SLC31A1, or transported intracellularly by ionophores (e.g., elesclomol) [17, 63]. Cytosolic Cu^+^ is buffered by glutathione (GSH), whereas excess Cu^+^ interacts with the NPL4/p97/UFD1 complex, inducing proteasome inhibition and protein misfolding. Within the mitochondrial matrix, FDX1 reduces Cu^2+^ to reactive Cu^+^ and facilitates LIAS‐dependent lipoylation of tricarboxylic acid (TCA) cycle enzymes, mainly DLAT [17]. Cu^+^ directly binds lipoylated DLAT, triggering aberrant oligomerization and concurrent destabilization of Fe–S cluster proteins [17]. These events precipitate dysfunction of the ETC and the TCA cycle, culminating in proteotoxic stress, cuproptosis, and mitochondrial membrane rupture [17, 214]. The released mitochondrial DNA (mtDNA) activates the cGAS–STING pathway to elicit antitumor immunity [220]. Concurrently, heme deficiency impairs Complex IV assembly, leaving unincorporated copper to provoke DLAT oligomerization and execute cuproptosis [216]. (B) Cuproptosis sensitivity necessitates active mitochondrial oxidative phosphorylation [17]. Tumor cells become resistant via glycolytic reprogramming: hypoxia‐induced HIF‐1α upregulates PDK1/3 to phosphorylate and degrade DLAT [28], while the XBP1s–MGRN1 axis ubiquitinates lipoyltransferase 1 (LIPT1) to suppress lipoylation. Additionally, AKT phosphorylates and inhibits FDX1, and BACH1 downregulation derepresses metallothionein (MT1E/MT1X) to sequester intracellular copper [221]. Conversely, radiotherapy enhances CTR1‐mediated copper influx [221]. Glucose oxidase (GOx) restricts glycolysis, and p53‐driven *circFRMD4A* inhibits PKM2 [215], both redirecting metabolic flux toward mitochondrial oxidation to restore cuproptosis sensitivity. Lactate exerts dual epigenetic control: METTL16 lactylation stabilizes *FDX1* mRNA via m^6^A modification, whereas NUDT21 lactylation promotes distal polyadenylation to degrade *FDX1* transcripts [212]. Finally, the circadian regulator BMAL1 drives ATP7A‐mediated copper efflux and SCD1‐dependent lipid desaturation to preserve membrane fluidity. However, excess copper triggers SQSTM1‐dependent autophagic degradation of BMAL1.

Cuproptosis delineation marks a conceptual departure from the lipid‐centered framework of ferroptosis and the caspase‐dependent cascades characteristic of apoptosis. As comparatively outlined in Table 1, cuproptosis is defined as a copper‐dependent, proteotoxic stress‐driven RCD modality initiated by intracellular copper overload within the mitochondrial matrix. It is mechanistically distinguished by the specific aggregation of lipoylated tricarboxylic acid (TCA) cycle enzymes and concurrent Fe–S cluster destabilization, rather than lipid peroxidation or protease activation. In contrast to oxidative stress‐driven processes in which metal ions promote indiscriminate macromolecular damage, cuproptosis is triggered by the selective binding of copper to lipoylated enzymes of the TCA cycle [214, 219]. This interaction converts mitochondrial respiration into a conditional liability, a process governed by the FDX1–lipoylation axis. The core molecular architecture defining this unique cuproptotic cascade, from copper import to proteotoxic execution, is depicted in Figure 5A.

The execution of this pathway is tightly dependent on FDX1, a mitochondrial Fe–S cluster protein that functions as an upstream regulator. FDX1 contributes to copper toxicity through two coordinated activities—reduction of Cu^2+^ to the more reactive Cu^+^ state and support of lipoyl synthase (LIAS) in lipoic acid biosynthesis [17, 222, 223]. Copper reduction is essential, as Cu^+^ exhibits the necessary affinity for lipoylated substrates. Accordingly, FDX1 expression correlates with cuproptosis sensitivity, and loss of FDX1 confers resistance, whereas its presence enables copper–protein engagement [224]. Notably, the progression of cuproptosis reveals a regulatory paradox. Although FDX1 is required for initiation, the terminal phase involves destabilization of Fe–S cluster proteins, resulting in depletion of FDX1 and LIAS themselves [221, 225].

FDX1 acts upstream of mitochondrial protein lipoylation through the LIAS pathway, thereby enabling lipoylation of mitochondrial dehydrogenase‐complex subunits such as DLAT and dihydrolipoamide *S*‐succinyltransferase (DLST), which serve as major copper‐binding effector proteins during cuproptosis [17]. Following FDX1‐dependent copper reduction, Cu^+^ binds directly to the lipoyl groups of these enzymes. This interaction promotes aberrant oligomerization and the formation of insoluble aggregates. Such aggregation represents a defining molecular hallmark of cuproptosis and has been observed in multiple tumor models, including osteosarcoma and triple‐negative breast cancer (TNBC) [226, 227]. Accumulation of these aggregates elicits acute proteotoxic stress, reflected by the induction of heat shock proteins, including HSP70, and activation of ER stress markers such as CHOP and EIF2A [228, 229].

Subsequent protein aggregation drives profound mitochondrial dysfunction. Biochemically, cuproptosis is associated with widespread destabilization of Fe–S cluster‐containing proteins—including FDX1, LIAS, ACO2, and SDHB—consistent with copper‐mediated disruption of Fe–S cluster assembly [230, 231, 232]. Morphologically, mitochondria exhibit features distinct from classical necrosis or apoptosis, including membrane rupture, vacuolization, marked shrinkage, and loss of cristae architecture [233, 234, 235]. Functionally, these alterations result in rapid TCA cycle arrest, ATP depletion, and dissipation of the mitochondrial membrane potential (ΔΨ_m_) [236, 237]. In certain contexts, mitochondrial membrane rupture permits release of mitochondrial DNA (mtDNA) into the cytosol, activating the cGAS–STING pathway and linking metabolic failure to innate immune signaling [220, 238].

### Metabolic gating and resistance mechanisms

In contrast to apoptosis or ferroptosis, which can proceed under varied metabolic states provided key effectors are engaged, cuproptosis is tightly constrained by cellular metabolism. Its execution, characterized by copper‐dependent oligomerization of lipoylated proteins, requires a specific convergence: intracellular copper accumulation coincident with a high abundance of lipoylated enzymes within the mitochondrial matrix [17, 239]. The intricate metabolic regulatory networks and resistance mechanisms that govern this threshold are detailed in Figure 5B. Susceptibility is therefore determined by TCA cycle activity and the extent of mitochondrial respiration. Tumor cells often exploit metabolic plasticity to evade this vulnerability, shifting from oxidative phosphorylation (OXPHOS) to aerobic glycolysis, consistent with the Warburg phenotype, to reduce reliance on mitochondrial metabolism and limit copper toxicity [17, 240]. A central determinant of this metabolic gating is pyruvate flux into mitochondria, which dictates the lipoylation status of the PDC [241]. Cells with high glycolytic dependence restrict mitochondrial pyruvate entry, thereby decreasing the pool of lipoylated substrates available for copper binding. In defined oncologic settings, including TNBC and lung adenocarcinoma, this resistance is reinforced through transcriptional reprogramming. XBP1s‐driven super‐enhancers promote MGRN1 expression to facilitate the ubiquitin‐proteasome‐mediated degradation of LIPT1, subsequently driving glycolysis reprogramming and effectively dismantling the lipoylation machinery required for cuproptosis [242]. Similarly, in HCC, a cuproptosis‐resistant phenotype is associated with enhanced glycolytic/hypoxic programs and reduced expression of pro‐cuproptosis genes, including FDX1 and dihydrolipoamide dehydrogenase (DLD) [243, 244, 245].

Conversely, genetic contexts that restrict glycolytic adaptation enhance sensitivity. ARID1A deficiency impairs HIF‐1α binding to the *PKM* promoter, limiting glycolytic enzyme induction and enforcing mitochondrial respiration, thereby increasing vulnerability to copper ionophores [246]. In parallel, p53 activation induces *circFRMD4A*, which binds PKM2 to suppress glycolysis and redirect carbon flux toward mitochondrial oxidation, thereby establishing a metabolic state that is permissive for cuproptosis [215]. Recent perspectives precisely dissect how p53 and noncoding RNAs orchestrate these cuproptotic susceptibilities [247, 248]. Strategies that reverse glycolytic dominance can sensitize resistant tumors. Pharmacologic inhibition of glycolysis with 2‐deoxy‐D‐glucose (2‐DG) or Glut1 inhibitors reduces ATP production and drives compensatory mitochondrial respiration, thereby enhancing copper toxicity [225, 238]. More direct metabolic manipulation, such as substitution of glucose with galactose in culture, forces reliance on the TCA cycle and increases DLAT lipoylation, resulting in heightened sensitivity to elesclomol (CupriActivitor1) [249, 250]. Induction of glucose deprivation using glucose oxidase (GOx) promotes mitochondrial copper transporters, including Slc25a3, and enhances DLAT oligomerization. These findings illustrate that metabolic stress can be exploited to lower the threshold for cuproptosis [251, 252, 253].

Beyond intrinsic metabolic programming, the hypoxic TME acts as an environmental checkpoint that suppresses cuproptosis. Hypoxia attenuates mitochondrial respiration and downregulates the FDX1–lipoylation axis, thereby generating a protective niche. This adaptation is mediated by HIF‐1α, which induces pyruvate dehydrogenase kinases 1 and 3 (PDK1/3) [17]. These kinases phosphorylate DLAT at Ser^100^, promoting ubiquitination and degradation and reducing the abundance of copper‐binding targets [28, 254]. Hypoxia also suppresses FDX1 transcription [255]. In radioresistant tumors, although stress may increase expression of the copper importer CTR1, resistance is maintained through BACH1 downregulation, which derepresses metallothioneins (MT1E/X) to sequester intracellular copper [221]. Accordingly, reoxygenation strategies, including catalase‐mimetic nanozymes, reduce HIF‐1α activity, restore mitochondrial respiration, and resensitize tumors to copper‐induced death [256, 257].

Regulation of FDX1 extends beyond transcription to post‐translational modulation. In TNBC, AKT1 phosphorylates FDX1 at Ser^63^, impairing its activity and reducing protein lipoylation, thereby conferring resistance [258]. Metabolic byproducts further influence this axis. Lactate promotes METTL16 lactylation in gastric cancer by stabilizing *FDX1* mRNA through *m* ^6^A modification and enhancing sensitivity [80]. In contrast, in ESCC, L‐lactate induces NUDT21 lactylation, promoting distal polyadenylation and destabilization of FDX1 mRNA, which diminishes cuproptosis susceptibility [212]. These context‐dependent effects underscore direct regulation of cell death machinery by metabolic intermediates [80]. Finally, cellular copper homeostasis is reinforced by active export and antioxidant buffering. ATP7A and ATP7B function as primary copper exporters. In glioblastoma stem cells, ATP7A expression is rhythmically controlled by the circadian regulator BMAL1, generating temporal windows of resistance [259]. Intracellular GSH limits copper reactivity by chelation, preventing engagement with lipoylated substrates [250]. Depletion of GSH, through agents such as buthionine sulfoximine (BSO) or cystine restriction, synergizes with copper ionophores to overcome this buffering capacity [222, 260, 261]. Additional protective layers include peroxiredoxin 1 (PRDX1) and induction of fatty acid metabolic enzymes such as FASN and SCD1, which preserve membrane integrity and mitigate mitochondrial dysfunction [259, 262, 263]. Thus, cuproptosis reflects a conditionally accessible lethality regulated by metabolic flux, hypoxic signaling, and antioxidant defenses rather than a simple consequence of copper accumulation.

### Crosstalk between ferroptosis and heme metabolism

Although cuproptosis is mechanistically defined by aggregation of lipoylated TCA cycle proteins, accumulating evidence indicates substantial crosstalk with ferroptosis and heme and iron metabolism [264, 265]. This intersection is mechanistic rather than additive and reflects shared dependencies in antioxidant buffering, mitochondrial bioenergetics, and metal‐catalyzed oxidative chemistry. A central node of convergence is the intracellular GSH pool, which performs dual protective functions: it serves as the obligate cofactor for GPX4 to suppress ferroptosis and acts as a physiological chelator of Cu^+^ to limit cuproptosis. Intracellular GSH acts as a specific trigger for copper‐based chemodynamic therapy, where the association of copper nanoparticles with GSH induces rapid GSH depletion and activates the catalytic conversion of H_2_O_2_ to cytotoxic hydroxyl radicals [266]. Foundational data show that copper (100 μM) oxidizes GSH to GSSG more extensively than iron, lowering the GSH:GSSG ratio [193]. In therapeutic contexts, copper‐based compounds can reduce intracellular GSH by up to 93% through redox cycling, thereby removing a shared constraint on both pathways. Loss of GSH liberates labile copper for interaction with lipoylated proteins and disables GPX4‐dependent detoxification [232, 234, 267].

**Figure 6:**
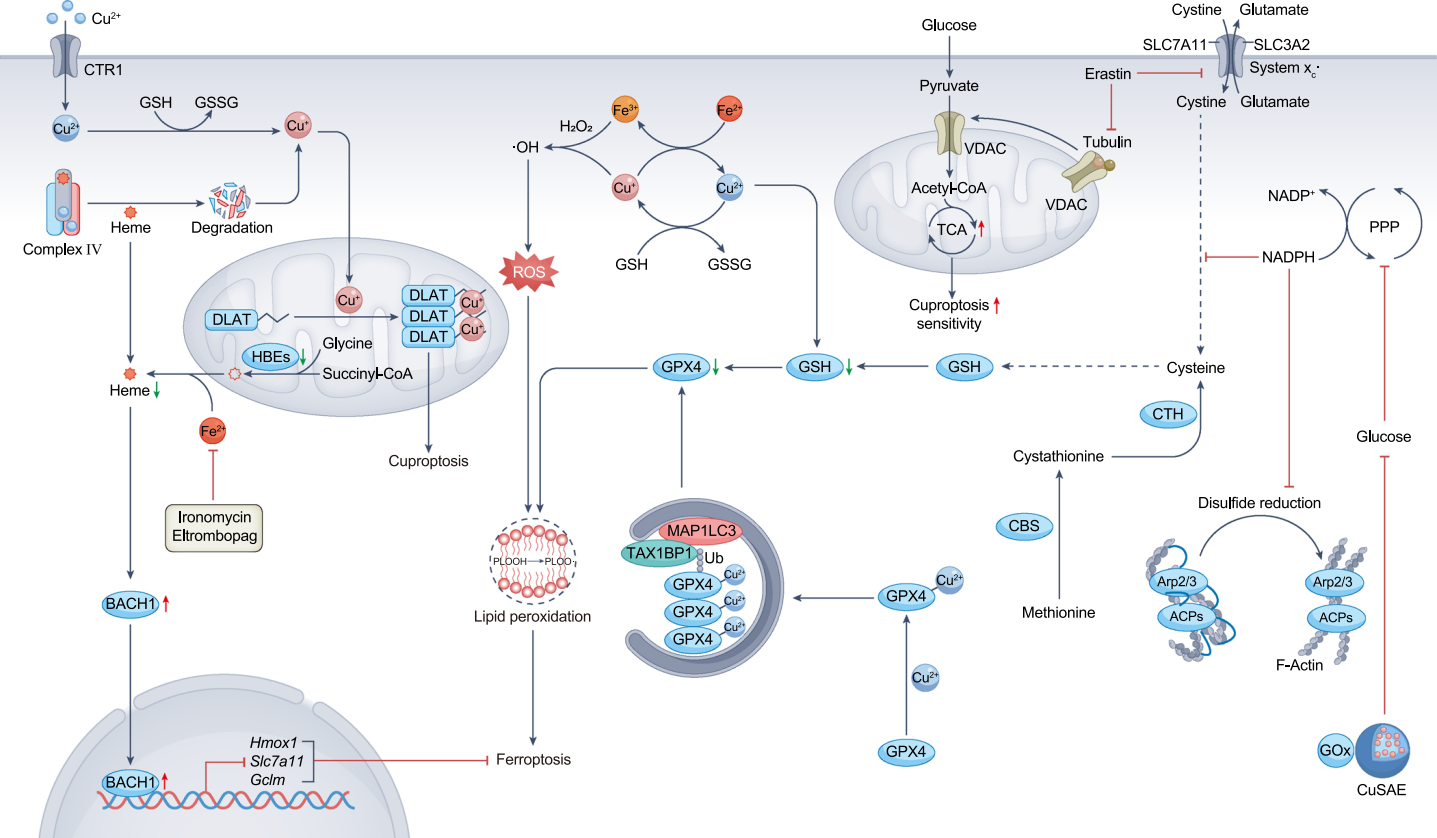
Mechanistic crosstalk among cuproptosis, ferroptosis, and metabolic regulation. Following cellular uptake primarily via CTR1, intracellular Cu^2+^ rapidly oxidizes glutathione (GSH) to GSSG, depleting the cytosolic GSH pool. The resultant labile Cu^+^ synergizes with iron to accelerate Fenton‐like reactions, generating reactive oxygen species (ROS) that promote lipid peroxidation [271]. Concurrently, Cu^2+^ directly binds GPX4, triggering its TAX1BP1‐mediated autophagic degradation to advance ferroptosis [187]. Within the mitochondria, impaired heme biosynthesis disrupts Complex IV assembly, redirecting unbound copper to bind lipoylated DLAT, thereby inducing proteotoxic oligomerization and cuproptosis. Concomitantly, heme deficiency stabilizes the transcription factor BACH1, promoting its nuclear translocation to repress antioxidant genes, including *Hmox1*, *Slc7a11*, and *Gclm* [216]. Furthermore, cellular metabolic states dynamically regulate these mechanisms. Erastin inhibits system x_c_^−^ and antagonizes tubulin‐VDAC interactions, shifting the metabolism toward the tricarboxylic acid (TCA) cycle to increase cuproptosis sensitivity [272]. Additionally, concurrent glucose starvation and NADPH depletion induced by GOx and copper nanozymes (CuSAE) impair physiological disulfide reduction. This impairment provokes aberrant disulfide bonding within F‐actin and Arp2/3 complexes, leading to cytoskeletal collapse and disulfidptosis [39, 273].

Copper also imposes direct proteotoxic stress on ferroptosis regulators, resulting in a convergent death phenotype. Copper binds the cysteine residues C107 and C148 of GPX4, promoting aggregation and subsequent TAX1BP1‐mediated autophagic degradation [187] (Figure 6). This copper‐driven loss of GPX4 induces ferroptosis independent of transcriptional changes, showing how proteotoxic mechanisms can precipitate lipid peroxidation [225]. Simultaneously, Cu^+^ generated during GSH depletion catalyzes decomposition of H_2_O_2_ into hydroxyl radicals through Fenton‐like reactions. Kinetic analyses indicate that copper‐mediated reactions can proceed up to 160‐fold faster than iron‐mediated Fenton chemistry in acidic microenvironments, producing an oxidative burst that overwhelms compromised antioxidant systems and drives lipid peroxidation and mitochondrial protein damage [268, 269, 270].

Crosstalk further extends into mitochondrial iron cofactors, linking copper toxicity to heme and Fe–S cluster integrity. Inhibition of heme biosynthesis has been reported to trigger cuproptosis. As heme is required for assembly of mitochondrial complex IV (cytochrome c oxidase), which incorporates copper, heme deficiency disrupts complex IV maturation and prevents the proper sequestration of copper into the enzyme complex, potentially leading to an accumulation of unassembled/unincorporated mitochondrial copper [274]. This excess copper can bind DLAT, promoting oligomerization and cell death, thereby establishing an inverse relationship between heme availability and copper sensitivity [216]. A defining feature of the cuproptosis is the destabilization of Fe–S cluster proteins, including FDX1 and LIAS [17]. As LIAS requires an Fe–S cluster for catalytic lipoylation, copper‐induced cluster disruption directly impairs iron‐dependent TCA cycle machinery [275, 276]. Copper overload can also engage iron metabolism to amplify lethality; activation of NCOA4‐dependent ferritinophagy releases substantial amounts of labile Fe^2+^, which can potentiate lipid peroxidation in a ferroptosis‐like manner [276, 277].

The execution of these interconnected programs remains metabolically gated. Cuproptosis requires active mitochondrial respiration, and interventions that shift metabolism from glycolysis toward the TCA cycle, including anti‐Warburg strategies or glucose deprivation, enhance susceptibility [252, 272]. Under conditions of glucose limitation combined with high SLC7A11 expression, copper agents can additionally provoke disulfidptosis together with ferroptosis, driven by NADPH depletion and cytoskeletal instability [273, 278]. Therapeutic approaches have begun to exploit this overlap [279]. Co‐delivery systems incorporating iron and copper generate a self‐reinforcing redox cycle in which the copper accelerates reduction of Fe^3+^ to Fe^2+^, amplifying oxidative stress beyond that achieved by either metal alone [271, 280, 281]. Similarly, the combination of copper ionophores with ferroptosis inducers overcomes metabolic resistance to cuproptosis, demonstrating that these pathways can be integrated to disrupt tumor survival networks [47, 282, 283]. Recent consensus guidelines further characterize intersections between metal‐dependent cell death and autophagy‐regulated degradation processes [6].

### Section summary

In summary, the conceptual evolution of cuproptosis has extended the definition of metal‐induced RCD from general toxicity to a targeted process regulated by metabolic states. Unlike lipid peroxidation‐driven mechanisms of ferroptosis [14], cuproptosis depends on the direct interaction of metal ions with specific metabolic pathways. Mechanistically, FDX1‐mediated copper reduction facilitates the selective binding and subsequent abnormal aggregation of lipoylated TCA cycle enzymes [17]. This process transforms normal mitochondrial OXPHOS into a source of severe proteotoxic stress. However, due to metabolic adaptations of the TME, clinical translation is limited. As cuproptosis requires active mitochondrial respiration, the prevalent aerobic glycolysis (Warburg effect) and hypoxic conditions within solid tumors inherently suppress OXPHOS [28, 242]. This metabolic shift reduces the availability of lipoylated substrates, conferring cellular resistance to copper‐induced cytotoxicity. Overcoming this limitation requires strategies targeting metabolic modulation and pathway integration, such as glycolysis inhibitors or hypoxia‐alleviating nanomaterials, which can redirect carbon flux toward OXPHOS, restoring cellular sensitivity to cuproptosis [236, 284]. Furthermore, leveraging the mechanistic overlap between cuproptosis and ferroptosis via synergistic bimetallic (copper and iron) induction accelerates GSH depletion, systematically compromising the antioxidant defenses of tumor cells [47, 280].

## EMERGING METAL‐DRIVEN LETHAL MODALITIES

### Overview

Physiologically, calcium, sodium, zinc, manganese, and cobalt are essential cellular regulators; however, exceeding strict intracellular thresholds transforms these metals into potent inducers of specific regulated cell death programs [4, 285]. Despite this shared terminal disruption, each pathway executes cellular demise through distinct molecular architectures (Table 1). Sustained Ca^2+^ overload can promote calcicoptosis by driving MCU‐dependent mitochondrial Ca^2+^ accumulation, which favors Ca^2+^‐induced conformational switching of F‐ATP synthase and mitochondrial permeability transition pore (mPTP) opening [38, 286, 287]. Massive sodium influx drives necrosis by sodium overload (NECSO) through lethal osmolar collapse and reverse NCX activation. Similarly, zinc toxicity induces zincoptosis‐like cell death by perturbing the lysosomal–mitochondrial signaling axis [60] and repressing the FDX2/LIAS pathway, thereby severely impairing Fe–S cluster biogenesis [24]. Moreover, excessive manganese promotes mnoptosis through enzymatic mismetallation, specifically replacing the native iron cofactor in coenzyme Q_7_ (CoQ_7_) and consequently arresting coenzyme Q (CoQ) biosynthesis [75]. Finally, toxic cobalt accumulation executes coptosis through pseudohypoxia and m^6^A hypermethylation‐driven epitranscriptomic toxicity. Crucially, these discrete cell death modalities rarely operate as isolated biological events; instead, they integrate through intricate in vivo crosstalk to orchestrate interconnected sequential cascades or simultaneous execution networks, a phenomenon broadly conceptualized as PANoptosis. By systematically tracing these pathways from physiological function toward overt cytotoxicity, progressing sequentially from calcium to zinc to manganese, and ultimately deciphering their overlapping lethal signaling architectures, this chapter delineates structural and metabolic vulnerabilities that directly inform emerging therapeutic translational strategies and precision metallometabolic interventions designed to circumvent single‐pathway resistance.

### Calcicoptosis: mPTP activation‐driven mitochondrial permeabilization

**Figure 7:**
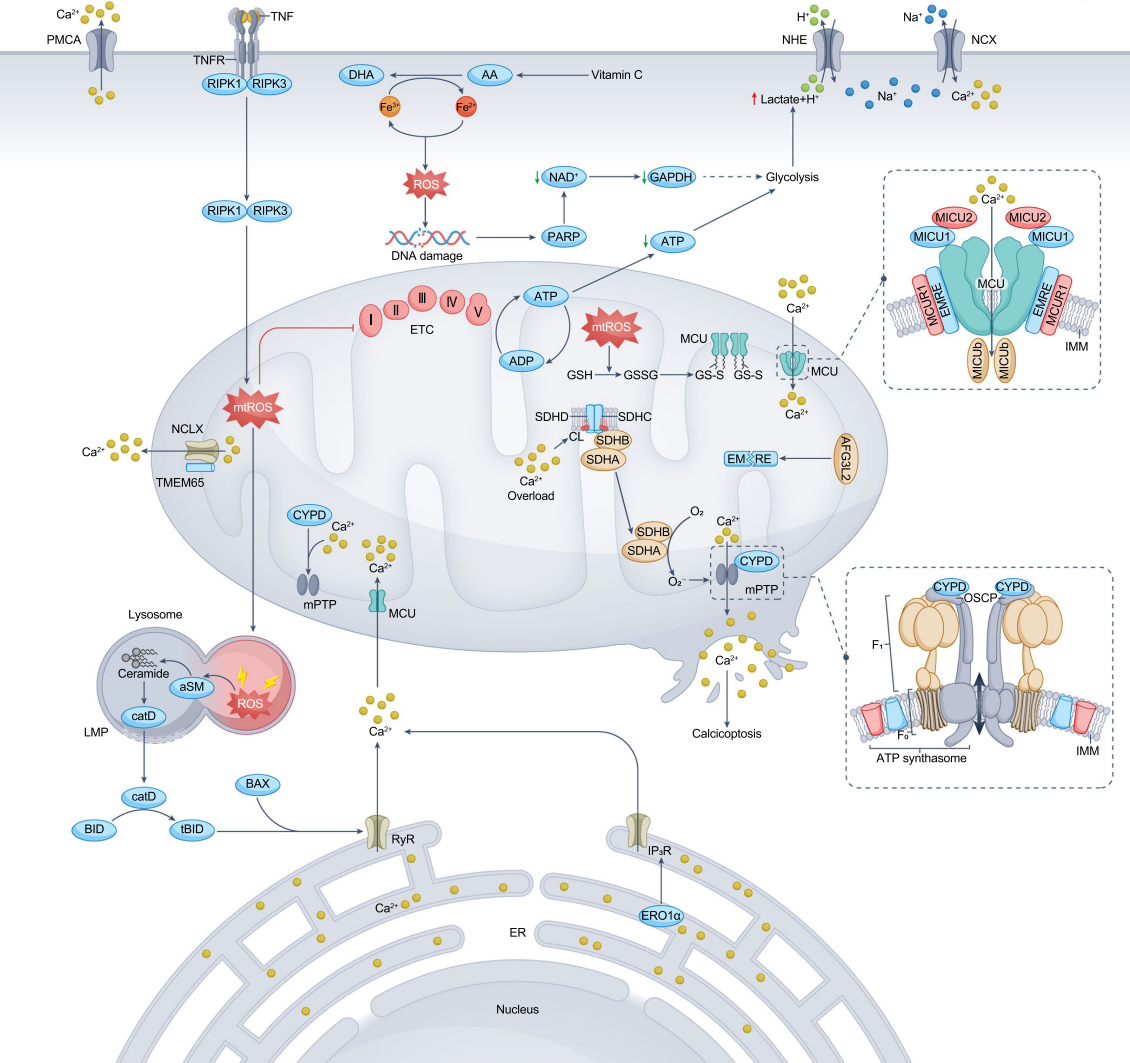
Molecular architecture of calcicoptosis driven by calcium dysregulation. Pathogenic stimuli drive interorganellar calcium (Ca^2+^) dysregulation. Tumor necrosis factor (TNF) signaling activates the RIPK1/RIPK3 complex, generating mitochondrial reactive oxygen species (mtROS), which, in turn, stimulates lysosomal acid sphingomyelinase (aSM) to produce ceramide, inducing lysosomal membrane permeabilization. Released cathepsin D (catD) cleaves BID into tBID, activating ER‐localized Bax to open ryanodine receptors (RyR) [289]. Concurrently, iron‐dependent vitamin C oxidation generates cytosolic ROS, activating ERO1α to sensitize inositol 1,4,5‐trisphosphate receptors (IP_3_R) [156]. Mobilized Ca^2+^ enters the mitochondrial matrix via the mitochondrial calcium uniporter (MCU) complex, gated by MICU1/MICU2/MICUb and scaffolded by MCUR1 and AFG3L2‐regulated EMRE [50, 290]. Oxidative stress exacerbates Ca^2+^ influx through MCU S‐glutathionylation [291]. Matrix Ca^2+^ overload ensues when sustained influx exceeds the efflux capacities of TMEM65‐stabilized NCLX and plasma membrane Ca^2+^ ATPase (PMCA) [81]. Elevated Ca^2+^ disrupts cardiolipin (CL), detaching the SDHA/SDHB subcomplex from respiratory complex II to generate superoxide [292]. Synergistically, ROS‐induced DNA damage activates PARP, depleting NAD^+^ and impairing GAPDH‐mediated glycolysis, thereby depleting ATP [51]. Ultimately, Ca^2+^ overload and oxidative stress trigger the conformational transition of F‐ATP synthase dimers into the mitochondrial permeability transition pore (mPTP), which is sensitized by cyclophilin D (CypD) binding to the OSCP subunit, thereby mediating calcicoptosis [19, 293].

Although calcium is widely recognized as a universal second messenger that regulates cell survival and adaptation, its dysregulation defines a regulated lethal program termed calcicoptosis. In contrast to accidental necrosis arising from acute mechanical injury or nonspecific membrane rupture, calcicoptosis represents a programmed response driven by sustained mitochondrial matrix calcium overload [38]. Excessive accumulation promotes opening of the mPTP, leading to rapid dissipation of ΔΨ_m_, collapse of OXPHOS, and remodeling of the inner membrane architecture [12, 288]. Distinct from caspase‐dependent apoptosis and lipid peroxidation‐mediated ferroptosis, calcicoptosis is strictly defined as a calcium‐dependent, mPTP megachannel opening‐driven RCD (Table 1). It is characterized by a defined signaling hierarchy in which the MCU complex functions as the upstream gatekeeper and the F‐ATP synthase conformational transition serves as the terminal pore‐forming effector, driving inner mitochondrial membrane permeabilization. The integrated molecular architecture of calcicoptosis is schematically illustrated in Figure 7.

The molecular identity of the mPTP remained debated for decades, but biochemical and electrophysiological evidence have challenged the role of the adenine nucleotide translocator, demonstrating instead that dimers of the F‐ATP synthase (F_1_F_0_‐ATP synthase) itself form the pore [19]. Under conditions of calcium excess and oxidative stress, F‐ATP synthase undergoes a conformational transition from an ATP‐producing enzyme to a high‐conductance pore that dissipates membrane potential [287, 293]. This transformation requires dimerization; reconstitution experiments demonstrate that only F‐ATP synthase dimers, not monomers, generate Ca^2+^‐dependent currents consistent with the approximately 1.3 nS conductance of the native megachannel [293]. Mechanistically, pore opening involves the disruption of the interactions between the e‐subunit and the c‐ring following calcium binding, permitting the formation of a leak pathway within the c‐subunit ring [294]. Sensitivity of this transition is modulated by cyclophilin D (CypD), which interacts with the OSCP subunit to lower the calcium threshold for pore activation [19, 295], a process inhibited by cyclosporin A [73].

Matrix calcium accumulation beyond a defined threshold is required for calcicoptosis and is governed by the MCU complex. As a highly selective channel lacking intrinsic calcium‐dependent inactivation, MCU depends on accessory subunits to prevent excessive influx [31, 296]. MICU1 and MICU2 provide cooperative gating; loss of MICU1 abolishes sigmoid activation kinetics, resulting in constitutive calcium entry even at low cytosolic concentrations and heightened oxidative stress [10, 297, 298]. Structural stability of the channel requires the scaffold MCUR1 and the essential subunit EMRE, whose assembly is regulated by the mitochondrial m‐AAA protease AFG3L2 [299, 300]. Deficiency of AFG3L2 promotes accumulation of partially assembled MCU–EMRE complexes that bypass MICU regulation, driving chronic calcium overload and neurodegeneration [50, 290]. Our team recently deciphered the critical pathophysiological and therapeutic potential of the MCU complex [301]. In addition, MCU acts as a redox‐responsive channel; oxidative conditions induce *S*‐glutathionylation at cysteine‐97, promoting channel clustering and sustained activation [291].

Cellular susceptibility further depends on the balance between MCU‐mediated influx and calcium extrusion mediated by the mitochondrial Na^+^/Ca^2+^ exchanger (NCLX). NCLX represents the primary route for calcium efflux, and its regulation is essential for maintaining homeostasis [302]. TMEM65 has been identified as a critical partner of NCLX; loss of TMEM65 abrogates efflux capacity, whereas overexpression increases resistance to calcium‐induced death [81]. When efflux is compromised, physiological signaling can convert to pathological overload. In brown adipose tissue, adrenergic stimulation normally promotes thermogenesis, yet deletion of NCLX under identical stimulation leads to excessive calcium accumulation, mPTP opening, and necrotic cell death [85]. These observations underscore that calcicoptosis often reflects a kinetic imbalance in which sustained influx exceeds extrusion capacity [299].

Ca^2+^‐overload‐driven calcicoptosis engages ER–mitochondrial crosstalk, in which pathological stimuli trigger ER Ca^2+^ release through IP_3_, followed by Ca^2+^ transfer to mitochondria at mitochondria‐associated membranes (MAMs) [303, 304]. Furthermore, BAX can activate ER ryanodine receptors to precipitate lethal mitochondrial calcium overload [289]. Within the matrix, elevated calcium binds cardiolipin, destabilizing respiratory complex II and releasing the SDHA/SDHB subcomplex. This dissociation generates a localized ROS burst required to maintain mPTP opening [292]. Following Bax/BAK‐mediated mitochondrial outer membrane permeabilization (MOMP) in apoptosis, subsequent caspase activation leads to mitochondrial structural disruption and loss of membrane potential, which can occur independently of, or be followed by, mitochondrial swelling [8, 305]. Mitochondrial inner membrane permeabilization (MIMP) enables release of matrix constituents, such as mtDNA and TFAM, and is frequently accompanied by OMA1‐dependent cleavage of OPA1, inhibiting fusion and reinforcing irreversible fragmentation [77, 306]. The specificity of this machinery provides therapeutic opportunities: inhibition of MCU or mPTP confers protection in ischemic injury [307], whereas suppression of efflux pathways, including NCLX or PMCA, may be exploited to induce lethal calcium overload in malignant cells [308, 309, 310].

### NECSO: Osmolar collapse‐driven plasma membrane rupture

Although sodium is canonically recognized as the primary extracellular osmolyte governing membrane potential, its unconstrained intracellular accumulation beyond stringent physiological thresholds precipitates a specific regulated lethal program, canonically recognized as necrosis by sodium overload (NECSO) [74, 78]. Unlike passive osmotic swelling, NECSO represents an orchestrated homeostatic failure initiated by the persistent hyperactivation of specific sodium conduits [78, 311]. Central to this lethal architecture is the transient receptor potential melastatin 4 (TRPM4), a calcium‐activated non‐selective cation channel whose pathogenic stability—often governed by the SEC. 62 complex—mediates catastrophic cytosolic sodium influx [82, 312]. Concurrently, severe intracellular acidosis and metabolic stress aggressively hyperactivate the Na^+^/H^+^ exchanger 1 (NHE1) [100, 313], while oxidative stress aggravates Na^+^ overload by impairing Nav1.5 inactivation and ROS–CaMKII signaling, thereby enhancing late/persistent Na^+^ current [79, 314]. Once these influx routes bypass cellular buffering capacity, physiological ionic signaling irreversibly transitions into a lethal trajectory.

The ensuing massive sodium influx rapidly depolarizes the plasma membrane and triggers a pleiotropic downstream signaling cascade anchored in bioenergetic collapse and ionic interdependency. In a futile attempt to re‐establish the collapsed transmembrane gradient, the Na^+^/K^+^‐ATPase pump operates in extreme overdrive, relentlessly hydrolyzing ATP. This monopolization of local energy reserves precipitates a fatal bioenergetic sink that metabolically starves parallel cellular processes [93, 315]. Furthermore, stress‐induced calpain activation promotes proteolysis of the ankyrin–fodrin scaffold, detaching Na^+^/K^+^‐ATPase from the membrane cytoskeleton and thereby impairing Na^+^ extrusion [316]. Most crucially, the unchecked cytosolic sodium surge fundamentally alters the thermodynamic driving force of the bidirectional Na^+^/Ca^2+^ exchanger (NCX) [317, 318]. Forced into reverse‐mode operation, the NCX aggressively extrudes sodium at the expense of importing massive quantities of extracellular calcium. This pivotal mechanistic inversion transforms the primary sodium burden into a catastrophic secondary calcium overload, which inextricably bridges NECSO with classical calcicoptosis and orchestrates widespread intracellular organelle dysfunction [319, 320].

Execution of NECSO lacks a solitary terminal effector, instead manifesting as a multidimensional phenotypic crossroad dictated by the cellular microenvironment and stress severity. The canonical execution pathway is driven by sheer osmolar collapse—conceptually unified as NECSO [74, 321]. Uncompensated intracellular hyperosmolarity obligates a massive water influx via aquaporins, inducing profound cellular edema, extreme organelle swelling, and ultimate necrotic plasma membrane rupture [78, 89]. Alternatively, when the reverse NCX‐mediated secondary calcium overload synergizes with mitochondrial sodium accumulation, it acts as an independent upstream signal that physically ruptures the outer mitochondrial membrane or hyperactivates the mPTP, precipitating cytochrome *c* release and mitochondrial‐mediated apoptosis [90, 322]. Furthermore, profound ionic and osmotic shifts severely compromise lysosomal integrity. The subsequent lysosomal rupture and cytosolic leakage of cathepsin B (CTSB) directly catalyze the phosphorylation of NF‐κB, which rapidly assembles the NLRP3 inflammasome to execute GSDMD‐dependent pyroptosis [83, 97, 323].

The multifaceted molecular architecture of NECSO unveils a bidirectional translational frontier. In contexts of myocardial and cerebral IRI or anthracycline‐induced cardiotoxicity, mitigating sodium overload confers profound cytoprotection. Pharmacological interventions utilizing targeted TRPM4 blockers (e.g., clotrimazole), specific NHE inhibitors (e.g., cariporide), or late sodium current antagonists like ranolazine successfully dismantle the necrotic cascade and rescue mitochondrial energetics [79, 100, 324]. Conversely, clinical oncology ingeniously leverages this lethal machinery to eradicate malignancies, particularly in therapy‐resistant breast, colorectal, and hepatocellular carcinomas [325, 326]. Pharmacological or nanomaterial‐enabled Na^+^ overload can induce immunogenic tumor cell death: NC1 persistently activates human TRPM4 to trigger NECSO, while sodium‐delivering nanoplatforms such as ultrasound‐activated Sonophage and NFPP ion reservoirs intensify Na^+^ accumulation, osmotic stress, and inflammatory antitumor immunity [78, 311, 321]. Crucially, NECSO defines an orthogonal sodium‐overload death program that converges on mitochondrial bioenergetics and Na^+^/Ca^2+^ homeostasis, positioning it as a promising combinatorial node with ferroptosis, calcicoptosis, and cuproptosis to bypass single‐pathway resistance [78, 311].

### Zincoptosis: An emerging zinc‐dependent RCD

**Figure 8:**
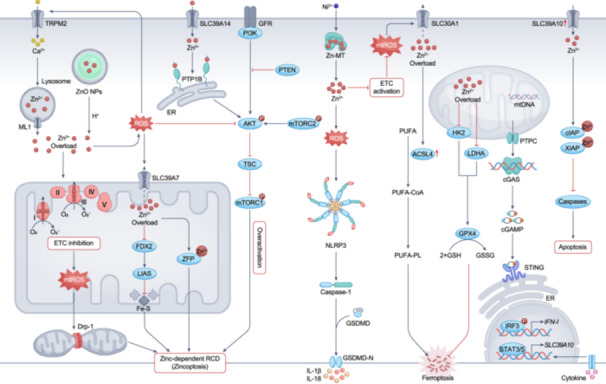
Molecular architecture of zinc‐mediated cytotoxicity and zincoptosis‐like cell death. At physiological concentrations, STAT3/5‐upregulated SLC39A10 (ZIP10) mediates zinc influx to stabilize cIAP and XIAP, suppressing caspase‐dependent apoptosis [330, 332]. Concurrently, SLC39A14‐transported zinc inhibits endoplasmic reticulum (ER)‐localized PTP1B to sustain pro‐survival Akt signaling [329]. Conversely, zinc overload triggers diverse regulated cell death pathways. Following reactive oxygen species (ROS)‐mediated TRPM2 activation and calcium influx, or the lysosomal degradation of acid‐responsive zinc oxide nanoparticles, TRPML1 facilitates lysosomal zinc release [22, 91, 98]. This zinc translocates into mitochondria, where it accumulates due to the downregulation of the efflux transporter SLC39A7 [87]. This inhibits the electron transport chain (ETC) and recruits Drp‐1 to drive fission [91, 333]. Within the matrix, zinc overload triggers zincoptosis by suppressing the FDX2–LIAS axis and disrupting Fe–S clusters and zinc finger proteins. This metabolic failure correlates with Akt/mTOR inhibition, correcting the depicted mTORC1 overactivation [87]. Furthermore, nickel competitively displaces zinc from metallothioneins; the liberated zinc inhibits ETC activity, contrary to the illustrated ETC activation, generating ROS to activate the NLRP3/GSDMD pyroptotic cascade [23, 334]. Simultaneously, zinc‐mediated suppression of HK2 and LDHA depletes glutathione to impair GPX4 [335], which, alongside ACSL4 upregulation following the loss of the SLC30A1 efflux transporter, precipitates ferroptosis [87]. Finally, mitochondrial permeabilization releases mtDNA, engaging the cGAS–STING–IRF3 axis to elicit type I interferon (IFN‐I) secretion [336].

Although zinc has traditionally been regarded as a cytoprotective antioxidant and an essential structural cofactor for more than 3,000 proteins, emerging evidence supports its role as a bidirectional signal constrained by strict homeostatic control, orchestrated by zinc transporters such as SLC39A4 (ZIP4) and SLC39A5 (ZIP5) [327, 328]. At physiological concentrations, intracellular zinc exerts antiapoptotic effects by stabilizing inhibitor of apoptosis proteins (IAPs) and inhibiting phosphatases such as PTP1B, thereby sustaining pro‐survival signaling [329, 330, 331]. For instance, SLC39A10 (ZIP10) facilitates crucial antiapoptotic signaling during early B‐cell development [332]. In contrast, disruption of zinc balance follows a U‐shaped lethality curve: deficiency promotes spontaneous caspase activation through IAP degradation, whereas excess zinc initiates distinct regulated death programs. This section outlines the transition from homeostatic zinc signaling to cytotoxic overload and introduces a distinct regulated cell death modality, herein first termed “zincoptosis,” which proceeds independently of canonical apoptosis [60]. Figure 8 maps this transition from physiological signaling to diverse RCD pathways.

Building upon the foundational discovery that lysosomal Zn^2+^ release triggers rapid, mitochondria‐mediated nonapoptotic cell death, a distinct zinc‐dependent RCD pathway driven by severe bioenergetic exhaustion has been formally defined as zincoptosis‐like cell death [60] (Table 1). Mechanistically delineating this zinc‐induced mitochondrial collapse, unlike cuproptosis, which directly targets lipoylated proteins within the mitochondrial matrix, is defined by the targeted suppression of the ferredoxin 2 (FDX2)–LIAS axis and consequent disruption of Fe–S cluster biogenesis. Elevated zinc reduces *FDX2* and *LIAS* expression or stability, leading to acute mitochondrial dysfunction and proteotoxic stress within respiratory chain complexes [24, 223]. This metabolic failure is associated with cell cycle arrest at G_1_/S and G_2_/M checkpoints, mediated in part by inhibition of Akt/mTOR signaling. Biochemically, zincoptosis‐like cell death may correlate with reduced expression of the efflux transporter SLC39A7 (ZIP7), enhancing mitochondrial zinc accumulation, and with selective impairment of zinc finger proteins localized to the mitochondrial matrix [24].

A principal effector mechanism involves the lysosomal–mitochondrial axis, in which lysosomes act as zinc reservoirs [337]. Oxidative stress or pharmacological activation of TRPML1 can trigger TRPML1‐dependent efflux of lysosomal Zn^2+^ into the cytosol [338]. ROS‐dependent activation of TRPM2 channels and secondary calcium influx facilitate this mobilization [91]. The liberated zinc accumulates within mitochondria and inhibits ETC activity, promoting further ROS generation in a feed‐forward cycle [98]. Sustained inhibition induces mitochondrial permeability transition (mPT), loss of ΔΨ_m_, and ATP depletion [333, 339]. In parallel, mitochondrial zinc enrichment promotes recruitment of the fission protein Drp‐1 to the outer membrane, driving fragmentation and reinforcing irreversible mitochondrial damage [60, 340, 341].

Zinc overload also intersects with other regulated death pathways. Elevated intracellular zinc activates the NLRP3 inflammasome, leading to cleavage of caspase‐1 and gasdermin D (GSDMD), pyroptotic membrane permeabilization, and IL‐1β secretion [23, 336, 342, 343]. Competitive ionic displacement can intensify this response; for example, nickel can displace zinc from metallothioneins, increasing the free zinc pool and exceeding lethal thresholds [334]. Zinc dysregulation further promotes ferroptotic signaling. Zinc oxide nanoparticles stimulate NCOA4‐dependent ferritinophagy, releasing labile Fe^2+^ and enhancing lipid peroxidation [344]. Concurrently, zinc overload inhibits glycolytic enzymes, including HK2 and LDHA, reducing NADPH availability, lowering GSH levels, and suppressing GPX4 activity [335, 345]. Loss of the zinc exporter SLC30A1 (*ZnT1*) augments susceptibility by increasing ACSL4 expression, contributing to mixed ferroptotic and necroptotic phenotypes [87].

Finally, zinc‐driven cell death exhibits intrinsic immunogenic features. Mitochondrial injury caused by zinc accumulation facilitates cytosolic release of mtDNA, which functions as a damage‐associated molecular pattern and activates the cGAS–STING pathway, eliciting a type I interferon (IFN‐I) response [269, 346]. Zinc exposure also enhances cell surface presentation of calreticulin, providing a prophagocytic signal required for dendritic cell activation [347, 348]. Accordingly, therapeutic approaches employing zinc overload, including acid‐responsive nanocarriers, have demonstrated the capacity to convert immunosuppressed tumors into immunologically active microenvironments. These findings position zinc not solely as a cytotoxic agent but as a potential modulator of metalloimmunotherapy [256, 349].

### Mnoptosis: A putative manganese‐dependent RCD

**Figure 9:**
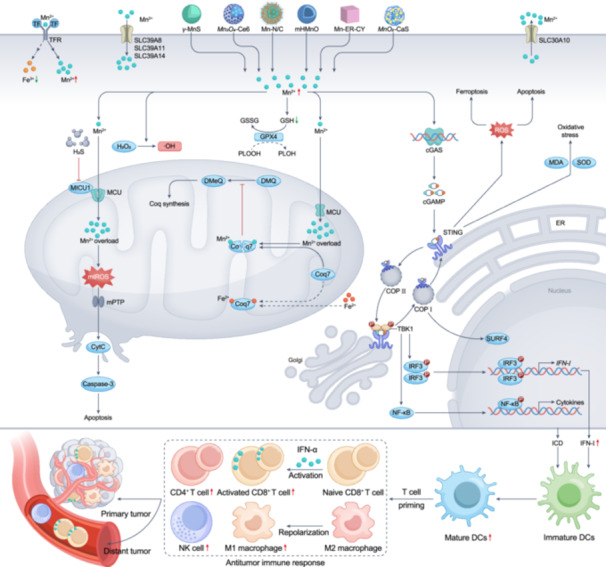
Mechanisms of manganese‐triggered cytotoxicity and systemic immunomodulation. Intracellular manganese (Mn^2+^) homeostasis is maintained by the influx transporter SLC39A8, SLC39A11, and SLC39A14 [352, 354, 356], together with the Mn^2+^ efflux transporter SLC30A10 [357]. Within the tumor microenvironment, disintegration of stimuli‐responsive nanoplatforms elevates cytosolic Mn^2+^ [359]. This elevated Mn^2+^ oxidizes glutathione (GSH) to glutathione disulfide (GSSG), inactivating GPX4, thereby inducing lipid peroxidation and ferroptosis, while concurrently catalyzing hydroxyl radical generation. Simultaneously, H_2_S‐mediated MICU1 downregulation permits mitochondrial Mn^2+^ influx via the MCU complex [360]. Within the mitochondrial matrix, Mn^2+^ competitively displaces the iron cofactor in CoQ_7_, arresting CoQ biosynthesis and leading to demethoxyubiquinone (DMQ) accumulation, thereby inducing mnoptosis‐like cell death [75]. Additionally, mitochondrial Mn^2+^ overload triggers mPTP opening, cytochrome c release, and caspase‐3‐dependent apoptosis [360, 361]. Furthermore, cytosolic Mn^2+^ activates cGAS, promoting cGAMP synthesis. STING translocates via COPII and COPI vesicles to recruit TBK1, leading to the phosphorylation of IRF3 and NF‐κB for IFN‐I and cytokine transcription [37, 59]. Secreted IFN‐I and ICD signals promote dendritic cell maturation, mediating systemic antitumor immunity through CD8^+^ and CD4^+^ T‐cell activation, natural killer cell recruitment, and M1 macrophage polarization [362, 363].

Manganese occupies a distinct position in metal biology, serving as an essential cofactor for multiple physiological enzymes while retaining the capacity to initiate regulated cytotoxic programs when homeostasis is disrupted [350]. In contrast to passive necrosis associated with nonspecific heavy metal accumulation, manganese‐dependent toxicity proceeds through defined molecular mechanisms, including mitochondrial enzymatic mismetallation and catalytic enhancement of oxidative stress. The shift from nutritional sufficiency to lethal overload is determined by tightly regulated transport systems [351]. SLC39A8 (ZIP8) is a physiologically relevant Mn^2+^ importer that preferentially transports Mn^2+^/Cd^2+^ over Zn^2+^, and endothelial SLC39A8 expression enhances cadmium accumulation and cadmium‐induced vascular injury [352, 353]. Furthermore, SLC39A14 and the longevity‐associated protein SLC39A11 have recently emerged as critical influx transporters essential for maintaining basal cellular manganese homeostasis [354, 355, 356]. Conversely, SLC30A10 mediates manganese efflux and serves as a primary defense against intracellular accumulation [351, 357, 358]. Structural analyses identify Asn43 in SLC30A10 as critical for manganese selectivity; mutation at this residue (e.g., N43H) or loss of SLC30A10 disrupts efflux and produces dose‐dependent cytotoxicity attributable specifically to manganese retention [87, 88]. These mechanisms of manganese‐induced cytotoxicity and immune activation are comprehensively illustrated in Figure 9.

When buffering capacity is exceeded, manganese toxicity is predominantly executed within mitochondria through a recently characterized regulated cell death pathway, herein termed first termed “mnoptosis.” As delineated in Table 1, this proposed modality is fundamentally defined as a manganese‐dependent, enzymatic mismetallation‐driven RCD. This pathway is characterized by the specific targeting of CoQ_7_ (demethoxy‐ubiquinone hydroxylase), a mitochondrial enzyme required for CoQ biosynthesis. Excess Mn^2+^ replaces the native iron cofactor in CoQ_7_, resulting in enzymatic inactivation and Pim1‐mediated proteolytic degradation. Impaired CoQ synthesis leads to accumulation of demethoxy‐CoQ (DMQ), respiratory chain dysfunction, and cell death. Rescue by supplementation with the CoQ precursor analog 2,4‐dihydroxybenzoic acid (diHB) supports the specificity of this metabolic lesion [75]. Mitochondrial access of manganese is regulated by the MCU complex. Under basal conditions, MICU1 limits ion entry through steric gating [364]; however, MICU1 deficiency or pathological activation permits sustained Mn^2+^ influx, promoting persistent mPTP opening and apoptotic signaling [86, 360].

In therapeutic contexts, manganese has been exploited as a catalyst in chemodynamic strategies that capitalize on the acidic and GSH‐enriched TME. High‐valence manganese oxides function as stimuli‐responsive reservoirs that degrade within the TME to release Mn^2+^, initiating a two‐phase cytotoxic cascade. First, reduction of Mn(IV/III) to Mn(II) consumes intracellular GSH, weakening antioxidant defenses [365, 366]. Second, liberated Mn^2+^ acts as a peroxidase‐like catalyst, converting endogenous H_2_O_2_ to hydroxyl radicals (·OH) through Fenton‐like chemistry [359]. This reaction is favored under acidic conditions typical of tumors and is attenuated at physiological pH, conferring relative selectivity [367, 368]. Beyond hydroxyl radical generation, manganese can facilitate the formation of carbon‐centered radicals from substrates such as dihydroartemisinin, resulting in DNA damage distinct from canonical ROS‐mediated injury [369].

Downstream outcomes of manganese signaling vary according to cellular context and redox state. GSH depletion and lipid peroxide accumulation frequently engage ferroptotic pathways, with manganese exposure associated with suppression of the GPX4–SLC7A11 axis and induction of ACSL4 expression [361, 370]. Alternatively, manganese‐induced oxidative stress can activate Oxeiptosis, a caspase‐independent pathway involving PGAM5 and AIFM1, or promote pyroptosis through NLRP3 inflammasome activation and gasdermin cleavage [371, 372]. Manganese also links metabolic injury to immune activation. Mn^2+^ acts as a physiological sensitizer of cGAS, lowering the activation threshold for cytosolic dsDNA [37]. This coupling of oxidative injury and innate immune signaling potentiates STING activation and IFN‐I production, positioning manganese not only as a cytotoxic mediator but also as an immunomodulatory factor [59, 362, 363, 373].

### Coptosis: A putative cobalt‐dependent RCD

Although cobalt is recognized as an essential trace element and structural cofactor for cobalamin (vitamin B_12_), its intracellular accumulation beyond physiological buffering capacities precipitates a specific transition metal‐dependent regulated cell death program, herein firstly termed “coptosis.” This conceptual paradigm is also partially supported by a recent mechanistic review [374]. Unlike accidental necrosis, this pathway is a highly orchestrated lethal response tied to homeostatic failure. Cellular cobalt influx is governed by promiscuous divalent metal transporters, specifically DMT1, alongside zinc transporters such as SLC39A8 and SLC39A14 [374, 375]. When cobalt homeostasis is overwhelmed, excess Co^2+^ can drive metalloprotein mismetallation by competing with iron in Fe–S cluster assembly and substituting for Zn in selected zinc‐binding motifs, thereby impairing Fe–S enzymes and zinc‐finger‐dependent functions [376, 377]. Pathologically, this unchecked accumulation underpins the severe localized and systemic toxicities observed in arthroprosthetic cobaltism syndrome, manifesting as severe neurodegeneration, cardiotoxicity, and macrophage‐driven inflammatory tissue necrosis secondary to the degradation of metal‐on‐metal orthopedic implants [378, 379, 380].

The molecular architecture of coptosis may be fundamentally anchored in the synergistic collapse of mitochondrial bioenergetics and epitranscriptomic stability. Upon entering the cytosol, cobalt preferentially accumulates within the mitochondrial matrix, inducing Drp1‐mediated mitochondrial fission, profound loss of ΔΨ_m_, and OXPHOS collapse, culminating in drastic ATP depletion [381, 382, 383]. Intriguingly, cobalt chloride acts as a chemical hypoxia mimetic, stabilizing HIF‐1α under normoxia partly by interfering with Fe^2+^‐dependent prolyl hydroxylase activity and subsequent VHL‐mediated degradation [384, 385]. This aberrant HIF‐1α signaling forces maladaptive metabolic reprogramming and upregulates pro‐death targets such as BNIP3 [386, 387]. Furthermore, cobalt exerts an unprecedented epigenetic toxicity. Cobalt‐driven ROS directly suppress the m^6^A RNA demethylases ALKBH5 and FTO [388, 389]. This epitranscriptomic imbalance drives the pathological m^6^A hypermethylation of critical mRNAs, including the cytoprotective antioxidant enzyme HO‐1, neutralizing cellular stress defenses and dictating an oxidative execution [388, 389].

Rather than relying on a solitary executioner, cobalt functions as a pleiotropic master switch that seamlessly orchestrates a multimodal cell death network. At basal thresholds, cobalt‐induced metabolic stress and ROS trigger classical intrinsic apoptosis, driven by Bax/Bcl‐2 ratio dysregulation, cytochrome c release, and caspase cascade activation [375, 390]. Concurrently, cobalt aggressively drives lipid peroxidation‐dependent ferroptosis by catalyzing Fenton‐like reactions, depleting GSH, and suppressing the GPX4–SLC7A11 axis. Paradoxically, cobalt also hyperactivates the noncanonical KEAP1/NRF2/HMOX1 pathway, precipitating massive intracellular Fe^2+^ overload [391, 392, 393]. Depending on the inflammatory context, cobalt interacts with innate immune sensors to activate extensive pyroptotic cascades via the NLRP3/caspase‐1/GSDMD axis, ROS‐dependent caspase‐8/GSDMC cleavage, or autophagy‐linked caspase‐3/GSDME activation [394, 395, 396, 397]. In highly aggressive tumor models, this pleiotropic capacity converges to trigger PANoptosis, wherein bimetallic nanozymes synchronize apoptosis, necroptosis, and pyroptosis through the NLRC5/NLRP3‐PANoptosome complex, unleashing an irreversible inflammatory lethal storm [398].

The multifaceted lethality of cobalt presents a unique dichotomy in translational medicine. In healthy tissues, unregulated Co^2+^ release dictates severe periprosthetic osteolysis and neurodegeneration, necessitating precise pharmacological interventions with phyto‐chelators, specific antioxidants, and ROS scavengers to rescue cellular viability [399, 400, 401]. Conversely, clinical oncology ingeniously repurposes this lethal architecture. Hypoxia‐activatable Co^3+^ prodrugs and ZIF‐67‐based cobalt MOF nanoplatforms have been engineered as TME‐responsive delivery systems, coupling Co^3+^‐to‐ Co^2+^ reductive activation with acid‐triggered drug release and, in redox‐active composites, GSH‐depleting tumor microenvironment remodeling [402, 403]. Upon targeted reduction to labile Co^2+^ species, these platforms dismantle tumor redox homeostasis and robustly provoke type II ICD, characterized by profound endoplasmic reticulum stress, calreticulin membrane translocation, and extracellular HMGB1 and ATP efflux [404]. Crucially, cobalt‐induced mitochondrial DNA release intrinsically hyperactivates the cGAS‐STING innate immune pathway. When synergized with immune checkpoint blockade, this promotes dendritic cell maturation and T cell infiltration, effectively reprogramming immunosuppressive tumors into immunologically active microenvironments for advanced metalloimmunotherapy [404, 405]. Crucially, as exemplified by the multifaceted downstream outcomes of cobalt alongside other aforementioned metal ions, these distinct cell death pathways do not operate as isolated biological events; rather, they involve extensive in vivo crosstalk to orchestrate a coordinated execution network.

### In vivo crosstalk and PANoptosis

Given the distinct molecular architectures of the ion‐specific RCDs delineated above, the concept of PANoptosis provides a critical framework for understanding the complex interrelationships among these diverse lethal programs [406, 407]. Accordingly, the rigid classification of cell death into discrete, nonoverlapping modalities, including apoptosis, necrosis, and autophagy, is increasingly viewed as an oversimplification of the biological complexity within physiological tissue microenvironments. Recent studies indicate that metal‐dependent RCD seldom occurs as an isolated event. Instead, dysregulation of metal ions initiates a continuum of lethal subroutines that interact through intricate crosstalk, sequential cascades, or simultaneous execution complexes, an integrated inflammatory module termed PANoptosis [407, 408]. Although PANoptosis was originally defined by the concurrent activation of pyroptosis, apoptosis, and necroptosis, metal‐dependent signaling has expanded this framework to encompass ferroptosis, cuproptosis, and additional metal‐dependent cell death modalities [409, 410]. Deciphering these interactions in vivo remains analytically challenging but represents a critical path toward overcoming therapeutic resistance and reducing off‐target toxicity.

**Figure 10:**
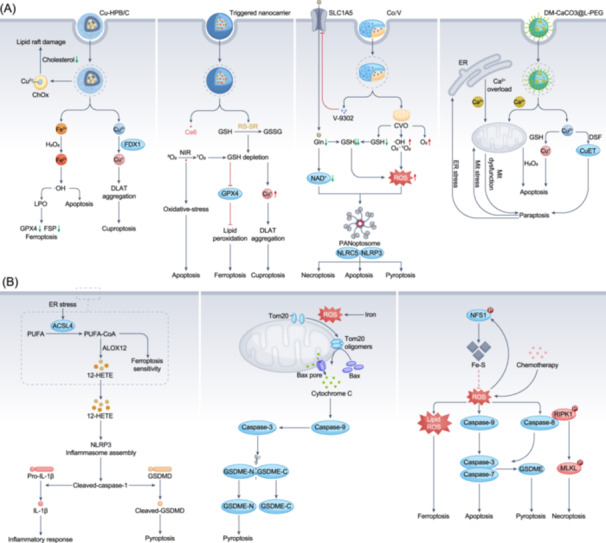
In vivo crosstalk and metal‐dependent PANoptosis. Metal dysregulation initiates interconnected lethal pathways, leading to concurrent execution or sequential cascades [4]. (A) Engineered nanoplatforms induce multimodal cell death. The Cu‐HPB/C system oxidizes cholesterol to disrupt lipid rafts, generating hydrogen peroxide and releasing copper and iron ions to drive apoptosis, ferroptosis, and cuproptosis [414]. Photo‐responsive nanocarriers deplete glutathione via singlet oxygen and thiol‐disulfide exchange, synchronously activating apoptosis, ferroptosis, and cuproptosis [225, 417]. Cobalt/vanadium (Co/V) platforms combine reactive oxygen species (ROS) generation with SLC1A5 blockade to deplete GSH/NAD^+^, assembling the NLRC5/NLRP3 PANoptosome to concurrently execute necroptosis, apoptosis, and pyroptosis [398]. DM‐CaCO_3_@L‐PEG releases Ca^2+^ and Cu^2+^ to trigger paraptosis, characterized by endoplasmic reticulum and mitochondrial vacuolization, alongside apoptosis [418]. (B) Endogenous metal signaling dictates sequential hierarchies. In heart failure, ACSL4‐driven lipid peroxidation generates 12‐HETE, initiating NLRP3‐mediated pyroptosis [29, 419]. In melanoma, iron‐amplified ROS oxidizes mitochondrial Tom20, thereby recruiting Bax. Further, activated caspase‐3 is atypically redirected to cleave GSDME to execute pyroptosis [420]. Finally, overcoming NFS1‐mediated Fe–S cluster defense during chemotherapy results in a catastrophic ROS storm, triggering widespread PANoptosis involving ferroptosis, apoptosis, pyroptosis, and necroptosis [111, 137].

Emerging evidence demonstrates that metal‐based agents can provoke a PANoptosis‐like collapse in which multiple death machineries are activated simultaneously, generating a hybrid phenotype capable of circumventing resistance associated with single‐pathway targeting [411, 412, 413] (Figure 10A). For example, in colorectal cancer, deficiency of the Fe–S cluster biosynthetic enzyme NFS1, combined with oxaliplatin treatment, induces a PANoptotic cascade involving apoptosis (caspase‐3/7), necroptosis (p‐MLKL), pyroptosis (GSDME), and ferroptosis (lipid ROS), driven by catastrophic oxidative stress [137]. Likewise, engineered nanoplatforms using copper‐iron or cobalt‐vanadium couples simultaneously activate caspase‐3, GSDMD, and p‐MLKL while depleting GPX4 [47, 398]. In hepatocellular carcinoma, disulfiram/copper treatment induces a cascade of ferroptosis and cuproptosis, with GSH depletion serving as a shared metabolic vulnerability linking lipid peroxidation to copper‐dependent proteotoxic stress [96]. Similarly, cupric‐doped nanoplatforms concurrently trigger apoptosis, ferroptosis, and cuproptosis [414]. These findings suggest that metal‐ion dyshomeostasis, particularly Cu^2+^/Zn^2+^ overload, can act as an upstream trigger for PANoptosome assembly, capable of eliminating heterogeneous tumors containing apoptosis‐sensitive and apoptosis‐resistant populations [415, 416].

In many physiological settings, this crosstalk appears as a sequential hierarchy rather than a simultaneous collapse, whereby metal signaling repurposes the machinery of one death modality to execute another (Figure 10B). In heart failure models, ACSL4‐driven ferroptosis functions as an upstream trigger generating 12‐HETE, which subsequently activates the NLRP3 inflammasome to induce pyroptosis [342, 419]. This establishes a therapeutic hierarchy in which blocking the metal‐dependent initiating step prevents downstream inflammatory execution. Conversely, in melanoma, iron overload is sensed by the mitochondrial protein Tom20, which recruits Bax and activates caspase‐3; however, instead of producing apoptosis, this context redirects caspase‐3 to cleave GSDME, ultimately inducing pyroptosis [420]. Such sequential signaling illustrates that the presence of a canonical executioner protein, such as caspase‐3, does not guarantee a specific morphological outcome, because the ultimate fate depends on the surrounding metal‐dependent cellular context [421, 422].

The decision between these death modalities is frequently governed by metabolic checkpoints and shared regulatory nodes. Fluctuations in metabolite availability can redirect cells toward alternative potassium efflux‐dependent fates [423]. For instance, in SLC7A11‐high tumors, glucose deprivation shifts cells from ferroptosis resistance toward disulfidptosis, a process driven by actin cytoskeleton collapse following NADPH depletion [39, 424]. Similarly, circadian regulators such as BMAL1 modulate copper toxicity in glioblastoma, linking circadian rhythmicity with metal susceptibility [259, 425]. This regulation extends to organelle‐level communication in which mitochondria, lysosomes, and the ER coordinate metal signaling [162, 348, 426]. Phospholipids containing two polyunsaturated acyl chains accumulate in mitochondria but generate ROS that drives ferroptosis primarily in the ER [92], whereas mitochondrial ROS can activate lysosomal pathways that feedback to trigger mitochondrial calcium overload and calcicoptosis [162, 288]. In many contexts, autophagy acts upstream of ferroptosis rather than merely in parallel, as NCOA4‐mediated ferritinophagy degrades ferritin to expand the labile iron pool that fuels lipid peroxidation [34, 124].

A major obstacle to progress lies in distinguishing these overlapping pathways within complex tissue environments, where conventional biomarkers often produce ambiguous or misleading interpretations. Classical markers such as TUNEL staining, historically associated with apoptosis, have been detected in ferroptosis [427] and in pyroptosis due to DNA fragmentation [23, 428]. Similarly, zinc‐associated pyroptosis, particularly after ZnO nanoparticle exposure, can yield Annexin V positivity and Annexin V/PI profiles overlapping with late apoptosis [23, 429]. This ambiguity is further amplified by the limited specificity of commonly used pathway inhibitors. Necrostatin‐1, frequently used to define necroptosis, also suppresses ferroptosis through an off‐target mechanism independent of RIPK1 inhibition [53, 136]. Similarly, numerous lipoxygenase inhibitors function as RTAs, which can incorrectly imply that ferroptosis is LOX‐dependent when the process may instead result from spontaneous lipid autoxidation [171]. Because ROS acts as a shared convergence point for ferroptosis, pyroptosis, and paraptosis, the application of broad‐spectrum antioxidants cannot reliably distinguish the specific lethal subroutine [102, 418].

To address these challenges and exploit pathway crosstalk therapeutically, the field must move beyond simple inhibitor‐based screening toward rigorous validation of specific in vivo molecular signatures [430]. Future studies should prioritize pathway‐resolved molecular readouts, such as 15‐HpETE‐PE/oxidized phosphatidylethanolamines for ferroptosis and lipoylated DLAT aggregation for cuproptosis, rather than relying solely on nonspecific oxidative‐stress indicators [431]. Tissue‐specific genetic perturbation of core regulators such as GPX4 or FTH, combined with lipid‐peroxidation readouts and pharmacological or genetic rescue, remains essential for establishing ferroptosis dependency in vivo [9, 53, 109]. Ultimately, deciphering the lethal signaling architecture, the interconnected network of shared nodes and metabolic checkpoints, will enable the development of metallometabolic therapies capable of overriding resistance mechanisms by deliberately inducing PANoptosis or preserving tissue viability through blockade of upstream metal triggers [417, 432].

### Section summary

While calcium, sodium, zinc, manganese, and cobalt govern vital physiological functions, exceeding strict homeostatic thresholds triggers irreversible RCD via distinct molecular mechanisms, causing organelle‐specific bioenergetic collapse. Specifically, calcium drives calcicoptosis via MCU‐mediated mPTP opening. Furthermore, unchecked sodium influx precipitates NECSO via lethal osmolar collapse and reverse NCX activation. Zinc induces zincoptosis through FDX2/LIAS suppression [24] and lysosomal–mitochondrial axis disruption [60], and manganese triggers mnoptosis via the enzymatic mismetallation of CoQ_7_ [75]. Finally, severe cobalt accumulation drives coptosis via pseudohypoxia and m^6^A hypermethylation‐mediated epitranscriptomic toxicity. Importantly, these metal‐dependent programs do not function in isolation; rather, they intersect through extensive in vivo crosstalk and shared metabolic checkpoints to orchestrate sequential hierarchies or concurrent PANoptotic collapse. However, clinical applications of these pathways are constrained by a narrow therapeutic window. Because these metals are indispensable for vital physiological processes, including nerve conduction, muscle contraction, and systemic immunity, untargeted systemic administration risks severe off‐target toxicities, notably calcium‐induced cardiotoxicity and manganese‐induced neurotoxicity. Furthermore, distinguishing these ion‐specific mechanisms from canonical apoptosis or accidental necrosis in vivo remains a significant translational challenge. Overcoming these barriers requires targeted spatiotemporal delivery systems. Stimuli‐responsive metal nanomedicines, including acid‐responsive MOFs and CDT agents, enable TME‐triggered in situ ion release and ROS amplification [433]; emerging Cu/Zn‐based systems further show that ion‐homeostasis disruption can deliberately induce PANoptosis to enhance antitumor immunity [415, 434]. Ultimately, these metals, particularly manganese, function as immunomodulators that activate the cGAS–STING pathway [37], functionally coupling localized cytotoxicity and interconnected lethal networks with systemic antitumor immunity to advance metalloimmunotherapy and the development of precision metallometabolic therapies [435].

## BIDIRECTIONAL THERAPEUTICS AND CLINICAL TRANSLATION

### Overview

**Figure 11:**
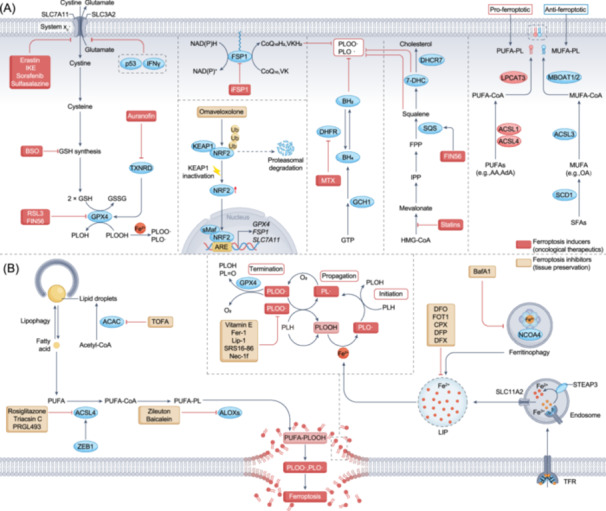
Therapeutic modulation of ferroptosis for oncological synthetic lethality and tissue preservation. (A) Exploiting metabolic vulnerabilities in cancer. The canonical defense involves the SLC7A11/GSH/GPX4 axis, antagonized by system $x_{c}^{-}$ inhibitors (e.g., Erastin, IKE, Sorafenib, and Sulfasalazine), the glutathione (GSH) synthesis inhibitor buthionine sulfoximine (BSO), and the GPX4 inhibitor RSL3, and repressed by p53 and IFNγ, alongside thioredoxin reductase (TXNRD) inhibition by Auranofin [33, 437]. GPX4‐independent cytoprotective mechanisms encompass FSP1‐mediated reduction of ubiquinone and vitamin K (antagonized by iFSP1), the GCH1‐BH4 pathway (susceptible to dihydrofolate reductase [DHFR] inhibition by methotrexate [MTX]), and 7‐DHC accumulation [66]. FIN56 induces ferroptosis by aberrantly activating squalene synthase (SQS) to deplete CoQ_10_ [27, 35, 94], whereas statins restrict the upstream mevalonate pathway by inhibiting HMG‐CoA reductase. Membrane lipid composition fundamentally dictates susceptibility; ACSL1, ACSL4, and LPCAT3 enrich pro‐ferroptotic polyunsaturated phospholipids (PUFA‐PL) [29], whereas SCD1, ACSL3, and MBOAT1/2 incorporate anti‐ferroptotic monounsaturated variants (MUFA‐PL) [70]. NRF2 confers resistance by transcriptionally upregulating antioxidant networks, establishing a cytoprotective node that can be therapeutically amplified by the targeted agonist omaveloxolone. (B) Pharmacological suppression of ferroptosis for tissue preservation. Expansion of the labile iron pool via NCOA4‐mediated ferritinophagy (pharmacologically blocked by bafilomycin A1 [BafA1]) and transferrin receptor uptake drives lipid peroxidation [34, 438]. The peroxidation cascade propagates via reactive alkyl (PL·), alkoxyl (PLO·), and peroxyl (PLOO·) radicals [170]. Lipophilic radical‐trapping antioxidants, such as vitamin E, ferrostatin‐1 (Fer‐1) and liproxstatin‐1 (Lip‐1), alongside the stabilized analog SRS16‐86 and the dual‐targeting inhibitor Nec‐1f, intercept these radicals to halt oxidative propagation [99]. Upstream interventions include iron chelators (deferoxamine [DFO], ciclopirox [CPX], deferiprone [DFP], and deferasirox [DFX]) or lipid synthesis inhibitors (including acetyl‐CoA carboxylase [ACAC] suppression by TOFA; ACSL4 blockade by rosiglitazone, triacsin C, and PRGL493; and ALOXs inhibition by zileuton and baicalein), preventing irreversible membrane permeabilization [14, 29].

The clinical translation of metal‐dependent RCD necessitates a bidirectional therapeutic paradigm: exploiting specific molecular vulnerabilities to selectively eradicate malignancies [14, 17] while pharmacologically inhibiting these same pathways to mitigate pathological cell loss in ischemic, neurodegenerative, and acute organ failure contexts [53] (Figure 11). Anchored in the principles of precision medicine, this dual framework transcends the conventional reliance on nonspecific systemic metal supplementation or broad‐spectrum chelation. Rather, therapeutic efficacy hinges on the precise modulation of compromised homeostatic thresholds, capitalizing on synthetic lethality alongside defined genetic and metabolic liabilities [55]. In oncological settings, oncogenic driver mutations and metabolic rewiring intrinsically deplete the cellular homeostatic reserve against metal‐driven toxicity, rendering malignant populations particularly susceptible to targeted RCD induction and synergistic immunoradiotherapy regimens [72]. Conversely, in non‐neoplastic pathologies, cytoprotection mandates the robust pharmacological reinforcement of endogenous defense networks through highly selective pathway inhibitors and strategically repurposed clinical agents [436]. To systematically articulate this translational landscape, this chapter first delineates the mechanistic basis and active clinical translation for therapeutically exploiting synthetic lethality within oncogenic and metabolic signaling networks. Subsequently, the chapter critically examines advanced pharmacological inhibition strategies, incorporating both targeted modulators and repurposed clinical‐grade drugs, engineered to stabilize endogenous regulatory nodes, establishing a clear molecular blueprint for tissue preservation. Finally, it outlines a multiparametric, biomarker‐guided patient stratification paradigm integrating lipidomic architecture, genetic determinants, metal dyshomeostasis indicators, and metabolic phenotyping to accurately anticipate bidirectional clinical trajectories.

### Exploiting oncogenic and metabolic vulnerabilities

#### Preclinical mechanisms of synthetic lethality

The therapeutic exploitation of metal‐dependent RCD is grounded in the principle of synthetic lethality. Specifically, oncogenic mutations and metabolic rewiring create selective dependencies that can be therapeutically targeted. Although these adaptations enhance proliferation and stress tolerance, they often lower the homeostatic reserve against metal‐driven toxicity, rendering malignant cells more vulnerable than normal tissues [1, 439, 440]. Mapping the interface between oncogenic signaling and metal or redox metabolism can facilitate the development of rational interventions that convert tumor adaptations into fatal liabilities. The discovery of ferroptosis originated from efforts to identify compounds selectively toxic to cells with activated RAS‐MAPK signaling. Early synthetic‐lethal high‐throughput screens in engineered BJ fibroblast‐derived models first identified erastin, and subsequently RSL3 and RSL5, as RAS‐selective lethal compounds that preferentially killed HRAS^V12^‐transformed cells over isogenic non‐HRAS^V12^ controls [441, 442, 443]. This selectivity reflects intrinsic redox stress in RAS‐driven cells, which exhibit elevated basal ROS generation through RAF/MEK/ERK signaling and NADPH oxidases (NOXs) [14]. To buffer this oxidative load, these cells depend on cystine import via system $x_{c}^{-}$ and iron uptake through transferrin receptor (TFRC) [95]. Inhibition of system $x_{c}^{-}$ or GPX4 destabilizes this balance and precipitates ferroptosis [184]. Furthermore, the execution of this ferroptotic cascade strictly requires transferrin import and active glutaminolysis [438]. FIN56 exemplifies this approach by promoting GPX4 degradation and reducing CoQ_10_ levels through interference with the mevalonate pathway, demonstrating preferential lethality in HRAS‐mutant settings [94]. Importantly, this vulnerability is context‐dependent; in malignancies such as rhabdomyosarcoma, RAS mutations can instead confer resistance, necessitating careful molecular stratification. These actionable oncogenic and metabolic vulnerabilities are systematically summarized in Figure 11A.

The tumor suppressor *p53* functions as a rheostat for ferroptosis sensitivity through transcription‐dependent and alternative mechanisms [72]. Wild‐type *p53* represses *SLC7A11*, limiting cystine uptake and lowering the ferroptotic threshold [72, 197]. The acetylation‐defective mutant *p53* ^3KR^, despite losing canonical apoptotic and cell cycle arrest functions, retains the capacity to repress *SLC7A11* and suppress tumorigenesis via ferroptosis [72, 444]. In addition, *p53* activation releases ALOX12 from *SLC7A11*‐mediated inhibition, enabling ALOX12‐dependent oxidation of membrane PUFAs independent of direct GPX4 inhibition [445]. Distinct mutations further modify susceptibility; cells harboring *p53* ^R273H^ display heightened sensitivity to the thioredoxin reductase inhibitor Auranofin, which induces ferroptosis through GPX4 downregulation, a phenotype absent in *p53*‐null or *p53* ^R175H^ contexts [437].

Conversely, constitutive activation of the NRF2–KEAP1 axis constitutes a major barrier to ferroptosis induction. *KEAP1* loss‐of‐function mutations, frequent in lung adenocarcinoma, produce sustained NRF2 activation and upregulation of key ferroptosis defense genes, notably *SLC7A11* and *FSP1*, establishing a reinforced antioxidant shield [107]. However, this adaptation imposes metabolic constraints. KEAP1‐deficient tumors rely on glutamate‐cysteine ligase (GCLC) to prevent glutamate toxicity under cystine starvation; inhibition of GCLC with BSO induces synthetic lethality in this context [446, 447]. Because these tumors depend heavily on the FSP1‐CoQ_10_ axis, they remain susceptible to FSP1 inhibition or blockade of CoQ biosynthesis even when resistant to GPX4 inhibition [123]. Furthermore, certain aggressive malignancies, such as pancreatic ductal adenocarcinoma, exhibit an extreme synthetic lethal dependency on cystine import to buffer KRAS‐driven oxidative stress, making them hypersensitive to targeted cysteine depletion via engineered cyst(e)inase [135]. Suppression of NRF2 can further enhance sensitivity by altering iron homeostasis through HERC2‐mediated regulation of NCOA4, combined with ferritinophagy blockage and expansion of the labile iron pool [158].

Cellular plasticity, particularly epithelial‐to‐mesenchymal transition and the acquisition of a therapy‐resistant persister state [55], also shapes ferroptotic vulnerability through remodeling of membrane lipid composition. Mesenchymal cells with elevated ZEB1 and ACSL4 expression accumulate long‐chain PUFAs, increasing reliance on GPX4 [55]. PKCβII‐mediated phosphorylation of ACSL4 further amplifies lipid remodeling [69]. Resistance frequently arises through enrichment of MUFAs via SCD1, which suppresses ferroptosis. Targeting SCD1 restores susceptibility in ovarian cancer stem cells [26]. In MYC‐driven tumors, loss of DHCR7 leads to accumulation of 7‐DHC, an endogenous radical‐trapping antioxidant that mitigates oxidative damage [66]. Hormone‐responsive malignancies may upregulate MBOAT1/2 to remodel phospholipids and reduce PUFA content, a phenotype reversible by hormone antagonists [70]. Moreover, this targeted induction strategy heavily synergizes with cancer immunotherapy, as CD8^+^ T cell‐derived IFN‐γ actively represses SLC7A11 to promote tumoral lipid peroxidation [103].

High *SLC7A11* expression confers oxidative resistance but imposes significant metabolic demand, because cystine reduction requires substantial NADPH [199]. This creates vulnerability to glucose deprivation, which limits NADPH production via the Pentose Phosphate Pathway [448]. In *SLC7A11*‐high tumors, energetic stress promotes accumulation of disulfide bonds and cytoskeletal collapse, a process termed disulfidptosis [39]. This susceptibility can be exploited using GOx‐based systems or GLUT inhibitors [273]. Stability of *SLC7A11* is itself regulated by deubiquitinases such as OTUB1 and by UFMylation machinery; disruption of these regulators destabilizes the transporter and promotes ferroptosis [208, 209, 449].

Synthetic lethality also applies to cuproptosis, which selectively targets lipoylated TCA cycle proteins. Sensitivity to copper ionophores correlates with mitochondrial respiratory dependence: cells relying on OXPHOS are markedly more susceptible to copper‐ionophore‐induced death than glycolysis‐dependent cells [17, 450]. Oncogenic programs can intensify this state. In MYC‐driven, IDH1‐high group‐3 medulloblastoma, c‐MYC induces DLAT, a key lipoylated PDC component, thereby sensitizing tumor cells to elesclomol‐induced cuproptosis [451]. Similarly, wild‐type IDH1 epigenetically sustains the c‐MYC–DLAT axis, priming MYC‐driven group‐3 medulloblastomas for elesclomol‐induced cuproptosis [451], while ARID1A deficiency in hepatocellular carcinoma forces a metabolic shift toward the TCA cycle, creating a synthetic lethal vulnerability to the copper ionophore elesclomol [246]. Conversely, hypoxia and HIF‐1α signaling suppress mitochondrial respiration and induce metallothioneins that sequester copper, promoting resistance [28]. Additional metal‐dependent vulnerabilities demonstrate similar precision; zinc dysregulation via TRPML1 activation induces mitochondrial dysfunction in metastatic melanoma [22, 60], whereas arsenic trioxide exploits the PML‐RARα fusion in acute promyelocytic leukemia [452, 453, 454]. Collectively, these findings underscore that metal‐dependent RCD constitutes a targeted therapeutic strategy aligned with the genetic and metabolic configuration of cancer cells rather than a nonspecific toxic insult. The therapeutic rationale for targeting cuproptosis in oncology is systematically evaluated in recent literature [455, 456].

#### Clinical translation in oncology

Clinically, the strategic repurposing of FDA‐approved drugs and the implementation of combinatorial regimens have markedly accelerated the clinical translation of metal‐dependent RCD, as comprehensively detailed in Table 2. In the context of ferroptosis induction, retrospective analyses of the Phase III STORM and IMbrave150 trials revealed that patients with HCC who exhibit ferroptosis‐initiation signatures, specifically defined by the KUSS50 biomarker and ACSL4 upregulation, experience significantly greater therapeutic benefit from the multikinase inhibitor sorafenib [457]. Furthermore, targeted combinatorial strategies have exhibited profound efficacy in overcoming radioresistance and immunoresistance. A completed Phase II clinical trial (ChiCTR2200066117) established that targeting SLC7A11 with sorafenib successfully sensitizes colorectal cancer liver metastases to stereotactic body radiotherapy (SBRT). This regimen significantly prolonged progression‐free survival (PFS), with posttreatment biopsies confirming the targeted in vivo execution of ferroptosis via markedly elevated 4‐HNE levels [458]. Radiotherapy‐induced ferroptosis is also being actively leveraged to potentiate novel immunotherapeutic combinations. A Phase I clinical trial (E20240009) utilizing low‐dose radiotherapy alongside anti‐PD‐1 blockade in treatment‐refractory nonsmall cell lung cancer achieved a preliminary objective response rate of 33.3%, driven by the explicit inhibition of the NRF2/HO‐1/GPX4 antioxidant axis to trigger ferroptosis [459]. Similarly, an investigator‐initiated prospective trial (ChiCTR2100053441) in advanced HCC demonstrated that concurrent administration of the TLR3 agonist Poly(I:C) and radiotherapy robustly promotes CD8^+^ T cell‐mediated tumoral ferroptosis, dramatically enhancing the abscopal effect against distant metastatic lesions [460]. Repurposed agents targeting ferroptosis‐related redox vulnerabilities have also entered early oncologic evaluation: sulfasalazine, a system x_c_^−^/xCT inhibitor, induces ferroptosis in glioma models, whereas auranofin inhibits thioredoxin reductase/TXNRD1 and can promote ferroptosis‐associated oxidative stress; however, their clinical efficacy across gliomas and other solid tumors remains unproven [184, 461, 462]. Translational strategies exploiting ferroptosis to overcome therapy resistance are deeply examined in recent literature [125, 463, 464]. Similarly, stabilizing the SEC. 62‐TRPM4 axis with cinobufagin deliberately provokes lethal sodium overload, offering a targeted translational strategy to bypass acquired chemoresistance in refractory multiple myeloma [82].

**Table 2:** Summary of ongoing and completed clinical trials targeting metal‐dependent regulated cell death pathways.

Intervention	Targeted pathway	Disease context	Trial phase and ID (Status)	Key clinical outcomes	References
Sorafenib	Ferroptosis induction (SLC7A11/ACSL4)	HCC	Phase III (STORM, IMbrave150) (completed)	Evaluated KUSS50 ferroptosis signature; predicted maximal clinical benefit; validated ACSL4 biomarker	[457]
Sorafenib + SBRT	Ferroptosis induction (SLC7A11 blockade)	mCRC (liver metastases)	Phase II (ChiCTR2200066117) (completed)	Prolonged PFS; overcame radioresistance; achieved high objective response (3 CRs, 8 PRs); elevated tumoral 4‐HNE	[458]
LDR + Anti‐PD‐1	Ferroptosis induction (NRF2 axis suppression)	Refractory NSCLC	Phase I (E20240009) (ongoing)	Achieved 33.3% ORR; overcame chemoimmunotherapy resistance; executed tumoral ferroptosis and elevated IFN‐γ	[459]
Poly(I:C) + RT	Ferroptosis induction (CD8^+^ T cell‐mediated)	Advanced HCC	Prospective clinical trial (ChiCTR2100053441) (ongoing)	Enhanced abscopal effect; promoted distant metastasis regression (1 CR, 3 PRs); reshaped immunosuppressive TME	[460]
ACXT‐3102	Ferroptosis induction (xCT inhibition)	Synovial sarcoma	Phase I (NCT02683148) (ongoing)	Exploited ME1‐null vulnerability; enhanced intracellular drug import; triggered lethal lipid peroxidation	[440]
ATI‐450	Ferroptosis sensitization (MK2 inhibition)	Metastatic BC	Phase I/II (NCT06374459) (ongoing)	Reversed ferroptosis resistance; suppressed MK2‐HSPB1 signaling; evaluated clinical tolerability	[449]
Elesclomol ± PTX	Cuproptosis induction (mitochondrial proteotoxicity)	Advanced melanoma	Phase III (SYMMETRY) (completed)	Prolonged PFS in low‐LDH cohort; failed unselected primary endpoint; required metabolic stratification	[17, 465, 466]
Disulfiram + CTx	Cuproptosis/ferroptosis induction	Recurrent GBM, NSCLC	Phase II/III (NCT02678975) (completed)	Induced dual cell death; failed to improve OS and had higher AEs in recurrent GBM; retrospective continuous use linked to improved NSCLC OS	[240, 343, 467, 468]
Deferiprone (DFP)	Ferroptosis inhibition (iron chelation)	Early‐stage PD	Phase II/III (FAIRPARK‐II, NCT02655315, NCT01539837) (completed/ongoing)	Reduced nigrostriatal iron deposition; worsened motor progression (levodopa‐naïve); highlighted stratification necessity	[101, 469, 470, 471]
Deferoxamine (DFO)	Ferroptosis inhibition (iron chelation)	Acute ischemic stroke	Phase II (NCT00777140) (completed)	Sequestered unstable iron pools yielded 90‐day neurological recovery and demonstrated clinical safety	[101, 472]
DFX + DFO	Ferroptosis inhibition (iron chelation)	Severe myocardial siderosis	Phase II (NCT01254227) (completed)	Reduced myocardial iron; improved cardiac ejection fractions; mitigated tissue degeneration	[101, 473]
Dipyridamole (DIPY)	Ferroptosis inhibition (SOD1/CREB1/HMOX1 axis)	ARDS	Phase II (ChiCTR2300078059) (completed)	Improved PaO_2_/FiO_2_ ratio; reduced SOFA scores; decreased serum MDA.	[474]
Tetrathiomolybdate (TTM)	Cuproptosis inhibition (copper chelation)	Wilson's disease/high‐risk BC	Phase II (NCT00195091) (completed/ongoing)	Neutralized aberrant copper; prevented proteotoxic stress; achieved 84% 6.3‐year OS (BC cohort)	[240, 475, 476]
Omaveloxolone	Ferroptosis inhibition (NRF2 agonism)	Friedreich's ataxia	Phase III (FDA approved)	Bolstered cellular antioxidant resistance; alleviated neurodegenerative pathology; validated clinical utility	[101, 477]
NAC/CoQ_10_	Ferroptosis inhibition (GSH precursor/antioxidant)	ALF/acute MI/CAD	Phase II/III (NCT00004467, NCT01501110, NCT01424761) (completed)	Augmented antioxidant enzyme activity; attenuated oxidative disturbances; improved transplant‐free survival	[101, 478, 479]

Abbreviations: AEs, adverse events; ALF, acute liver failure; ARDS, acute respiratory distress syndrome; BC, breast cancer; CAD, coronary artery disease; CR, complete response; CREB1, cAMP responsive element binding protein 1; CTx, chemotherapy; DFO, deferoxamine; DFP, deferiprone; DFX, deferasirox; DIPY, dipyridamole; GBM, glioblastoma multiforme; GSH, glutathione; HMOX1, heme oxygenase 1; HO‐1, heme oxygenase‐1; LDH, lactate dehydrogenase; LDR, low‐dose radiotherapy; mCRC, metastatic colorectal cancer; ME1, malic enzyme 1; MI, myocardial infarction; MK2, MAPK‐activated protein kinase 2; NAC, N‐acetylcysteine; ORR, objective response rate; OS, overall survival; PD, Parkinson's disease; PD‐1, programmed cell death protein 1; PFS, progression‐free survival; PR, partial response; PTX, paclitaxel; RT, radiotherapy; SBRT, stereotactic body radiotherapy; SOD1, superoxide dismutase 1; SOFA, sequential organ failure assessment; SSZ, sulfasalazine; TME, tumor microenvironment; TTM, tetrathiomolybdate; TXNRD, thioredoxin reductase.

This translational momentum closely parallels advancements in cuproptosis and copper‐targeting therapeutics. The copper ionophore elesclomol has been extensively evaluated across multiple Phase II and Phase III clinical trials for melanoma, ovarian cancer, and prostate cancer, establishing critical clinical proof‐of‐concept for exploiting mitochondrial proteotoxic stress in patients [240, 465, 466]. Crucially, retrospective analyses of these trials underscore the absolute necessity of metabolic biomarker stratification. These data demonstrate that patients with melanoma presenting with low serum lactate dehydrogenase (LDH) levels, a biomarker indicative of a high reliance on mitochondrial phosphorylation, experience the most substantial survival benefits from elesclomol‐induced cuproptosis [465, 466, 480]. Parallel translational efforts are currently investigating disulfiram, a well‐tolerated FDA‐approved drug capable of inducing concurrent copper‐dependent toxicity and ferroptosis. Early clinical evidence suggested a potential survival signal for disulfiram in metastatic non‐small cell lung cancer cohorts [467], whereas the subsequent DIRECT randomized phase II/III trial in recurrent glioblastoma found that adding disulfiram/copper to alkylating chemotherapy did not improve survival and increased toxicity [240, 468].

However, the clinical translation of metal‐dependent RCD is inherently bidirectional; while oncology requires targeted execution, preserving vulnerable tissues in degenerative diseases necessitates the active pharmacological suppression of these exact same pathways.

### Pathway inhibitors of tissue preservation

#### Pharmacological strategies for cytoprotection

The protective strategy in non‐neoplastic pathologies focuses explicitly on fortifying endogenous defense networks. Dysregulated homeostasis of iron, copper, zinc, calcium, and manganese contributes directly to widespread pathological cell loss in IRI, acute organ failure, and severe neurodegenerative diseases such as Alzheimer's disease [168, 481]. Accordingly, therapeutic strategies are evolving from nonspecific chelation, which carries the risk of systemic metal depletion, toward targeted modulation of defined regulatory nodes that reinforce endogenous homeostatic thresholds rather than indiscriminately removing excess ions [482, 483].

Among these modalities, ferroptosis is the most extensively characterized and therefore represents a principal target for intervention. As depicted in Figure 11B, modern interventions intentionally bypass upstream initiation mechanisms to directly halt the propagation phase of lipid peroxidation or stabilize terminal regulatory nodes. Protective strategies converge on three control points—suppression of lipid peroxidation, restriction of labile iron availability, and strengthening of intrinsic antioxidant systems. RTAs, particularly Fer‐1 and liproxstatin‐1 (Lip‐1), remain benchmark inhibitors of ferroptotic injury [436]. Mechanistic analyses indicate that Fer‐1 not only scavenges lipid radicals but also forms a transient complex with Fe^2+^, enabling regeneration of the active inhibitor and establishing a pseudocatalytic protection cycle that surpasses vitamin E under iron‐rich conditions [99]. Therapeutic efficacy has been demonstrated in renal and hepatic models; Lip‐1 prevents acute renal failure in GPX4‐deficient mice and preserves tubular morphology [53, 484], whereas Fer‐1 and the stabilized analog SRS16‐86 attenuate injury in oxalate nephropathy and liver fibrosis [115, 484]. Crucially, dual‐targeting inhibitors like Nec‐1f provide advanced tissue preservation during AKI by simultaneously blocking ferroptosis and necroptosis [9]. Similarly, alpha‐lipoic acid mitigates orthopedic implant‐induced arthroprosthetic cobaltism by chelating cobalt nanoparticles and rescuing GPX4‐dependent ferroptosis in vulnerable tissues [400].

Neuroprotective benefits have also been heavily documented, including reduced neuronal loss and improved cognitive outcomes in models of ischemic stroke and Alzheimer's disease [114, 485]. Clinically, elevated brain iron strongly correlates with accelerated cognitive decline in Alzheimer's pathology [44], and the targeted preservation of FPN expression has been shown to block memory impairment driven by iron‐dependent death [114]. Furthermore, ferroptosis inhibitors provide robust tissue preservation in cardiovascular contexts, significantly mitigating doxorubicin‐induced cardiomyopathy and myocardial IRI [108]. Our group systematically charted these actionable cytoprotective targets, particularly emphasizing the cardiovascular pathogenesis [16, 486]. In parallel, modulation of endogenous metabolites provides an alternative strategy. Inhibition of DHCR7 increases 7‐DHC levels, supplying a potent intrinsic RTA [35], while vitamin K (MK4) supports tissue preservation through FSP1‐dependent reduction pathways [120].

Beyond direct antioxidant reinforcement, inhibition of lipid substrate synthesis represents a complementary strategy. ACSL4, which enriches membranes with polyunsaturated fatty acids, constitutes a central druggable node. Pharmacological inhibition of ACSL4 using thiazolidinediones or selective compounds, such as PRGL493, limits membrane remodeling and preserves tissue integrity in heart failure and AKI [29, 163, 419]. Similarly, targeted enzymatic inhibition of ALOX15 mitigates myocardial IRI by preventing the generation of specific lipid death signals [168]. Targeting upstream amplifiers further enhances protection; blockade of PKCβII prevents ACSL4 phosphorylation and activation [69], and inhibition of the Thrombin‐cPLA_2_α‐ACSL4 axis improves neuronal survival during cerebral IRI. With respect to iron handling, although broad chelators such as deferoxamine retain utility in specific settings, including osteoarthritis [487], refined approaches now focus on LIPs. Suppression of NCOA4‐mediated ferritinophagy limits labile iron release and attenuates ferroptotic damage driven by cigarette smoke, a key factor in COPD pathogenesis [54, 427]; similarly, stabilization of the iron exporter FPN mitigates neurodegenerative pathology [114]. Furthermore, pharmacologically stabilizing MARCH7 with emodinanthrone potently blunts ferroptosis, providing profound in vivo cardioprotection against doxorubicin‐induced and ischemia‐reperfusion myocardial injuries [36].

In contrast to the lipid‐driven framework of ferroptosis, defensive inhibition of cuproptosis similarly bypasses upstream signaling to focus entirely on stabilizing terminal regulatory nodes—specifically preventing the aggregation of lipoylated TCA cycle proteins and preserving mitochondrial metabolism [488]. Tetrathiomolybdate (TM) sequesters bioavailable copper from copper‐trafficking/protein‐bound pools, thereby limiting Cu–TAX1BP1‐dependent autophagic GPX4 degradation and ferroptosis; this copper‐lowering mechanism underlies its relevance to Wilson disease and its protective effect in ferroptosis‐associated acute pancreatitis [187, 489]. However, mitigation of toxicity does not invariably require copper removal. In metabolic insufficiency models, thiamine (vitamin B1) supplementation enhances pyruvate dehydrogenase activity, thereby increasing tolerance to copper without altering systemic copper levels [490]. Additionally, precision delivery of si‐*FDX1* via ROS‐responsive hydrogels halts reactive Cu^+^ generation at the source, preventing cuproptosis and preserving cardiac function during myocardial IRI [491].

Management of zinc‐ and manganese‐associated injury similarly demands pathway‐specific strategies addressing transporter dysfunction and metabolic derailment [337]. During cerebral ischemia, synaptic zinc accumulation contributes to neuronal death; extracellular chelation with CaEDTA [492] or pharmacological downregulation of ZnT3 using Riluzole reduces this burden [493, 494]. Furthermore, highly specific zinc chelators enhance retinal ganglion cell survival in optic nerve crush models, highlighting profound tissue preservation against neurotoxicity [495]. In Type 2 diabetes mellitus, inhibition of ZnT8 protects pancreatic β‐cells from stress‐induced injury [340, 496]. For manganese toxicity, characterized by mismetallation of mitochondrial enzymes, metabolic bypass using 2,4‐dihydroxybenzoic acid restores CoQ biosynthesis and improves tissue viability [75]. Targeting calcicoptosis focuses on the mPTP as the terminal effector. Cyclosporin A and the nonimmunosuppressive analog NIM811 increase the calcium threshold for pore opening and preserve mitochondrial integrity during acute metabolic stress, such as adrenergic activation in brown adipose tissue [85, 290], whereas peptides modeled on the *Artemia* e‐subunit provide a biomimetic strategy to stabilize the F‐ATP synthase leak channel [294]. Neurons in diverse neurodegenerative disease models exhibit elevated basal mitochondrial calcium and reduced buffering capacity, rendering them vulnerable to degeneration; thus, reducing mitochondrial calcium uptake via MCU inhibition represents a potential therapeutic strategy to preserve neuronal viability [497].

#### Clinical progress in tissue preservation

Because benchmark radical‐trapping antioxidants, such as Fer‐1 and Lip‐1, exhibit suboptimal pharmacokinetic properties and limited in vivo stability, current clinical translation for tissue preservation relies predominantly on the strategic repurposing of FDA‐approved pharmacological inhibitors and clinical‐grade metal chelators. Concerning ferroptosis, iron chelation has advanced to robust clinical evaluation, yielding complex but highly informative outcomes. For instance, early‐phase trials demonstrated that the orally active iron chelator deferiprone (DFP) could successfully reduce pathological iron deposition in the substantia nigra in Parkinson's disease [469, 470]. However, the subsequent large‐scale multicenter FAIRPARK‐II clinical trial (NCT02655315) revealed that DFP paradoxically worsened motor progression in levodopa naïve patients. This outcome underscores that although excess iron drives neurotoxicity, basal iron pools remain essential for compensatory dopamine synthesis, a mechanistic dichotomy that highlights the absolute necessity of precise patient stratification [471]. Conversely, in acute ischemic settings in which iron overload acutely exacerbates IRI, the clinical‐grade iron chelator DFO has displayed substantial therapeutic promise. A completed Phase II clinical trial (NCT00777140) for acute ischemic stroke demonstrated the safety of DFO administration, and the drug yielded highly encouraging 90‐day neurological recovery by rapidly sequestering unstable iron pools [101, 472]. Moreover, in the HYPERION trial (NCT01254227), combined deferasirox–deferoxamine therapy improved myocardial T2* and markedly reduced liver iron burden in patients with severe transfusional myocardial siderosis, while left ventricular ejection fraction remained stable [101, 473].

Beyond direct chelation, the repurposing of established systemic agents to target upstream regulatory nodes of metal‐dependent cell death is gaining significant clinical traction. Preclinically, the FDA‐approved system x_c_^−^ inhibitor sulfasalazine mitigates cancer‐induced depression by preventing tumoral glutamate release [498]. A recent proof‐of‐concept clinical trial (ChiCTR2300078059) identified dipyridamole (DIPY), an FDA‐approved antiplatelet drug, as a potent ferroptosis inhibitor operating via the SOD1/CREB1/HMOX1 signaling axis. In patients with acute respiratory distress syndrome (ARDS), adjunctive DIPY therapy significantly improved oxygenation, reduced Sequential Organ Failure Assessment scores, and markedly decreased the levels of serum malondialdehyde (MDA), a definitive in vivo biomarker of ferroptotic lipid peroxidation [474]. Furthermore, augmenting endogenous antioxidant defenses has yielded notable clinical benefits. Omaveloxolone, an Nrf2 agonist that bolsters cellular resistance against ferroptosis, recently received FDA approval for the treatment of Friedreich's ataxia, further validating the clinical utility of targeting this pathway [101, 477].

Parallel translational momentum is evident in the mitigation of copper‐dependent toxicity and broader metabolic insufficiency. Tetrathiomolybdate (TTM), a highly specific copper chelator that prevents cuproptotic proteotoxic stress, demonstrated marked efficacy in Phase II clinical trials for Wilson's disease by efficiently neutralizing aberrant intracellular copper accumulation and improving both hepatic and neurological symptoms [499]. Underscoring the bidirectional translational utility of these metallotherapeutics, TTM has also been effectively deployed as an adjuvant therapy in oncology, achieving an overall survival rate of 84% over 6.3 years in a Phase II trial (NCT00195091) for high‐risk breast cancer [475, 476]. Additionally, clinical trials targeting metal‐induced oxidative stress with established metabolic cofactors are rapidly advancing. Intravenous N‐acetylcysteine, a glutathione precursor, has improved transplant‐free survival during early acute liver failure (NCT00004467) [478] and attenuated oxidative disturbances in acute myocardial infarction (NCT01501110) [500], whereas CoQ_10_ supplementation significantly enhanced antioxidant enzyme activity in a randomized trial (NCT01424761) of patients with coronary artery disease [101, 479]. Collectively, these clinical trial data validate that strategically reinforcing endogenous metal homeostatic thresholds with repurposed clinical‐grade inhibitors provides a robust and immediately actionable pathway for organ and tissue preservation, as summarized in Table 2.

Despite these advances, translational challenges persist, particularly regarding temporal dynamics and pathway convergence. In chronic toxic exposures, such as arsenic injury, ferroptotic signaling may become entrenched following irreversible mitochondrial damage, reducing responsiveness to conventional inhibitors [198]. In complex tissues, concurrent activation of multiple regulated death programs, collectively termed PANoptosis, may necessitate upstream metabolic reprogramming rather than isolated pathway blockade [87]. Thus, durable tissue preservation will likely depend on proactive modulation of the molecular circuitry governing metal‐dependent lethality rather than reactive chelation alone.

### Biomarker‐guided patient stratification

To safely execute the bidirectional tumor‐ablative and tissue‐preserving strategies discussed in Sections 5.1 and 5.2, interventions must be carefully calibrated against patient‐specific homeostatic thresholds. Consequently, effective clinical translation demands robust biomarker‐guided stratification, mapping individual lipidomic, genetic, and metabolic profiles to dictate the optimal deployment of metal‐dependent modulators, as comprehensively detailed in Table 3.

**Table 3:** Metal‐dependent regulated cell death biomarkers and treatment strategies.

Key biomarker	RCD pattern	Biomarker category	Core mechanism	Translational strategy and effect
ACSL4	Ferroptosis	Lipid metabolism	Enriches membranes with oxidizable PUFAs [29].	Oncology: Predicts sensitivity to ferroptosis inducers [29]. Tissue injury: Identifies high‐risk tissues for ferroptotic damage in AKI/stroke [501].
7‐DHC/DHCR7	Ferroptosis	Lipid metabolism	Traps radicals shielding membranes from autoxidation [35, 66].	Oncology: Identifies tumors shielded from peroxidation, indicating a need for metabolic priming [35].
PUFA‐ePLs/PC‐PUFA_2_s	Ferroptosis	Lipid metabolism	Provides distinct lipid substrates driving peroxidation [116].	Oncology: Stratify patients for GPX4 inhibitors [116]. Tissue Injury: Oxidized species predict necrosis [30].
MDA/4‐HNE	Ferroptosis	Lipid metabolism	Toxic aldehydes from unrestricted lipid peroxidation.	Tissue injury: Predicts IRI/neurodegeneration severity; guides RTA dosing [53].
SLC7A11/OTUB1	Ferroptosis	Antioxidant defense	Imports cystine supporting cellular glutathione synthesis [208].	Oncology: Deploy inhibitors overcoming therapy resistance [502]. Tissue injury: Depletion indicates ischemic susceptibility [503].
GPX4	Ferroptosis	Antioxidant defense	Reduces toxic lipid hydroperoxides, preventing ROS [56].	Oncology: Deploy inhibitors triggering ferroptotic death [43]. Tissue injury: Depletion identifies preservation candidates [504].
Brain Iron/CSF ferritin	Ferroptosis	Metal metabolism	Reflects pathological CNS labile iron expansion [14].	Tissue injury: Predicts cognitive decline; guides iron chelation [505].
FSP1	Ferroptosis	Antioxidant defense	Regenerates CoQ_10_, trapping lipid peroxyl radicals [506].	Oncology: Target FSP1 overcoming GPX4 resistance [506].
p53	Ferroptosis	Genetics & drivers	Represses SLC7A11, lowering ferroptotic survival threshold [72].	Oncology: Exploit specific mutant variants to confer selective sensitivity to redox‐active agents [507].
NF2/YAP	Ferroptosis	Genetics & drivers	Upregulates pro‐ferroptotic ACSL4 and TFRC transcription [166].	Oncology: Exploit NF2 loss to increase susceptibility to ferroptosis [166].
CD8^+^ T cells/IFN‐γ	Ferroptosis	Tumor microenvironment	Downregulates SLC7A11, promoting tumor lipid peroxidation [103].	Oncology: Exploit immune signals to reduce SLC7A11 expression and sensitize tumors to ferroptosis [103].
FDX1	Cuproptosis	Genetics & drivers	Reduces Cu^2+^, facilitating lipoylated protein aggregation [17].	Oncology: Deploy copper ionophores triggering cell death [508].
DLAT (lipoylated)	Cuproptosis	Mitochondrial metabolism	Binds copper, causing toxic protein oligomerization [17].	Oncology: Induce aggregation overcoming therapeutic resistance [508]. Tissue injury: Marks neurodegenerative proteotoxic stress [509].
ARID1A	Cuproptosis	Genetics & drivers	Enforces mitochondrial respiration, creating synthetic vulnerability [246].	Oncology: Treat with ionophores exploiting TCA reliance [246].
HIF‐1α/MT2A	Cuproptosis	Genetics & drivers	Suppresses metabolism, inducing protective copper sequestration [28].	Oncology: Combine inhibitors with copper ionophores [28].
ATP7B/serum Cp	Cuproptosis	Metal transporters	Mediates copper efflux, preventing intracellular accumulation.	Oncology: Downregulate ATP, driving intracellular toxicity [510]. Tissue injury: Mutations predict Wilson's hepatotoxicity [511].

Abbreviations: HIF‐1α, hypoxia‐inducible factor 1‐alpha; KEAP1, Kelch‐like ECH‐associated protein 1.

#### Lipidomic and metabolic signatures

For ferroptosis, susceptibility is mainly dictated by membrane lipid architecture, specifically the abundance and configuration of PUFAs. ACSL4 functions as a key biomarker because it directs the incorporation of arachidonic and adrenic acids into phospholipids, establishing substrates for lipid peroxidation [29]. Clinical observations associate high ACSL4 expression with enhanced responsiveness to immunotherapy and improved survival in melanoma, whereas ACSL4‐deficient tumors, including melanoma and colon cancer subtypes, display intrinsic resistance to ferroptosis and immunotherapy [68]. High‐resolution lipidomic analyses refine this prediction. Enrichment of PUFA‐ePLs, generated by AGPS, characterizes hypersensitive tumors such as clear cell renal cell carcinoma [116]. Similarly, elevated phosphatidylcholine species containing two PUFA chains (PC‐PUFA_2_s) distinguish responsive models more accurately than overall lipid saturation indices [92]. Conversely, accumulation of monounsaturated fatty acids driven by SCD1 or elevation of 7‐DHC due to DHCR7 loss‐of‐function identifies tumors shielded from peroxidation and potentially requiring metabolic priming [26, 35, 66]. Importantly, these identical lipidomic signatures provide critical predictive value for non‐neoplastic tissue damage. Elevated ACSL4 expression and the rapid accumulation of specific oxidized polyunsaturated phospholipids (e.g., 15‐HpETE‐PE), alongside canonical end‐products like MDA and 4‐HNE, signify a precipitously lowered ferroptotic threshold. In AKI and ischemic stroke, these biomarkers identify highly susceptible tissues requiring immediate intervention with radical‐trapping inhibitors [485, 501, 512, 513]. Furthermore, in neurodegenerative diseases, dysregulated iron metabolism markers, such as elevated cerebrospinal fluid (CSF) ferritin and localized brain iron deposition quantified via magnetic resonance imaging (MRI) quantitative susceptibility mapping (QSM), predict neuronal susceptibility to lipid peroxidation, functioning as early prognostic biomarkers for accelerated cognitive decline in Alzheimer's and Parkinson's diseases [44, 505].

In contrast, cuproptosis stratification centers on mitochondrial metabolic state and lipoylation status. Copper‐induced lethality is contingent on active TCA cycle flux; tumors dominated by glycolytic metabolism or sustained hypoxia exhibit relative resistance due to suppressed mitochondrial respiration [28, 508]. High expression of FDX1 and lipoylated DLAT represents positive predictive markers in malignant settings [230, 239]. In HCC and TNBC, elevated DLAT correlates with adverse prognosis yet predicts responsiveness to copper ionophores [251, 514]. Resistance is mediated by HIF‐1α‐driven metabolic rewiring and MT2A‐dependent mitochondrial copper sequestration [28]. Conversely, alterations that enforce OXPHOS, including ARID1A deficiency, generate synthetic vulnerability to copper‐mediated death [246]. Beyond oncology, assessing copper dyshomeostasis is equally critical for anticipating severe tissue degeneration. In Wilson's disease, biallelic pathogenic ATP7B variants and hypoceruloplasminemia are established diagnostic markers of impaired biliary copper export and systemic copper overload, identifying patients who require early copper‐lowering therapy. Mechanistically, copper overload may further promote cuproptosis‐like mitochondrial proteotoxicity through copper binding to lipoylated TCA‐cycle proteins, such as DLAT, although this axis remains better established in experimental models than as a clinical biomarker [509, 510, 511, 515].

#### Genetic determinants and redundant defense nodes

Oncogenic drivers and tumor suppressor alterations further shape metal‐dependent dependencies. The NF2‐YAP pathway is a prominent determinant of ferroptosis sensitivity; NF2 loss results in YAP activation and transcriptional upregulation of ACSL4 and TFRC, increasing susceptibility [166]. *p53* status also influences response in a context‐dependent manner. Wild‐type *p53* lowers the ferroptotic threshold through repression of *SLC7A11* [72, 507] and enhancement of mitochondrial respiration via circFRMD4A [215], whereas specific mutant variants confer selective sensitivity to redox‐active agents such as auranofin [437]. KEAP1/NRF2 signaling provides another stratification axis. KEAP1 deficiency drives NRF2 activation and induction of *SLC7A11*, GPX4, and FSP1, conferring resistance to canonical inducers [150, 503, 516]. However, this antioxidant adaptation creates reliance on the FSP1‐CoQ_10_ pathway and GCLC‐dependent glutamate detoxification, identifying NRF2‐high tumors as candidates for FSP1 inhibition rather than GPX4‐directed strategies [123, 446]. In non‐neoplastic injury settings, such as ischemic stroke and acute kidney injury, disruption of the NRF2–SLC7A11/GPX4 antioxidant axis promotes ferroptotic neuronal and renal tubular cell loss [517, 518]. Evaluating the status of these antioxidant defense nodes serves as an essential diagnostic indicator of a critically exhausted homeostatic reserve, profiling tissues that are highly vulnerable to ischemia‐reperfusion injury and thus require immediate antioxidant reinforcement [502, 504, 519, 520].

Considering the redundancy within the antioxidant systems, patient selection must evaluate multiple axes simultaneously. Elevated *SLC7A11* expression is a strong indicator of ferroptosis resistance, but predictive accuracy depends on regulators, such as OTUB1 and CD44, which stabilize the transporter [183, 208]. FSP1 expression should be assessed independently of GPX4, as high FSP1 can sustain survival despite GPX4 inhibition [27, 56, 506]. Similarly, the GCH1‐BH_4_ pathway constitutes an independent protective module; high GCH1 expression predicts reduced sensitivity unless combined with DHFR inhibitors such as methotrexate [57, 121]. Simultaneously, metal efflux mechanisms influence outcome: prominin‐2‐mediated iron export via exosomes [84] and ATP7B‐driven copper efflux [251] act as negative predictors for ferroptosis and cuproptosis efficacy, respectively, in oncological contexts. Conversely, in a bidirectional framework, the failure or suppression of these vital efflux systems acts as a direct harbinger of toxic metal accumulation, robustly predicting progressive tissue vulnerability in degenerative conditions.

#### Emerging biomarkers for multiplexed modalities

Emerging biomarkers extend stratification to additional ion‐driven modalities and highlight contributions from the TME and damaged tissue microenvironments. SLC39A10 marks a JAK–STAT‐linked zinc‐homeostatic vulnerability in B‐cell lymphoma, ZnT3‐dependent synaptic zinc release, and pathological zinc accumulation lower the threshold for zinc‐mediated neurotoxicity [332, 521]. In calcium‐dependent injury, altered MCU‐complex stoichiometry—especially reduced MICU1:MCU or MICU1/2:MCU ratios and MICU2 insufficiency—lowers the threshold for mitochondrial Ca^2+^ uptake, thereby sensitizing tissues to Ca^2+^ overload and injury [522, 523]. Crucially, monitoring these MCU complex alterations provides vital prognostic insights into cellular vulnerability during non‐neoplastic pathologies. Tissues exhibiting defective calcium extrusion or constitutive MCU activation are highly susceptible to calcicoptosis following acute ischemic events or neurodegeneration, allowing clinicians to preemptively deploy MCU inhibitors to preserve neuronal viability [497]. These intrinsic determinants are modulated by immune signals; CD8^+^ T‐cell infiltration and IFN‐γ secretion reduce *SLC7A11* expression and sensitize tumors to ferroptosis [103], whereas TYRO3 signaling promotes resistance [203]. Accordingly, precision application of metal‐dependent RCD requires multiparametric profiling of metabolic substrates, positive regulators, and counter‐regulatory defenses to identify the accessible lethal program within an individual tumor or to anticipate and selectively prevent pathological cell loss in vulnerable tissues [264].

### Section summary

The clinical translation of metal‐dependent RCD requires advancing from nonspecific metal modulation to bidirectional therapeutic strategies guided by metabolic and genetic profiling and emerging clinical trial data. In oncology, exploiting synthetic lethality or metabolic vulnerabilities associated with RAS hyperactivation [14], specific p53 mutations [72], or IDH1‐driven cuproptosis vulnerability [451], selectively induces RCD in malignant cells exhibiting reduced homeostatic reserves. Conversely, mitigating ischemic, neurodegenerative, and acute organ injuries necessitates targeted pharmacological inhibitors, such as Fer‐1 [14], Lip‐1 [53], dual‐targeting agents like Nec‐1f, or ACSL4 antagonists [29], alongside strategically repurposed clinical agents like DIPY and omaveloxolone. These agents suppress lipid peroxidation, stabilize terminal execution nodes, and preserve cellular integrity, avoiding the systemic toxicity of broad‐spectrum metal chelation. Because RCD regulatory networks exhibit substantial redundancy (enabling tumors to circumvent GPX4 blockade via FSP1 [27] or GCH1‐BH_4_ pathways [57], or resist cuproptosis through HIF‐1α signaling and ATP7B‐mediated efflux), evaluating isolated biomarkers like SLC7A11 or FDX1 provides incomplete predictive value. Accurate clinical prognostication requires comprehensive multiparametric profiling that integrates lipidomic architecture (including specific oxidized species like 15‐HpETE‐PE), metabolic phenotyping (such as serum lactate dehydrogenase and ceruloplasmin), organ‐specific metal dyshomeostasis signatures (e.g., magnetic resonance imaging‐quantified brain iron), and driver mutation analysis. Ultimately, advancing precision therapeutics relies on integrated multi‐omics metabolic targeting. Future applications depend on rationally designed multi‐target combinations (e.g., co‐inhibiting FSP1 and GCLC in NRF2‐hyperactive tumors), modulating cellular sensitivity via nutritional or targeted enzymatic interventions (e.g., glucose deprivation to induce disulfidptosis in SLC7A11‐high malignancies [39]), and regulating upstream signaling pathways to manage concurrent cell death programs, including PANoptosis [137].

## BIORESPONSIVE NANOMEDICINE AND SPATIOTEMPORAL DELIVERY

### Overview

The clinical translation of conventional small‐molecule metallotherapeutics is fundamentally hindered by dose‐limiting systemic toxicity and indiscriminate tissue distribution. To overcome these bottlenecks, advanced nanomedicines and bioresponsive delivery platforms leverage precise materials engineering to spatiotemporally restrict lethal metal accumulation to pathological niches, thereby broadening the therapeutic window [95]. Central to this evolution is a paradigm shift driven by chemodynamic therapy (CDT) and SACs: a transition from stoichiometric drug‐target interactions to catalytically amplified mechanisms [132, 524]. By exploiting low pH and elevated GSH in tumors and biofilm‐associated infection microenvironments, these catalytic architectures accelerate Fenton‐like ROS generation while weakening antioxidant defenses, thereby driving oxidative stress above cytotoxic thresholds [525, 526]. To systematically delineate these translational frontiers, this chapter first examines the rational design of SACs and advanced nanozymes with atomic‐level precision [527]. Subsequently, it evaluates the mechanistic release logic of multistimuli‐responsive and biomimetic delivery systems, elucidating how diverse biological carriers (such as extracellular vesicles, protein carriers, and engineered living vectors) synergize to orchestrate interconnected cell death networks and potent spatiotemporal immunomodulation. Finally, the chapter explores the cross‐kingdom applicability of these metal homeostasis‐targeted strategies, highlighting their adaptation as next‐generation antimicrobial therapeutics to eradicate the immunosuppressive tumor microbiome and multidrug‐resistant bacteria [528].

### Chemodynamic therapy and single‐atom catalysts

Moving beyond simple metal ion delivery, CDT relies on the rational design of single‐atom catalysts (SACs) and advanced nanozymes. These systems are engineered to emulate and amplify peroxidase (POD) or oxidase (OXD) activities with catalytic efficiencies that exceed those of native enzymes and conventional metal oxide nanoparticles [413, 529]. By anchoring isolated metal atoms on defined supports, SACs maximize atom utilization and tune metal–support coordination, thereby enhancing TME‐responsive ROS generation and promoting ferroptosis‐ or cuproptosis‐associated tumor therapy [132, 530]. The precise chemical design principles underlying these multifunctional magnetic nanomaterials are comprehensively summarized elsewhere [531].

**Figure 12:**
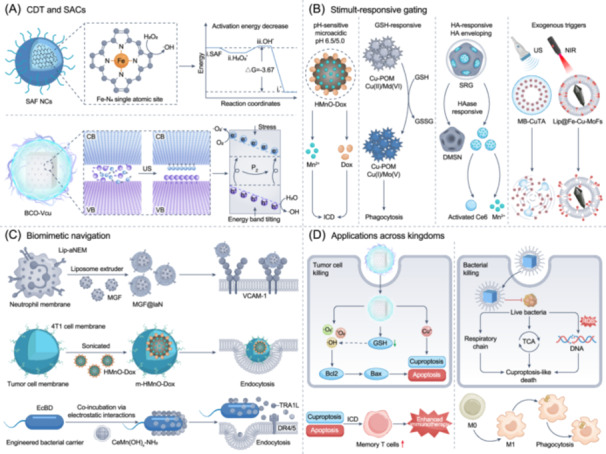
Bioresponsive nanomedicine and spatiotemporal delivery. (A) Chemodynamic therapy and single‐atom catalysts. Single‐atom Fe nanocatalysts (SAF NCs) featuring Fe‐N_4_ coordination sites reduce the activation energy barrier for hydroxyl radical generation [132]. BCO‐VCu nanoparticles utilize ultrasound‐induced energy‐band tilting to promote electron‐hole separation, generating reactive oxygen species (ROS) [535]. (B) Stimuli‐responsive gating. Microenvironmental acidosis (pH 6.5/5.0) triggers HMnO‐Dox disassembly to release Mn^2+^ and doxorubicin, inducing ICD [373]. GSH reduces Cu‐POM, depleting antioxidant pools. Hyaluronidase degrades hyaluronic acid envelopes [368], whereas exogenous ultrasound [235] and near‐infrared irradiation spatiotemporally trigger nanocarrier disassembly. (C) Biomimetic navigation. Hybrid neutrophil membrane‐camouflaged nanodecoys (MGF@laN) target VCAM‐1. 4T1 cancer cell membranes facilitate specific endocytosis of m‐HMnO‐Dox [373]. Engineered bacterial carriers (EcBD) co‐incubated with CeMn(OH)_x_‐NH_2_ present TRAIL to activate DR4/5 receptors [536]. (D) Applications across kingdoms. In eukaryotic tumor cells, ROS generation and Cu^+^ accumulation synergistically deplete GSH and modulate Bcl‐2/Bax pathways, executing concurrent apoptosis and cuproptosis to elicit ICD and expand memory T cells [250, 411]. In prokaryotes, metal overload suppresses the tricarboxylic acid cycle and respiratory chain, while ROS damage DNA, inducing cuproptosis‐like bacterial death and repolarizing macrophages from M0 to M1 phenotypes for enhanced phagocytosis.

These catalytic amplification strategies utilizing single‐atom catalysts and nanozymes are illustrated in Figure 12A. Fe‐SACs maximize metal utilization by isolating Fe atoms as well‐defined catalytic centers [532, 533]. Huo et al. reported single‐atom Fe nanocatalysts (SAF NCs) containing isolated Fe‐N_4_ coordination sites. Density functional theory analysis demonstrated that this configuration reduces the activation barrier for H_2_O_2_ dissociation, yielding *ΔG* =–3.67 eV under acidic conditions. Importantly, catalytic activity is attenuated at neutral pH, limiting Fenton reactivity in normal tissues and confining ROS generation to acidic tumor niches [132]. Extending this approach to manganese, Mn‐N/C SACs exhibit superior POD‐like activity relative to bulk MnO_2_ [534]. These catalysts promote H_2_O_2_ decomposition with a low activation barrier of 0.16 eV, generating hydroxyl radicals (·OH) that induce cytotoxicity and promote ICD, in part through Mn^2+^‐mediated activation of the cGAS‐STING pathway [367].

Copper‐based SACs have been designed to exploit metabolic liabilities in cancer cells. Cu‐N/C systems emulate NOX activity by concurrently producing ROS and depleting intracellular NADPH, thereby enforcing redox collapse. This combined mechanism promotes hybrid ferroptotic and disulfidptotic signaling, overcoming resistance in tumors with elevated antioxidant capacity [273]. Catalyst performance can be further enhanced through ligand engineering; coordination of single copper atoms with conjugated ligands, as in Cu‐DBCO, decreases the desorption energy barrier for ·OH to 0.91 eV, accelerating ROS turnover and facilitating cuproptosis [537]. More recently, heterotrimetallic platforms incorporating Cu, Co, and Fe within a unified catalytic center have been developed. Adjustment of the d‐band center to −1.64 eV optimizes electron transfer dynamics and enhances catalytic flux beyond that achievable with mono‐ or bimetallic systems, thereby amplifying oxidative stress within the TME [538].

In parallel with SAC innovation, defect engineering and external‐stimulus activation have emerged as complementary strategies to improve nanozyme catalytic efficiency. Introduction of copper vacancies (V_Cu) in Bi_2_CuO_4_ nanoparticles generates hole‐trapping sites that suppress electron‐hole recombination and enhance ROS output under ultrasound stimulation, integrating sonodynamic activation with metal‐dependent cytotoxicity [535] (Figure 12A). Similarly, copper‐doped ZnAl layered double hydroxides (LDHs) undergo Jahn‐Teller distortion, modifying electronic structure to favor sonodynamic ROS production [539]. Z‐scheme heterojunctions, including Cu_2_O‐CoWO_4_ [540] and GQD/Cu_2_O composites [232], promote spatial charge separation and sustained regeneration of Cu^+^ species. Notably, copper exhibits a Fenton‐like reaction rate constant of *k* = 1 × 10^4 ^M^−1^ s^−1^, substantially exceeding that of iron‐catalyzed reactions, thereby supporting rapid and potent oxidative damage [541]. Distinct from DNA‐targeting platinum chemotherapeutics, ultrasmall Pt nanoclusters (*L*‐Asp‐Pt) act as highly efficient peroxidase mimics. These nanoclusters generate oxidative flux approximately 30‐fold greater than conventional ferroptosis inducers within minutes, producing rapid necrotic injury that can bypass established resistance pathways [542].

### Stimuli‐responsive nanomedicines and spatiotemporally controlled delivery

#### Endogenous microenvironmental gating

To safely translate the potent catalytic activity of SACs and nanozymes, delivery systems must address therapeutic index constraints by restricting lethal metal accumulation to malignant tissues while minimizing off‐target toxicity. This selectivity is achieved through stimuli‐responsive gating strategies that exploit defining physicochemical features of the TME, including acidosis, redox imbalance, and aberrant enzyme activity, or through externally applied physical triggers. These microenvironmental and exogenous gating mechanisms are comprehensively illustrated in Figure 12B. Among endogenous cues, the pH differential between normal tissues (pH 7.4) and acidic tumor interstitium (pH 6.5) or lysosomal compartments (pH 5.0) is widely used to control release kinetics [543, 544]. Metal‐organic frameworks, particularly zeolitic imidazolate framework‐8 (ZIF‐8), function as acid‐labile zinc reservoirs that remain stable in circulation yet rapidly degrade within acidic endosomal environments, releasing Zn^2+^ to induce mitochondrial dysfunction and zincoptosis [346, 545, 546]. Similarly, MnO_2_ coatings act as pH‐sensitive gatekeepers; dissolution under acidic conditions liberates therapeutic cargo and generates oxygen, alleviating hypoxia and restoring sensitivity to ROS‐dependent modalities [363, 547, 548]. Carbonate‐based systems, including CuMnCO_3_ nanoplatforms, exploit acidic microenvironments to trigger localized ion release within osteoclastic lacunae or tumor tissue [372, 549]. Recent advances have further engineered pH‐responsive liquid nanoparticles and programmable Lipiodol Pickering emulsions that undergo rapid structural degradation in the mildly acidic TME to precisely liberate catalytic metal ions (e.g., Fe^2+^), driving intense Fenton‐like reactions and initiating robust lipid peroxidation for transarterial ferro‐embolization [550, 551]. Moreover, biomimetic Cu‐based nanoplatforms, Cu‐based heterojunctions, and metal‐organic frameworks (MOFs) have been designed to leverage acidity‐triggered metal release, effectively synergizing CDT with cuproptosis [552, 553]. Similarly, cobalt‐based MOFs and hypoxia‐responsive Co(III) prodrugs exploit acidic, GSH‐rich microenvironments to unleash targeted oxidative lethality, overcoming tumor chemoresistance with high spatiotemporal precision [404, 554].

Redox dysregulation within tumors, characterized by elevated intracellular GSH levels (2‐10 mM), provides an additional logic gate for selective activation. Nanocarriers incorporating disulfide‐linked organosilica frameworks [225, 555] or copper‐coordination polymers [253, 556] are engineered to undergo structural disassembly in high‐GSH conditions (Figure 12B). This design establishes a self‐reinforcing loop: carrier degradation releases catalytic metal ions to initiate oxidative stress while simultaneously consuming GSH and converting it to GSSG, thereby diminishing antioxidant buffering capacity [439, 557]. The resulting depletion lowers the threshold for ferroptosis and cuproptosis, enhancing susceptibility to the delivered payload [267, 417, 514]. Advanced nanoplatforms, such as copper‐based coordination polymers and flavopiridol‐copper complexes, actively exploit this redox vulnerability to selectively release Cu^+^/Cu^2+^ while concurrently exhausting endogenous GSH pools [253, 558]. Crucially, this GSH‐depleting mechanism not only downregulates GPX4 to augment lipid peroxidation but also promotes the aberrant oligomerization of lipoylated TCA cycle enzymes, profoundly sensitizing tumor cells to concurrent ferroptosis and cuproptosis [559, 560]. Furthermore, to navigate and overcome adaptive therapy resistance, cascade‐activated DNA nanoplatforms have been engineered to sequentially respond to GSH and overexpressed TME enzymes (e.g., APE1) for precise spatiotemporal payload release, initiating enzyme‐mediated adaptive ferroptosis [561]. Enzyme‐responsive architectures further refine specificity. Enzyme‐ and ROS‐responsive designs, such as HAase‐triggered size switching [562], MMP‐2/9‐cleavable motifs, and thioketal linkages [563], enable TME‐conditioned nanoplatform disassembly, localized drug/catalyst release, MMP‐activated iron‐catalyzed Fenton reactions [564], and ROS‐amplified feed‐forward activation [565].

#### Exogenous physical triggers

External stimuli provide spatiotemporal control, allowing modulation of catalytic activity within defined tissue volumes. Ultrasound can induce inertial cavitation to disrupt stromal barriers [566] or activate piezoelectric nanomaterials that promote electron‐hole separation, or trigger metal nanosonosensitizers to generate ROS in deep‐seated tumors [228, 567, 568]. Capitalizing on their superior deep‐tissue penetrability, ultrasound‐responsive nanoplatforms acting as sonosensitizers, such as Bi_2_MoO_6_‐MXene heterojunctions or reversible copper‐crosslinked nanosystems, generate massive ROS bursts to trigger potent sonodynamic therapy (SDT) and localized metal‐dependent cell death [539, 569, 570]. Near‐infrared (NIR) photothermal approaches employ phase‐transition materials, such as lauric acid, to regulate metal release upon localized heating [571] (allowing for the explosive, on‐demand release of copper ionophores to maximize cuproptosis) or to accelerate catalytic turnover rates through temperature‐dependent kinetics [572]. In particular, the application of second near‐infrared (NIR‐II) light has significantly advanced spatiotemporal precision, augmenting photothermal and photodynamic cascades to drive targeted ferroptosis and cuproptosis while strictly mitigating off‐target systemic toxicity [573, 574, 575]. Electromagnetic‐field‐responsive platforms provide externally programmable control: AMFs can trigger magnetothermal activation and ROS‐amplifying energy conversion, while electrically activable ZnS nanochips enable voltage‐controlled co‐release of Zn^2+^ and H_2_S for gas‐ionic antitumor therapy [336, 576].

#### Multistimuli and spatiotemporal immunomodulation

Building upon single‐stimulus strategies, contemporary delivery systems increasingly employ multistimuli cooperative gating to simultaneously activate interconnected metal‐dependent cell death networks. By integrating exogenous physical triggers (e.g., ultrasound, NIR light) with endogenous TME factors (e.g., GSH overexpression, acidosis, specific metabolites such as H_2_S), engineered nanoplatforms can execute highly synergistic combination therapeutics with unprecedented precision and biosafety [281, 577, 578]. For instance, carrier‐free bimetallic (Fe/Cu) MOFs, cell‐membrane‐camouflaged biomimetic copper nitride nanoparticles, and GSH‐responsive Cu‐Ce6 nanoplatforms have been strategically designed to concurrently precipitate cuproptosis and ferroptosis [579, 580]. In these dual‐action systems, external irradiation or acoustic stimulation catalyzes massive ROS storms via photodynamic or sonodynamic effects, whereas the TME‐triggered release of Cu^+^ and Fe^2+^ directly drives mitochondrial proteotoxic stress and lethal lipid peroxidation, culminating in an irreversible collapse of the cellular redox defense infrastructure [283, 581, 582].

Crucially, the spatiotemporally controlled execution of multimodal metal‐dependent cell death by these advanced delivery systems transcends localized cytotoxicity by profoundly remodeling the immunosuppressive TME. The severe oxidative bursts and organelle dysfunction inherent to nanoparticle‐induced cuproptosis and ferroptosis robustly trigger ICD, leading to the massive emission of damage‐associated molecular patterns (DAMPs) [583, 584, 585]. This immunogenic efflux promotes dendritic cell maturation, repolarizes immunosuppressive M2 macrophages toward an anti‐tumor M1 phenotype, and significantly enhances the infiltration of cytotoxic CD8^+^ T lymphocytes. When synergized with immune checkpoint blockade (e.g., anti‐PD‐L1 therapy) or specific metabolic inhibitors, these stimuli‐responsive metallotherapeutics eradicate primary lesions, establish durable immunological memory, and exert potent abscopal effects to suppress distant metastases, providing a transformative blueprint for precision metalloimmunotherapy [134, 228, 586].

### Biomimetic navigation and biological carriers

#### Cell membrane camouflage

Although stimuli‐responsive gating provides conditional TME activation, rapid systemic immune clearance frequently hinders clinical translation. To circumvent this, biomimetic surface engineering and biological carriers cloak synthetic metallotherapeutics within endogenous materials [587, 588]. This biomimetic navigation paradigm profoundly improves pharmacokinetic profiles, achieves exceptional tumor‐targeting precision, and circumvents the systemic toxicity associated with traditional metal ionophores. These diverse biomimetic navigation strategies using cellular camouflage are detailed in Figure 12C.

Cloaking nanoparticles with tumor cell membranes leverages homotypic adhesion molecules, including cadherins, to promote preferential accumulation within the originating tumor [133, 252, 373]. This homologous targeting strategy has been extensively adapted for the selective delivery of diverse metallotherapeutics. For example, encapsulating hydrogenated manganese oxide loaded with doxorubicin (HMnO‐Dox) within 4T1 cancer cell membranes (yielding m‐HMnO‐Dox) facilitates specific endocytosis into homologous tumor cells, directly linking metal‐catalyzed oxidative bursts to robust cGAS‐STING activation [373]. Similarly, tumor cell membrane camouflage efficiently transports copper‐based nanomaterials to provoke severe intracellular copper overload, thereby triggering robust cuproptosis and ICD [574]. Furthermore, encapsulating iron‐based MOFs or manganese dioxide nanozymes within homologous cancer cell membranes significantly prolongs their systemic circulation and enhances the targeted execution of Fenton‐like and peroxidase‐like reactions, amplifying ferroptosis and CDT without exerting off‐target toxicity [589, 590].

For traversal of biological barriers, immune cell membrane coatings confer functional advantages [591]. Live neutrophils enable chemotactic homing and facilitate active infiltration into tumor tissues in breast cancer models [280], and their biomimetic camouflage has recently been utilized to navigate iron‐based nanozymes into neuroinflammatory lesions to inhibit neuronal ferroptosis [592]. Specifically, hybrid pH‐responsive neutrophil membrane‐camouflaged Ga‐Mn bimetallic nanodecoys (MGF@laN) target VCAM‐1‐overexpressing tumor vascular endothelial cells, triggering a triad of apoptosis, immunity, and metastasis suppression [593], whereas macrophage membranes enhance targeting of metastatic lesions through VCAM‐1‐integrin interactions [594, 595]. These macrophage membranes are increasingly employed to direct copper complexes or manganese dioxide nanozymes, reshaping the immunosuppressive microenvironment via targeted cuproptosis or catalytic immunotherapy [596, 597]. Platelet‐derived membranes exploit P‐selectin binding to CD44 [129, 250], precisely delivering copper‐ or iron‐doped nanoparticles to highly metastatic tumors to incite metal‐induced cell death [129, 598], and natural killer cell membranes utilize NKG2D‐mediated recognition to improve specificity [599]. Additionally, erythrocyte membrane cloaking efficiently prolongs the circulation half‐life of platinum and manganese nanoagonists, amplifying ferroptosis and radiosensitization while minimizing systemic clearance [600, 601]. Furthermore, advanced hybrid membrane cloaking, which fuses tumor cell membranes with immune cell or erythrocyte membranes, endows metallotherapeutics with dual‐targeting specificity and robust immune‐evasion capabilities to amplify metal‐induced immunotherapy [602, 603, 604].

#### Extracellular vesicles and protein carriers

Beyond cell membrane cloaking, extracellular vesicles and exosomes represent highly efficient biological carriers for targeted metal delivery. Inheriting the intercellular communication abilities of their parent cells, these naturally secreted vesicles exhibit exceptional biocompatibility and deep tissue penetration. Autologous tumor‐derived exosomes can act as biomimetic delivery vehicles that encapsulate zero‐valent iron or manganese ions, selectively homing back to homologous tumor cells to unleash targeted ferroptosis and provoke STING‐pathway‐mediated innate immunity [605, 606]. Macrophage‐derived exosomes have similarly proven effective in crossing biological barriers to deliver metal therapeutic agents. Modifying M2 macrophage exosomes with ultrafine copper sulfide nanoparticles actively targets activated T cells to induce cuproptosis, establishing antigen‐specific immune tolerance in rheumatoid arthritis (RA) [257]. In oncology, macrophage exosomes loaded with Fe_3_O_4_ nanoparticles overcome dense desmoplastic stroma, triggering massive immunogenic cell death and reprogramming the immunosuppressive TME [607]. Likewise, ultrasound‐activated sonophages spatiotemporally deliver saturated brine into tumors, synergizing sonodynamic oxidative stress with catastrophic osmotic collapse to execute robust immunogenic saltoptosis [321].

Protein‐based carriers such as H‐ferritin [608, 609] and albumin [610] offer biocompatible, low‐immunogenic delivery platforms that exploit endogenous receptor‐mediated transport, including TfR1‐dependent HFn uptake and gp60/caveolae‐mediated albumin transcytosis [611, 612]. Ferritin, characterized by its unique nanocage architecture, demonstrates an exceptional natural affinity for encapsulating transition metal cations. By leveraging the significant overexpression of transferrin receptor 1 (CD71) on specific target cells, ferritin nanocages have been rationally engineered to navigate iron‐polyphenol complexes directly into acute myeloid leukemia cells, inciting a robust ferroptotic cascade [613]. Similarly, HFn carriers have been utilized to co‐encapsulate photodynamic agents and actively transport them into hypertrophic scar fibroblasts, establishing a localized ferroptosis amplifier that capitalizes on labile iron pool expansion [614]. In addition, these protein cages are readily adapted to load therapeutic metal radioisotopes (e.g., ^64^Cu), highlighting their immense potential in precision nanoparticulate theranostics [615].

#### Engineered living vectors and microbial vesicles

Emerging platforms employ engineered bacteria or outer membrane vesicles to access hypoxic tumor cores, delivering copper and other metallotherapeutics directly to metabolically constrained regions and inducing localized mitochondrial collapse [488, 536, 616]. For example, as illustrated in Figure 12C, engineered bacterial carriers (EcBD) co‐incubated via electrostatic interactions with metal‐based nanoparticles (e.g., CeMn(OH)_x_‐NH_2_) can present targeted ligands like mTRAIL to engage tumor death receptors (DR4/5) and enhance targeted apoptosis [536]. Living engineered bacteria, such as attenuated *Salmonella* or *Escherichia coli*, naturally exhibit robust hypoxia tropism, allowing them to actively penetrate deep into solid tumors. Bacteria–nanomaterial biohybrids can integrate tumor tropism with externally controlled metal‐dependent therapy: magnetic Fe‐based systems enhance ferroptosis‐immunotherapy, while CuS‐engineered bacteria enable NIR‐triggered CDA release, STING activation, and cuproptosis‐amplified systemic immunity [617, 618, 619]. In parallel, bacterially derived outer membrane vesicles (OMVs) preserve the immunogenic properties of their parent strains while avoiding live‐infection risks. Recent studies have utilized OMVs to encapsulate copper ionophores [488], gold‐iron nanozymes [620], ferrocene [621], or rhenium‐palladium bimetallic complexes [622]. By integrating metal‐induced severe oxidative stress with the inherent pathogen‐associated molecular patterns (PAMPs) of OMVs, these biomimetic immune organelles function as potent immune adjuvants, profoundly activating dendritic cells and orchestrating robust systemic anti‐tumor T cell responses. Finally, probiotic‐based systems, such as anaerobic bacteria synthesizing selenium nanoradiosensitizers *in situ*, further underscore the vast potential of these biological carriers in anchoring metallotherapeutics selectively to tumors [623].

### Targeting bacterial metal dyshomeostasis

Extending the metallolethality paradigm beyond eukaryotic nanomedicine, spatiotemporally controlled platforms are being deployed against the immunosuppressive tumor microbiome and multidrug‐resistant infections. By exploiting shared vulnerabilities within the TME or infection microenvironment (IME), this cross‐kingdom strategy bypasses classical antibiotic resistance to profoundly remodel pathological niches.

Recent studies have characterized prokaryotic cuproptosis in bacteria, including MRSA, *E. coli*, and *P. aeruginosa*, recapitulating lipoylated protein aggregation observed in mammalian systems. Distinct from nonspecific oxidative injury, this modality selectively disrupts central carbon metabolism. Multi‐omics profiling demonstrates that copper overload downregulates core TCA cycle genes, including citrate synthase, succinate dehydrogenase subunits (*sdhA/B*), and pyruvate dehydrogenase (*pdhA/B*), resulting in pyruvate accumulation and ATP depletion [260, 528, 624] (Figure 12D). This metabolic failure is commonly accompanied by inhibition of Respiratory Chain Complexes I and II and perturbation of the glyoxylate shunt, generating a lethal signature defined by lipid peroxide accumulation and membrane depolarization [528, 625].

Effective induction of lethal metal overload requires circumvention of bacterial efflux pumps, including CopB and CopZ, and penetration of the biofilm matrix. Advanced nanomedicine platforms exploit physicochemical characteristics of the infection microenvironment (IME) to achieve localized activation. By leveraging the acidic pH of infected tissue (~5.5‐6.0), systems such as Fe3O4@CuTA ionic microbubbles and CuCo_2_O_4_ nanoflowers enable pH‐triggered ion release, accelerating the Cu^2+^ to Cu^+^ valence transition selectively at infection sites while remaining stable at physiological pH [624]. Targeting specificity is reinforced through ligand functionalization; copper‐gallic acid nanoneedles are mediated by the amidation reaction between the carboxyl groups of gallic acid and the teichoic acid on gram‐positive cell walls on gram‐positive cell walls, promoting selective adhesion and penetration [528]. Furthermore, low‐intensity ultrasound disrupts biofilm architecture via inertial cavitation, facilitating deeper ion penetration [566], whereas mild photothermal stimulation induces a transient hypermetabolic state that upregulates bacterial copper transporters, exacerbating dysregulated copper uptake [625].

Sustained bacterial eradication requires amplification of metal toxicity beyond the buffering capacity of intrinsic antioxidant systems, primarily GSH. Current designs implement dual‐mechanism strategies integrating CDT with targeted metabolic interference. Bimetallic oxides and SACs catalyze the conversion of endogenous H_2_O_2_ to hydroxyl radicals through Fenton‐like reactions [528, 624]. Concurrently, catalytic amplifiers are incorporated to deplete intracellular GSH reserves. Pharmacologic inhibition of GSH biosynthesis using BSO establishes synthetic lethality, markedly enhancing copper‐mediated cytotoxicity [260]. Small‐molecule ionophores, including elesclomol and pyrithione, further facilitate metal translocation across bacterial membranes against concentration gradients, collapsing membrane potential and respiratory function where free metal ions alone are neutralized by efflux systems [25, 271].

Beyond copper, antibacterial metallotherapeutic strategies now encompass zinc, manganese, and modulation of host iron homeostasis. Zincoptosis‐like antibacterial activity has been demonstrated using PYT@ZIF8@siRNA nanoparticles, which co‐deliver pyrithione and SLC30A1 siRNA to amplify zinc dyshomeostasis and eradicate intratumoral *P. gingivalis* through membrane disruption and ROS accumulation [25]. Similarly, zinc‐manganese sulfide nanoparticles impair redox balance and biofilm stability in MRSA by releasing H_2_S to suppress respiratory activity [626]. In parallel, host‐directed modulation of iron metabolism provides a complementary approach; inhibition of macrophage ferroptosis using iron chelators limits necrotic cell death and reduces intracellular pathogen burden, including *Mycobacterium tuberculosis* [627]. Crucially, metal‐mediated bacterial killing exhibits intrinsic immunogenicity. ROS‐driven bacterial lysis promotes antigen release and repolarizes macrophages from the tolerance‐associated M2 phenotype toward the bactericidal M1 state [260, 566] (Figure 12D). Ultimately, this metalloimmunotherapeutic interface links infection control with tumor immunity, as the targeted elimination of intratumoral bacteria through metal overload alleviates immunosuppressive features of the TME and potentiates immune checkpoint blockade [25].

### Section summary

Transitioning from the systemic toxicity of conventional small‐molecule metallotherapeutics to spatiotemporally controllable nanomedicine represents a critical advancement in metal‐dependent cell death. SACs and nanozymes bypass traditional stoichiometric limitations by establishing localized catalytic environments [132, 628]. However, to overcome microenvironmental constraints, such as insufficient endogenous H_2_O_2_ and excessive GSH buffering [629] as well as complex manufacturing (Chemistry, Manufacturing, and Controls [CMC]) hurdles, next‐generation platforms increasingly integrate stimuli‐responsive logic gating and biomimetic camouflage [129, 540], encompassing versatile biological carriers like extracellular vesicles, protein carriers, and engineered living vectors. By synergizing these elements, these architectures achieve precise spatiotemporal release to concurrently activate interconnected cell death networks and potentiate precision metalloimmunotherapy. Ultimately, expanding this targeted delivery framework across kingdoms provides a transformative strategy to eradicate the tumor microbiome and multidrug‐resistant infections, bridging localized metallotoxicity with systemic immune remodeling [528, 625].

## SYSTEMIC IMMUNE REMODELING IN METALLOIMMUNOTHERAPY

### Overview

This chapter establishes a conceptual reorientation in metalloimmunotherapy, framing metal‐dependent RCD as a dual‐edged systemic cascade that not only restructures the immunosuppressive TME to sustain adaptive immunity but also drives pathogenic sterile inflammation in non‐neoplastic diseases. ICD is a key driver of this remodeling, as metal‐dependent lethal programs—particularly ferroptosis and cuproptosis—can promote DAMP release and thereby amplify antitumor immunity [229, 630, 631]. Bridging this localized damage with systemic innate immunity is the dual function of intracellular manganese. Manganese provokes cytosolic leakage of mitochondrial and nuclear DNA, alongside astrocytic double‐stranded RNA (dsRNA), while concurrently serving as a direct physiological agonist of the cGAS‐STING pathway, yielding robust IFN‐I production [37, 59]. Finally, the chapter elucidates the mechanistic convergence of these innate responses with immune checkpoint blockade (ICB). Crucially, we extend this paradigm beyond oncology to explore how metal‐driven DAMP emission and innate immune hyperactivation precipitate IRI, autoimmune disorders, and neurodegeneration, underscoring the need to therapeutically balance antitumoral memory with the restoration of systemic tolerance through spatiotemporally precise, bidirectional interventions.

### Immunogenic cell death and sterile inflammation

#### Antitumor immunogenic cell death

Distinct from uncontrolled necrosis and immunologically silent apoptosis, metal‐dependent RCD is intrinsically pro‐inflammatory and capable of initiating antigen‐specific adaptive immunity in oncological contexts, while concurrently driving pathogenic sterile (aseptic) inflammation in non‐neoplastic diseases [632, 633, 634]. Metal dysregulation induces ICD through defined stress‐responsive pathways that govern the spatiotemporal emission of DAMPs. Central to this immunogenic conversion is a catastrophic oxidative surge generated by Fenton and Fenton‐like reactions. Experimental data demonstrate that copper‐based nanoplatforms, including ECPCP and CRIM, catalyze H_2_O_2_ conversion into highly reactive hydroxyl radicals while concurrently exhausting intracellular GSH reserves [567, 635]. This redox collapse provokes ER stress and enforces calreticulin (CRT) translocation to the plasma membrane, where it functions as a dominant pro‐phagocytic signal [636, 637]. Simultaneously, membrane destabilization, an active process orchestrated by the cell swelling‐induced oligomerization of pore‐forming proteins such as NINJ1 [638], permits the extracellular release of high mobility group box 1 (HMGB1) and ATP, which serve as inflammatory mediators and chemoattractants, respectively [556, 639]. The catalytic efficiency of metal platforms is highlighted by findings that single‐atom manganese nanozymes and iron‐based catalysts elicit CRT exposure and HMGB1 liberation at levels substantially exceeding noncatalytic controls, supporting the conclusion that metal‐amplified oxidative stress represents a potent initiator of ICD [129, 367, 640].

In addition to generalized oxidative injury, discrete metal‐driven death programs recruit distinct molecular mechanisms to amplify immunogenic output. Cuproptosis, defined by copper‐induced aggregation of lipoylated TCA cycle proteins, constitutes a particularly robust ICD trigger. Oligomerization of lipoylated proteins such as DLAT provokes intense proteotoxic stress and pronounced ATP depletion. This bioenergetic crisis triggers AMPK‐mediated phosphorylation and subsequent extracellular release of HMGB1 [641], concomitantly producing intense proteotoxic stress that enhances tumor antigen availability [284]. In vitro analyses report that cuproptosis‐driven ICD significantly enhances dendritic cell (DC) maturation, suggesting that aggregate‐associated antigen processing may facilitate presentation efficiency [222, 234, 267]. By comparison, ferroptosis derives immunogenic capacity from iron‐dependent lipid peroxidation. Progressive accumulation of lipid hydroperoxides compromises membrane integrity and promotes DAMP release [642]. However, ferroptotic immunogenicity exhibits temporal sensitivity; fully lytic ferroptosis generates stimulatory cues, whereas pre‐lytic states may emit lipid‐derived ROS that suppress DC maturation, underscoring the importance of kinetic regulation in therapeutic design [643, 644].

Additional ion‐driven modalities broaden the spectrum of immunogenic activation. During calcicoptosis, sustained mitochondrial calcium overload provokes organelle rupture and bioenergetic collapse, culminating in necrotic membrane permeabilization that recruits dendritic cells [21, 645, 646]. Zinc dysregulation similarly engages inflammatory execution pathways, including GSDMD‐dependent pyroptosis or paraptosis, establishing a self‐amplifying loop of cytokine release and antigen dissemination distinct from canonical mitochondrial damage [334, 349, 647]. Moreover, targeted sodium nanocatalysts bypass canonical channels to provoke catastrophic osmotic swelling and gasdermin‐dependent pyroptosis, triggering massive DAMP emission to profoundly immunize the TME [97, 323]. To potentiate systemic immunity, contemporary strategies increasingly harness PANoptosis or hybridized death programs. For example, biomimetic cobalt‐vanadium oxide (CoVO_x_) nanocakes have been engineered to initiate convergent ROS‐metabolic signaling that activates the PANoptosome, producing a hyperinflammatory response characterized by IL‐1β secretion and LDH release [398]. Such coordinated induction of multiple lethal subroutines reprograms immunologically quiescent tumors into immune‐reactive niches, reinforcing the principle that effective metalloimmunotherapy depends on integration of mechanistically distinct yet immunologically convergent cell death pathways [47, 398].

#### DAMP‐mediated sterile inflammation and autoimmunity

Beyond the scope of tumor immunology, the spatiotemporal emission of DAMPs following metal‐dependent RCD serves as a primary catalyst for pathogenic sterile inflammation in non‐neoplastic diseases, particularly IRI and acute organ failure [632, 648]. In these conditions, catastrophic oxidative bursts and unremitting metal dyshomeostasis precipitate the massive extracellular leakage of specific danger signals, including HMGB1, ATP, mtDNA, and decorin [633, 648, 649, 650]. For instance, in myocardial, renal, and spinal cord IRI, ferroptotic or oxidatively injured cells provide HMGB1‐rich DAMP signals that amplify local sterile inflammation, with the advanced glycosylation end‐product specific receptor (AGER/RAGE) mediating macrophage responses to ferroptotic cells and HMGB1–Toll‐like receptor 4 (TLR4) signaling driving broader IRI‐associated inflammatory cascades [631, 651, 652, 653]. Metabolic–epigenetic crosstalk may further amplify HMGB1‐driven DAMP signaling in IRI, as shown by PKM2/p300‐dependent H4K12 lactylation at the HMGB1 promoter in intestinal ischemia/reperfusion (I/R) and by HMGB1 translocation/release and lactylation‐linked injury in renal I/R [654, 655, 656]. Similarly, in acute organ failure models, such as postmyocardial infarction heart failure, SLC31A1‐mediated copper overload provokes macrophage cuproptosis, unleashing HMGB1 to hyperactivate the NLRP3 inflammasome [657]. Furthermore, iron‐induced mitochondrial lipid peroxidation uniquely promotes the leakage of mtDNA into the cytosol, engaging the cGAS‐STING and Caspase‐1 pathways to orchestrate macrophage‐driven paracrine inflammation [649]. Collectively, these released DAMPs establish a self‐amplifying ferroptosis‐driven inflammatory cascade that drives neutrophil recruitment and polarizes macrophages toward a pro‐inflammatory M1 phenotype, significantly exacerbating tissue damage in septic AKI and ischemic stroke [658, 659]. Consequently, neutralizing this metal‐dependent DAMP emission (either through pharmacological inhibition of the ferroptosis/HMGB1 axis with agents like glycyrrhizin, fisetin, and isoliquiritigenin, or by utilizing targeted nanoreactors and mild hypothermia) has emerged as a highly promising strategy to decouple bioenergetic collapse from pathological tissue destruction [655, 658, 660, 661].

Systemically, the consequences of this unabated, metal‐driven sterile inflammation are deeply embedded in the pathogenesis of neutrophil‐ and macrophage‐mediated autoimmune diseases [632, 633]. The chronic execution of metal‐dependent cell death furnishes a continuous supply of autoantigens and DAMPs, progressively eroding peripheral immune tolerance. In systemic lupus erythematosus (SLE), autoantibodies and interferon‐alpha cooperatively suppress GPX4 expression via the CaMKIV/CREMα axis, precipitating widespread neutrophil ferroptosis [188, 644]. The consequent release of HMGB1 and other cellular contents forms a vicious pathogenic feedback loop that persistently overstimulates autoreactive B cells and plasmacytoid dendritic cells to drive systemic autoimmunity [644]. Analogously, ferroptotic injury is linked to RA and experimental autoimmune encephalomyelitis (EAE) progression; in RA joints, focal iron overload preferentially eliminates anti‐inflammatory M2 macrophages through ferroptosis, thereby skewing the macrophage balance toward a pro‐inflammatory M1‐dominant state and amplifying synovitis [662, 663]. However, translating these mechanistic insights into systemic pharmacological interventions reveals profound biological complexity. In antineutrophil cytoplasmic antibody (ANCA)‐associated vasculitis (AAV), while targeted genetic inhibition of endothelial ferroptosis (e.g., via endothelial *ACSL4* deletion) is locally protective against necrotizing crescentic glomerulonephritis, systemic pharmacological ferroptosis blockade using canonical agents like ferrostatin‐1 paradoxically exacerbates the disease. This therapeutic failure occurs because broad‐spectrum systemic ferroptosis inhibitors inadvertently hyperactivate pro‐inflammatory functions in myeloid compartments, specifically enhancing monocyte‐mediated Th17 polarization and amplifying the release of neutrophil extracellular traps (NETs) [664]. This dichotomy underscores that while metal‐dependent RCDs represent critical therapeutic targets in sterile inflammation and autoimmunity, their successful clinical management requires exquisite spatial or cell‐type‐specific precision to safely disentangle tissue preservation from the unintentional exacerbation of systemic immune dysregulation.

### Manganese‐driven cGAS‐STING activation

#### Innate immune potentiation in oncology

Following its canonical function as a metabolic cofactor, Mn^2+^ has emerged as a potent physiological agonist of the cGAS‐stimulator of interferon genes (STING) pathway, redefining its role within metalloimmunotherapy. Therapeutic activation of this axis depends on a coordinated co‐stimulatory mechanism coupling cytotoxic injury with direct innate immune potentiation [665, 666]. The initial signal arises from the accumulation of cytosolic dsDNA ligands. Manganese‐based nanoplatforms, including Mn‐doped ZnO and MnS nanocapsules, provoke severe mitochondrial stress manifested by membrane rupture, cristae disruption, and mPTP opening [360, 667]. This structural damage enables mtDNA escape into the cytosol, where it activates cGAS [251, 269]. In parallel, manganese‐driven CDT generates hydroxyl radicals through Fenton‐like reactions that fragment nuclear DNA, thereby augmenting the cytosolic reservoir of immunostimulatory nucleic acids [367, 405].

The second signal involves direct sensitization of the innate immune sensor by intracellular Mn^2+^. In the absence of manganese, isolated DNA release frequently elicits only partial STING activation. By contrast, Mn^2+^ enhances cGAS responsiveness to cytosolic DNA and strengthens cGAMP binding affinity to STING, functioning as an essential signal amplifier [37, 626, 668]. This potentiation markedly increases phosphorylation within the downstream cascade, including p‐STING, p‐TBK1, and p‐IRF3, culminating in robust IFN‐I production [362, 595]. Experimental evidence demonstrates that STING activation increases with intracellular Mn^2+^ availability and is blunted by Mn^2+^ sequestration or pharmacologic STING inhibition [669, 670]. Moreover, combinatorial strategies integrating manganese delivery with calcium sulfide‐induced pyroptosis show that GSDME‐dependent pore formation synergistically enhances mtDNA release, intensifying pathway activation [366]. Analogously, cobalt nanocatalysts inherently trigger caspase‐1/GSDMD‐dependent pyroptosis, actively releasing mtDNA to self‐cascade and specifically hyperactivate STING‐mediated antitumor immunity [405].

To translate this mechanism while minimizing systemic neurotoxicity associated with manganese exposure, bioengineering efforts have prioritized concentration‐amplifying systems that restrict activation to the TME. High‐valence manganese nanomaterials, such as MnO_2_, function as redox‐responsive dual agents within the GSH‐rich TME. Upon cellular uptake, they oxidize GSH to GSSG, simultaneously weakening antioxidant buffering to facilitate ferroptosis and reducing to Mn^2+^, which activates immune signaling [370, 671]. Alternative acid‐sensitive platforms, including Mn‐phosphate and Mn‐doped silica, remain stable in circulation but undergo rapid degradation in lysosomal or intratumoral compartments with pH 5.5–6.5. These constructs achieve intracellular manganese concentrations approaching 40 μM, sufficient for cGAS activation, while maintaining systemic exposure within physiological limits [536, 672]. In addition, post‐operative ROS‐responsive hydrogels have been engineered to release Mn‐pectin microspheres locally, sustaining immunostimulatory levels at resection sites to reduce recurrence risk [363].

Activation of the Mn‐cGAS‐STING axis produces extensive systemic immune remodeling that converts immunosuppressed tumors into immunologically active targets. Elevated secretion of pro‐inflammatory cytokines, including IFN‐β, TNF‐α, and IL‐6, promotes dendritic cell (DC) maturation, with significant expansion of CD80^+^/CD86^+^ populations observed in tumor‐draining lymph nodes [87, 616]. Concurrently, manganese drives the repolarization of tumor‐associated macrophages (TAMs) from an M2 to an M1 phenotype, marked by upregulated inducible nitric oxide synthase (iNOS) and CD86 expression [363, 610]. These changes support infiltration and activation of cytotoxic CD8^+^ T cells and facilitate the establishment of durable immunological memory. In bilateral tumor models, localized manganese administration frequently induces regression of distant untreated lesions, consistent with an abscopal effect, and enhances responsiveness to ICB, achieving synergistic tumor inhibition exceeding 90% in selected systems [59, 362, 365, 673]. Collectively, manganese‐mediated cGAS‐STING activation exemplifies a strategy in which the metal ion functions not solely as a cytotoxic agent but as a defined immunologic adjuvant linking localized damage to systemic antitumor immunity.

#### Pathological neuroinflammation in the central nervous system

However, the potent immunomodulatory capacity of the manganese‐driven innate immune response operates as a dual physiological role when extending this paradigm beyond the TME to non‐neoplastic pathologies. Although the basal physiological tone of type I IFN signaling is essential for normal central nervous system (CNS) development and cognitive function [674], aberrant systemic manganese accumulation drives maladaptive aseptic chronic neuroinflammation, fundamentally contributing to the pathogenesis of neurodegenerative disorders, including Parkinson's disease, Alzheimer's disease, and manganism [674, 675, 676]. In the CNS, excessive manganese exposure converges on IFN‐I‐driven inflammatory cascades across key glial populations via distinct sensing mechanisms. Specifically, manganese overexposure severely impairs PINK1/Parkin‐mediated mitophagy, leading to profound microglial mitochondrial dysfunction. The subsequent cytosolic leakage of damaged mitochondrial DNA (mtDNA) robustly engages the cGAS‐STING‐IRF3 axis, unleashing a massive release of IFN‐I and neurotoxic pro‐inflammatory cytokines that precipitate Parkinsonian neurobehavioral deficits, a cascade that can be pharmacologically restrained by mitophagy activators such as urolithin A [675]. Furthermore, independent of cytosolic DNA sensing, intracellular Mn^2+^ can allosterically activate microglial cGAS directly, initiating noncanonical 2'3’‐cGAMP synthesis that exacerbates NF‐κB and IFN‐I‐dependent neuroinflammation and cognitive decline, a vulnerability susceptible to targeted pathway inhibitors like sesamol [676]. This metallo‐immune dysregulation exhibits distinct cell‐type‐specific complexities; in mature astrocytes, sub‐cytotoxic manganese disrupts the mitochondrial transcriptome, prompting the cytosolic efflux of mitochondrial dsRNA [674]. Instead of engaging cGAS, this dsRNA activates the cytosolic sensor MDA5, which converges on the TBK1/IRF3 signaling node to unleash a parallel IFN‐I transcriptional cascade [674]. Consequently, the precise molecular events that confer innate immune potentiation in oncology (mitochondrial structural damage, nucleic acid compartmentalization failure, and hyperactivated IFN‐I signaling) act as the primary instigators of chronic neurodegeneration in the CNS. This mechanistic dichotomy explicitly underscores the critical necessity for spatiotemporally restricted bioresponsive delivery architectures and targeted immunomodulators in the clinical translation of metalloimmunotherapeutics.

### Synergizing ICB and modulating systemic autoimmunity

#### Reversing tumor immunosuppression and ICB resistance

Clinical implementation of ICB, particularly targeting the PD‐1/PD‐L1 axis, is frequently constrained by the immunologically quiescent character of the TME, marked by limited T‐cell infiltration and a predominance of suppressive myeloid populations [677, 678, 679]. Metalloimmunotherapy has emerged as a formidable strategy that not only exerts direct cytotoxicity but also reshapes the immune milieu to enhance checkpoint responsiveness. Effective ICB requires the conversion of these non‐inflamed tumors into immune‐active tissues, a transformation mediated by ferroptosis through the recruitment of effector lymphocytes and the reprogramming of innate immune compartments [680].

A principal driver of this synergy is the reversal of immunosuppression via the repolarization of TAMs [681]. The induction of ferroptosis preferentially eliminates immunosuppressive M2 macrophages while preserving pro‐inflammatory M1 subsets, reflecting the absence of an adequate iNOS/NO· buffering system in M2 cells necessary to counter‐lipid peroxidation [682]. As a result, metal‐based platforms, including manganese‐ferrite nanohybrids and zinc–nickel hydroxide nanosheets, consistently shift the TME toward an M1‐dominant phenotype [334, 595, 683]. This myeloid reconfiguration is accompanied by the depletion of regulatory T cells (Tregs) and the enhanced infiltration of CD8^+^ cytotoxic T cells [684]. For example, copper‐doped polydopamine nanoparticles inducing combined ferroptosis and cuproptosis, increase CD8^+^ T‐cell infiltration by approximately 20% in lung tumor models, expanding the therapeutic impact of anti‐PD‐L1 therapy fivefold [267]. Likewise, copper‐based nanoplatforms that trigger cuproptosis elevate CD8^+^ T‐cell frequencies to 75.7% relative to 44.8% in control conditions, thereby establishing a permissive context for checkpoint blockade [255, 685].

Metal‐dependent signaling intersects dynamically with the PD‐1/PD‐L1 axis through dual mechanisms involving target upregulation and resistance reversal. In certain contexts, metal‐induced stress creates a conditional dependence on PD‐1 signaling. Copper ionophores and manganese nanozymes markedly increase *PD‐L1* expression via activation of the cGAS‐STING‐IFN‐I pathway and metabolic stress responses [367, 686, 687]. Although *PD‐L1* induction may be immunosuppressive in isolation, its enhanced surface density provides increased binding targets for anti‐PD‐L1 antibodies, sensitizing tumors previously refractory to therapy. Conversely, alternative metallo‐therapeutic designs promote PD‐L1 degradation to overcome resistance. Manganese‐mediated AMPK activation accelerates the lysosomal turnover of PD‐L1, whereas copper‐organic frameworks engineered to modulate m6A RNA methylation destabilize PD‐L1 transcripts [688]. In addition, copper‐based nanozymes combined with cholesterol oxidase reduce PD‐1 and TIM‐3 expression on T cells by alleviating ER stress, restoring effector function even without exogenous antibody administration [537].

This cooperative interaction is reinforced by reciprocal feedback between ferroptosis and adaptive immunity. Following ICB, activated CD8^+^ T cells secrete IFN‐γ, which transcriptionally represses system x_c_^−^ subunits SLC3A2 and SLC7A11, depleting intracellular GSH and heightening susceptibility to lipid peroxidation [103]. Clinical data sets from melanoma cohorts support this mechanism, identifying ACSL4 expression as a positive correlate of anti‐PD‐1 responsiveness [69]. Tumors may acquire resistance through the upregulation of TYRO3, which suppresses ferroptosis via AKT/NRF2 signaling; inhibition of this pathway reinstates T‐cell‐mediated lethality [203]. The PKCβII–ACSL4 axis further amplifies this loop by intensifying lipid remodeling and oxidative damage, thereby strengthening PD‐1 blockade efficacy [69]. The role of IFN‐γ in linking antitumor immunity with ferroptosis‐mediated therapeutic synergy has been comprehensively discussed [103, 689].

Systemic immune activation culminating in an abscopal response represents the strongest validation of this synergy. The local induction of metal‐dependent RCD repeatedly suppresses the growth of distant untreated metastases in bilateral tumor models [690, 691]. Copper‐selenium frameworks administered with anti‐PD‐L1 significantly inhibit the progression of contralateral tumors [228], while electrically activatable zinc‐sulfide nanochips combined with anti‐CTLA‐4 eradicate both primary and secondary lesions [336]. These outcomes are sustained by the development of long‐term immunological memory, as treated subjects frequently reject tumor rechallenge due to the expansion of central memory T‐cell populations [250, 692].

#### Managing adverse events and restoring systemic tolerance

Notwithstanding these advances, the application of metalloimmunotherapy demands contextual precision because metal‐induced inflammation may not universally confer therapeutic benefit. In glioblastoma, neutrophil‐associated ferroptosis correlates with necrotic progression rather than protective immunity, indicating potential pro‐tumorigenic consequences in specific microenvironments [693]. Moreover, ferroptotic cancer cells can impose an oxidized lipid burden on dendritic cells, promoting lipid‐droplet accumulation and thereby compromising antigen cross‐presentation [643, 694, 695]. Furthermore, the unconstrained systemic immune hyperactivation elicited by ICB therapy can inadvertently precipitate severe immune‐related adverse events. For instance, localized T‐cell overactivation coupled with aberrant iron‐dependent ferroptosis in nontarget organs can drive profound reproductive toxicity, highlighting the delicate threshold between therapeutic efficacy and immune‐mediated pathology [696]. These observations underscore the necessity for precise modulation of the TME, potentially integrating metal ionophores with STING agonists or metabolic inhibitors, to ensure that metal‐dependent cytotoxicity culminates in durable therapeutic immunity rather than chronic inflammatory pathology or unintended autoimmune toxicity.

Beyond the neoplastic context, the pleiotropic interplay between metal‐driven immune activation and regulated cell death serves as a fundamental pathogenic driver in nontumor systemic autoimmune diseases, including RA, SLE, and multiple sclerosis (MS) [662, 697]. Within the hyperplastic RA synovium, focal iron overload profoundly disrupts macrophage homeostasis by unmasking a heterogeneous susceptibility to lipid peroxidation. Specifically, anti‐inflammatory M2 macrophages are exquisitely vulnerable to iron‐induced ferroptosis following autophagic GPX4 degradation, whereas pro‐inflammatory M1 macrophages demonstrate robust resistance [663]. The subsequent ferroptotic demise of M2 macrophages triggers the catastrophic release of DAMPs, notably HMGB1. These DAMPs ligate TLR4 on surviving M1 macrophages, hyperactivating the STAT3 signaling axis and precipitating severe joint inflammation [698]. This metal‐driven M1 polarization, coupled with aberrant CD8^+^ T‐cell overactivation, orchestrates a destructive feed‐forward loop that perpetuates synovial fibroblast proliferation and cartilage erosion [699, 700]. Analogous iron‐dependent immune hyperactivation extends to other autoimmune landscapes. In SLE and lupus nephritis, pathological iron accumulation (facilitated by TfR1 upregulation and enhanced erythrophagocytosis by CD163^+^ tissue‐infiltrating macrophages) amplifies NF‐κB signaling and inflicts severe oxidative tissue damage [701, 702]. Concurrently, in progressive MS, NCOA4‐mediated ferritinophagy within microglia and infiltrating macrophages liberates redox‐active iron, fueling extensive lipid peroxidation and chronic neurodegeneration [662].

Recognizing these metal‐driven immunological etiologies has catalyzed the development of bidirectional therapeutic paradigms aimed at restoring systemic immune tolerance. A primary approach focuses on mitigating pathogenic ferroptosis to resolve aberrant inflammation. Pharmacological interventions and bioresponsive nanoplatforms engineered to fortify endogenous antioxidant networks, such as the SLC7A11‐GSH‐GPX4 and Nrf2‐GPX4 axes, or to promote cellular iron export via FPN, effectively arrest macrophage ferroptosis, reverse pro‐inflammatory M1 polarization, and alleviate autoimmune arthritis [703, 704, 705]. Furthermore, targeting specific intracellular signaling nodes, such as the inhibition of P2Y12 receptors, can successfully decouple the pathogenic crosstalk between iron‐driven lipid peroxidation and NLRP3 inflammasome hyperactivation [706]. Conversely, exploiting the principles of synthetic lethality, advanced metallo‐therapeutics can be designed to deliberately induce ferroptosis in hyperactive pathogenic cell populations. For instance, targeted nanosystems have been deployed to selectively trigger ROS‐mediated ferroptosis in pro‐inflammatory M1 macrophages [707] or to dismantle the acquired ferroptotic resistance of pathogenic synovial fibroblasts via dual‐targeting nanoplatforms, thereby arresting joint destruction [708, 709, 710]. Complementarily, our group recently demonstrated that copper fundamentally supports regulatory T cell energy metabolism, serving as a crucial metabolic checkpoint to sustain peripheral immune tolerance against autoimmunity [711]. Ultimately, the spatiotemporally precise modulation of metal‐dependent cell death, delicately balancing the induction of robust anti‐tumor immunity with the strict suppression of systemic autoimmunity, represents a critical and transformative frontier in next‐generation metalloimmunotherapy.

### Section summary

In summary, this chapter underscores a pivotal conceptual shift in metalloimmunotherapy: metal‐dependent RCD functions not merely as localized cytotoxicity, but as an initiator of systemic antitumor immune remodeling [435] and a driver of pathogenic sterile inflammation. Mechanistically, metal‐catalyzed oxidative stress drives the spatiotemporal emission of DAMPs to induce ICD [635]. Concurrently, manganese (Mn^2+^)‐mediated activation of the cGAS‐STING axis stimulates innate immunity, converting immunosuppressive TMEs into immune‐reactive niches enriched with M1 macrophages and CD8^+^ T cells [59]. However, metal‐induced stress presents inherent translational challenges. Excessive metal accumulation can provoke pro‐tumorigenic chronic inflammation, as evidenced by necrotic progression in glioblastoma [693], and serves as a fundamental pathogenic trigger in neurodegenerative disorders and autoimmune diseases like RA and SLE. Furthermore, effector T cells and dendritic cells (DCs) remain vulnerable to collateral lipid peroxidation; notably, dysregulated lipid metabolism induces lipid droplet accumulation in DCs, impairing antigen cross‐presentation [643]. Addressing these bottlenecks requires precise modulation of the TME. By employing responsive drug delivery systems with controlled release kinetics, targeted metal inducers can be synergized with immune checkpoint blockade (e.g., anti‐PD‐L1) [712]. This rational approach selectively sensitizes tumor cells to ferroptosis while preserving infiltrating lymphocytes, synergizing with checkpoint blockade to enhance antitumor immunity [103]. It strictly safeguards against unintended autoimmune toxicity and neuroinflammation by leveraging bidirectional therapeutic paradigms, such as fortifying endogenous tolerance or exploiting targeted synthetic lethality.

## CRITICAL ANALYSIS AND FUTURE PERSPECTIVES

### Overview

Despite the exponential progress in elucidating the molecular mechanisms of metal‐dependent RCD [14, 17], a formidable translational gap persists between preclinical discovery and clinical application. This disconnect is driven by three fundamental obstacles. First, a critical dichotomy exists between systemic toxicity and therapeutic selectivity; because metal‐dependent pathways exploit evolutionarily conserved housekeeping nodes (e.g., GPX4) [33], systemic induction can precipitate severe off‐target pathology in metabolically active organs, drastically narrowing the therapeutic index [53]. Second, clinical translation is hindered by pharmacological, manufacturing, and diagnostic bottlenecks, including poor in vivo drug‐likeness of ferroptosis tool compounds, CMC/GMP barriers for complex bioresponsive nanomedicines, and the lack of clinically validated noninvasive biomarkers [713, 714, 715]. Third, profound disparities between standard in vitro models and complex in vivo microenvironments frequently generate unexpected metabolic resistance. Addressing these constraints, this chapter critically dissects the systemic toxicity paradigm, navigates the complex microenvironmental barriers governing in vivo drug responsiveness, and evaluates prevailing translational barriers. By systematically integrating insights from active clinical trials (including precision medicinal chemistry optimizations and multimodal radio‐immunotherapies) and emphasizing the absolute necessity of biomarker‐guided patient stratification (e.g., utilizing serum LDH or multigene transcriptomic signatures, this chapter ultimately delineates a strategic roadmap for advancing fundamental metallobiology into precision clinical therapeutics.

### The challenge of systemic toxicity and selectivity

#### Broad‐spectrum toxicity and the narrow therapeutic index

Translating metal‐dependent RCD into clinical application is fundamentally constrained by a narrow therapeutic index. Although induction of ferroptosis, cuproptosis, and related ion‐driven programs offers a compelling approach for eliminating therapy‐refractory malignancies, these pathways are evolutionarily conserved and integral to normal tissue homeostasis. As a result, a mechanistic tension arises: processes that eliminate malignant cells, including lipid peroxidation, mitochondrial proteotoxic stress, and redox collapse, also threaten metabolically active organs such as the liver, kidneys, heart, and brain [95, 125, 716]. A principal obstacle to translation lies in the essential nature of core molecular targets. Small‐molecule ferroptosis inducers directed at GPX4 illustrate this limitation. Because GPX4 is a major lipid peroxidation scavenger required for cell survival, its specific deficiency in T cells induces ferroptosis and severely impairs humoral immune responses [717]. Experimental models demonstrate that *GPX4* knockout in mice results in acute renal failure and progressive neurodegeneration, indicating that systemic GPX4 inhibition confers an unacceptably narrow safety margin [718]. Likewise, the ER oxidoreductase POR, a critical mediator of ferroptotic execution, is indispensable for xenobiotic metabolism and steroid biosynthesis; its ablation produces embryonic lethality or profound developmental defects, limiting therapeutic feasibility [76].

These toxicities are substantiated clinically. The cardiotoxic profile of doxorubicin, a mainstay chemotherapeutic, has been reinterpreted as unintended induction of mitochondrial iron accumulation and ferroptosis in cardiomyocytes, mitigable through mitochondrial‐targeted iron chelation [16, 719]. Chronic exposure to metallotoxicants, such as arsenic, induces persistent pulmonary ferroptosis through gut‐lung axis signaling, illustrating how systemic metal imbalance drives distal organ pathology [198]. In neurodegenerative disease, cerebral iron accumulation actively contributes to cognitive decline in Alzheimer's disease, raising concern that systemic ferroptosis induction could exacerbate latent neurodegenerative processes in aged populations [44, 720]. Comparable limitations affect copper‐ and manganese‐based therapeutics. Copper ionophores, including elesclomol, effectively trigger cuproptosis yet produce hepatotoxicity and weight loss at systemic doses in preclinical models [28]. Because the liver functions as a primary copper reservoir through metallothionein induction, hepatic injury frequently precedes attainment of therapeutic intratumoral concentrations. Similarly, manganese‐based cytotoxic strategies are constrained by Mn neurotoxicity and by MICU1–MCU gatekeeping, which normally limits mitochondrial Mn^2+^ overload and oxidative cell death in nonmalignant cells [86, 364, 721]. Likewise, systemic cobalt overload from orthopedic implants provokes severe off‐target hepatotoxicity and neurodegeneration via unrestrained lipid peroxidation, emphasizing the narrow safety margins of metallotherapeutics [399, 401].

#### Clinical translation and biomarker‐guided stratification

The clinical consequences of these narrow safety margins are profoundly illustrated by the clinical trajectory of the copper ionophore elesclomol. Although elesclomol showed promising preclinical activity and acceptable early clinical tolerability, the pivotal phase III SYMMETRY trial of elesclomol plus paclitaxel in chemotherapy‐naïve advanced melanoma was stopped early after failing to improve PFS in an unselected population and showing an early imbalance in overall deaths, particularly among patients with elevated LDH [466, 480]. However, a retrospective subgroup analysis unveiled a critical mechanistic insight: patients exhibiting normal or low baseline levels of serum LDH, a surrogate biomarker indicating reliance on mitochondrial OXPHOS rather than hypoxia‐induced aerobic glycolysis, experienced a clinically meaningful 1.6‐month increase in median PFS. Because cuproptosis is strictly contingent upon active TCA cycle flux to drive the proteotoxic aggregation of lipoylated proteins, highly glycolytic tumors (reflected by elevated LDH levels) inherently resist copper‐induced cytotoxicity [465, 466]. In unstratified populations, systemic elesclomol administration fails to achieve tumoricidal thresholds; instead, the unassimilated heavy metal burden is shunted to the liver and other detoxification organs, exacerbating systemic toxicity. This high‐profile clinical failure serves as a cautionary paradigm: without rigorous patient stratification utilizing validated metabolic biomarkers, the systemic deployment of metal‐dependent RCD inducers inevitably yields off‐target multiorgan toxicity and diminished clinical efficacy [240, 465].

To circumvent this toxicity‐selectivity paradox, a new wave of clinical trials is increasingly deploying precision biomarker‐guided and spatiotemporally localized strategies to safely harness metal‐dependent RCD (Table 2). In oncology, a recent Phase II trial (ChiCTR2200066117) restricting enrollment exclusively to individuals with validated high *SLC7A11* expression demonstrated that combining SBRT with the system x_c_^−^ inhibitor sorafenib successfully overcame radiation resistance and prolonged PFS in patients with metastatic colorectal cancer [458]. Similarly, retrospective transcriptomic analyses of the Phase III STORM and IMbrave150 trials utilized a multigene signature (KUSS50) to precisely identify patients with HCC metabolically highly susceptible to ferroptosis, predicting maximal clinical benefit from sorafenib. Furthermore, an investigator‐initiated prospective trial (ChiCTR2100053441) confirmed that TLR3 activation via Poly(I:C) during radiotherapy significantly enhanced the abscopal effect in advanced HCC by promoting CD8^+^ T cell‐mediated tumor ferroptosis without exacerbating systemic toxicity [460]. Early‐phase trials (e.g., Phase I E20240009) are also actively evaluating targeted ferroptosis induction via low‐dose radiotherapy combined with anti‐PD‐1 ICB in refractory lung cancer [459], whereas other studies (e.g., Phase I/II NCT06374459) are exploring novel targets such as the MK2 inhibitor ATI‐450 to overcome ferroptosis resistance in metastatic breast cancer [449]. Beyond oncology, clinical trials deploying metal‐dependent pathway inhibitors for defensive tissue preservation, such as the ferroptosis inhibitor DIPY in Phase II trials (ChiCTR2300078059) for ARDS [474], the iron chelator deferiprone in Phase II/III trials (e.g., FAIRPARK II, NCT01539837) for Parkinson's disease [101, 469], and the copper chelator TTM in Phase II trials for Wilson's disease and osteoarthritis, further emphasize that precisely defining and monitoring the targeted metabolic state is a fundamental prerequisite for safe clinical translation.

#### Microenvironmental barriers and targeted delivery

Beyond systemic toxicity and metabolic buffering, therapeutic selectivity is further complicated by TME heterogeneity and immune cell vulnerability. A notable paradox is the sensitivity of effector lymphocytes to metal‐driven death. CD8^+^ T cells and T follicular helper cells depend on GPX4 activity; thus, indiscriminate ferroptosis induction may impair antitumor immunity and counteract the benefits of ICD [190, 717]. In parallel, tumor metabolic plasticity fosters resistance that necessitates potentially toxic dose escalation. Hypoxia‐inducible factor 1α (HIF‐1α), for instance, promotes cuproptosis resistance by suppressing lipoylated TCA enzymes and inducing metallothioneins, thereby requiring higher, hepatotoxic copper exposure to achieve tumoricidal thresholds [28]. Conversely, certain normal cell populations exhibit intrinsic hypersensitivity; microglia represent the most susceptible brain cell type to iron‐driven ferroptosis and actively propagate neurodegenerative pathology [722].

To address the permeability‐selectivity trade‐off, investigative focus has shifted from systemic small molecules toward bioresponsive nanomedicine and engineered delivery architectures [723]. Platforms exploiting TME‐specific physicochemical conditions enable molecular masking of catalytic activity during circulation. Nanoparticles incorporating acid‐labile linkages remain inert at physiological pH yet release payloads within acidic endosomes or intratumoral compartments, limiting systemic exposure and shielding copper‐enzyme catalytic interfaces until tumor localization [253, 724, 725]. Self‐amplifying systems that liberate copper ionophores only under elevated intratumoral ROS establish localized positive feedback loops, prolong tumor retention, and reduce hepatic deposition [635]. Separation of the targeting vector from the cytotoxic cargo represents an additional refinement. Two‐step bio‐orthogonal strategies permit mitochondrial pre‐targeting before payload administration, redistributing biodistribution away from renal clearance toward tumor enrichment [692]. Ligand functionalization, including holo‐lactoferrin and triphenylphosphonium conjugation, further concentrates metal ions within defined tissues, such as pulmonary tumors, while minimizing systemic dissemination [155].

Beyond delivery optimization, selective vulnerability can be achieved through the exploitation of metabolic thresholds. Cuproptosis remains contingent on OXPHOS; thus, glycolytic inhibition can sensitize tumors and permit lower copper dosing [214]. Rational target selection also widens safety margins. Genetic evidence indicates that SLC7A11 deletion is compatible with viability, whereas GPX4 loss is lethal, supporting cystine deprivation strategies over direct peroxidase inhibition. In neurodegenerative contexts, partial attenuation of mitochondrial calcium influx through MCU heterozygosity rescues pathology without the systemic toxicity observed with complete channel suppression, illustrating the value of graded modulation rather than full inhibition [497].

Future progress requires reframing systemic metal overload as precision metabolic tuning. Robust biomarker development is essential, as the lack of specific, broadly validated in vivo markers still complicates the discrimination of ferroptosis from accidental necrosis and apoptosis. Real‐time probes tracking lipid oxidation signatures or lipoylated protein aggregation will be critical for monitoring off‐target toxicity [726, 727]. Context‐dependent dosing paradigms are equally important. Metal ionophores such as elesclomol may function as copper chaperones at low concentrations yet induce cuproptosis at higher exposures, indicating that metabolic context and dose stratification determine outcome [728]. Consistent with this, appropriately dosed elesclomol safely rescues regulatory T cell oxidative phosphorylation, restoring peripheral immune tolerance without triggering cuproptosis [711]. Integration of metabolically coupled interventions, including HIF‐1α inhibition to reverse hypoxic resistance, may restore sensitivity to reduced metal doses and preserve detoxification organs. In summary, although systemic toxicity remains a central barrier to clinical translation of metal‐dependent RCD inducers, convergence of stimuli‐responsive delivery systems, bio‐orthogonal targeting, and metabolically informed patient stratification provides a pathway to deploy these lethal programs with greater therapeutic precision.

### Pharmacological, manufacturing, and diagnostic hurdles

Even with optimized delivery architectures and patient stratification, translating these precision strategies remains constrained by severe pharmacological, manufacturing, and diagnostic bottlenecks.

#### Pharmacological and manufacturing barriers

Specifically, a major bottleneck arises from the fact that most chemical probes used to establish the field display poor drug‐like characteristics, rendering them unsuitable for systemic use in humans. Prototypical ferroptosis inducers, such as erastin and RSL3, exhibit low aqueous solubility together with limited metabolic stability [95, 126]. For example, erastin shows a water solubility of only 0.086 × 10^−3^ M, which has driven development of structural analogs, including the metabolically stable imidazole ketone erastin (IKE), to achieve enhanced stability and solubility for in vivo tumor suppression [95]. To overcome these inherent pharmacological obstacles, rational medicinal chemistry optimization is actively bridging the gap between preclinical discovery and clinical application. For example, while the clinical translation of erastin was historically hindered by poor tumor tropism and inadequate cellular uptake, conjugating erastin to a Sigma‐2 receptor ligand generated a highly optimized derivative, ACXT‐3102 [440]. This modification drastically enhanced intracellular drug internalization, enabling the successful induction of robust lipid peroxidation in in vivo malic enzyme 1 (*ME1*)‐null synovial sarcoma models. Consequently, this targeted optimization established the requisite preclinical efficacy to propel ACXT‐3102 into Phase I human clinical trials (NCT02683148) [440]. However, the ester‐labile scaffold and poor in vivo stability of Fer‐1 limit its sustained application [729], prompting the development of more in vivo‐compatible ferroptosis inhibitors, including soluble/stable Fer‐1 analogs such as UAMC‐3203 [730, 731] and the potent radical‐trapping antioxidant Lip‐1 [53]. Consequently, the divergence between in vitro potency and in vivo pharmacokinetics often becomes so pronounced that foundational investigations rely on genetic manipulation or complementary genetic validation strategies to confirm pathway dependency, because existing inhibitors fail to reach therapeutic concentrations in animal models [55, 732].

Although nanomedicine approaches offer potential solutions to solubility limitations and off‐target toxicity, they introduce substantial CMC barriers that complicate clinical translation [733, 734, 735]. Fabrication of sophisticated platforms, including MXene@MnO_2_ heterojunctions or enzyme‐loaded nanoreactors [46, 155, 736], encounters major challenges related to batch reproducibility, manufacturing scale‐up, and preservation of enzymatic stability throughout production [737, 738]. In addition, interaction of nanomaterials with biological fluids generates a protein corona that alters particle surface characteristics, while sequestration by the mononuclear phagocyte system markedly reduces delivery efficiency. Together, these factors make extrapolation of human pharmacokinetics from animal studies notoriously unreliable [155]. As a result, successful translation of these advanced materials requires rigorous process standardization together with the development of scalable manufacturing strategies that remain unavailable for many academic nanoplatforms.

#### Diagnostic and biomarker limitations

Parallel to manufacturing hurdles, clinical implementation is fundamentally dependent on reliable, noninvasive biomarkers validated for clinical use. Unlike apoptosis, which can be definitively detected through caspase‐3 activation, metal‐dependent RCD lacks a universal irreversible commitment marker appropriate for clinical monitoring [739, 740]. Widely used experimental probes, such as C11‐BODIPY and Phen Green SK, display limited specificity and poor stability in complex tissue environments, making them unsuitable for diagnostic biopsies [741, 742]. Moreover, candidate markers identified in vitro frequently fail during translational evaluation; for instance, IL‐8 secretion, a strong indicator of microglial ferroptosis in culture, proved indistinguishable from generalized inflammation in Parkinson's disease tissue [722, 743] samples. Effective clinical translation, therefore, requires prospective validation of predictive biomarkers, including ACSL4 and ZEB1, as indicators of ferroptosis sensitivity [55] or MT2A as a determinant of cuproptosis resistance [28], in order to identify patient populations with defined metabolic vulnerabilities.

The critical necessity of such biomarker‐guided patient stratification is increasingly corroborated by clinical trial outcomes. For instance, retrospective analyses of Phase III trials evaluating the copper ionophore elesclomol in advanced melanoma revealed that while unselected cohorts failed to meet primary survival endpoints, a stratified subpopulation with low serum LDH, indicative of a strict reliance on mitochondrial OXPHOS, exhibited significantly improved progression‐free survival [240, 465]. Similarly, transcriptomic evaluation of the Phase III STORM trial demonstrated that hepatocellular carcinoma patients harboring a distinct 50‐gene signature (KUSS50) are robustly primed for sorafenib‐induced ferroptosis, thereby maximizing therapeutic benefit. Parallel efforts establishing sodium‐overload transcriptomic signatures, such as NEK8‐driven panels, now offer vital prognostic frameworks to successfully stratify patients and guide immune checkpoint blockade in gastrointestinal malignancies [312, 326]. Moving beyond retrospective observations, an investigator‐initiated Phase II trial (ChiCTR2200066117) prospectively stratified colorectal cancer patients with liver metastases based on elevated *SLC7A11* expression. This precision approach enabled sorafenib to synergize effectively with stereotactic body radiotherapy (SBRT), significantly prolonging progression‐free survival by exacerbating iron‐dependent lipid peroxidation and overcoming canonical radiation resistance [458].

#### In vitro−in vivo translational disparities

Ultimately, the success of biomarker‐guided translation is frequently derailed by a final barrier: the assumption that mechanisms observed in vitro faithfully replicate those operating in vivo. Tissue microenvironments and systemic metabolic conditions exert powerful control over metal‐dependent death pathways, frequently generating resistance mechanisms absent in cultured cells. The redox conditions of conventional cell culture media, which contain high cystine concentrations, differ fundamentally from physiological environments where cysteine predominates, producing substantial differences in drug responsiveness. Moreover, anatomical tumor location can dictate susceptibility; melanoma cells residing in lymphatic tissues are protected from ferroptosis through elevated oleic acid and ACSL3 levels, rendering systemic ferroptosis inducers ineffective against lymphatic metastases despite strong activity in circulating tumor cells [176, 744, 745]. Likewise, inflammatory cytokines such as TNF may enhance GSH biosynthesis in synovial fibroblasts, thereby increasing the ferroptotic threshold, whereas hypoxic signaling stabilizes HIF‐1α and suppresses cuproptosis‐related targets [746]. Crucially, driven by the chronic metabolic stress endemic to these restrictive in vivo niches, cellular subpopulations frequently acquire Ferroptosis Adaptive Tolerance (FAT)—an evolutionarily conserved, GPX4‐independent cellular escape mechanism. By orchestrating the adaptive remodeling of redox and lipid networks, these cells evade lethal lipid peroxidation and persist in a dedifferentiated state, thereby establishing a deep, latent pathogenic reservoir that relentlessly fuels disease chronicity and multidrug resistance [213]. Overcoming these limitations requires shifting from identification of potent monotherapies toward development of metallometabolic therapeutic strategies [747] that exploit defined metabolic dependencies while incorporating bioresponsive delivery systems [745]. Future investigations should therefore prioritize disease models that better recapitulate irreversible human pathology, together with validation of biomarkers capable of guiding patient stratification in clinical trials.

Encouragingly, an expanding repertoire of multimodal regimens explicitly designed to remodel the immunosuppressive in vivo microenvironment and therapeutically trigger metal‐dependent RCD is currently advancing through human clinical trials. In oncology, an ongoing Phase I trial (E20240009) is combining low‐dose radiotherapy with anti‐PD‐1 ICB to deliberately suppress the Nrf2/HO‐1/GPX4 axis, successfully inducing tumoral ferroptosis to combat chemoimmunotherapy‐resistant nonsmall cell lung cancer [459]. Concurrently, an investigator‐initiated prospective trial (ChiCTR2100053441) established that administering the TLR3 agonist Poly(I:C) alongside radiotherapy safely promotes CD8^+^ T cell‐mediated tumor ferroptosis, dramatically reinforcing the abscopal effect in advanced HCC [460]. In an effort to reverse intrinsic ferroptosis resistance within the TME, the MK2 inhibitor ATI‐450 is advancing through Phase I/II evaluation (NCT06374459) for metastatic breast cancer [449]. Beyond oncology, clinical interventions aimed at preserving tissue viability via targeted pathway inhibition highlight both the promise and profound complexity of clinical translation. Although early studies displayed potential, the recent large‐scale FAIRPARK II Phase II trial (NCT01539837) for early‐stage Parkinson's disease demonstrated that although the iron chelator DFP successfully reduced brain iron (confirming target engagement), it unexpectedly worsened motor symptoms in levodopa‐naïve patients [471]. This critical finding underscores that iron plays a dual role, acting both as a toxic mediator of lipid peroxidation and an essential cofactor for dopamine synthesis, necessitating highly refined, disease‐stage‐specific interventions. Conversely, the FDA‐approved agent DIPY recently demonstrated proof‐of‐concept clinical efficacy (ChiCTR2300078059) as a novel adjunctive ferroptosis inhibitor, significantly mitigating lipid peroxidation and pulmonary endothelial injury in patients with ARDS [474]. Ultimately, these milestone clinical trials validate that despite persistent pharmacological and diagnostic hurdles, the rational modulation of metal‐dependent cell death networks is rapidly transitioning from foundational discovery into a transformative pillar of precision medicine (Table 2).

### Section summary

Translating metal‐dependent RCD from mechanistic discovery to clinical application is currently limited by significant translational barriers. A primary discrepancy exists between standard in vitro culture models, characterized by hyper‐physiological oxygen and cystine levels, and the restrictive in vivo solid TME, which features hypoxia, metabolic plasticity [28], and lipid‐mediated lymphatic shielding [176]. Furthermore, therapeutic translation is hindered by the suboptimal pharmacokinetics of chemical probes (necessitating targeted medicinal chemistry optimizations, such as receptor‐ligand conjugation), the manufacturing complexities of nanomedicines [190], and the systemic toxicity associated with targeting conserved housekeeping enzymes [53] or disrupting the context‐dependent physiological roles of essential biometals. This is compounded by a lack of validated, noninvasive in vivo molecular probes capable of differentiating specific metal‐dependent RCDs from accidental necrosis or apoptosis [136], alongside an urgent requirement for predictive diagnostic signatures (e.g., serum LDH, SLC7A11 expression, or the KUSS50 transcriptomic panel) to guide precise patient stratification. To advance clinical translation, researchers must prioritize physiologically relevant in situ tumor and chronic disease models that accurately recapitulate these complex metabolic dependencies. Ultimately, by developing specific in vivo molecular biomarkers and integrating bioresponsive materials science, immunology (e.g., synergizing radiotherapy with immune checkpoint blockade or TLR3 agonists), and metabolomics, metal‐targeted interventions can overcome current limitations to advance precision medicine [151].

## CONCLUSION

The conceptual evolution of metal‐induced cytotoxicity, progressing from indiscriminate oxidative injury toward a highly coordinated network of RCD, represents a major transformation in molecular pathology. Transition and alkaline earth metals, including iron, copper, calcium, zinc, and manganese, act as finely tuned rheostats controlling cellular fate. When compartmentalized homeostatic thresholds are exceeded, these ions initiate mechanistically distinct lethal programs: membrane lipid peroxidation characteristic of ferroptosis, proteotoxic mitochondrial arrest associated with cuproptosis, and organelle‐restricted bioenergetic collapse underlying calcicoptosis, zincoptosis, and mnoptosis. Importantly, these death modalities rarely function independently; instead, they intersect within a dynamic in vivo continuum of interconnected death pathways collectively conceptualized as PANoptosis. Understanding this structural complexity establishes a bidirectional therapeutic imperative: exploiting synthetic lethality and metabolic gating with targeted agonists to selectively eliminate therapy‐resistant malignancies while deploying repurposed clinical‐grade pharmacological inhibitors that strengthen endogenous cytoprotective buffers against degenerative tissue loss and pathogenic sterile inflammation.

Translating the biological promise of metal‐dependent RCD into clinical practice requires moving beyond single‐pathway reductionism, together with overcoming the dose‐limiting liabilities associated with systemic elemental overload. The emerging translational frontier lies at the convergence of multiparametric biomarker‐guided stratification (integrating high‐resolution lipidomic and metabolic signatures), bioresponsive materials engineering, and tightly controlled spatiotemporal immunomodulation. By designing stimuli‐responsive nanomedicines and single‐atom catalytic systems capable of confining lethal oxidative flux within pathological niches, widespread multiorgan toxicity may be substantially mitigated. Moreover, repurposing localized metal‐driven bioenergetic collapse to stimulate robust DAMP emission, systemic cGAS–STING activation, and ICD establishes an important frontier in metalloimmunotherapy that reshapes immunosuppressive microenvironments and enhances responsiveness to ICB.

Given the inherent multidimensional complexity of metal homeostasis and diverse metal‐regulated cell death cascades, we further propose interdisciplinary theoretical frameworks, including Ferrology, Zincology, and Cuprology, to systematically unify elemental metabolic biology and programmed cell death research. Ultimately, bridging the persistent translational gap requires shifting the clinical paradigm toward precise metallometabolic tuning. Through rigorous validation of in vivo molecular signatures, together with the development of targeted delivery architectures for multimodal combinatorial regimens, controlled modulation of metal‐dependent vulnerabilities may overcome existing pharmacological limitations and position this field as a central pillar of precision medicine.

## AUTHOR CONTRIBUTIONS

**Haoliang Hu**: conceptualization; visualization; writing – original draft; writing – review and editing; funding acquisition; project administration. **Zhe Chen**: writing – original draft; writing – review and editing. **Yaqi Li**: writing – original draft; writing – review and editing. **Jiayi Peng**: writing – original draft; writing – review and editing. **Jiangang Cao**: writing – original draft; writing – review and editing. **Hong Zhou**: writing – original draft; writing – review and editing. **Mengqi Wang**: writing – original draft; writing – review and editing. **Yuejia Du**: writing – original draft; writing – review and editing; visualization. **Hailin Wu**: writing – original draft; writing – review and editing; visualization. **Huiqin Zhao**: writing – original draft; writing – review and editing; visualization. **Shifang Huang**: writing – original draft; writing – review and editing. **Dianmei Yu**: writing – original draft; writing – review and editing; visualization. **Meiqing Liu**: writing – original draft; writing – review and editing. **Olga V. Shevchenko**: writing – original draft; writing – review and editing. **Natalia Yu Matveeva:** writing – original draft; writing – review and editing. **Yiyuan Yang**: writing – original draft; writing – review and editing. **Kerui Huang**: conceptualization; writing – original draft; writing – review and editing; visualization; project administration; funding acquisition. **Deguan Lv**: conceptualization; writing – original draft; writing – review and editing; funding acquisition; visualization; project administration. **Junxia Min**: conceptualization; funding acquisition; visualization; project administration; writing – review and editing; writing – original draft. **Linxi Chen**: conceptualization; writing – original draft; funding acquisition; visualization; writing – review and editing; project administration. **Fudi Wang**: conceptualization; funding acquisition; visualization; project administration; writing – review and editing; writing – original draft. All authors have read the final manuscript and approved it for publication.

## CONFLICT OF INTEREST

The authors declare no conflicts of interest.

## ETHICS STATEMENT

No animals or humans were involved in this study.

## Data Availability

Data sharing is not applicable to this article as no new data were created or analyzed in this study. Supporting Information (graphical abstract, slides, videos, Chinese translated version, and update materials) may be found in the online DOI or *iMeta* Science http://www.imeta.science/.

## References

[1] Stockwell, Brent R. , José Pedro Friedmann Angeli , Hülya Bayir , Ashley I. Bush , Marcus Conrad , Scott J. Dixon , Simone Fulda , et al. 2017. “Ferroptosis: A Regulated Cell Death Nexus Linking Metabolism, Redox Biology, and Disease.” Cell 171: 273–285. 10.1016/j.cell.2017.09.021 28985560 PMC5685180

[2] Berghe, Tom Vanden , Andreas Linkermann , Sandrine Jouan‐Lanhouet , Henning Walczak , and Peter Vandenabeele . 2014. “Regulated Necrosis: the Expanding Network of Non‐Apoptotic Cell Death Pathways.” Nature Reviews Molecular Cell Biology 15: 135–147. 10.1038/nrm3737 24452471

[3] Jiang, Xuejun , Brent R. Stockwell , and Marcus Conrad . 2021. “Ferroptosis: Mechanisms, Biology and Role in Disease.” Nature Reviews Molecular Cell Biology 22: 266–282. 10.1038/s41580-020-00324-8 33495651 PMC8142022

[4] Tang, Daolin , Rui Kang , Tom Vanden Berghe , Peter Vandenabeele , and Guido Kroemer . 2019. “The Molecular Machinery of Regulated Cell Death.” Cell Research 29: 347–364. 10.1038/s41422-019-0164-5 30948788 PMC6796845

[5] Dixon, Scott J. , and Brent R. Stockwell . 2014. “The Role of Iron and Reactive Oxygen Species in Cell Death.” Nature Chemical Biology 10: 9–17. 10.1038/nchembio.1416 24346035

[6] Galluzzi, Lorenzo , Ilio Vitale , Stuart A. Aaronson , John M. Abrams , Dieter Adam , Patrizia Agostinis , Emad S. Alnemri , et al. 2018. “Molecular Mechanisms of Cell Death: Recommendations of the Nomenclature Committee on Cell Death 2018.” Cell Death & Differentiation 25: 486–541. 10.1038/s41418-017-0012-4 29362479 PMC5864239

[7] Wang, Huan , Anqi Wang , Xinqiao Wang , Xiangyin Zeng , and Houjuan Xing . 2022. “AMPK/PPAR‐γ/NF‐κB Axis Participates in ROS‐Mediated Apoptosis and Autophagy Caused by Cadmium in Pig Liver.” Environmental Pollution 294: 118659. 10.1016/j.envpol.2021.118659 34896222

[8] He, Lihua , Guy A. Perkins , Ann T. Poblenz , Jeffrey B. Harris , Michael Hung , Mark H. Ellisman , and Donald A. Fox . 2003. “Bcl‐xL Overexpression Blocks Bax‐Mediated Mitochondrial Contact Site Formation and Apoptosis in Rod Photoreceptors of Lead‐Exposed Mice.” Proceedings of the National Academy of Sciences 100: 1022–1027. 10.1073/pnas.0333594100 PMC298719 12540825

[9] Tonnus, Wulf , Claudia Meyer , Christian Steinebach , Alexia Belavgeni , Anne von Mässenhausen , Nadia Zamora Gonzalez , Francesca Maremonti , et al. 2021. “Dysfunction of the Key Ferroptosis‐Surveilling Systems Hypersensitizes Mice to Tubular Necrosis During Acute Kidney Injury.” Nature Communications 12: 4402. 10.1038/s41467-021-24712-6 PMC8292346 34285231

[10] Logan, Clare V. , György Szabadkai , Jenny A. Sharpe , David A. Parry , Silvia Torelli , Anne‐Marie Childs , Marjolein Kriek , et al. 2014. “Loss‐Of‐Function Mutations in MICU1 Cause a Brain and Muscle Disorder Linked to Primary Alterations in Mitochondrial Calcium Signaling.” Nature Genetics 46: 188–193. 10.1038/ng.2851 24336167

[11] Mallilankaraman, Karthik , Patrick Doonan , César Cárdenas , Harish C. Chandramoorthy , Marioly Müller , Russell Miller , Nicholas E. Hoffman , et al. 2012. “MICU1 Is an Essential Gatekeeper for MCU‐Mediated Mitochondrial Ca^2+^ Uptake That Regulates Cell Survival.” Cell 151: 630–644. 10.1016/j.cell.2012.10.011 23101630 PMC3486697

[12] Berridge, Michael J. , Martin D. Bootman , and Peter Lipp . 1998. “Calcium—A Life and Death Signal.” Nature 395: 645–648. 10.1038/27094 9790183

[13] Hu, Ruizhi , Junchang Qin , Wei Feng , Xinran Song , Hui Huang , Chen Dai , Bo Zhang , and Yu Chen . 2025. “Lysosomal Zinc Nanomodulation Blocks Macrophage Pyroptosis for Counteracting Atherosclerosis Progression.” Science Advances 11: eadu3919. 10.1126/sciadv.adu3919 40561010 PMC12189970

[14] Dixon, Scott J. , Kathryn M. Lemberg , Michael R. Lamprecht , Rachid Skouta , Eleina M. Zaitsev , Caroline E. Gleason , Darpan N. Patel , et al. 2012. “Ferroptosis: An Iron‐Dependent Form of Nonapoptotic Cell Death.” Cell 149: 1060–1072. 10.1016/j.cell.2012.03.042 22632970 PMC3367386

[15] Tang, Daolin , Xin Chen , Rui Kang , and Guido Kroemer . 2021. “Ferroptosis: Molecular Mechanisms and Health Implications.” Cell Research 31: 107–125. 10.1038/s41422-020-00441-1 33268902 PMC8026611

[16] Fang, Xuexian , Hossein Ardehali , Junxia Min , and Fudi Wang . 2023. “The Molecular and Metabolic Landscape of Iron and Ferroptosis in Cardiovascular Disease.” Nature Reviews Cardiology 20: 7–23. 10.1038/s41569-022-00735-4 35788564 PMC9252571

[17] Tsvetkov, Peter , Shannon Coy , Boryana Petrova , Margaret Dreishpoon , Ana Verma , Mai Abdusamad , Jordan Rossen , et al. 2022. “Copper Induces Cell Death by Targeting Lipoylated TCA Cycle Proteins.” Science 375: 1254–1261. 10.1126/science.abf0529 35298263 PMC9273333

[18] Yang, Lingxiao , Kaiyue Wang , Jia Dong , Xuening Zhang , Xiaoyang Liu , Jiarong Cui , Jin Liu , Min Zhou , and Kai Wang . 2025. “Biomineralized Microspheres Trigger Synergistic Calcicoptosis‐Ferroptosis for Enhanced Non‐Small Cell Lung Cancer Therapy.” Acta Biomaterialia 207: 617–632. 10.1016/j.actbio.2025.09.053 41043671

[19] Giorgio, Valentina , Sophia von Stockum , Manuela Antoniel , Astrid Fabbro , Federico Fogolari , Michael Forte , Gary D. Glick , et al. 2013. “Dimers of Mitochondrial ATP Synthase Form the Permeability Transition Pore.” Proceedings of the National Academy of Sciences 110: 5887–5892. 10.1073/pnas.1217823110 PMC3625323 23530243

[20] Qiu, Yue , Hongyang Wang , Mingjie Fan , Huaye Pan , Jing Guan , Yangwei Jiang , Zexiao Jia , et al. 2023. “Impaired AIF‐CHCHD4 Interaction and Mitochondrial Calcium Overload Contribute to Auditory Neuropathy Spectrum Disorder in Patient‐iPSC‐Derived Neurons With AIFM1 Variant.” Cell Death & Disease 14: 375. 10.1038/s41419-023-05899-6 37365177 PMC10293272

[21] Gao, Yang , Ziting Xu , Yingshan Gao , Li Zhang , Yu Liang , Qiuyu Li , Minyi Liu , et al. 2025. “Low‐Intensity Pulsed Ultrasound (LIPUS)‐Augmented Calcicoptosis by Calcium‐Based Nanoparticles for Enhanced Immunogenic Cell Death Induction in Triple‐Negative Breast Cancer.” Journal of Nanobiotechnology 24: 60. 10.1186/s12951-025-03914-w 41408548 PMC12822242

[22] Qi, Jiansong , Yanhong Xing , Yucheng Liu , Meng‐Meng Wang , Xiangqing Wei , Zhongheng Sui , Lin Ding , et al. 2021. “MCOLN1/TRPML1 Finely Controls Oncogenic Autophagy in Cancer by Mediating Zinc Influx.” Autophagy 17: 4401–4422. 10.1080/15548627.2021.1917132 33890549 PMC8726724

[23] Pei, Xingyao , Haiyang Jiang , Cun Li , Daowen Li , and Shusheng Tang . 2023. “Oxidative Stress‐Related Canonical Pyroptosis Pathway, as a Target of Liver Toxicity Triggered by Zinc Oxide Nanoparticles.” Journal of Hazardous Materials 442: 130039. 10.1016/j.jhazmat.2022.130039 36166902

[24] Xi, Zi‐Yue , Chuan‐Yong Fan , Ying‐Ying Jiang , Xin‐Ran Xi , Gan‐Yu Nie , Shuang Zhu , Jun‐Jie Zhang , and Lu Xu . 2025. “Nanocatalytic System Releases Overloaded Zinc Ions and ROS to Induce Znproptosis and Interrupt Cell Cycle Through Inhibiting Akt/mTOR Pathway.” Theranostics 15: 4734–4762. 10.7150/thno.107025 40225560 PMC11984402

[25] Zhou, Jia‐Ying , Qing‐Hua Shen , Yu‐Wen Xiong , Jia‐Wen Chen , Qi Hui , Rui Zhou , Yi‐Fei Luo , et al. 2025. “Zinc‐Mediated Metalloimmunotherapy With Dual Elimination of Tumor and Intratumoral Bacteria in Oral Squamous Cell Carcinoma.” Biomaterials 323: 123439. 10.1016/j.biomaterials.2025.123439 40450766

[26] Tesfay, Lia , Bibbin T. Paul , Anna Konstorum , Zhiyong Deng , Anderson O. Cox , Jingyun Lee , Cristina M. Furdui , et al. 2019. “Stearoyl‐CoA Desaturase 1 Protects Ovarian Cancer Cells From Ferroptotic Cell Death.” Cancer Research 79: 5355–5366. 10.1158/0008-5472.CAN-19-0369 31270077 PMC6801059

[27] Bersuker, Kirill , Joseph M. Hendricks , Zhipeng Li , Leslie Magtanong , Breanna Ford , Peter H. Tang , Melissa A. Roberts , et al. 2019. “The CoQ Oxidoreductase FSP1 Acts Parallel to GPX4 to Inhibit Ferroptosis.” Nature 575: 688–692. 10.1038/s41586-019-1705-2 31634900 PMC6883167

[28] Yang, Zhou , Wei Su , Xiyi Wei , Yitong Pan , Mengying Xing , Lili Niu , Baijie Feng , et al. 2025. “Hypoxia Inducible factor‐1α Drives Cancer Resistance to Cuproptosis.” Cancer Cell 43: 937–954.e9. 10.1016/j.ccell.2025.02.015 40054467

[29] Doll, Sebastian , Bettina Proneth , Yulia Y. Tyurina , Elena Panzilius , Sho Kobayashi , Irina Ingold , Martin Irmler , et al. 2017. “ACSL4 Dictates Ferroptosis Sensitivity by Shaping Cellular Lipid Composition.” Nature Chemical Biology 13: 91–98. 10.1038/nchembio.2239 27842070 PMC5610546

[30] Kagan, Valerian E. , Gaowei Mao , Feng Qu , Jose Pedro Friedmann Angeli , Sebastian Doll , Claudette St Croix , Haider Hussain Dar , et al. 2017. “Oxidized Arachidonic and Adrenic PEs Navigate Cells to Ferroptosis.” Nature Chemical Biology 13: 81–90. 10.1038/nchembio.2238 27842066 PMC5506843

[31] Kirichok, Yuriy , Grigory Krapivinsky , and David E. Clapham . 2004. “The Mitochondrial Calcium Uniporter Is a Highly Selective Ion Channel.” Nature 427: 360–364. 10.1038/nature02246 14737170

[32] Kanzawa, Takao , Li Zhang , Lianchun Xiao , Isabelle M. Germano , Yasuko Kondo , and Seiji Kondo . 2005. “Arsenic Trioxide Induces Autophagic Cell Death in Malignant Glioma Cells by Upregulation of Mitochondrial Cell Death Protein BNIP3.” Oncogene 24: 980–991. 10.1038/sj.onc.1208095 15592527

[33] Yang, Wan Seok , Rohitha SriRamaratnam , Matthew E. Welsch , Kenichi Shimada , Rachid Skouta , Vasanthi S. Viswanathan , Jaime H. Cheah , et al. 2014. “Regulation of Ferroptotic Cancer Cell Death by GPX4.” Cell 156: 317–331. 10.1016/j.cell.2013.12.010 24439385 PMC4076414

[34] Hou, Wen , Yangchun Xie , Xinxin Song , Xiaofang Sun , Michael T. Lotze , Herbert J. Zeh Iii , Rui Kang , and Daolin Tang . 2016. “Autophagy Promotes Ferroptosis by Degradation of Ferritin.” Autophagy 12: 1425–1428. 10.1080/15548627.2016.1187366 27245739 PMC4968231

[35] Li, Yaxu , Qiao Ran , Qiuhui Duan , Jiali Jin , Yanjin Wang , Lei Yu , Chaojie Wang , et al. 2024. “7‐Dehydrocholesterol Dictates Ferroptosis Sensitivity.” Nature 626: 411–418. 10.1038/s41586-023-06983-9 38297130 PMC11298758

[36] Huang, Wenxiang , Ruijun Wang , Xinquan Yang , Shuangjie Yang , Xueliang Yang , Gaolu He , Songjun Dai , et al. 2026. “Stabilizing MARCH7 as a Ferro‐Guardian Against Ferroptosis.” Cell 189: 3553–3570.e30. 10.1016/j.cell.2026.03.052 42049018

[37] Wang, Chenguang , Yukun Guan , Mengze Lv , Rui Zhang , Zhaoying Guo , Xiaoming Wei , Xiaoxia Du , et al. 2018. “Manganese Increases the Sensitivity of the cGAS‐STING Pathway for Double‐Stranded DNA and Is Required for the Host Defense Against DNA Viruses.” Immunity 48: 675–687.e7. 10.1016/j.immuni.2018.03.017 29653696

[38] Zhang, Meng , Ruixue Song , Yanyan Liu , Zhigao Yi , Xianfu Meng , Jiawen Zhang , Zhongmin Tang , et al. 2019. “Calcium‐Overload‐Mediated Tumor Therapy by Calcium Peroxide Nanoparticles.” Chem 5: 2171–2182. 10.1016/j.chempr.2019.06.003

[39] Liu, Xiaoguang , Litong Nie , Yilei Zhang , Yuelong Yan , Chao Wang , Medina Colic , Kellen Olszewski , et al. 2023. “Actin Cytoskeleton Vulnerability to Disulfide Stress Mediates Disulfidptosis.” Nature Cell Biology 25: 404–414. 10.1038/s41556-023-01091-2 36747082 PMC10027392

[40] Wang, Xue , Qian Wu , Meijuan Zhong , Ying Chen , Yudi Wang , Xin Li , Wenxi Zhao , et al. 2025. “Adipocyte‐Derived Ferroptotic Signaling Mitigates Obesity.” Cell Metabolism 37: 673–691. 10.1016/j.cmet.2024.11.010 39729998

[41] Yang, Wan Seok , and Brent R. Stockwell . 2016. “Ferroptosis: Death by Lipid Peroxidation.” Trends in Cell Biology 26: 165–176. 10.1016/j.tcb.2015.10.014 26653790 PMC4764384

[42] Tsoi, Jennifer , Lidia Robert , Kim Paraiso , Carlos Galvan , Katherine M. Sheu , Johnson Lay , Deborah J. L. Wong , et al. 2018. “Multi‐Stage Differentiation Defines Melanoma Subtypes with Differential Vulnerability to Drug‐Induced Iron‐Dependent Oxidative Stress.” Cancer Cell 33: 890–904.e5. 10.1016/j.ccell.2018.03.017 29657129 PMC5953834

[43] Hangauer, Matthew J. , Vasanthi S. Viswanathan , Matthew J. Ryan , Dhruv Bole , John K. Eaton , Alexandre Matov , Jacqueline Galeas , et al. 2017. “Drug‐Tolerant Persister Cancer Cells Are Vulnerable to GPX4 Inhibition.” Nature 551: 247–250. 10.1038/nature24297 29088702 PMC5933935

[44] Ayton, Scott , Yamin Wang , Ibrahima Diouf , Julie A. Schneider , John Brockman , Martha Clare Morris , and Ashley I. Bush . 2020. “Brain Iron Is Associated With Accelerated Cognitive Decline in People With Alzheimer Pathology.” Molecular Psychiatry 25: 2932–2941. 10.1038/s41380-019-0375-7 30778133 PMC6698435

[45] Tsai, Chen‐Wei , Madison X. Rodriguez , Anna M. Van Keuren , Charles B. Phillips , Hannah M. Shushunov , Jessica E. Lee , Anastacia M. Garcia , et al. 2022. “Mechanisms and Significance of Tissue‐Specific MICU Regulation of the Mitochondrial Calcium Uniporter Complex.” Molecular Cell 82: 3661–3676.e8. 10.1016/j.molcel.2022.09.006 36206740 PMC9557913

[46] Liu, Shijian , Sijie Shao , Zhe Huang , Panpan Xue , Shuangqian Yan , Mengru Cao , and Xuemei Zeng . 2026. “A Multifunctional Manganese‐Based Nanozyme Platform for Synergistic Hypoxia Alleviation and Cholesterol Depletion to Potentiate STING‐Mediated Cancer Immunotherapy.” Biomaterials 327: 123760. 10.1016/j.biomaterials.2025.123760 41045760

[47] Zhang, Yiqun , Ni Zhang , Jianghao Xing , Yiwei Sun , Xu Jin , Cailiang Shen , Liang Cheng , Yuanyin Wang , and Xianwen Wang . 2024. “In Situ Hydrogel Based on Cu–Fe_3_O_4_ Nanoclusters Exploits Oxidative Stress and the Ferroptosis/Cuproptosis Pathway for Chemodynamic Therapy.” Biomaterials 311: 122675. 10.1016/j.biomaterials.2024.122675 38943822

[48] Grad, Jennifer M. , Nizar J. Bahlis , Isildinha Reis , Marc M. Oshiro , William S. Dalton , and Lawrence H. Boise . 2001. “Ascorbic Acid Enhances Arsenic Trioxide‐Induced Cytotoxicity in Multiple Myeloma Cells.” Blood 98: 805–813. 10.1182/blood.v98.3.805 11468182

[49] Davison, K. , S. Côté , S. Mader , and W. H. Miller . 2003. “Glutathione Depletion Overcomes Resistance to Arsenic Trioxide in Arsenic‐Resistant Cell Lines.” Leukemia 17: 931–940. 10.1038/sj.leu.2402876 12750708

[50] Mallilankaraman, Karthik , César Cárdenas , Patrick J. Doonan , Harish C. Chandramoorthy , Krishna M. Irrinki , Tünde Golenár , György Csordás , et al. 2012. “MCUR1 Is an Essential Component of Mitochondrial Ca^2+^ Uptake That Regulates Cellular Metabolism.” Nature Cell Biology 14: 1336–1343. 10.1038/ncb2622 23178883 PMC3511605

[51] Kang, Young‐Hee , Min‐Jung Yi , Min‐Jung Kim , Moon‐Taek Park , Sangwoo Bae , Chang‐Mo Kang , Chul‐Koo Cho , et al. 2004. “Caspase‐Independent Cell Death by Arsenic Trioxide in Human Cervical Cancer Cells.” Cancer Research 64: 8960–8967. 10.1158/0008-5472.CAN-04-1830 15604259

[52] Kanzawa, Takao , Yasuko Kondo , Hideaki Ito , Seiji Kondo , and Isabelle Germano . 2003. “Induction of Autophagic Cell Death in Malignant Glioma Cells by Arsenic Trioxide.” Cancer Research 63: 2103–2108 https://www.ncbi.nlm.nih.gov/pubmed/12727826 12727826

[53] Friedmann Angeli, Jose Pedro , Manuela Schneider , Bettina Proneth , Yulia Y. Tyurina , Vladimir A. Tyurin , Victoria J. Hammond , Nadja Herbach , et al. 2014. “Inactivation of the Ferroptosis Regulator Gpx4 Triggers Acute Renal Failure in Mice.” Nature Cell Biology 16: 1180–1191. 10.1038/ncb3064 25402683 PMC4894846

[54] Mancias, Joseph D. , Xiaoxu Wang , Steven P. Gygi , J. Wade Harper , and Alec C. Kimmelman . 2014. “Quantitative Proteomics Identifies NCOA4 as the Cargo Receptor Mediating Ferritinophagy.” Nature 509: 105–109. 10.1038/nature13148 24695223 PMC4180099

[55] Viswanathan, Vasanthi S. , Matthew J. Ryan , Harshil D. Dhruv , Shubhroz Gill , Ossia M. Eichhoff , Brinton Seashore‐Ludlow , Samuel D. Kaffenberger , et al. 2017. “Dependency of a Therapy‐Resistant State of Cancer Cells on a Lipid Peroxidase Pathway.” Nature 547: 453–457. 10.1038/nature23007 28678785 PMC5667900

[56] Doll, Sebastian , Florencio Porto Freitas , Ron Shah , Maceler Aldrovandi , Milene Costa da Silva , Irina Ingold , Andrea Goya Grocin , et al. 2019. “FSP1 Is a Glutathione‐Independent Ferroptosis Suppressor.” Nature 575: 693–698. 10.1038/s41586-019-1707-0 31634899

[57] Kraft, Vanessa A. N. , Carla T. Bezjian , Susanne Pfeiffer , Larissa Ringelstetter , Constanze Müller , Fereshteh Zandkarimi , Juliane Merl‐Pham , et al. 2020. “GTP Cyclohydrolase 1/Tetrahydrobiopterin Counteract Ferroptosis Through Lipid Remodeling.” ACS Central Science 6: 41–53. 10.1021/acscentsci.9b01063 31989025 PMC6978838

[58] Mao, Chao , Xiaoguang Liu , Yilei Zhang , Guang Lei , Yuelong Yan , Hyemin Lee , Pranavi Koppula , et al. 2021. “DHODH‐mediated Ferroptosis Defence Is a Targetable Vulnerability in Cancer.” Nature 593: 586–590. 10.1038/s41586-021-03539-7 33981038 PMC8895686

[59] Lv, Mengze , Meixia Chen , Rui Zhang , Wen Zhang , Chenguang Wang , Yan Zhang , Xiaoming Wei , et al. 2020. “Manganese Is Critical for Antitumor Immune Responses Via cGAS‐STING and Improves the Efficacy of Clinical Immunotherapy.” Cell Research 30: 966–979. 10.1038/s41422-020-00395-4 32839553 PMC7785004

[60] Du, Wanlu , Mingxue Gu , Meiqin Hu , Prateeksunder Pinchi , Wei Chen , Michael Ryan , Timothy Nold , Ahmed Bannaga , and Haoxing Xu . 2021. “Lysosomal Zn^2+^ Release Triggers Rapid, Mitochondria‐Mediated, Non‐Apoptotic Cell Death in Metastatic Melanoma.” Cell Reports 37: 109848. 10.1016/j.celrep.2021.109848 34686351 PMC8559338

[61] Maret, Wolfgang . 2022. “The Quintessence of Metallomics: a Harbinger of a Different Life Science Based on the Periodic Table of the Bioelements.” Metallomics 14: mfac051. 10.1093/mtomcs/mfac051 35820043 PMC9406523

[62] Tkachenko, Anton , and Anatolii Onishchenko . 2023. “Zincoptosis: Does It Exist?” Apoptosis 28: 681–682. 10.1007/s10495-023-01836-2 36961571

[63] Chen, Liyun , Junxia Min , and Fudi Wang . 2022. “Copper Homeostasis and Cuproptosis in Health and Disease.” Signal Transduction and Targeted Therapy 7: 378. 10.1038/s41392-022-01229-y 36414625 PMC9681860

[64] Machesky, Laura M . 2023. “Deadly Actin Collapse by Disulfidptosis.” Nature Cell Biology 25: 375–376. 10.1038/s41556-023-01100-4 36918690

[65] Mohanty, Ayeskanta , Adityanarayan Mohapatra , Woojin Yang , Seunghyun Choi , Aravindkumar Sundaram , Yong‐Yeon Jeong , Chang‐Moon Lee , Jiwon Seo , and In‐Kyu Park . 2025. “Programable Prodrug Nanomodulator Targets Tumor Redox Homeostasis Imbalance to Amplify Disulfidptosis and Immunogenic Pyroptosis for Breast Tumor Immunotherapy.” Advanced Healthcare Materials 14: e2500272. 10.1002/adhm.202500272 40109062

[66] Freitas, Florencio Porto , Hamed Alborzinia , Ancély Ferreira dos Santos , Palina Nepachalovich , Lohans Pedrera , Omkar Zilka , Alex Inague , et al. 2024. “7‐Dehydrocholesterol Is an Endogenous Suppressor of Ferroptosis.” Nature 626: 401–410. 10.1038/s41586-023-06878-9 38297129

[67] MouYanhua , Jun Wang , Jinchun Wu , Dan He , Chunfang Zhang , Chaojun Duan , and Bin Li . 2019. “Ferroptosis, a New Form of Cell Death: Opportunities and Challenges in Cancer.” Journal of Hematology & Oncology 12: 34. 10.1186/s13045-019-0720-y 30925886 PMC6441206

[68] Liao, Peng , Weimin Wang , Weichao Wang , Ilona Kryczek , Xiong Li , Yingjie Bian , Amanda Sell , et al. 2022. “CD^8^ ^+^ T Cells and Fatty Acids Orchestrate Tumor Ferroptosis and Immunity via ACSL4.” Cancer Cell 40: 365–378.e6. 10.1016/j.ccell.2022.02.003 35216678 PMC9007863

[69] Zhang, Hai‐Liang , Bing‐Xin Hu , Zhi‐Ling Li , Tian Du , Jia‐Lu Shan , Zhi‐Peng Ye , Xiao‐Dan Peng , et al. 2022. “PKCβII Phosphorylates ACSL4 to Amplify Lipid Peroxidation to Induce Ferroptosis.” Nature Cell Biology 24: 88–98. 10.1038/s41556-021-00818-3 35027735

[70] Liang, Deguang , Yan Feng , Fereshteh Zandkarimi , Hua Wang , Zeda Zhang , Jinnie Kim , Yanyan Cai , et al. 2023. “Ferroptosis Surveillance Independent of GPX4 and Differentially Regulated by Sex Hormones.” Cell 186: 2748–2764.e2722. 10.1016/j.cell.2023.05.003 37267948 PMC10330611

[71] Zhang, Yilei , Jiejun Shi , Xiaoguang Liu , Li Feng , Zihua Gong , Pranavi Koppula , Kapil Sirohi , et al. 2018. “BAP1 Links Metabolic Regulation of Ferroptosis to Tumour Suppression.” Nature Cell Biology 20: 1181–1192. 10.1038/s41556-018-0178-0 30202049 PMC6170713

[72] Jiang, Le , Ning Kon , Tongyuan Li , Shang‐Jui Wang , Tao Su , Hanina Hibshoosh , Richard Baer , and Wei Gu . 2015. “FerropWang, Zhiyuan Zhang, andtosis as a p53‐Mediated Activity during Tumour Suppression.” Nature 520: 57–62. 10.1038/nature14344 25799988 PMC4455927

[73] Alavian, Kambiz N. , Gisela Beutner , Emma Lazrove , Silvio Sacchetti , Han‐ A. Park , Pawel Licznerski , Hongmei Li , et al. 2014. “An Uncoupling Channel Within the C‐Subunit Ring of the F1FO ATP Synthase Is the Mitochondrial Permeability Transition Pore.” Proceedings of the National Academy of Sciences 111: 10580–10585. 10.1073/pnas.1401591111 PMC4115574 24979777

[74] Wang, Jinxiang , Qin Tian , Yuchen Liu , Chao‐Yun Cai , Shuying Fu , Jia Li , Yupeng Guan , et al. 2025. “Targeting Metalloptosis in Tumor Therapy: from Molecular Mechanisms to Application of Metal Nanoparticles.” Molecular Cancer 24: 260. 10.1186/s12943-025-02414-7 41094656 PMC12529859

[75] Diessl, Jutta , Jens Berndtsson , Filomena Broeskamp , Lukas Habernig , Verena Kohler , Carmela Vazquez‐Calvo , Arpita Nandy , et al. 2022. “Manganese‐Driven CoQ Deficiency.” Nature Communications 13: 6061. 10.1038/s41467-022-33641-x PMC9563070 36229432

[76] Yan, Bo , Youwei Ai , Qi Sun , Yan Ma , Yang Cao , Jiawen Wang , Zhiyuan Zhang , and Xiaodong Wang . 2021. “Membrane Damage during Ferroptosis Is Caused by Oxidation of Phospholipids Catalyzed by the Oxidoreductases POR and CYB5R1.” Molecular Cell 81: 355–369.e10. 10.1016/j.molcel.2020.11.024 33321093

[77] Quarato, Giovanni , Fabien Llambi , Cliff S. Guy , Jaeki Min , Marisa Actis , Huan Sun , Shilpa Narina , et al. 2022. “Ca^2+^‐mediated Mitochondrial Inner Membrane Permeabilization Induces Cell Death Independently of Bax and Bak.” Cell Death & Differentiation 29: 1318–1334. 10.1038/s41418-022-01025-9 35726022 PMC9287385

[78] Fu, Wan , Jianghuang Wang , Tianyu Li , Yuhui Qiao , Zili Zhang , Xiaomin Zhang , Mingkai He , et al. 2025. “Persistent Activation of TRPM4 Triggers Necrotic Cell Death Characterized by Sodium Overload.” Nature Chemical Biology 21: 1238–1249. 10.1038/s41589-025-01841-3 39915626

[79] Corradi, Francesco , Luca Paolini , and Raffaele De Caterina . 2014. “Ranolazine in the Prevention of Anthracycline Cardiotoxicity.” Pharmacological Research 79: 88–102. 10.1016/j.phrs.2013.11.001 24269342

[80] Sun, Lianhui , Yuan Zhang , Boyu Yang , Sijun Sun , Pengshan Zhang , Zai Luo , Tingting Feng , et al. 2023. “Lactylation of METTL16 Promotes Cuproptosis Via m(6)A‐Modification on FDX1 mRNA in Gastric Cancer.” Nature Communications 14: 6523. 10.1038/s41467-023-42025-8 PMC10589265 37863889

[81] Garbincius, Joanne F. , Oniel Salik , Henry M. Cohen , Carmen Choya‐Foces , Adam S. Mangold , Angelina D. Makhoul , Anna E. Schmidt , et al. 2025. “TMEM65 Regulates and Is Required for NCLX‐Dependent Mitochondrial Calcium Efflux.” Nature Metabolism 7: 714–729. 10.1038/s42255-025-01250-9 PMC12087536 40200126

[82] Yin, Zhao , Guangchao Li , Qi Zhong , Xiaoting Zhang , Ruiming Ou , Huijuan Shen , Jing Huang , et al. 2025. “Cinobufagin Overcomes Bortezomib Resistance in Multiple Myeloma Strains by Targeting SEC. 62/TRPM4‐Mediated NECSO.” Phytomedicine 147: 157171. 10.1016/j.phymed.2025.157171 40839992

[83] Zhao, Quanwei , Hui Li , Danan Liu , Bo Zhou , Caiwei Gong , Long Chen , and Fujun Liao . 2025. “The SGLT2 Inhibitor Dapagliflozin Suppresses Endothelial Cell Pyroptosis Mediated by the NF‐κB/NLRP3 Pathway Through Downregulation of CTSB.” Biochemical Pharmacology 236: 116857. 10.1016/j.bcp.2025.116857 40058708

[84] Brown, Caitlin W. , John J. Amante , Peter Chhoy , Ameer L. Elaimy , Haibo Liu , Lihua Julie Zhu , Christina E. Baer , Scott J. Dixon , and Arthur M. Mercurio . 2019. “Prominin2 Drives Ferroptosis Resistance by Stimulating Iron Export.” Developmental Cell 51: 575–586.e4. 10.1016/j.devcel.2019.10.007 31735663 PMC8316835

[85] Assali, Essam A. , Anthony E. Jones , Michaela Veliova , Rebeca Acín‐Pérez , Mahmoud Taha , Nathanael Miller , Michaël Shum , et al. 2020. “NCLX Prevents Cell Death During Adrenergic Activation of the Brown Adipose Tissue.” Nature Communications 11: 3347. 10.1038/s41467-020-16572-3 PMC7334226 32620768

[86] Wettmarshausen, Jennifer , Valerie Goh , Kai‐Ting Huang , Daniela M. Arduino , Utkarsh Tripathi , Anja Leimpek , Yiming Cheng , et al. 2018. “MICU1 Confers Protection From MCU‐Dependent Manganese Toxicity.” Cell Reports 25: 1425–1435. 10.1016/j.celrep.2018.10.037 30403999

[87] Sun, Shumin , Enjun Xie , Shan Xu , Suyu Ji , Shufen Wang , Jie Shen , Rong Wang , et al. 2024. “The Intestinal Transporter SLC30A1 Plays a Critical Role in Regulating Systemic Zinc Homeostasis.” Advanced Science 11: e2406421. 10.1002/advs.202406421 39422023 PMC11633486

[88] Gurol, Kerem C. , Danyang Li , Karin Broberg , and Somshuvra Mukhopadhyay . 2023. “Manganese Efflux Transporter SLC30A10 Missense Polymorphism T95I Associated With Liver Injury Retains Manganese Efflux Activity.” American Journal of Physiology‐Gastrointestinal and Liver Physiology 324: G78–G88. 10.1152/ajpgi.00213.2022 36414535 PMC9829465

[89] Liu, Quanhai , Jiawei Lai , Jiancang Ma , and Fangshi Xu . 2026. “Necrosis by Sodium Overload: A Potential Mechanism for Renal Diseases Associated with Mitochondrial Dysfunction.” Cell Death Discovery 12: 226. 10.1038/s41420-026-03111-0 41963293 PMC13184122

[90] Fang, Kwang‐Ming , An‐Sheng Lee , Ming‐Jai Su , Chien‐Liang Lin , Chung‐Liang Chien , and Mei‐Lin Wu . 2008. “Free Fatty Acids Act as Endogenous Ionophores, Resulting in Na^+^ and Ca^2+^ Influx and Myocyte Apoptosis.” Cardiovascular Research 78: 533–545. 10.1093/cvr/cvn030 18267958

[91] Abuarab, Nada , Tim S. Munsey , Lin‐Hua Jiang , Jing Li , and Asipu Sivaprasadarao . 2017. “High Glucose‐Induced ROS Activates TRPM2 to Trigger Lysosomal Membrane Permeabilization and Zn^2+^‐Mediated Mitochondrial Fission.” Science Signaling 10: eaal4161. 10.1126/scisignal.aal4161 28765513

[92] Qiu, Baiyu , Fereshteh Zandkarimi , Carla T. Bezjian , Eduard Reznik , Rajesh Kumar Soni , Wei Gu , Xuejun Jiang , and Brent R. Stockwell . 2024. “Phospholipids With Two Polyunsaturated Fatty Acyl Tails Promote Ferroptosis.” Cell 187: 1177–1190. 10.1016/j.cell.2024.01.030 38366593 PMC10940216

[93] Ludlow, Melanie J. , Hannah J. Gaunt , Hussein N. Rubaiy , Katie E. Musialowski , Nicola M. Blythe , Naveen S. Vasudev , Katsuhiko Muraki , and David J. Beech . 2017. “(−)‐Englerin A‐Evoked Cytotoxicity Is Mediated by Na^+^ Influx and Counteracted by Na^+^/K^+^‐ATPase.” Journal of Biological Chemistry 292: 723–731. 10.1074/jbc.M116.755678 27875305 PMC5241745

[94] Shimada, Kenichi , Rachid Skouta , Anna Kaplan , Wan Seok Yang , Miki Hayano , Scott J. Dixon , Lewis M. Brown , et al. 2016. “Global Survey of Cell Death Mechanisms Reveals Metabolic Regulation of Ferroptosis.” Nature Chemical Biology 12: 497–503. 10.1038/nchembio.2079 27159577 PMC4920070

[95] Liang, Chen , Xinglin Zhang , Mengsu Yang , and Xiaochen Dong . 2019. “Recent Progress in Ferroptosis Inducers for Cancer Therapy.” Advanced Materials 31: e1904197. 10.1002/adma.201904197 31595562

[96] Zhang, Ping , Chaoting Zhou , Xueying Ren , Qiangan Jing , Yan Gao , Chen Yang , Yuhuan Shen , et al. 2024. “Inhibiting the Compensatory Elevation of xCT Collaborates With Disulfiram/Copper‐Induced GSH Consumption for Cascade Ferroptosis and Cuproptosis.” Redox Biology 69: 103007. 10.1016/j.redox.2023.103007 38150993 PMC10788306

[97] Li, Jing , Binbin Ding , Jia Tan , Hao Chen , Qi Meng , Xinyang Li , Pan Zheng , Ping'an Ma , and Jun Lin . 2023. “Sodium Citrate Nanoparticles Induce Dual‐Path Pyroptosis for Enhanced Antitumor Immunotherapy Through Synergistic Ion Overload and Metabolic Disturbance.” Nano Letters 23: 10034–10043. 10.1021/acs.nanolett.3c03382 37903236

[98] Zhang, Jun , Xia Qin , Bin Wang , Ge Xu , Zhexue Qin , Jian Wang , Lanxiang Wu , et al. 2017. “Zinc Oxide Nanoparticles Harness Autophagy to Induce Cell Death in Lung Epithelial Cells.” Cell Death & Disease 8: e2954. 10.1038/cddis.2017.337 28749469 PMC5550878

[99] Miotto, Giovanni , Monica Rossetto , Maria Luisa Di Paolo , Laura Orian , Rina Venerando , Antonella Roveri , Ana‐Marija Vučković , et al. 2020. “Insight Into the Mechanism of Ferroptosis Inhibition by Ferrostatin‐1.” Redox Biology 28: 101328. 10.1016/j.redox.2019.101328 31574461 PMC6812032

[100] Teshima, Yasushi , Masaharu Akao , Steven P. Jones , and Eduardo Marbán . 2003. “Cariporide (HOE642), a Selective Na^+^‐H^+^ Exchange Inhibitor, Inhibits the Mitochondrial Death Pathway.” Circulation 108: 2275–2281. 10.1161/01.CIR.0000093277.20968.C7 14568900

[101] Ru, Qin , Yusheng Li , Lin Chen , Yuxiang Wu , Junxia Min , and Fudi Wang . 2024. “Iron Homeostasis and Ferroptosis in Human Diseases: Mechanisms and Therapeutic Prospects.” Signal Transduction and Targeted Therapy 9: 271. 10.1038/s41392-024-01969-z 39396974 PMC11486532

[102] Peng, Dongjie , Junyan Li , Yue Deng , Xiaojuan Zhu , Lin Zhao , Yuwen Zhang , Zhaocong Li , et al. 2020. “Sodium Para‐Aminosalicylic Acid Inhibits Manganese‐Induced NLRP3 Inflammasome‐Dependent Pyroptosis by Inhibiting NF‐κB Pathway Activation and Oxidative Stress.” Journal of Neuroinflammation 17: 343. 10.1186/s12974-020-02018-6 33203418 PMC7670624

[103] Wang, Weimin , Michael Green , Jae Eun Choi , Miguel Gijón , Paul D. Kennedy , Jeffrey K. Johnson , Peng Liao , et al. 2019. “CD8(+) T Cells Regulate Tumour Ferroptosis During Cancer Immunotherapy.” Nature 569: 270–274. 10.1038/s41586-019-1170-y 31043744 PMC6533917

[104] Dong, Xian‐Ping , Xiping Cheng , Eric Mills , Markus Delling , Fudi Wang , Tino Kurz , and Haoxing Xu . 2008. “The Type IV Mucolipidosis‐Associated Protein TRPML1 Is an Endolysosomal Iron Release Channel.” Nature 455: 992–996. 10.1038/nature07311 18794901 PMC4301259

[105] Yu, Yingying , Li Jiang , Hao Wang , Zhe Shen , Qi Cheng , Pan Zhang , Jiaming Wang , et al. 2020. “Hepatic Transferrin Plays a Role in Systemic Iron Homeostasis and Liver Ferroptosis.” Blood 136: 726–739. 10.1182/blood.2019002907 32374849 PMC7414596

[106] Tao, Liang , Xinquan Yang , Chaodong Ge , Peng Zhang , Wenjian He , Xingbo Xu , Xin Li , et al. 2024. “Integrative Clinical and Preclinical Studies Identify FerroTerminator1 as a Potent Therapeutic Drug for MASH.” Cell Metabolism 36: 2190–2206.e5. 10.1016/j.cmet.2024.07.013 39142286

[107] Sun, Xiaofang , Zhanhui Ou , Ruochan Chen , Xiaohua Niu , De Chen , Rui Kang , and Daolin Tang . 2016. “Activation of the p62‐Keap1‐NRF2 Pathway Protects Against Ferroptosis in Hepatocellular Carcinoma Cells.” Hepatology 63: 173–184. 10.1002/hep.28251 26403645 PMC4688087

[108] Fang, Xuexian , Hao Wang , Dan Han , Enjun Xie , Xiang Yang , Jiayu Wei , Shanshan Gu , et al. 2019. “Ferroptosis as a Target for Protection Against Cardiomyopathy.” Proceedings of the National Academy of Sciences 116: 2672–2680. 10.1073/pnas.1821022116 PMC6377499 30692261

[109] Fang, Xuexian , Zhaoxian Cai , Hao Wang , Dan Han , Qi Cheng , Pan Zhang , Feng Gao , et al. 2020. “Loss of Cardiac Ferritin H Facilitates Cardiomyopathy via Slc7a11‐Mediated Ferroptosis.” Circulation Research 127: 486–501. 10.1161/CIRCRESAHA.120.316509 32349646

[110] Liu, Qian , Fengli Wang , Yingxian Chen , Hengkang Cui , and Hao Wu . 2024. “A Regulatory Module Comprising G3BP1‐FBXL5‐IRP2 Axis Determines Sodium Arsenite‐Induced Ferroptosis.” Journal of Hazardous Materials 465: 133038. 10.1016/j.jhazmat.2023.133038 38118197

[111] Alvarez, Samantha W. , Vladislav O. Sviderskiy , Erdem M. Terzi , Thales Papagiannakopoulos , Andre L. Moreira , Sylvia Adams , David M. Sabatini , Kıvanç Birsoy , and Richard Possemato . 2017. “NFS1 Undergoes Positive Selection in Lung Tumours and Protects Cells From Ferroptosis.” Nature 551: 639–643. 10.1038/nature24637 29168506 PMC5808442

[112] Zhang, Zili , Zhen Yao , Ling Wang , Hai Ding , Jiangjuan Shao , Anping Chen , Feng Zhang , and Shizhong Zheng . 2018. “Activation of Ferritinophagy Is Required for the RNA‐Binding Protein ELAVL1/HuR to Regulate Ferroptosis in Hepatic Stellate Cells.” Autophagy 14: 2083–2103. 10.1080/15548627.2018.1503146 30081711 PMC6984765

[113] Zhang, Zili , Mei Guo , Yujia Li , Min Shen , Desong Kong , Jiangjuan Shao , Hai Ding , et al. 2020. “RNA‐Binding Protein ZFP36/TTP Protects Against Ferroptosis by Regulating Autophagy Signaling Pathway in Hepatic Stellate Cells.” Autophagy 16: 1482–1505. 10.1080/15548627.2019.1687985 31679460 PMC7469536

[114] Bao, Wen‐Dai , Pei Pang , Xiao‐Ting Zhou , Fan Hu , Wan Xiong , Kai Chen , Jing Wang , et al. 2021. “Loss of Ferroportin Induces Memory Impairment by Promoting Ferroptosis in Alzheimer's Disease.” Cell Death & Differentiation 28: 1548–1562. 10.1038/s41418-020-00685-9 33398092 PMC8166828

[115] Meng, Hongen , Yingying Yu , Enjun Xie , Qian Wu , Xiangju Yin , Bin Zhao , Junxia Min , and Fudi Wang . 2023. “Hepatic HDAC3 Regulates Systemic Iron Homeostasis and Ferroptosis via the Hippo Signaling Pathway.” Research 6: 0281. 10.34133/research.0281 38034086 PMC10687581

[116] Zou, Yilong , Whitney S. Henry , Emily L. Ricq , Emily T. Graham , Vaishnavi V. Phadnis , Pema Maretich , Sateja Paradkar , et al. 2020. “Plasticity of Ether Lipids Promotes Ferroptosis Susceptibility and Evasion.” Nature 585: 603–608. 10.1038/s41586-020-2732-8 32939090 PMC8051864

[117] Yang, Wan Seok , Katherine J. Kim , Michael M. Gaschler , Milesh Patel , Mikhail S. Shchepinov , and Brent R. Stockwell . 2016. “Peroxidation of Polyunsaturated Fatty Acids by Lipoxygenases Drives Ferroptosis.” Proceedings of the National Academy of Sciences 113: E4966–E4975. 10.1073/pnas.1603244113 PMC5003261 27506793

[118] Zou, Yilong , Haoxin Li , Emily T. Graham , Amy A. Deik , John K. Eaton , Wenyu Wang , Gerardo Sandoval‐Gomez , et al. 2020. “Cytochrome P450 Oxidoreductase Contributes to Phospholipid Peroxidation in Ferroptosis.” Nature Chemical Biology 16: 302–309. 10.1038/s41589-020-0472-6 32080622 PMC7353921

[119] Zhang, Yilei , Robert V. Swanda , Litong Nie , Xiaoguang Liu , Chao Wang , Hyemin Lee , Guang Lei , et al. 2021. “mTORC1 Couples Cyst(e)Ine Availability With GPX4 Protein Synthesis and Ferroptosis Regulation.” Nature Communications 12: 1589. 10.1038/s41467-021-21841-w PMC7952727 33707434

[120] Mishima, Eikan , Junya Ito , Zijun Wu , Toshitaka Nakamura , Adam Wahida , Sebastian Doll , Wulf Tonnus , et al. 2022. “A Non‐Canonical Vitamin K Cycle Is a Potent Ferroptosis Suppressor.” Nature 608: 778–783. 10.1038/s41586-022-05022-3 35922516 PMC9402432

[121] Soula, Mariluz , Ross A. Weber , Omkar Zilka , Hanan Alwaseem , Konnor La , Frederick Yen , Henrik Molina , et al. 2020. “Metabolic Determinants of Cancer Cell Sensitivity to Canonical Ferroptosis Inducers.” Nature Chemical Biology 16: 1351–1360. 10.1038/s41589-020-0613-y 32778843 PMC8299533

[122] Sun, Wan‐Yang , Vladimir A. Tyurin , Karolina Mikulska‐Ruminska , Indira H. Shrivastava , Tamil S. Anthonymuthu , Yu‐Jia Zhai , Ming‐Hai Pan , et al. 2021. “Phospholipase iPLA2β Averts Ferroptosis by Eliminating a Redox Lipid Death Signal.” Nature Chemical Biology 17: 465–476. 10.1038/s41589-020-00734-x 33542532 PMC8152680

[123] Koppula, Pranavi , Guang Lei , Yilei Zhang , Yuelong Yan , Chao Mao , Lavanya Kondiparthi , Jiejun Shi , et al. 2022. “A Targetable CoQ‐FSP1 Axis Drives Ferroptosis‐ and Radiation‐Resistance in KEAP1 Inactive Lung Cancers.” Nature Communications 13: 2206. 10.1038/s41467-022-29905-1 PMC9033817 35459868

[124] Gao, Minghui , Prashant Monian , Qiuhui Pan , Wei Zhang , Jenny Xiang , and Xuejun Jiang . 2016. “Ferroptosis Is an Autophagic Cell Death Process.” Cell Research 26: 1021–1032. 10.1038/cr.2016.95 27514700 PMC5034113

[125] Chen, Xin , Rui Kang , Guido Kroemer , and Daolin Tang . 2021. “Broadening Horizons: the Role of Ferroptosis in Cancer.” Nature Reviews Clinical Oncology 18: 280–296. 10.1038/s41571-020-00462-0 33514910

[126] Chen, Xin , Jingbo Li , Rui Kang , Daniel J. Klionsky , and Daolin Tang . 2021. “Ferroptosis: Machinery and Regulation.” Autophagy 17: 2054–2081. 10.1080/15548627.2020.1810918 32804006 PMC8496712

[127] Galy, Bruno , Marcus Conrad , and Martina Muckenthaler . 2024. “Mechanisms Controlling Cellular and Systemic Iron Homeostasis.” Nature Reviews Molecular Cell Biology 25: 133–155. 10.1038/s41580-023-00648-1 37783783

[128] Xu, Tian , Yuying Ma , Qinling Yuan , Huixin Hu , Xinkai Hu , Zhiyu Qian , Janiqua Kyiesha Rolle , Yueqing Gu , and Siwen Li . 2020. “Enhanced Ferroptosis by Oxygen‐Boosted Phototherapy Based on a 2‐in‐1 Nanoplatform of Ferrous Hemoglobin for Tumor Synergistic Therapy.” ACS Nano 14: 3414–3425. 10.1021/acsnano.9b09426 32155051

[129] Jiang, Qin , Kuang Wang , Xingyu Zhang , Boshu Ouyang , Haixia Liu , Zhiqing Pang , and Wuli Yang . 2020. “Platelet Membrane‐Camouflaged Magnetic Nanoparticles for Ferroptosis‐Enhanced Cancer Immunotherapy.” Small 16: e2001704. 10.1002/smll.202001704 32338436

[130] Friedmann Angeli, José Pedro , Dmitri V. Krysko , and Marcus Conrad . 2019. “Ferroptosis at the Crossroads of Cancer‐Acquired Drug Resistance and Immune Evasion.” Nature Reviews Cancer 19: 405–414. 10.1038/s41568-019-0149-1 31101865

[131] Hassannia, Behrouz , Peter Vandenabeele , and Tom Vanden Berghe . 2019. “Targeting Ferroptosis to Iron out Cancer.” Cancer Cell 35: 830–849. 10.1016/j.ccell.2019.04.002 31105042

[132] Huo, Minfeng , Liying Wang , Youwei Wang , Yu Chen , and Jianlin Shi . 2019. “Nanocatalytic Tumor Therapy by Single‐Atom Catalysts.” ACS Nano 13: 2643–2653. 10.1021/acsnano.9b00457 30753056

[133] Liu, Chang , Xiaoyu Xu , Yongyang Chen , Miao Yin , Ermei Mäkilä , Wenhui Zhou , Wenmei Su , and Hongbo Zhang . 2024. “Metabolism‐Regulating Nanozyme System for Advanced Nanocatalytic Cancer Therapy.” Small 20: e2307794. 10.1002/smll.202307794 38168483

[134] Xie, Lisi , Jie Li , Guohao Wang , Wei Sang , Mengze Xu , Wenxi Li , Jie Yan , et al. 2022. “Phototheranostic Metal‐Phenolic Networks With Antiexosomal PD‐L1 Enhanced Ferroptosis for Synergistic Immunotherapy.” Journal of the American Chemical Society 144: 787–797. 10.1021/jacs.1c09753 34985903

[135] Badgley, Michael A. , Daniel M. Kremer , H. Carlo Maurer , Kathleen E. DelGiorno , Ho‐Joon Lee , Vinee Purohit , Irina R. Sagalovskiy , et al. 2020. “Cysteine Depletion Induces Pancreatic Tumor Ferroptosis in Mice.” Science 368: 85–89. 10.1126/science.aaw9872 32241947 PMC7681911

[136] Stockwell, Brent R . 2022. “Ferroptosis Turns 10: Emerging Mechanisms, Physiological Functions, and Therapeutic Applications.” Cell 185: 2401–2421. 10.1016/j.cell.2022.06.003 35803244 PMC9273022

[137] Lin, Jin‐Fei , Pei‐Shan Hu , Yi‐Yu Wang , Yue‐Tao Tan , Kai Yu , Kun Liao , Qi‐Nian Wu , et al. 2022. “Phosphorylated NFS1 Weakens Oxaliplatin‐Based Chemosensitivity of Colorectal Cancer by Preventing PANoptosis.” Signal Transduction and Targeted Therapy 7: 54. 10.1038/s41392-022-00889-0 35221331 PMC8882671

[138] Dalton, Timothy P. , Lei He , Bin Wang , Marian L. Miller , Li Jin , Keith F. Stringer , Xiaoqing Chang , C. Stuart Baxter , and Daniel W. Nebert . 2005. “Identification of Mouse SLC39A8 As the Transporter Responsible for Cadmium‐Induced Toxicity in the Testis.” Proceedings of the National Academy of Sciences 102: 3401–3406. 10.1073/pnas.0406085102 PMC552906 15722412

[139] Chen, Peng , Qibiao Wu , Jiao Feng , Lili Yan , Yitian Sun , Shuiping Liu , Yu Xiang , et al. 2020. “Erianin, a Novel Dibenzyl Compound in Dendrobium Extract, Inhibits Lung Cancer Cell Growth and Migration via Calcium/Calmodulin‐Dependent Ferroptosis.” Signal Transduction and Targeted Therapy 5: 51. 10.1038/s41392-020-0149-3 32382060 PMC7205607

[140] Müller, Sebastian , Fabien Sindikubwabo , Tatiana Cañeque , Anne Lafon , Antoine Versini , Bérangère Lombard , Damarys Loew , et al. 2020. “CD44 Regulates Epigenetic Plasticity by Mediating Iron Endocytosis.” Nature Chemistry 12: 929–938. 10.1038/s41557-020-0513-5 PMC7612580 32747755

[141] Cañeque, Tatiana , Leeroy Baron , Sebastian Müller , Alanis Carmona , Ludovic Colombeau , Antoine Versini , Stéphanie Solier , et al. 2025. “Activation of Lysosomal Iron Triggers Ferroptosis in Cancer.” Nature 642: 492–500. 10.1038/s41586-025-08974-4 40335696 PMC12158755

[142] Shi, Haifeng , Krisztina Z. Bencze , Timothy L. Stemmler , and Caroline C. Philpott . 2008. “A Cytosolic Iron Chaperone That Delivers Iron to Ferritin.” Science 320: 1207–1210. 10.1126/science.1157643 18511687 PMC2505357

[143] Ryu, Moon‐Suhn , Deliang Zhang , Olga Protchenko , Minoo Shakoury‐Elizeh , and Caroline C. Philpott . 2017. “PCBP1 and NCOA4 Regulate Erythroid Iron Storage and Heme Biosynthesis.” The Journal of Clinical Investigation 127: 1786–1797. 10.1172/JCI90519 28375153 PMC5409075

[144] Kabeya, Yukiko , Noboru Mizushima , Takashi Ueno , Akitsugu Yamamoto , Takayoshi Kirisako , Takeshi Noda , Eiki Kominami , Yoshinori Ohsumi , and Tamotsu Yoshimori . 2000. “LC3, a Mammalian Homologue of Yeast Apg8p, Is Localized in Autophagosome Membranes after Processing.” The EMBO Journal 19: 5720–5728. 10.1093/emboj/19.21.5720 11060023 PMC305793

[145] Yu, Fan , Qianping Zhang , Hanyu Liu , Jinming Liu , Song Yang , Xiaofan Luo , Wei Liu , et al. 2022. “Dynamic O‐GlcNAcylation Coordinates Ferritinophagy and Mitophagy to Activate Ferroptosis.” Cell Discovery 8: 40. 10.1038/s41421-022-00390-6 35504898 PMC9065108

[146] Fuhrmann, Dominik C. , Antonia Mondorf , Josefine Beifuß , Michaela Jung , and Bernhard Brüne . 2020. “Hypoxia Inhibits Ferritinophagy, Increases Mitochondrial Ferritin, and Protects From Ferroptosis.” Redox Biology 36: 101670. 10.1016/j.redox.2020.101670 32810738 PMC7452134

[147] Zhang, Xiao‐Yi , Yan Luo , Nan‐Nan Liang , Qiang‐Sheng Li , Zhi‐Hui Zhang , Yi‐Chao Huang , and De‐Xiang Xu . 2026. “Arsenic Exposure Reduces Testosterone Synthesis Partially by Evoking Leydig Cell Ferroptosis in Mouse Testes.” Environmental Pollution 390: 127547. 10.1016/j.envpol.2025.127547 41412458

[148] Mai, Trang Thi , Ahmed Hamaï , Antje Hienzsch , Tatiana Cañeque , Sebastian Müller , Julien Wicinski , Olivier Cabaud , et al. 2017. “Salinomycin Kills Cancer Stem Cells by Sequestering Iron in Lysosomes.” Nature Chemistry 9: 1025–1033. 10.1038/nchem.2778 PMC5890907 28937680

[149] Hassannia, Behrouz , Bartosz Wiernicki , Irina Ingold , Feng Qu , Simon Van Herck , Yulia Y. Tyurina , Hülya Bayır , et al. 2018. “Nano‐Targeted Induction of Dual Ferroptotic Mechanisms Eradicates High‐Risk Neuroblastoma.” The Journal of Clinical Investigation 128: 3341–3355. 10.1172/JCI99032 29939160 PMC6063467

[150] Dodson, Matthew , Raul Castro‐Portuguez , and Donna D. Zhang . 2019. “NRF2 Plays a Critical Role in Mitigating Lipid Peroxidation and Ferroptosis.” Redox Biology 23: 101107. 10.1016/j.redox.2019.101107 30692038 PMC6859567

[151] Zhi, Hui , Weimin Yin , Shiyu Chen , Xiaoyou Zhang , Zichen Yang , Fulong Man , Rongjie Li , et al. 2026. “Lactate Metabolism Regulating Nanosystem Synergizes Cuproptosis and Ferroptosis to Enhance Cancer Immunotherapy.” Biomaterials 325: 123538. 10.1016/j.biomaterials.2025.123538 40680708

[152] Yao, Fan , Yalan Deng , Yang Zhao , Ying Mei , Yilei Zhang , Xiaoguang Liu , Consuelo Martinez , et al. 2021. “A Targetable LIFR−NF‐κB−LCN2 Axis Controls Liver Tumorigenesis and Vulnerability to Ferroptosis.” Nature Communications 12: 7333. 10.1038/s41467-021-27452-9 PMC8683481 34921145

[153] Liu, Jiao , Xinxin Song , Feimei Kuang , Qiuhong Zhang , Yangchun Xie , Rui Kang , Guido Kroemer , and Daolin Tang . 2021. “NUPR1 Is a Critical Repressor of Ferroptosis.” Nature Communications 12: 647. 10.1038/s41467-021-20904-2 PMC7843652 33510144

[154] Yuan, Hua , Xuemei Li , Xiuying Zhang , Rui Kang , and Daolin Tang . 2016. “CISD1 Inhibits Ferroptosis by Protection against Mitochondrial Lipid Peroxidation.” Biochemical and Biophysical Research Communications 478: 838–844. 10.1016/j.bbrc.2016.08.034 27510639

[155] Zeng, Jin , Zuyun Yan , Dong Wang , Tao He , Zhaochen Tong , Jinglei Miao , Jinsong Li , et al. 2025. “Mitochondria‐Targeted MXene@MnO(2)‐TPP Nanoheterostructures for Synergistic Enhancement of Sonodynamic Therapy and Immunotherapy in Osteosarcoma.” Bioactive Materials 54: 450–465. 10.1016/j.bioactmat.2025.08.029 40918737 PMC12410470

[156] Vaishampayan, Prajakta , and Yool Lee . 2024. “Redox‐Active Vitamin C Suppresses Human Osteosarcoma Growth by Triggering Intracellular ROS‐iron‐calcium Signaling Crosstalk and Mitochondrial Dysfunction.” Redox Biology 75: 103288. 10.1016/j.redox.2024.103288 39083898 PMC11342202

[157] Yang, Xiaoyan , Seong‐Hoon Park , Hsiang‐Chun Chang , Jason S. Shapiro , Athanassios Vassilopoulos , Konrad T. Sawicki , Chunlei Chen , et al. 2017. “Sirtuin 2 Regulates Cellular Iron Homeostasis via Deacetylation of Transcription Factor NRF2.” The Journal of Clinical Investigation 127: 1505–1516. 10.1172/JCI88574 28287409 PMC5373873

[158] Anandhan, Annadurai , Matthew Dodson , Aryatara Shakya , Jinjing Chen , Pengfei Liu , Yongyi Wei , Hui Tan , et al. 2023. “NRF2 Controls Iron Homeostasis and Ferroptosis Through HERC2 and VAMP8.” Science Advances 9: eade9585. 10.1126/sciadv.ade9585 36724221 PMC9891695

[159] Yang, Mi , Xixi Wu , Jinlong Hu , Yingqiao Wang , Yin Wang , Longshan Zhang , Weiqiang Huang , et al. 2022. “COMMD10 Inhibits HIF1α/CP Loop to Enhance Ferroptosis and Radiosensitivity by Disrupting Cu‐Fe Balance in Hepatocellular Carcinoma.” Journal of Hepatology 76: 1138–1150. 10.1016/j.jhep.2022.01.009 35101526

[160] Dierge, Emeline , Elena Debock , Céline Guilbaud , Cyril Corbet , Eric Mignolet , Louise Mignard , Estelle Bastien , et al. 2021. “Peroxidation of n‐3 and n‐6 Polyunsaturated Fatty Acids in the Acidic Tumor Environment Leads to Ferroptosis‐Mediated Anticancer Effects.” Cell Metabolism 33: 1701–1715.e5. 10.1016/j.cmet.2021.05.016 34118189

[161] Liu, Yu'e , Shiping Lu , Lei‐lei Wu , Liang Yang , Lixue Yang , and Jinghan Wang . 2023. “The Diversified Role of Mitochondria in Ferroptosis in Cancer.” Cell Death & Disease 14: 519. 10.1038/s41419-023-06045-y 37580393 PMC10425449

[162] Dixon, Scott J. , and James A. Olzmann . 2024. “The Cell Biology of Ferroptosis.” Nature Reviews Molecular Cell Biology 25: 424–442. 10.1038/s41580-024-00703-5 38366038 PMC12187608

[163] Liu, Lixiao , Zikai Zheng , Wanbang You , Peng Yang , Yifan Wen , Yicheng Qiao , Shuai Ma , et al. 2026. “Vitamin C Inhibits ACSL4 to Alleviate Ferro‐Aging in Primates.” Cell Metabolism 38: 673–693.e17. 10.1016/j.cmet.2026.02.010 41819088

[164] Lin, Zhi , Jiao Liu , Fei Long , Protects Metastasizing Melanoma Rui Kang , Guido Kroemer , Daolin Tang , and Minghua Yang . 2022. “The Lipid Flippase SLC47A1 Blocks Metabolic Vulnerability to Ferroptosis.” Nature Communications 13: 7965. 10.1038/s41467-022-35707-2 PMC9794750 36575162

[165] Kim, Jong Woo , Ji‐Yoon Lee , Mihee Oh , and Eun‐Woo Lee . 2023. “An Integrated View of Lipid Metabolism in Ferroptosis Revisited via Lipidomic Analysis.” Experimental & Molecular Medicine 55: 1620–1631. 10.1038/s12276-023-01077-y 37612411 PMC10474074

[166] Wu, Jiao , Alexander M. Minikes , Minghui Gao , Huijie Bian , Yong Li , Brent R. Stockwell , Zhi‐Nan Chen , and Xuejun Jiang . 2019. “Intercellular Interaction Dictates Cancer Cell Ferroptosis Via NF2‐YAP Signalling.” Nature 572: 402–406. 10.1038/s41586-019-1426-6 31341276 PMC6697195

[167] Wenzel, Sally E. , Yulia Y. Tyurina , Jinming Zhao , Claudette M. St. Croix , Haider H. Dar , Gaowei Mao , Vladimir A. Tyurin , et al. 2017. “PEBP1 Wardens Ferroptosis by Enabling Lipoxygenase Generation of Lipid Death Signals.” Cell 171: 628–641.e26. 10.1016/j.cell.2017.09.044 29053969 PMC5683852

[168] Cai, Wenbin , Le Liu , Xuelian Shi , Yanan Liu , Jin Wang , Xuan Fang , Zhipeng Chen , et al. 2023. “Alox15/15‐HpETE Aggravates Myocardial Ischemia‐Reperfusion Injury by Promoting Cardiomyocyte Ferroptosis.” Circulation 147: 1444–1460. 10.1161/CIRCULATIONAHA.122.060257 36987924

[169] Xie, Yangchun , Shan Zhu , Xinxin Song , Xiaofang Sun , Yong Fan , Jinbao Liu , Meizuo Zhong , et al. 2017. “The Tumor Suppressor p53 Limits Ferroptosis by Blocking DPP4 Activity.” Cell Reports 20: 1692–1704. 10.1016/j.celrep.2017.07.055 28813679

[170] Zilka, Omkar , Ron Shah , Bo Li , José Pedro Friedmann Angeli , Markus Griesser , Marcus Conrad , and Derek A. Pratt . 2017. “On the Mechanism of Cytoprotection by Ferrostatin‐1 and Liproxstatin‐1 and the Role of Lipid Peroxidation in Ferroptotic Cell Death.” ACS Central Science 3: 232–243. 10.1021/acscentsci.7b00028 28386601 PMC5364454

[171] Shah, Ron , Mikhail S. Shchepinov , and Derek A. Pratt . 2018. “Resolving the Role of Lipoxygenases in the Initiation and Execution of Ferroptosis.” ACS Central Science 4: 387–396. 10.1021/acscentsci.7b00589 29632885 PMC5879472

[172] Zhou, Zinan , Alan T. Tang , Weng‐Yew Wong , Sharika Bamezai , Lauren M. Goddard , Robert Shenkar , Su Zhou , et al. 2016. “Cerebral Cavernous Malformations Arise From Endothelial Gain of MEKK3‐KLF2/4 Signalling.” Nature 532: 122–126. 10.1038/nature17178 27027284 PMC4864035

[173] von Krusenstiern, A. Nikolai , Ryan N. Robson , Naixin Qian , Baiyu Qiu , Fanghao Hu , Eduard Reznik , Nailah Smith , et al. 2023. “Identification of Essential Sites of Lipid Peroxidation in Ferroptosis.” Nature Chemical Biology 19: 719–730. 10.1038/s41589-022-01249-3 36747055 PMC10238648

[174] Sassano, Maria Livia , Yulia Y. Tyurina , Antigoni Diokmetzidou , Ellen Vervoort , Vladimir A. Tyurin , Sanket More , Rita La Rovere , et al. 2025. “Endoplasmic Reticulum–Mitochondria Contacts Are Prime Hotspots of Phospholipid Peroxidation Driving Ferroptosis.” Nature Cell Biology 27: 902–917. 10.1038/s41556-025-01668-z 40514428 PMC12173944

[175] Magtanong, Leslie , Pin‐Joe Ko , Milton To , Jennifer Yinuo Cao , Giovanni C. Forcina , Amy Tarangelo , Carl C. Ward , et al. 2019. “Exogenous Monounsaturated Fatty Acids Promote a Ferroptosis‐Resistant Cell State.” Cell Chemical Biology 26: 420–432.e9. 10.1016/j.chembiol.2018.11.016 30686757 PMC6430697

[176] Ubellacker, Jessalyn M. , Alpaslan Tasdogan , Vijayashree Ramesh , Bo Shen , Evann C. Mitchell , Misty S. Martin‐Sandoval , Zhimin Gu , et al. 2020. “Lymph Protects Signaling Suppresses FerroptosisMetastasizing Melanoma Cells From Ferroptosis.” Nature 585: 113–118. 10.1038/s41586-020-2623-z 32814895 PMC7484468

[177] Yi, Junmei , Jiajun Zhu , Jiao Wu , Craig B. Thompson , and Xuejun Jiang . 2020. “Oncogenic Activation of PI3K‐AKT‐mTOR Signaling Suppresses Ferroptosis via SREBP‐mediated Lipogenesis.” Proceedings of the National Academy of Sciences 117: 31189–31197. 10.1073/pnas.2017152117 PMC7733797 33229547

[178] Ashraf, Azhaar , Jérôme Jeandriens , Harold G. Parkes , and Po‐Wah So . 2020. “Iron Dyshomeostasis, Lipid Peroxidation and Perturbed Expression of Cystine/Glutamate Antiporter in Alzheimer's Disease: Evidence of Ferroptosis.” Redox Biology 32: 101494. 10.1016/j.redox.2020.101494 32199332 PMC7083890

[179] Lee, Hyemin , Fereshteh Zandkarimi , Yilei Zhang , Jitendra Kumar Meena , Jongchan Kim , Li Zhuang , Siddhartha Tyagi , et al. 2020. “Energy‐Stress‐Mediated AMPK Activation Inhibits Ferroptosis.” Nature Cell Biology 22: 225–234. 10.1038/s41556-020-0461-8 32029897 PMC7008777

[180] Zou, Yilong , Michael J. Palte , Amy A. Deik , Haoxin Li , John K. Eaton , Wenyu Wang , Yuen‐Yi Tseng , et al. 2019. “A GPX4‐dependent Cancer Cell State Underlies the Clear‐Cell Morphology and Confers Sensitivity to Ferroptosis.” Nature Communications 10: 1617. 10.1038/s41467-019-09277-9 PMC6453886 30962421

[181] Dixon, Scott J. , Georg E. Winter , Leila S. Musavi , Eric D. Lee , Berend Snijder , Manuele Rebsamen , Giulio Superti‐Furga , and Brent R. Stockwell . 2015. “Human Haploid Cell Genetics Reveals Roles for Lipid Metabolism Genes in Nonapoptotic Cell Death.” ACS Chemical Biology 10: 1604–1609. 10.1021/acschembio.5b00245 25965523 PMC4509420

[182] Ingold, Irina , Carsten Berndt , Sabine Schmitt , Sebastian Doll , Gereon Poschmann , Katalin Buday , Antonella Roveri , et al. 2018. “Selenium Utilization by GPX4 Is Required to Prevent Hydroperoxide‐Induced Ferroptosis.” Cell 172: 409–422.e21. 10.1016/j.cell.2017.11.048 29290465

[183] Koppula, Pranavi , Li Zhuang , and Boyi Gan . 2021. “Cystine Transporter SLC7A11/xCT in Cancer: Ferroptosis, Nutrient Dependency, and Cancer Therapy.” Protein & Cell 12: 599–620. 10.1007/s13238-020-00789-5 33000412 PMC8310547

[184] Dixon, Scott J. , Darpan N. Patel , Matthew Welsch , Rachid Skouta , Eric D. Lee , Miki Hayano , Ajit G. Thomas , et al. 2014. “Pharmacological Inhibition of Cystine–Glutamate Exchange Induces Endoplasmic Reticulum Stress and Ferroptosis.” Elife 3: e02523. 10.7554/eLife.02523 24844246 PMC4054777

[185] Ye, Yuanzhi , An Chen , Li Li , Qingchun Liang , Siyi Wang , Qianqian Dong , Mingwei Fu , et al. 2022. “Repression of the Antiporter SLC7A11/Glutathione/Glutathione Peroxidase 4 Axis Drives Ferroptosis of Vascular Smooth Muscle Cells to Facilitate Vascular Calcification.” Kidney International 102: 1259–1275. 10.1016/j.kint.2022.07.034 36063875

[186] Song, Xinxin , Shan Zhu , Pan Chen , Wen Hou , Qirong Wen , Jiao Liu , Yangchun Xie , et al. 2018. “AMPK‐Mediated BECN1 Phosphorylation Promotes Ferroptosis by Directly Blocking System Xc‐ Activity.” Current Biology 28: 2388–2399.e5. 10.1016/j.cub.2018.05.094 30057310 PMC6081251

[187] Xue, Qian , Ding Yan , Xi Chen , Xiaofen Li , Rui Kang , Daniel J. Klionsky , Guido Kroemer , et al. 2023. “Copper‐Dependent Autophagic Degradation of GPX4 Drives Ferroptosis.” Autophagy 19: 1982–1996. 10.1080/15548627.2023.2165323 36622894 PMC10283421

[188] Xie, Yangchun , Rui Kang , Daniel J. Klionsky , and Daolin Tang . 2023. “GPX4 in Cell Death, Autophagy, and Disease.” Autophagy 19: 2621–2638. 10.1080/15548627.2023.2218764 37272058 PMC10472888

[189] Nakamura, Toshitaka , Clara Hipp , André Santos Dias Mourão , Jan Borggräfe , Maceler Aldrovandi , Bernhard Henkelmann , Jonas Wanninger , et al. 2023. “Phase Separation of FSP1 Promotes Ferroptosis.” Nature 619: 371–377. 10.1038/s41586-023-06255-6 37380771 PMC10338336

[190] Lei, Guang , Li Zhuang , and Boyi Gan . 2022. “Targeting Ferroptosis as a Vulnerability in Cancer.” Nature Reviews Cancer 22: 381–396. 10.1038/s41568-022-00459-0 35338310 PMC10243716

[191] Gan, Boyi . 2021. “Mitochondrial Regulation of Ferroptosis.” Journal of Cell Biology 220: e202105043. 10.1083/jcb.202105043 34328510 PMC8329737

[192] Alim, Ishraq , Joseph T. Caulfield , Yingxin Chen , Vivek Swarup , Daniel H. Geschwind , Elena Ivanova , Javier Seravalli , et al. 2019. “Selenium Drives a Transcriptional Adaptive Program to Block Ferroptosis and Treat Stroke.” Cell 177: 1262–1279.e25. 10.1016/j.cell.2019.03.032 31056284

[193] Hansen, Jason M. , Hong Zhang , and Dean P. Jones . 2006. “Differential Oxidation of thioredoxin‐1, thioredoxin‐2, and Glutathione by Metal Ions.” Free Radical Biology and Medicine 40: 138–145. 10.1016/j.freeradbiomed.2005.09.023 16337887

[194] Liu, Zengyi , Ruixin Kang , Ning Yang , Xiuhua Pan , Jie Yang , Hongjie Yu , Wanli Deng , et al. 2024. “Tetrahydrobiopterin Inhibitor‐Based Antioxidant Metabolic Strategy for Enhanced Cancer Ferroptosis‐Immunotherapy.” Journal of Colloid and Interface Science 658: 100–113. 10.1016/j.jcis.2023.12.042 38100967

[195] Huber, Sabrina M. , Ulrike Begley , Anwesha Sarkar , William Gasperi , Evan T. Davis , Vasudha Surampudi , May Lee , et al. 2022. “Arsenite Toxicity Is Regulated by Queuine Availability and Oxidation‐Induced Reprogramming of the Human tRNA Epitranscriptome.” Proceedings of the National Academy of Sciences 119: e2123529119. 10.1073/pnas.2123529119 PMC9499598 36095201

[196] Tian, Ruilin , Anthony Abarientos , Jason Hong , Sayed Hadi Hashemi , Rui Yan , Nina Dräger , Kun Leng , et al. 2021. “Genome‐Wide CRISPRi/a Screens in Human Neurons Link Lysosomal Failure to Ferroptosis.” Nature Neuroscience 24: 1020–1034. 10.1038/s41593-021-00862-0 34031600 PMC8254803

[197] Galluzzi, L. , J. M. Bravo‐San Pedro , and G. Kroemer . 2015. “Ferroptosis in p53‐dependent Oncosuppression and Organismal Homeostasis.” Cell Death & Differentiation 22: 1237–1238. 10.1038/cdd.2015.54 26143748 PMC4495364

[198] Sajid, Sanaullah , Xu Chen , Yanqin Sun , Junjie Luo , Bin Zhang , Linkang Chen , Jieliang Huang , et al. 2025. “A Translational In Vitro to In Vivo Study on Chronic Arsenic Exposure Induced Pulmonary Ferroptosis and Multi‐Omics Analysis of Gut‐Lung Axis Correlation.” Journal of Hazardous Materials 495: 139049. 10.1016/j.jhazmat.2025.139049 40614423

[199] He, Feng , Peng Zhang , Junlai Liu , Ruolei Wang , Randal J. Kaufman , Benjamin C. Yaden , and Michael Karin . 2023. “ATF4 Suppresses Hepatocarcinogenesis by Inducing SLC7A11 (xCT) to Block Stress‐Related Ferroptosis.” Journal of Hepatology 79: 362–377. 10.1016/j.jhep.2023.03.016 36996941 PMC11332364

[200] Wang, Liyuan , Yichen Liu , Tingting Du , Heng Yang , Lei Lei , Mengqi Guo , Han‐Fei Ding , et al. 2020. “ATF3 Promotes Erastin‐Induced Ferroptosis by Suppressing System Xc.” Cell Death & Differentiation 27: 662–675. 10.1038/s41418-019-0380-z 31273299 PMC7206049

[201] Lang, Xueting , Michael D. Green , Weimin Wang , Jiali Yu , Jae Eun Choi , Long Jiang , Peng Liao , et al. 2019. “Radiotherapy and Immunotherapy Promote Tumoral Lipid Oxidation and Ferroptosis via Synergistic Repression of SLC7A11.” Cancer Discovery 9: 1673–1685. 10.1158/2159-8290.CD-19-0338 31554642 PMC6891128

[202] Wang, Yue , Lixin Zheng , Wenjing Shang , Zongcheng Yang , Tongyu Li , Fen Liu , Wei Shao , et al. 2022. “Wnt/Beta‐Catenin Signaling Confers Ferroptosis Resistance by Targeting GPX4 in Gastric Cancer.” Cell Death & Differentiation 29: 2190–2202. 10.1038/s41418-022-01008-w 35534546 PMC9613693

[203] Jiang, Zhou , Seung‐Oe Lim , Meisi Yan , Jennifer L. Hsu , Jun Yao , Yongkun Wei , Shih‐Shin Chang , et al. 2021. “TYRO3 Induces Anti‐PD‐1/PD‐L1 Therapy Resistance by Limiting Innate Immunity and Tumoral Ferroptosis.” The Journal of Clinical Investigation 131(8): e139434. 10.1172/JCI139434 33855973 PMC8262501

[204] Lv, Deguan , Cuiqing Zhong , Deobrat Dixit , Kailin Yang , Qiulian Wu , Bhaskar Godugu , Briana C. Prager , et al. 2023. “EGFR Promotes ALKBH5 Nuclear Retention to Attenuate N6‐methyladenosine and Protect Against Ferroptosis in Glioblastoma.” Molecular Cell 83: 4334–4351.e7. 10.1016/j.molcel.2023.10.025 37979586 PMC10842222

[205] Wang, Huanhuan , Xudan Liu , Yao Chen , Wanying Li , Yanhong Ge , Huning Liang , Bin Xu , and Xin Li . 2024. “The Regulatory Role of miR‐21 in Ferroptosis by Targeting FTH1 and the Contribution of Microglia‐Derived miR‐21 in Exosomes to Arsenic‐Induced Neuronal Ferroptosis.” Journal of Hazardous Materials 478: 135580. 10.1016/j.jhazmat.2024.135580 39186845

[206] Zhang, Haiyang , Ting Deng , Rui Liu , Tao Ning , Haiou Yang , Dongying Liu , Qiumo Zhang , et al. 2020. “CAF Secreted miR‐522 Suppresses Ferroptosis and Promotes Acquired Chemo‐Resistance in Gastric Cancer.” Molecular Cancer 19: 43. 10.1186/s12943-020-01168-8 32106859 PMC7045485

[207] Gao, Ming , Changying Li , Ming Xu , Yun Liu , Min Cong , and Sijin Liu . 2018. “LncRNA MT1DP Aggravates Cadmium‐Induced Oxidative Stress by Repressing the Function of Nrf2 and Is Dependent on Interaction With miR‐365.” Advanced Science 5: 1800087. 10.1002/advs.201800087 30027041 PMC6051394

[208] Liu, Tong , Le Jiang , Omid Tavana , and Wei Gu . 2019. “The Deubiquitylase OTUB1 Mediates Ferroptosis via Stabilization of SLC7A11.” Cancer Research 79: 1913–1924. 10.1158/0008-5472.CAN-18-3037 30709928 PMC6467774

[209] Yang, Jingjing , Yulu Zhou , Shuduo Xie , Ji Wang , Zhaoqing Li , Lini Chen , Misha Mao , et al. 2021. “Metformin Induces Ferroptosis by Inhibiting UFMylation of SLC7A11 in Breast Cancer.” Journal of Experimental & Clinical Cancer Research 40: 206. 10.1186/s13046-021-02012-7 34162423 PMC8223374

[210] Ding, Yahui , Xiaoping Chen , Can Liu , Weizhi Ge , Qin Wang , Xin Hao , Mengmeng Wang , Yue Chen , and Quan Zhang . 2021. “Identification of a Small Molecule as Inducer of Ferroptosis and Apoptosis Through Ubiquitination of GPX4 in Triple Negative Breast Cancer Cells.” Journal of Hematology & Oncology 14: 19. 10.1186/s13045-020-01016-8 33472669 PMC7816340

[211] Yang, Yongfei , Meiying Luo , Kexin Zhang , Jun Zhang , Tongtong Gao , Douglas O' Connell , Fengping Yao , et al. 2020. “Nedd4 Ubiquitylates VDAC2/3 to Suppress Erastin‐Induced Ferroptosis in Melanoma.” Nature Communications 11: 433. 10.1038/s41467-020-14324-x PMC6978386 31974380

[212] Lin, Jinlong , Yixin Yin , Jinghua Cao , Yiyang Zhang , Jiewei Chen , Rixin Chen , Bingxu Zou , et al. 2025. “NUDT21 Lactylation Reprograms Alternative Polyadenylation to Promote Cuproptosis Resistance.” Cell Discovery 11: 52. 10.1038/s41421-025-00804-1 40425546 PMC12116747

[213] Wang, Fudi . 2026. “Targeting Ferroptosis to Halt MASLD and MASH.” Trends in Endocrinology & Metabolism. In press. 10.1016/j.tem.2026.01.007 41916778

[214] Tang, Daolin , Xin Chen , and Guido Kroemer . 2022. “Cuproptosis: a Copper‐Triggered Modality of Mitochondrial Cell Death.” Cell Research 32: 417–418. 10.1038/s41422-022-00653-7 35354936 PMC9061796

[215] Liao, Quan , Jun Deng , Jing Tong , Yu Gan , Weiwei Hong , Hanzhi Dong , Mingming Cao , et al. 2025. “p53 Induces circFRMD4A to Suppress Cancer Development Through Glycolytic Reprogramming and Cuproptosis.” Molecular Cell 85: 132–149.e7. 10.1016/j.molcel.2024.11.013 39637854

[216] Lewis, Alexander C. , Emily Gruber , Rheana Franich , Jessica Armstrong , Madison J. Kelly , Carlos M. Opazo , Yau C. Low , et al. 2026. “Inhibition of Heme Biosynthesis Triggers Cuproptosis in Acute Myeloid Leukemia.” Cell 189: 215–232.e24. 10.1016/j.cell.2025.10.028 41265435

[217] Xue, Qian , Rui Kang , Daniel J. Klionsky , Daolin Tang , Jinbao Liu , and Xin Chen . 2023. “Copper Metabolism in Cell Death and Autophagy.” Autophagy 19: 2175–2195. 10.1080/15548627.2023.2200554 37055935 PMC10351475

[218] Wang, Fudi , WenYe Liu , Cong Tao , Liyun Chen , Ningjun Ni , Liujing Huang , Yueyang Ouyang , et al. 2026. “Copper and Cuproptosis: Mechanisms, Biology, and Roles in Disease.” Science Bulletin. In press. 10.1016/j.scib.2026.05.007 42209382

[219] Li, Su‐Ran , Lin‐Lin Bu , and Lulu Cai . 2022. “Cuproptosis: Lipoylated TCA Cycle Proteins‐Mediated Novel Cell Death Pathway.” Signal Transduction and Targeted Therapy 7: 158. 10.1038/s41392-022-01014-x 35562341 PMC9106713

[220] Yu, Xinying , Bei Li , Jie Yan , Wenxi Li , Hao Tian , Guohao Wang , Songtao Zhou , and Yunlu Dai . 2024. “Cuproptotic Nanoinducer‐Driven Proteotoxic Stress Potentiates Cancer Immunotherapy by Activating the mtDNA‐cGAS‐STING Signaling.” Biomaterials 307: 122512. 10.1016/j.biomaterials.2024.122512 38430646

[221] Lei, Guang , Mingchuang Sun , Jun Cheng , Rui Ye , Zhengze Lu , Amber Horbath , David Huo , et al. 2025. “Radiotherapy Promotes Cuproptosis and Synergizes with Cuproptosis Inducers to Overcome Tumor Radioresistance.” Cancer Cell 43: 1076–1092.e5. 10.1016/j.ccell.2025.03.031 40215978 PMC12151758

[222] Lu, Sheng , Yifan Li , and Yingjie Yu . 2024. “Glutathione‐Scavenging Celastrol‐Cu Nanoparticles Induce Self‐Amplified Cuproptosis for Augmented Cancer Immunotherapy.” Advanced Materials 36: e2404971. 10.1002/adma.202404971 38935977

[223] Schulz, Vinzent , Somsuvro Basu , Sven‐ A. Freibert , Holger Webert , Linda Boss , Ulrich Mühlenhoff , Fabien Pierrel , et al. 2023. “Functional Spectrum and Specificity of Mitochondrial Ferredoxins FDX1 and FDX2.” Nature Chemical Biology 19: 206–217. 10.1038/s41589-022-01159-4 36280795 PMC10873809

[224] Lu, Xufeng , Xiaodong Chen , Chengyin Lin , Yongdong Yi , Shengsheng Zhao , Bingzi Zhu , Wenhai Deng , et al. 2024. “Elesclomol Loaded Copper Oxide Nanoplatform Triggers Cuproptosis to Enhance Antitumor Immunotherapy.” Advanced Science 11: e2309984. 10.1002/advs.202309984 38430531 PMC11095170

[225] Zhang, Meiru , Hui Xu , Xiaozan Wu , Botao Chen , Xiyu Gong , and Yongju He . 2025. “Engineering Dual‐Responsive Nanoplatform Achieves Copper Metabolism Disruption and Glutathione Consumption to Provoke Cuproptosis/Ferroptosis/Apoptosis for Cancer Therapy.” ACS Applied Materials & Interfaces 17: 20726–20740. 10.1021/acsami.4c22546 40134095

[226] Wang, Yang , Tingting Yan , Jinming Cai , Hongjing Dou , Yu Zhu , Bijiang Geng , Dengyu Pan , and Longxiang Shen . 2025. “A Heterojunction‐Engineering Nanodrug With Tumor Microenvironment Responsiveness for Tumor‐Specific Cuproptosis and Chemotherapy Amplified Sono‐Immunotherapy.” Biomaterials 321: 123319. 10.1016/j.biomaterials.2025.123319 40187098

[227] Xie, Dingqi , Chuan Hu , Yutao Zhu , Jia Yao , Jianyi Li , Jiechao Xia , Lin Ye , et al. 2025. “Sequential Therapy for Osteosarcoma and Bone Regeneration via Chemodynamic Effect and Cuproptosis Using a 3D‐Printed Scaffold with TME‐Responsive Hydrogel.” Small 21: e2406639. 10.1002/smll.202406639 39908123

[228] Zhao, Chen , Xiaoying Tang , Xiaoyuan Chen , Chen , and Zhenqi Jiang . 2024. “Multifaceted Carbonized Metal‐Organic Frameworks Synergize With Immune Checkpoint Inhibitors for Precision and Augmented Cuproptosis Cancer Therapy.” ACS Nano 18: 17852–17868. 10.1021/acsnano.4c04022 38939981

[229] Li, Jiehan , Ge Zhang , Zhao Sun , Meimei Jiang , Guiyun Jia , Hao Liu , Nannan Liu , et al. 2025. “Immunogenic Cuproptosis in Cancer Immunotherapy Via an in Situ Cuproptosis‐Inducing System.” Biomaterials 319: 123201. 10.1016/j.biomaterials.2025.123201 40020502

[230] Liao, You , Dongmei Wang , Chenglu Gu , Xue Wang , Shuang Zhu , Ziye Zheng , Fuquan Zhang , Junfang Yan , and Zhanjun Gu . 2024. “A Cuproptosis Nanocapsule for Cancer Radiotherapy.” Nature Nanotechnology 19: 1892–1902. 10.1038/s41565-024-01784-1 39300223

[231] Jiang, Tiaoyan , Tianying Jia , Yipengchen Yin , Tianyu Li , Xinran Song , Wei Feng , Sheng Wang , et al. 2025. “Cuproptosis‐Inducing Functional Nanocomposites for Enhanced and Synergistic Cancer Radiotherapy.” ACS Nano 19: 5429–5446. 10.1021/acsnano.4c13753 39895200

[232] Yan, Lang , Liang Chang , Yijun Tian , Jinyan Hu , Zhi Cao , Xiang Guo , and Bijiang Geng . 2025. “Graphene Quantum Dot Sensitized Heterojunctions Induce Tumor‐Specific Cuproptosis to Boost Sonodynamic and Chemodynamic Enhanced Cancer Immunotherapy.” Advanced Science 12: e2410606. 10.1002/advs.202410606 39716968 PMC11831527

[233] Ni, Cheng , Zhijun Ouyang , Gaoming Li , Junjie Liu , Xueyan Cao , Linfeng Zheng , Xiangyang Shi , and Rui Guo . 2023. “A Tumor Microenvironment‐Responsive Core‐Shell Tecto Dendrimer Nanoplatform for Magnetic Resonance Imaging‐Guided and Cuproptosis‐Promoted Chemo‐Chemodynamic Therapy.” Acta Biomaterialia 164: 474–486. 10.1016/j.actbio.2023.04.003 37040813

[234] Liu, Shiwei , Wennan Yan , Wenyue Zhang , Ji Zhang , Ziyi Li , Yingshu Guo , Hong‐Yuan Chen , and Jing‐Juan Xu . 2025. “Nanoenhanced‐Cuproptosis Results from the Synergy of Calcium Overload and GSH Depletion with the Increasing of Intracellular Ca/Mn/Cu Ions.” Advanced Science 12: e2412067. 10.1002/advs.202412067 39928524 PMC11967785

[235] Huang, Jia , Fuzhen Hu , Hanchen Zhang , Zheng Cao , Haihua Xiao , Zhiying Yang , Qionghua Jin , and Kun Shang . 2025. “Ultrasound‐Triggered Nanoparticles Induce Cuproptosis for Enhancing Immunogenic Sonodynamic Therapy.” Advanced Materials 37: e2504228. 10.1002/adma.202504228 40357877

[236] Cun, Ju‐E , Ziyun He , Xi Fan , Qingqing Pan , Kui Luo , Bin He , and Yuji Pu . 2025. “Copper‐Based Bio‐Coordination Nanoparticle for Enhanced Pyroptosis‐Cuproptosis Cancer Immunotherapy Through Redox Modulation and Glycolysis Inhibition.” Small 21: e2409875. 10.1002/smll.202409875 39757406

[237] Wang, Zhenxin , Yuting Li , Congcong Wang , Jun Lan , Jiale Li , Guanhong Liu , Yuxing Chen , et al. 2026. “Disrupting Intracellular Redox Homeostasis Through Copper‐Driven Dual Cell Death to Induce Anti‐Tumor Immunotherapy.” Biomaterials 324: 123523. 10.1016/j.biomaterials.2025.123523 40592037

[238] Jiang, Minhao , Penghui Li , Yinuo Shu , Guoshi Xu , Yinghua Peng , Fang Pu , Anjun Song , Jinsong Ren , and Xiaogang Qu . 2026. “Self‐Carrier Nanoagonist Enabling Positive Feedback Regulation of Cuproptosis‐Immunity for Potent Antitumor Therapy.” ACS Nano 20: 2399–2412. 10.1021/acsnano.5c19675 41490802

[239] Cobine, Paul A. , and Donita C. Brady . 2022. “Cuproptosis: Cellular and Molecular Mechanisms Underlying Copper‐Induced Cell Death.” Molecular Cell 82: 1786–1787. 10.1016/j.molcel.2022.05.001 35594843

[240] Tang, Daolin , Guido Kroemer , and Rui Kang . 2024. “Targeting Cuproplasia and Cuproptosis in Cancer.” Nature Reviews Clinical Oncology 21: 370–388. 10.1038/s41571-024-00876-0 38486054

[241] Dreishpoon, Margaret B. , Nolan R. Bick , Boryana Petrova , Douglas M. Warui , Alison Cameron , Squire J. Booker , Naama Kanarek , Todd R. Golub , and Peter Tsvetkov . 2023. “FDX1 Regulates CellulCuproptosis‐Based Nanomedicinear Protein Lipoylation Through Direct Binding to LIAS.” Journal of Biological Chemistry 299(9): 105046. 10.1016/j.jbc.2023.105046 37453661 PMC10462841

[242] Gu, Yan , Hongchang Wang , Wentao Xue , Linjia Zhu , Chenghao Fu , Wenhao Zhang , Guang Mu , et al. 2025. “Endoplasmic Reticulum Stress Related Super‐Enhancers Suppress Cuproptosis via Glycolysis Reprogramming in Lung Adenocarcinoma.” Cell Death & Disease 16: 316. 10.1038/s41419-025-07613-0 40253387 PMC12009302

[243] Zhang, Zhen , Xiangyang Zeng , Yinghua Wu , Yang Liu , Xi Zhang , and Zewen Song . 2022. “Cuproptosis‐Related Risk Score Predicts Prognosis and Characterizes the Tumor Microenvironment in Hepatocellular Carcinoma.” Frontiers in Immunology 13: 925618. 10.3389/fimmu.2022.925618 35898502 PMC9311491

[244] Sun, Bo , Peng Ding , Yinghui Song , Jia Zhou , Xu Chen , Chuang Peng , and Sulai Liu . 2024. “FDX1 Downregulation Activates Mitophagy and the PI3K/AKT Signaling Pathway to Promote Hepatocellular Carcinoma Progression by Inducing ROS Production.” Redox Biology 75: 103302. 10.1016/j.redox.2024.103302 39128228 PMC11366913

[245] Li, Xinqiang , Peng Jiang , Ruixia Li , Bin Wu , Kai Zhao , Shipeng Li , and Jinzhen Cai . 2022. “Analysis of Cuproptosis in Hepatocellular Carcinoma Using Multi‐Omics Reveals a Comprehensive HCC Landscape and the Immune Patterns of Cuproptosis.” Frontiers in Oncology 12: 1009036. 10.3389/fonc.2022.1009036 36408192 PMC9666696

[246] Xing, Tao , Li Li , Yiran Chen , Gaoda Ju , Guilan Li , Xiaoyun Zhu , Yubo Ren , et al. 2023. “Targeting the TCA Cycle Through Cuproptosis Confers Synthetic Lethality on ARID1A‐Deficient Hepatocellular Carcinoma.” Cell Reports Medicine 4: 101264. 10.1016/j.xcrm.2023.101264 37939712 PMC10694624

[247] Xiong, Chen , Hong Ling , Qian Hao , and Xiang Zhou . 2023. “Cuproptosis: p53‐regulated Metabolic Cell Death?” Cell Death & Differentiation 30: 876–884. 10.1038/s41418-023-01125-0 36755067 PMC10070433

[248] Sheng, Xiyang , Chen Mi , Gengyuan Shi , Longbo Wang , Yongzhao Li , Dongdong Wang , Wei Wang , et al. 2026. “Targeting Copper Death‐Related Long Non‐Coding RNAs: A Novel Strategy to Overcome Immunotherapy Resistance in Liver Cancer.” Frontiers in Immunology 17: 1743964. 10.3389/fimmu.2026.1743964 41846915 PMC12989404

[249] Shen, Wenhao , Pei Pei , Chonghai Zhang , Junmei Li , Xiangming Han , Teng Liu , Xiumin Shi , et al. 2023. “A Polymeric Hydrogel to Eliminate Programmed Death‐Ligand 1 for Enhanced Tumor Radio‐Immunotherapy.” ACS Nano 17: 23998–24011. 10.1021/acsnano.3c08875 37988029

[250] Ning, Shipeng , Meng Lyu , Daoming Zhu , Jacky W. Y. Lam , Qinqin Huang , Tianfu Zhang , and Ben Zhong Tang . 2023. “Type‐I AIE Photosensitizer Loaded Biomimetic System Boosting Cuproptosis to Inhibit Breast Cancer Metastasis and Rechallenge.” ACS Nano 17: 10206–10217. 10.1021/acsnano.3c00326 37183977

[251] Xu, Xinzhi , Hang Zhou , Ruixia Hong , Jiaqi Gong , Yujie Wan , Qihuan Fu , Kaifeng Huang , et al. 2025. “A Self‐Accelerating ‘Copper Bomb’ Strategy Activated Innate and Adaptive Immune Response against Triple‐Negative Breast Cancer.” Bioactive Materials 49: 193–206. 10.1016/j.bioactmat.2025.02.019 40130080 PMC11931225

[252] Xu, Yiming , Yuan Wu , Xinjie Zheng , Dongxue Wang , Hangqi Ni , Weiyu Chen , and Kai Wang . 2025. “A Smart Nanomedicine Unleashes a Dual Assault of Glucose Starvation and Cuproptosis to Supercharge alphaPD‐L1 Therapy.” Advanced Science 12: e2411378. 10.1002/advs.202411378 39632613 PMC11775525

[253] Xu, Yuzhi , Si‐Yang Liu , Leli Zeng , Hansu Ma , Yanfei Zhang , Huihui Yang , Yuchen Liu , et al. 2022. “An Enzyme‐Engineered Nonporous Copper(I) Coordination Polymer Nanoplatform for Cuproptosis‐Based Synergistic Cancer Therapy.” Advanced Materials 34: e2204733. 10.1002/adma.202204733 36054475

[254] Bonnet, Sébastien , Stephen L. Archer , Joan Allalunis‐Turner , Alois Haromy , Christian Beaulieu , Richard Thompson , Christopher T. Lee , et al. 2007. “A Mitochondria‐K+ Channel Axis Is Suppressed in Cancer and Its Normalization Promotes Apoptosis and Inhibits Cancer Growth.” Cancer Cell 11: 37–51. 10.1016/j.ccr.2006.10.020 17222789

[255] Xiao, Chen , Xing Wang , Shiyou Li , Zhijie Zhang , Jiayuan Li , Qingyuan Deng , Xiang Chen , Chen , Xiangliang Yang , and Zifu Li . 2025. “A Cuproptosis‐Based Nanomedicine Suppresses Triple Negative Breast Cancers by Regulating Tumor Microenvironment and Eliminating Cancer Stem Cells.” Biomaterials 313: 122763. 10.1016/j.biomaterials.2024.122763 39180917

[256] Tao, Jiaojiao , Yu Dong , Bingjie Wang , Teng Wang , Aijia Zhang , Shuang Li , Rui Chen , et al. 2025. “Dual Metal Nanoflower Oxygen Pump Microneedles Based on Cuproptosis and STING Pathway Activation for Cancer Immunotherapy.” Small 21: e2409187. 10.1002/smll.202409187 39950396

[257] Wu, Guoquan , Tianyu Su , Peng Zhou , Rongze Tang , Xu Zhu , Jin Wang , Minghao Chao , et al. 2025. “Engineering M2 Macrophage‐Derived Exosomes Modulate Activated T Cell Cuproptosis to Promote Immune Tolerance in Rheumatoid Arthritis.” Biomaterials 315: 122943. 10.1016/j.biomaterials.2024.122943 39509857

[258] Sun, Zicheng , Huazhen Xu , Guanming Lu , Ciqiu Yang , Xinya Gao , Jing Zhang , Xin Liu , et al. 2025. “AKT1 Phosphorylates FDX1 to Promote Cuproptosis Resistance in Triple‐Negative Breast Cancer.” Advanced Science 12: e2408106. 10.1002/advs.202408106 39976173 PMC12061301

[259] Yuan, Fanen , Xujia Wu , Huairui Yuan , Donghai Wang , Tengfei Huang , Po Zhang , Hailong Mi , et al. 2026. “Glioblastoma Stem Cells Resist Cuproptosis With Circadian Variation of Copper Levels.” The Journal of Clinical Investigation 136(1): e192599. 10.1172/JCI192599 41480765 PMC12721906

[260] Hu, Huiqun , Shiyuan Hua , Feng Lu , Wenting Zhang , Zengwen Zhang , Jiarong Cui , Xiaoyue Lei , et al. 2025. “Mucous Permeable Nanoparticle for Inducing Cuproptosis‐Like Death in Broad‐Spectrum Bacteria for Nebulized Treatment of Acute Pneumonia.” Advanced Science 12: e2408580. 10.1002/advs.202408580 39985298 PMC12005761

[261] Wang, Yu , Xiaoyang Yao , Yingying Lu , Juan Ruan , Zhao Yang , Chunhui Wang , Niantong Yang , Yan Gao , and Shuo Shi . 2025. “A PROTAC‐Based Cuproptosis Sensitizer in Lung Cancer Therapy.” Advanced Materials 37: e2501435. 10.1002/adma.202501435 40495637

[262] Feng, Li , Ti‐Zhi Wu , Xin‐Rui Guo , Yun‐Jie Wang , Xin‐Jia Wang , Shao‐Xuan Liu , Rui Zhang , et al. 2025. “Discovery of Natural Resorcylic Acid Lactones as Novel Potent Copper Ionophores Covalently Targeting PRDX1 to Induce Cuproptosis for Triple‐Negative Breast Cancer Therapy.” ACS Central Science 11: 357–370. 10.1021/acscentsci.4c02188 40028362 PMC11869127

[263] Li, Zi‐Zhan , Yi Liu , Kan Zhou , Lei‐Ming Cao , Guang‐Rui Wang , Jinmei Wu , Yi‐Fu Yu , et al. 2025. “ORL@Cu‐MOF Boost Cuproptosis and Suppress Fatty Acid Metabolism for Cancer Lymph Node Metastasis Synergistic Therapy.” Advanced Science 12: e02154. 10.1002/advs.202502154 40548889 PMC12463119

[264] Shen, Yefeng , Deyu Li , Qiong Liang , Mengsi Yang , Youguang Pan , and Hui Li . 2022. “Cross‐Talk between Cuproptosis and Ferroptosis Regulators Defines the Tumor Microenvironment for the Prediction of PrognMitochondrial Calcium Uniporter Regulationosis and Therapies in Lung Adenocarcinoma.” Frontiers in Immunology 13: 1029092. 10.3389/fimmu.2022.1029092 36733399 PMC9887127

[265] Gou, Yi , MeiRong Chen , Shanhe Li , JunGang Deng , Jinlong Li , GuiHua Fang , Feng Yang , and GuoJin Huang . 2021. “Dithiocarbazate‐Copper Complexes for Bioimaging and Treatment of Pancreatic Cancer.” Journal of Medicinal Chemistry 64: 5485–5499. 10.1021/acs.jmedchem.0c01936 33861929

[266] Ma, Baojin , Shu Wang , Feng Liu , Shan Zhang , Jiazhi Duan , Zhao Li , Ying Kong , et al. 2019. “Self‐Assembled Copper–Amino Acid Nanoparticles for in Situ Glutathione ‘AND’ H_2_O_2_ Sequentially Triggered Chemodynamic Therapy.” Journal of the American Chemical Society 141: 849–857. 10.1021/jacs.8b08714 30541274

[267] Jiang, Cong , Xianglong Li , Shiyue Wan , Shuyu Ji , Qinghua Wang , Shiqi Hu , Pengcheng Chen , et al. 2025. “Copper‐Doped Polydopamine Nanoparticles‐Mediated GSH/GPX4‐Depleted Ferroptosis and Cuproptosis Sensitizes Lung Tumor to Checkpoint Blockade Immunotherapy.” Small 21: e2503208. 10.1002/smll.202503208 40231637

[268] Xu, Tangbing , Qiming Ma , Chi Zhang , Xiaoyan He , Qian Wang , Yunfeng Wu , Kunpeng Qin , et al. 2025. “A Novel Nanomedicine for Osteosarcoma Treatment: Triggering Ferroptosis Through GSH Depletion and Inhibition for Enhanced Synergistic PDT/PTT Therapy.” Journal of Nanobiotechnology 23: 323. 10.1186/s12951-025-03380-4 40301915 PMC12039277

[269] Liu, Bin , Xiaorui Chen , Yanlin Zhu , Hao Chen , Jia Tan , Zhuang Yang , Jing Li , et al. 2025. “One‐Step Symbiosis of Bimetallic Peroxides Nanoparticles to Induce Ferroptosis/Cuproptosis and Activate cGAS‐STING Pathway for Enhanced Tumor Immunotherapy.” Advanced Materials 37: e2500337. 10.1002/adma.202500337 40181655

[270] Huang, Lin , Jiaoyang Zhu , Wei Xiong , Jie Feng , Jing Yang , Xuanyi Lu , Yudie Lu , et al. 2023. “Tumor‐Generated Reactive Oxygen Species Storm for High‐Performance Ferroptosis Therapy.” ACS Nano 17: 11492–11506. 10.1021/acsnano.3c01369 37283506

[271] Li, Yuanhui , Jian Li , Yuxuan Zhong , Qingshun Zhang , Yuchun Wu , Jinpeng Huang , Kaicheng Pang , et al. 2025. “pH‐Responsive and Nanoenzyme‐Loaded Artificial Nanocells Relieved Osteomyelitis Efficiently by Synergistic Chemodynamic and Cuproptosis Therapy.” Biomaterials 313: 122762. 10.1016/j.biomaterials.2024.122762 39178559

[272] Li, Youyou , Jing Liu , Yimei Chen , Ralph R. Weichselbaum , and Wenbin Lin . 2024. “Nanoparticles Synergize Ferroptosis and Cuproptosis to Potentiate Cancer Immunotherapy.” Advanced Science 11: e2310309. 10.1002/advs.202310309 38477411 PMC11187894

[273] Yu, Wenxin , Duo Jin , Yajie Zhang , Shenghu Wang , Jiaji Yu , Manman Liu , Yi Dai , et al. 2025. “Provoking Tumor Disulfidptosis by Single‐Atom Nanozyme Via Regulating Cellular Energy Supply and Reducing Power.” Nature Communications 16: 4877. 10.1038/s41467-025-60015-w PMC12106855 40419525

[274] Watson, Shane A. , and Gavin P. McStay . 2020. “Functions of Cytochrome C Oxidase Assembly Factors.” International Journal of Molecular Sciences 21: 7254. 10.3390/ijms21197254 33008142 PMC7582755

[275] Xia, Jiechao , Chuan Hu , Yinwen Ji , Min Wang , Yang Jin , Lin Ye , Dingqi Xie , et al. 2023. “Copper‐Loaded Nanoheterojunction Enables Superb Orthotopic Osteosarcoma Therapy via Oxidative Stress and Cell Cuproptosis.” ACS Nano 17: 21134–21152. 10.1021/acsnano.3c04903 37902237

[276] Zhang, Meixiang , Xinran Song , Yu Qin , Yuanyuan Peng , Shanshan Zhang , Wei Feng , Hui Huang , Yu Chen , and Jianqiao Zhou . 2025. “Single‐Atom‐Doped Piezocatalyst Induces Copper‐Free Cuproptosis in Tumor Therapy.” Science Advances 11: eadt8451. 10.1126/sciadv.adt8451 39951535 PMC11827870

[277] Guo, Hongrui , Yujuan Ouyang , Heng Yin , Hengmin Cui , Huidan Deng , Huan Liu , Zhijie Jian , et al. 2022. “Induction of Autophagy via the ROS‐Dependent AMPK‐mTOR Pathway Protects Copper‐Induced Spermatogenesis Disorder.” Redox Biology 49: 102227. 10.1016/j.redox.2021.102227 34979450 PMC8728583

[278] Jin, Xiao‐Kang , Shun‐Kang Zhang , Shi‐Man Zhang , Jun‐Long Liang , Xiao Yan , Yan‐Tong Lin , Ran Meng , et al. 2025. “Disrupting Intracellular Homeostasis by Copper‐Based Nanoinducer with Multiple Enzyme‐Mimicking Activities to Induce Disulfidptosis‐Enhanced Pyroptosis for Tumor Immunotherapy.” Advanced Materials 37: e2410957. 10.1002/adma.202410957 39468892

[279] Gu, Lina , Ying Sun , Tingjie Bai , Sijie Shao , Shumin Tang , Panpan Xue , Wanlin Cai , et al. 2025. “Functional Nanozyme System for Synergistic Tumor Immunotherapy via Cuproptosis and Ferroptosis Activation.” Journal of Nanobiotechnology 23: 212. 10.1186/s12951-025-03284-3 40089774 PMC11909888

[280] Chen, Kerong , Anwei Zhou , Xinyuan Zhou , Jielei He , Yurui Xu , and Xinghai Ning . 2024. “Cellular Trojan Horse Initiates Bimetallic Fe‐Cu MOF‐mediated Synergistic Cuproptosis and Ferroptosis against Malignancies.” Science Advances 10: eadk3201. 10.1126/sciadv.adk3201 38598629 PMC11006215

[281] Chen, Wei , Wenyu Xie , Zhimin Gao , Chen Lin , Meiling Tan , Yaru Zhang , and Zhiyao Hou . 2023. “Mild‐Photothermal Effect Induced High Efficiency Ferroptosis‐Boosted‐Cuproptosis Based on Cu(2) O@Mn(3) Cu(3) O(8) Nanozyme.” Advanced Science 10: e2303694. 10.1002/advs.202303694 37822154 PMC10667815

[282] Wang, Weikai , Kaizhong Lu , Xin Jiang , Qi Wei , Liyuan Zhu , Xian Wang , Hongchuan Jin , and Lifeng Feng . 2023. “Ferroptosis Inducers Enhanced Cuproptosis Induced by Copper Ionophores in Primary Liver Cancer.” Journal of Experimental & Clinical Cancer Research 42: 142. 10.1186/s13046-023-02720-2 37277863 PMC10242978

[283] Zhu, Yang , Xuegang Niu , Chengyu Ding , Yuanxiang Lin , Wenhua Fang , Lingjun Yan , Junjie Cheng , et al. 2024. “Carrier‐Free Self‐Assembly Nano‐Sonosensitizers for Sonodynamic‐Amplified Cuproptosis‐Ferroptosis in Glioblastoma Therapy.” Advanced Science 11: e2402516. 10.1002/advs.202402516 38582500 PMC11187904

[284] Huang, Yuting , Xueliang Liu , Jiawei Zhu , Zhejie Chen , Lu Yu , Xin Huang , Chuhuang Dong , et al. 2024. “Enzyme Core Spherical Nucleic Acid That Enables Enhanced Cuproptosis and Antitumor Immune Response Through Alleviating Tumor Hypoxia.” Journal of the American Chemical Society 146: 13805–13816. 10.1021/jacs.3c14247 38552185

[285] Bock, Florian J. , and Stephen W. G. Tait . 2020. “Mitochondria As Multifaceted Regulators of Cell Death.” Nature Reviews Molecular Cell Biology 21: 85–100. 10.1038/s41580-019-0173-8 31636403

[286] De Stefani, Diego , Anna Raffaello , Enrico Teardo , Ildikò Szabò , and Rosario Rizzuto . 2011. “A Forty‐Kilodalton Protein of the Inner Membrane Is the Mitochondrial Calcium Uniporter.” Nature 476: 336–340. 10.1038/nature10230 21685888 PMC4141877

[287] Giorgio, Valentina , Victoria Burchell , Marco Schiavone , Claudio Bassot , Giovanni Minervini , Valeria Petronilli , Francesco Argenton , et al. 2017. “Ca^2+^ Binding to F‐ATP Synthase β Subunit Triggers the Mitochondrial Permeability Transition.” The EMBO Reports 18: 1065–1076. 10.15252/embr.201643354 28507163 PMC5494526

[288] Haworth, Robert A. , and Douglas R. Hunter . 1979. “The Ca2+‐induced Membrane Transition in Mitochondria.” Archives of Biochemistry and Biophysics 195: 460–467. 10.1016/0003-9861(79)90372-2 38751

[289] Roca, Francisco J. , Laura J. Whitworth , Sarah Redmond , Ana A. Jones , and Lalita Ramakrishnan . 2019. “TNF Induces Pathogenic Programmed Macrophage Necrosis in Tuberculosis Through a Mitochondrial‐Lysosomal‐Endoplasmic Reticulum Circuit.” Cell 178: 1344–1361.e11<e1311. 10.1016/j.cell.2019.08.004 31474371 PMC6736209

[290] König, Tim , Simon E. Tröder , Kavya Bakka , Anne Korwitz , Ricarda Richter‐Dennerlein , Philipp A. Lampe , Maria Patron , et al. 2016. “The m‐AAA Protease Associated With Neurodegeneration Limits MCU Activity in Mitochondria.” Molecular Cell 64: 148–162. 10.1016/j.molcel.2016.08.020 27642048

[291] Dong, Zhiwei , Santhanam Shanmughapriya , Dhanendra Tomar , Naveed Siddiqui , Solomon Lynch , Neeharika Nemani , Sarah L. Breves , et al. 2017. “Mitochondrial Ca(2+) Uniporter Is a Mitochondrial Luminal Redox Sensor That Augments MCU Channel Activity.” Molecular Cell 65: 1014–1028.e7. 10.1016/j.molcel.2017.01.032 28262504 PMC5357178

[292] Hwang, M. S. , C. T. Schwall , E. Pazarentzos , C. Datler , N. N. Alder , and S. Grimm . 2014. “Mitochondrial Ca(2+) Influx Targets Cardiolipin to Disintegrate Respiratory Chain Complex II for Cell Death Induction.” Cell Death & Differentiation 21: 1733–1745. 10.1038/cdd.2014.84 24948011 PMC4211371

[293] Urbani, Andrea , Valentina Giorgio , Andrea Carrer , Cinzia Franchin , Giorgio Arrigoni , Chimari Jiko , Kazuhiro Abe , et al. 2019. “Purified F‐ATP Synthase Forms a Ca^2+^‐Dependent High‐Conductance Channel Matching the Mitochondrial Permeability Transition Pore.” Nature Communications 10: 4341. 10.1038/s41467-019-12331-1 PMC6761146 31554800

[294] Kumar, Amrendra , Juliana da Fonseca Rezende e Mello , Yangyu Wu , Daniel Morris , Ikram Mezghani , Erin Smith , Stephane Rombauts , et al. 2025. “Cryo‐EM Structure of the Brine Shrimp Mitochondrial ATP Synthase Suggests an Inactivation Mechanism for the ATP Synthase Leak Channel.” Cell Death & Differentiation 32: 1518–1535. 10.1038/s41418-025-01476-w 40108410 PMC12325954

[295] Baines, Christopher P. , Robert A. Kaiser , Nicole H. Purcell , N. Scott Blair , Hanna Osinska , Michael A. Hambleton , Eric W. Brunskill , et al. 2005. “Loss of Cyclophilin D Reveals a Critical Role for Mitochondrial Permeability Transition in Cell Death.” Nature 434: 658–662. 10.1038/nature03434 15800627

[296] Cao, Chan , Shuqing Wang , Tanxing Cui , Xun‐Cheng Su , and James J. Chou . 2017. “Ion and Inhibitor Binding of the Double‐Ring Ion Selectivity Filter of the Mitochondrial Calcium Uniporter.” Proceedings of the National Academy of Sciences 114: E2846–E2851. 10.1073/pnas.1620316114 PMC5389301 28325874

[297] Paillard, Melanie , György Csordás , Kai‐Ting Huang , Peter Várnai , Suresh K. Joseph , and György Hajnóczky . 2018. “MICU1 Interacts With the D‐Ring of the MCU Pore to Control Its Ca^2+^ Flux and Sensitivity to Ru360.” Molecular Cell 72: 778–785.e3. 10.1016/j.molcel.2018.09.008 30454562 PMC6251499

[298] Patron, Maria , Vanessa Checchetto , Anna Raffaello , Enrico Teardo , Denis Vecellio Reane , Maura Mantoan , Veronica Granatiero , et al. 2014. “MICU1 and MICU2 Finely Tune the Mitochondrial Ca^2+^ Uniporter by Exerting Opposite Effects on MCU Activity.” Molecular Cell 53: 726–737. 10.1016/j.molcel.2014.01.013 24560927 PMC3988891

[299] Lee, Samuel K. , Santhanam Shanmughapriya , Mac C. Y. Mok , Zhiwei Dong , Dhanendra Tomar , Edmund Carvalho , Sudarsan Rajan , et al. 2016. “Structural Insights Into Mitochondrial Calcium Uniporter Regulation by Divalent Cations.” Cell Chemical Biology 23: 1157–1169. 10.1016/j.chembiol.2016.07.012 27569754 PMC5035232

[300] Delgado de la Herran, Hilda , Denis Vecellio Reane , Yiming Cheng , Máté Katona , Fabian Hosp , Elisa Greotti , Jennifer Wettmarshausen , et al. 2024. “Systematic Mapping of Mitochondrial Calcium Uniporter Channel (MCUC)‐mediated Calcium Signaling Networks.” The EMBO Journal 43: 5288–5326. 10.1038/s44318-024-00219-w 39261663 PMC11535509

[301] Wang, Jin , Jinyong Jiang , Haoliang Hu , and Linxi Chen . 2025. “MCU Complex: Exploring Emerging Targets and Mechanisms of Mitochondrial Physiology and Pathology.” Journal of Advanced Research 68: 271–298. 10.1016/j.jare.2024.02.013 38417574 PMC11785567

[302] Palty, Raz , William F. Silverman , Michal Hershfinkel , Teresa Caporale , Stefano L. Sensi , Julia Parnis , Christiane Nolte , et al. 2010. “NCLX Is an Essential Component of Mitochondrial Na^+^/Ca^2+^ Exchange.” Proceedings of the National Academy of Sciences 107: 436–441. 10.1073/pnas.0908099107 PMC2806722 20018762

[303] Kerkhofs, Martijn , Mart Bittremieux , Giampaolo Morciano , Carlotta Giorgi , Paolo Pinton , Jan B. Parys , and Geert Bultynck . 2018. “Emerging Molecular Mechanisms in Chemotherapy: Ca2+ Signaling at the Mitochondria‐Associated Endoplasmic Reticulum Membranes.” Cell Death & Disease 9: 334. 10.1038/s41419-017-0179-0 29491433 PMC5832420

[304] Szabadkai, György , Katiuscia Bianchi , Péter Várnai , Diego De Stefani , Mariusz R. Wieckowski , Dario Cavagna , Anikó I. Nagy , Tamás Balla , and Rosario Rizzuto . 2006. “Chaperone‐Mediated Coupling of Endoplasmic Reticulum and Mitochondrial Ca2+ Channels.” Journal of Cell Biology 175: 901–911. 10.1083/jcb.200608073 17178908 PMC2064700

[305] Ricci, Jean‐Ehrland , Cristina Muñoz‐Pinedo , Patrick Fitzgerald , Béatrice Bailly‐Maitre , Guy A. Perkins , Nagendra Yadava , Immo E. Scheffler , Mark H. Ellisman , and Douglas R. Green . 2004. “Disruption of Mitochondrial Function during Apoptosis Is Mediated by Caspase Cleavage of the p75 Subunit of Complex I of the Electron Transport Chain.” Cell 117: 773–786. 10.1016/j.cell.2004.05.008 15186778

[306] Head, Brian , Lorena Griparic , Mandana Amiri , Shilpa Gandre‐Babbe , and Alexander M. van der Bliek . 2009. “Inducible Proteolytic Inactivation of OPA1 Mediated by the OMA1 Protease in Mammalian Cells.” Journal of Cell Biology 187: 959–966. 10.1083/jcb.200906083 20038677 PMC2806274

[307] Luongo, Timothy S. , Jonathan P. Lambert , Ancai Yuan , Xueqian Zhang , Polina Gross , Jianliang Song , Santhanam Shanmughapriya , et al. 2015. “The Mitochondrial Calcium Uniporter Matches Energetic Supply with Cardiac Workload during Stress and Modulates Permeability Transition.” Cell Reports 12: 23–34. 10.1016/j.celrep.2015.06.017 26119731 PMC4517182

[308] Guidarelli, Andrea , Mara Fiorani , Liana Cerioni , and Orazio Cantoni . 2019. “Calcium Signals between the Ryanodine Receptor‐ and Mitochondria Critically Regulate the Effects of Arsenite on Mitochondrial Superoxide Formation and on the Ensuing Survival Vs Apoptotic Signaling.” Redox Biology 20: 285–295. 10.1016/j.redox.2018.10.015 30388683 PMC6216081

[309] Frandsen, Stine K. , Mie B. Krüger , Uma M. Mangalanathan , Trine Tramm , Faisal Mahmood , Ivana Novak , and Julie Gehl . 2017. “Normal and Malignant Cells Exhibit Differential Responses to Calcium Electroporation.” Cancer Research 77: 4389–4401. 10.1158/0008-5472.CAN-16-1611 28760856

[310] Eskiocak, Ugur , Vijayashree Ramesh , Jennifer G. Gill , Zhiyu Zhao , Stacy W. Yuan , Meng Wang , Travis Vandergriff , et al. 2016. “Synergistic Effects of Ion Transporter and MAP Kinase Pathway Inhibitors in Melanoma.” Nature Communications 7: 12336. 10.1038/ncomms12336 PMC4996948 27545456

[311] Qiao, Yuhui , Jianghuang Wang , Bohong Wang , Hong Zhou , Qianlin Ni , Wan Fu , Zeping Hu , and Qing Zhong . 2025. “Sodium Disrupts Mitochondrial Energy Metabolism to Execute NECSO.” Nature Communications 17: 493. 10.1038/s41467-025-67181-x PMC12804697 41390760

[312] Qu, Xiangyu , Yigang Zhang , Yilun Shi , Suchen Wang , Yi Tan , Lianbao Kong , and Deming Zhu . 2025. “Construction of a Prognostic Model for Hepatocellular Carcinoma Based on Necrosis by Sodium Overload‐Related Genes and Identification of ANKRD13B As a New Prognostic Marker.” Functional & Integrative Genomics 25: 192. 10.1007/s10142-025-01674-2 40956482

[313] Uria‐Avellanal, Cristina , and Nicola J. Robertson . 2014. “Na^+^/H^+^ Exchangers and Intracellular pH in Perinatal Brain Injury.” Translational Stroke Research 5: 79–98. 10.1007/s12975-013-0322-x 24452957 PMC3913853

[314] Song, Yejia , John C. Shryock , Stefan Wagner , Lars S. Maier , and Luiz Belardinelli . 2006. “Blocking Late Sodium Current Reduces Hydrogen Peroxide‐Induced Arrhythmogenic Activity and Contractile Dysfunction.” The Journal of Pharmacology and Experimental Therapeutics 318: 214–222. 10.1124/jpet.106.101832 16565163

[315] Castro, J. , I. Ruminot , O. H. Porras , C. M. Flores , T. Hermosilla , E. Verdugo , F. Venegas , et al. 2006. “ATP Steal between Cation Pumps: A Mechanism Linking Na^+^ Influx to the Onset of Necrotic Ca^2+^ Overload.” Cell Death & Differentiation 13: 1675–1685. 10.1038/sj.cdd.4401852 16410794

[316] Inserte, Javier , David Garcia‐Dorado , Victor Hernando , and Jordi Soler‐Soler . 2005. “Calpain‐Mediated Impairment of Na^+^/K^+^–ATPase Activity During Early Reperfusion Contributes to Cell Death After Myocardial Ischemia.” Circulation Research 97: 465–473. 10.1161/01.RES.0000181170.87738.f3 16100049

[317] Pignataro, Giuseppe , Rossana Sirabella , Serenella Anzilotti , Gianfranco Di Renzo , and Lucio Annunziato . 2014. “Does Na^+^/Ca^2+^ Exchanger, NCX, Represent a New Druggable Target in Stroke Intervention?” Translational Stroke Research 5: 145–155. 10.1007/s12975-013-0308-8 24323727

[318] Karwatowska‐Prokopczuk, Ewa , Judith A. Nordberg , Hai Ling Li , Robert L. Engler , and Roberta A. Gottlieb . 1998. “Effect of Vacuolar Proton ATPase on Phi, Ca^2+^, and Apoptosis in Neonatal Cardiomyocytes during Metabolic Inhibition/Recovery.” Circulation Research 82: 1139–1144. 10.1161/01.res.82.11.1139 9633914

[319] Chen, Zhenhuan , Lu Chen , Kaitong Chen , Hairuo Lin , Mengjia Shen , Lin Chen , Hailin Zhu , et al. 2020. “Overexpression of Na^+^‐HCO_3_‐ Cotransporter Contributes to the Exacerbation of Cardiac Remodeling in Mice with Myocardial Infarction by Increasing Intracellular Calcium Overload.” Biochimica et Biophysica Acta (BBA) ‐ Molecular Basis of Disease 1866: 165623. 10.1016/j.bbadis.2019.165623 31778748

[320] Castaldo, P. , M. Cataldi , S. Magi , V. Lariccia , S. Arcangeli , and S. Amoroso . 2009. “Role of the Mitochondrial Sodium/Calcium Exchanger in Neuronal Physiology and in the Pathogenesis of Neurological Diseases.” Progress in Neurobiology 87: 58–79. 10.1016/j.pneurobio.2008.09.017 18952141

[321] Chen, Kerong , Jielei He , Anwei Zhou , Jiayi Zhu , Shiqin Sheng , Zhen Fu , and Xinghai Ning . 2026. “Ultrasound‐Activated Sonophage Synergizes Sonodynamic Therapy and Saltoptosis for Solid Tumor Eradication.” Advanced Materials 38: e08245. 10.1002/adma.202508245 41104711

[322] Yang, K. T. , W. L. Chang , P. C. Yang , C. L. Chien , M. S. Lai , M. J. Su , and M. L. Wu . 2006. “Activation of the Transient Receptor Potential M2 Channel and Poly(ADP‐Ribose) Polymerase Is Involved in Oxidative Stress‐Induced Cardiomyocyte Death.” Cell Death & Differentiation 13: 1815–1826. 10.1038/sj.cdd.4401813 16294211

[323] Zhu, Zitong , Huan Wang , Mengmeng Liu , Yanjun Ji , Anjun Song , Jinsong Ren , and Xiaogang Qu . 2025. “A Sodium Iron Phosphate Nanocatalyst‐Regulated High‐Mobility Ion Reservoir Enables Tumor‐Selective Immunotherapy.” Journal of the American Chemical Society 147: 38958–38972. 10.1021/jacs.5c16505 41084895

[324] Belardinelli, L. , J. C. Shryock , and H. Fraser . 2006. “Inhibition of the Late Sodium Current As a Potential Cardioprotective Principle: Effects of the Late Sodium Current Inhibitor Ranolazine.” Heart 92(Suppl 4): iv6–iv14. 10.1136/hrt.2005.078790 16775092 PMC1861317

[325] Zhu, Yingze , Yaxin Guo , Yanlin Su , Zhuoqi Zhang , Yige Lu , Xianghan Zhang , and Hui Pang . 2025. “Necrosis by Sodium Overload‐Associated Genes TRPM4 and SLC9A1: Biological Roles and Clinical Implications in Breast Cancer Progression.” Frontiers in Immunology 16: 1623511. 10.3389/fimmu.2025.1623511 41235237 PMC12605240

[326] Ji, Yanchao , Bo Yu , Xuedong Yin , Tianqi Yu , Xu Wu , Zongrui Zhao , Zheng Wang , et al. 2026. “Multi‐Omics Profiling of Sodium‐Overload (NECSO) Programs Identifies NEK8 As a Central Driver of Colorectal Cancer Progression Through Single‐Cell and Spatial Transcriptomics.” Frontiers in Immunology 17: 1765055. 10.3389/fimmu.2026.1765055 41743703 PMC12929418

[327] Wang, Fudi , Byung‐Eun Kim , Michael J. Petris , and David J. Eide . 2004. “The Mammalian Zip5 Protein Is a Zinc Transporter That Localizes to the Basolateral Surface of Polarized Cells.” Journal of Biological Chemistry 279: 51433–51441. 10.1074/jbc.M408361200 15322118

[328] Wang, F . 2004. “Acrodermatitis Enteropathica Mutations Affect Transport Activity, Localization and Zinc‐Responsive Trafficking of the Mouse ZIP4 Zinc Transporter.” Human Molecular Genetics 13: 563–571. 10.1093/hmg/ddh049 14709598

[329] Kim, Min‐Hyun , Tolunay B. Aydemir , Jinhee Kim , and Robert J. Cousins . 2017. “Hepatic ZIP14‐Mediated Zinc Transport Is Required for Adaptation to Endoplasmic Reticulum Stress.” Proceedings of the National Academy of Sciences 114: E5805–E5814. 10.1073/pnas.1704012114 PMC5530682 28673968

[330] Lelliott, Emily J. , Jonathan Naddaf , Katherine Ganio , Jessica Michie , Shelly Wang , Lin Liu , Natasha Silke , et al. 2024. “Intracellular Zinc Protects Tumours from T Cell‐Mediated Cytotoxicity.” Cell Death & Differentiation 31: 1707–1716. 10.1038/s41418-024-01369-4 39261596 PMC11618339

[331] Moldoveanu, Tudor , Qian Liu , Ante Tocilj , Mark Watson , Gordon Shore , and Kalle Gehring . 2006. “The X‐Ray Structure of a BAK Homodimer Reveals an Inhibitory Zinc Binding Site.” Molecular Cell 24: 677–688. 10.1016/j.molcel.2006.10.014 17157251

[332] Miyai, Tomohiro , Shintaro Hojyo , Tomokatsu Ikawa , Masami Kawamura , Tarou Irié , Hideki Ogura , Atsushi Hijikata , et al. 2014. “Zinc Transporter SLC39A10/ZIP10 Facilitates Antiapoptotic Signaling during Early B‐Cell Development.” Proceedings of the National Academy of Sciences 111: 11780–11785. 10.1073/pnas.1323549111 PMC4136617 25074913

[333] Lin, Li‐Sen , Jun‐Feng Wang , Jibin Song , Yijing Liu , Guizhi Zhu , Yunlu Dai , Zheyu Shen , et al. 2019. “Cooperation of Endogenous and Exogenous Reactive Oxygen Species Induced by Zinc Peroxide Nanoparticles to Enhance Oxidative Stress‐Based Cancer Therapy.” Theranostics 9: 7200–7209. 10.7150/thno.39831 31695762 PMC6831298

[334] Chen, Youdong , Yujie Lu , Huali Lei , Lin Liu , Xianmin Li , Yuqi Yang , Shumin Sun , et al. 2024. “Zinc‐Nickel Bimetallic Hydroxide Nanosheets Activate the Paraptosis‐Pyroptosis Positive Feedback Cycle for Enhanced Tumor Immunotherapy.” ACS Nano 18: 29913–29929. 10.1021/acsnano.4c10378 39404652

[335] Sun, Yun , Liting Qin , Yuhan Yang , Jingzhe Gao , Yudi Zhang , Hongyu Wang , Qingyuan Wu , Bolong Xu , and Huiyu Liu . 2024. “Zinc‐Based ROS Amplifiers Trigger Cancer Chemodynamic/Ion Interference Therapy Through Self‐Cascade Catalysis.” Small 20: e2402320. 10.1002/smll.202402320 38881259

[336] Wang, Gang , Jingrui Li , Shumin Sun , Yuqi Yang , Zhihui Han , Zifan Pei , and Liang Cheng . 2025. “An Electrically Activable Nanochip to Intensify Gas‐Ionic‐Immunotherapy.” Science Bulletin 70: 390–406. 10.1016/j.scib.2024.11.035 39667986

[337] Chapman, Eric M. , Benjamin Lant , Yota Ohashi , Bin Yu , Michael Schertzberg , Christopher Go , Deepika Dogra , et al. 2019. “A Conserved CCM Complex Promotes Apoptosis Non‐Autonomously by Regulating Zinc Homeostasis.” Nature Communications 10: 1791. 10.1038/s41467-019-09829-z PMC6470173 30996251

[338] Xing, Yanhong , Zhongheng Sui , Yucheng Liu , Meng‐meng Wang , Xiangqing Wei , Qixia Lu , Xinyan Wang , et al. 2022. “Blunting TRPML1 Channels Protects Myocardial Ischemia/Reperfusion Injury by Restoring Impaired Cardiomyocyte Autophagy.” Basic Research in Cardiology 117: 20. 10.1007/s00395-022-00930-x 35389129

[339] Bossy‐Wetzel, Ella , Maria V. Talantova , Wilson D. Lee , Marion N. Schölzke , Anne Harrop , Emily Mathews , Thomas Götz , et al. 2004. “Crosstalk between Nitric Oxide and Zinc Pathways to Neuronal Cell Death Involving Mitochondrial Dysfunction and p38‐activated K+ Channels.” Neuron 41: 351–365. 10.1016/s0896-6273(04)00015-7 14766175

[340] Hu, Rui , Qing Ma , Yunhui Kong , Zhaoyue Wang , Minglu Xu , Xiangyi Chen , Yajuan Su , et al. 2025. “A Compound Screen Based on Isogenic hESC‐Derived Beta Cell Reveals an Inhibitor Targeting ZnT8‐Mediated Zinc Transportation to Protect Pancreatic Beta Cell from Stress‐Induced Cell Death.” Advanced Science 12: e2413161. 10.1002/advs.202413161 40192532 PMC12120731

[341] Frank, Stephan , Brigitte Gaume , Elke S. Bergmann‐Leitner , Wolfgang W. Leitner , Everett G. Robert , Frédéric Catez , Carolyn L. Smith , and Richard J. Youle . 2001. “The Role of Dynamin‐Related Protein 1, a Mediator of Mitochondrial Fission, in Apoptosis.” Developmental Cell 1: 515–525. 10.1016/s1534-5807(01)00055-7 11703942

[342] Shi, Jianjin , Yue Zhao , Kun Wang , Xuyan Shi , Yue Wang , Huanwei Huang , Yinghua Zhuang , et al. 2015. “Cleavage of GSDMD by Inflammatory Caspases Determines Pyroptotic Cell Death.” Nature 526: 660–665. 10.1038/nature15514 26375003

[343] Skrott, Zdenek , Martin Mistrik , Klaus Kaae Andersen , Søren Friis , Dusana Majera , Jan Gursky , Tomas Ozdian , et al. 2017. “Alcohol‐Abuse Drug Disulfiram Targets Cancer Via p97 Segregase Adaptor NPL4.” Nature 552: 194–199. 10.1038/nature25016 29211715 PMC5730499

[344] Qin, Xia , Jun Zhang , Bin Wang , Ge Xu , Xi Yang , Zhen Zou , and Chao Yu . 2021. “Ferritinophagy Is Involved in the Zinc Oxide Nanoparticles‐Induced Ferroptosis of Vascular Endothelial Cells.” Autophagy 17: 4266–4285. 10.1080/15548627.2021.1911016 33843441 PMC8726675

[345] Kejun, Dong , Hu Hao , Cheng Shuangshuang , Mu Yaoqin , Zhang Wei , Zhou Ting , Zhang Jiarui , et al. 2025. “Multifunctional DNA Nano‐Sponge System for Targeted Sensitization of Ovarian Cancer Chemotherapy via Metabolic Reprogramming and Ferroptosis Induction.” Journal of Controlled Release 382: 113663. 10.1016/j.jconrel.2025.113663 40158809

[346] Wang, Zhenxin , Peng Zhou , Yuting Li , Dazhen Zhang , Fuchao Chu , Feng Yuan , Bin Pan , and Fenglei Gao . 2024. “A Bimetallic Polymerization Network for Effective Increase in Labile Iron Pool and Robust Activation of cGAS/STING Induces Ferroptosis‐Based Tumor Immunotherapy.” Small 20: e2308397. 10.1002/smll.202308397 38072786

[347] Qiu, Qian , Jiexin Li , He Ren , Jingyu Zhang , Gengqi Liu , Ruiqi Yang , Boyang Sun , Chen Zhang , and Yumiao Zhang . 2024. “Zinc Coordination Lipid Nanoparticles Co‐Delivering Calcium Peroxide and Chelating STING Agonist for Enhanced Cancer Metalloimmunotherapy.” Small 20: e2402308. 10.1002/smll.202402308 39114869

[348] Liao, Lan‐Shan , Yin Chen , Cheng Hou , Yang‐Han Liu , Gui‐Fa Su , Hong Liang , and Zhen‐Feng Chen . 2023. “Potent Zinc(II)‐Based Immunogenic Cell Death Inducer Triggered by ROS‐Mediated ERS and Mitochondrial Ca^2+^ Overload.” Journal of Medicinal Chemistry 66: 10497–10509. 10.1021/acs.jmedchem.3c00603 37498080

[349] Huang, Yechen , Li Wang , Jie Wu , Xiaoliang Cui , Yuqi Yang , Zifan Pei , Shumin Sun , et al. 2026. “A Powerful Agonist for Metal Ion Interference Therapy: Multiple Programs of Cell Death to Amplify Tumor Metalloimmunotherapy.” Biomaterials 328: 123888. 10.1016/j.biomaterials.2025.123888 41365036

[350] Xia, Zhidan , Xinran Li , Rui Liu , Karin Tuschl , Junxia Min , and Fudi Wang . 2026. “Manganese: Biology, Physiology and Role in Disease.” Cell Discovery 12: 43. 10.1038/s41421-026-00894-5 42303625 PMC13272668

[351] Tuschl, Karin , Peter T. Clayton , Sidney M. Gospe , Shamshad Gulab , Shahnaz Ibrahim , Pratibha Singhi , Roosy Aulakh , et al. 2012. “Syndrome of Hepatic Cirrhosis, Dystonia, Polycythemia, and Hypermanganesemia Caused By Mutations in SLC30A10, a Manganese Transporter in Man.” The American Journal of Human Genetics 90: 457–466. 10.1016/j.ajhg.2012.01.018 22341972 PMC3309187

[352] Lin, Wen , David R. Vann , Paschalis‐Thomas Doulias , Tao Wang , Gavin Landesberg , Xueli Li , Emanuela Ricciotti , et al. 2017. “Hepatic Metal Ion Transporter ZIP8 Regulates Manganese Homeostasis and Manganese‐Dependent Enzyme Activity.” The Journal of Clinical Investigation 127: 2407–2417. 10.1172/JCI90896 28481222 PMC5451243

[353] Choi, Eun‐Kyung , Trang‐Tiffany Nguyen , Neil Gupta , Shigeki Iwase , and Young Ah Seo . 2018. “Functional Analysis of SLC39A8 Mutations and Their Implications for Manganese Deficiency and Mitochondrial Disorders.” Scientific Reports 8: 3163. 10.1038/s41598-018-21464-0 29453449 PMC5816659

[354] Xin, Yongjuan , Hong Gao , Jia Wang , Yuzhen Qiang , Mustapha Umar Imam , Yang Li , Jianyao Wang , et al. 2017. “Manganese Transporter Slc39a14 Deficiency Revealed Its Key Role in Maintaining Manganese Homeostasis in Mice.” Cell Discovery 3: 17025. 10.1038/celldisc.2017.25 28751976 PMC5519003

[355] Tuschl, Karin , Esther Meyer , Leonardo E. Valdivia , Ningning Zhao , Chris Dadswell , Alaa Abdul‐Sada , Christina Y. Hung , et al. 2016. “Mutations in SLC39A14 Disrupt Manganese Homeostasis and Cause Childhood‐Onset Parkinsonism–Dystonia.” Nature Communications 7: 11601. 10.1038/ncomms11601 PMC4894980 27231142

[356] Xia, Zhidan , Biyao Tang , Xiaopeng Li , Xinran Li , Yangfan Jia , Jianwei Jiang , Jingyao Chen , et al. 2024. “A Novel Role for the Longevity‐Associated Protein SLC39A11 As a Manganese Transporter.” Research 7: 0440. 10.34133/research.0440 39114488 PMC11304475

[357] Mercadante, Courtney J. , Milankumar Prajapati , Heather L. Conboy , Miriam E. Dash , Carolina Herrera , Michael A. Pettiglio , Layra Cintron‐Rivera , et al. 2019. “Manganese Transporter Slc30a10 Controls Physiological Manganese Excretion and Toxicity.” The Journal of Clinical Investigation 129: 5442–5461. 10.1172/JCI129710 31527311 PMC6877324

[358] Shen, Xurui , Jinlun Kylian Zhang , Peixin Sun , Huiwen Zhong , Rui He , Shiliang Wang , Xiaojun Guo , and Hanting Yang . 2025. “Molecular Mechanisms of SLC30A10‐mediated Manganese Transport.” Nature Communications 16: 8581. 10.1038/s41467-025-63616-7 PMC12480533 41022720

[359] Wang, Peng , Chen Liang , Jiawei Zhu , Nan Yang , Aihong Jiao , Wenjun Wang , Xuejiao Song , and Xiaochen Dong . 2019. “Manganese‐Based Nanoplatform As Metal Ion‐Enhanced ROS Generator for Combined Chemodynamic/Photodynamic Therapy.” ACS Applied Materials & Interfaces 11: 41140–41147. 10.1021/acsami.9b16617 31603650

[360] Zhuang, Hongjun , Xiaofang He , Huiyan Li , Yang Chen , Tong Wu , Xingwu Jiang , Huilin Zhang , et al. 2023. “MnS Nanocapsule Mediates Mitochondrial Membrane Permeability Transition for Tumor Ion‐Interference Therapy.” ACS Nano 17: 13872–13884. 10.1021/acsnano.3c03670 37458394

[361] Zhang, Zhimin , Jirui Yang , Qiongli Zhou , Shiyin Zhong , Jinghao Luo , Xueting Chai , Jingjing Liu , et al. 2025. “The Role and Mechanism of the cGAS‐STING Pathway‐Mediated ROS in Apoptosis and Ferroptosis Induced By Manganese Exposure.” Redox Biology 85: 103761. 10.1016/j.redox.2025.103761 40652697 PMC12274772

[362] Zhao, Zhiyu , Shuming Dong , Yue Liu , Jianxin Wang , Li Ba , Cong Zhang , Xinyu Cao , Changjun Wu , and Piaoping Yang . 2022. “Tumor Microenvironment‐Activable Manganese‐Boosted Catalytic Immunotherapy Combined with PD‐1 Checkpoint Blockade.” ACS Nano 16: 20400–20418. 10.1021/acsnano.2c06646 36441901

[363] Gao, Nan , Yiran Huang , Shisuo Jing , Meng Zhang , Ergang Liu , Lu Qiu , Jing Huang , Bahtiyor Muhitdinov , and Yongzhuo Huang . 2024. “Environment‐Responsive Dendrobium Polysaccharide Hydrogel Embedding Manganese Microsphere As a Post‐Operative Adjuvant to Boost Cascaded Immune Cycle against Melanoma.” Theranostics 14: 3810–3826. 10.7150/thno.94354 38994034 PMC11234272

[364] Kamer, Kimberli J. , Yasemin Sancak , Yevgenia Fomina , Joshua D Meisel , Dipayan Chaudhuri , Zenon Grabarek , and Vamsi K. Mootha . 2018. “MICU1 Imparts the Mitochondrial Uniporter with the Ability to Discriminate between Ca^2+^ and Mn^2+^ .” Proceedings of the National Academy of Sciences 115: E7960–E7969. 10.1073/pnas.1807811115 PMC6112746 30082385

[365] Yang, Yilin , Ning Wang , Zhihua Wang , Fei Yan , Zhan Shi , and Shouhua Feng . 2025. “Glutathione‐Responsive Metal‐Organic‐Framework‐Derived Mn_x_O_y_/(A/R)TiO_2_ Nanoparticles for Enhanced Synergistic Sonodynamic/Chemodynamic/Immunotherapy.” ACS Nano 19: 885–899. 10.1021/acsnano.4c12304 39752569

[366] Yang, Pengfei , Jie Zhang , Guanglei Ma , Songsong Zhi , Yi Chang , Lina Ding , Xiaoming Ma , and Yuming Guo . 2026. “Multifunctional Self‐Cascade Nanoplatform for Efficient Immunotherapy through Synergizing Pyroptosis and Self‐Augmented Activation of cGAS‐STING Pathway.” Journal of Colloid and Interface Science 704: 139423. 10.1016/j.jcis.2025.139423 41218499

[367] Qiao, Wen , Jingqi Chen , Huayuan Zhou , Cegui Hu , Sumiya Dalangood , Hanjun Li , Dandan Yang , Yu Yang , and Jun Gui . 2024. “A Single‐Atom Manganese Nanozyme Mn‐N/C Promotes Anti‐Tumor Immune Response Via Eliciting Type I Interferon Signaling.” Advanced Science 11: e2305979. 10.1002/advs.202305979 38308189 PMC11005736

[368] Liu, Junjie , Xiu Zhao , Weimin Nie , Yue Yang , Chengcheng Wu , Wei Liu , Kaixiang Zhang , Zhenzhong Zhang , and Jinjin Shi . 2021. “Tumor Cell‐Activated ‘Sustainable ROS Generator’ With Homogeneous Intratumoral Distribution Property for Improved Anti‐Tumor Therapy.” Theranostics 11: 379–396. 10.7150/thno.50028 33391481 PMC7681092

[369] Lin, Qian , Yueyang He , Yang Li , Qiuyue Sun , Fu‐Nan Li , Jinyan Lin , and Xuan Zhu . 2025. “Tumor‐Microenvironment‐Driven Carbon‐Center Radical Generation Accompanied by Glutathione Exhaustion for Intensified Chemodynamic Therapy.” Journal of Colloid and Interface Science 692: 137545. 10.1016/j.jcis.2025.137545 40228460

[370] Wang, Shuaifei , Fangyuan Li , Ruirui Qiao , Xi Hu , Hongwei Liao , Lumin Chen , Jiahe Wu , et al. 2018. “Arginine‐Rich Manganese Silicate Nanobubbles as a Ferroptosis‐Inducing Agent for Tumor‐Targeted Theranostics.” ACS Nano 12: 12380–12392. 10.1021/acsnano.8b06399 30495919

[371] Chang, Mingyu , Jingcheng Lv , Lianhui Sun , Aihong Chen , Fengbo Zhang , Guangjian Fan , Zhanliang Liu , et al. 2025. “Cu‐Mn Bimetallic Mesoporous Silica Nanosonosensitizers Enable Oxeiptosis‐Mediated Sonodynamic Therapy for Ultra‐Minimally Invasive Treatment of Benign Prostatic Hyperplasia.” ACS Applied Materials & Interfaces 17: 51820–51839. 10.1021/acsami.5c12846 40911431 PMC12447393

[372] Wu, Chuan , Mingquan Gao , Weidong Xiao , Xie Huang , Xinrui Yang , Zifei Wu , Xudong Yu , et al. 2025. “Light‐Activatable Manganese Carbonate Nanocubes Elicit Robust Immunotherapy by Amplifying Endoplasmic Reticulum Stress‐Meditated Pyroptotic Cell Death.” Journal of Experimental & Clinical Cancer Research 44: 147. 10.1186/s13046-025-03408-5 40380194 PMC12082914

[373] Wang, Peng , Yinfeng Wang , Huimin Li , Miaomiao Wang , Yue Wang , Xiaofei Wang , Lang Ran , et al. 2024. “A Homologous‐Targeting cGAS‐STING Agonist Multimodally Activates Dendritic Cells for Enhanced Cancer Immunotherapy.” Acta Biomaterialia 177: 400–413. 10.1016/j.actbio.2024.02.003 38336268

[374] Song, Qinghang , Yuxuan Yang , and Lina Yang . 2025. “Navigating Transition Metal‐Dependent Cell Death: Mechanisms, Crosstalk, and Future Directions.” Advanced Science 12: e01974. 10.1002/advs.202501974 41178545 PMC12697847

[375] Lin, Xuexin , Xuexin , Liling Li , Jia Luo , Dan Chen , Jingqian Tan , and Peng Li . 2024. “Cobalt‐Induced Apoptosis of Cochlear Organotypic Cultures and HEI‐OC1 Cells Is Mediated by Dicer.” NeuroToxicology 100: 85–99. 10.1016/j.neuro.2023.12.009 38101458

[376] Ranquet, Caroline , Sandrine Ollagnier‐De‐Choudens , Laurent Loiseau , Frédéric Barras , and Marc Fontecave . 2007. “Cobalt Stress in Escherichia Coli.” Journal of Biological Chemistry 282: 30442–30451. 10.1074/jbc.M702519200 17642475

[377] Osman, Deenah , Andrew W. Foster , Junjun Chen , Kotryna Svedaite , Jonathan W. Steed , Elena Lurie‐Luke , Thomas G. Huggins , and Nigel J. Robinson . 2017. “Fine Control of Metal Concentrations Is Necessary for Cells to Discern Zinc from Cobalt.” Nature Communications 8: 1884. 10.1038/s41467-017-02085-z PMC5709419 29192165

[378] Huk, Olga L. , Isabelle Catelas , Fackson Mwale , John Antoniou , David J. Zukor , and Alain Petit . 2004. “Induction of Apoptosis and Necrosis by Metal Ions In Vitro.” The Journal of Arthroplasty 19: 84–87. 10.1016/j.arth.2004.09.011 15578559

[379] Petit, Alain , Fackson Mwale , David J. Zukor , Isabelle Catelas , John Antoniou , and Olga L. Huk . 2004. “Effect of Cobalt and Chromium Ions on bcl‐2, Bax, Caspase‐3, and Caspase‐8 Expression in Human U937 Macrophages.” Biomaterials 25: 2013–2018. 10.1016/j.biomaterials.2003.08.040 14741615

[380] Catelas, Isabelle , Alain Petit , David J. Zukor , John Antoniou , Olga L. Huk . 2003. “TNF‐α Secretion and Macrophage Mortality Induced by Cobalt and Chromium Ions In Vitro‐Qualitative Analysis of Apoptosis.” Biomaterials 24: 383–391. 10.1016/s0142-9612(02)00351-4 12423593

[381] Zhu, Minghui , Shihang Xu , Guochao Li , Gang Xu , Zhenlei Zhang , Hong Liang , and Feng Yang . 2025. “Development of a High‐Efficacy and Low‐Toxicity Cobalt(II) Agent for Targeting Inhibition of Tumor Growth through Mitochondrial Damage‐Mediated Chemotherapy and Immunotherapy.” Journal of Medicinal Chemistry 68: 13113–13126. 10.1021/acs.jmedchem.5c01235 40500876

[382] He, Yuting , Xueqi Gan , Ling Zhang , Beilei Liu , Zhuoli Zhu , Tao Li , Junfei Zhu , Junsheng Chen , and Haiyang Yu . 2018. “CoCl_2_ Induces Apoptosis via a ROS‐dependent Pathway and Drp1‐mediated Mitochondria Fission in Periodontal Ligament Stem Cells.” American Journal of Physiology‐Cell Physiology 315: C389–C397. 10.1152/ajpcell.00248.2017 29768044

[383] Karovic, Olga , Ilaria Tonazzini , Nelson Rebola , Erik Edström , Cecilia Lövdahl , Bertil B. Fredholm , and Elisabetta Daré . 2007. “Toxic Effects of Cobalt in Primary Cultures of Mouse Astrocytes.” Biochemical Pharmacology 73: 694–708. 10.1016/j.bcp.2006.11.008 17169330

[384] Yuan, Yong , George Hilliard , Tsuneo Ferguson , and David E. Millhorn . 2003. “Cobalt Inhibits the Interaction between Hypoxia‐Inducible factor‐α and Von Hippel‐Lindau Protein by Direct Binding to Hypoxia‐Inducible Factor‐α.” Journal of Biological Chemistry 278: 15911–15916. 10.1074/jbc.M300463200 12606543

[385] Muñoz‐Sánchez, Jorge , and María E. Chánez‐Cárdenas . 2019. “The Use of Cobalt Chloride as a Chemical Hypoxia Model.” Journal of Applied Toxicology 39: 556–570. 10.1002/jat.3749 30484873

[386] Lee, Yuan‐Wen , Yih‐Giun Cherng , Shun‐Tai Yang , Shing‐Hwa Liu , Ta‐Liang Chen , and Ruei‐Ming Chen . 2021. “Hypoxia Induced by Cobalt Chloride Triggers Autophagic Apoptosis of Human and Mouse Drug‐Resistant Glioblastoma Cells Through Targeting the PI3K‐AKT‐mTOR Signaling Pathway.” Oxidative Medicine and Cellular Longevity 2021: 5558618. 10.1155/2021/5558618 34136065 PMC8177987

[387] Song, Z.‐C. , W. Zhou , R. Shu , and J. Ni . 2012. “Hypoxia Induces Apoptosis and Autophagic Cell Death in Human Periodontal Ligament Cells ThroughHIF‐1α Pathway.” Cell Proliferation 45: 239–248. 10.1111/j.1365-2184.2012.00810.x 22429763 PMC6496313

[388] Su, Qianqian , Lingyan Wu , Chunyan Zheng , Xianqi Ji , Xinpei Lin , Yu Zhang , Fuli Zheng , et al. 2024. “ALKBH5‐Mediated N6‐Methyladenosine Modification of HO‐1 mRNA Regulates Ferroptosis in Cobalt‐Induced Neurodegenerative Damage.” Environment International 190: 108897. 10.1016/j.envint.2024.108897 39047545

[389] Tang, Jianping , Qianqian Su , Zhenkun Guo , Jinfu Zhou , Fuli Zheng , Guangxia Yu , Wenya Shao , et al. 2022. “N6‐methyladenosine(m(6)A) Demethylase FTO Regulates Cellular Apoptosis Following Cobalt‐Induced Oxidative Stress.” Environmental Pollution 297: 118749. 10.1016/j.envpol.2021.118749 34968619

[390] Alajroush, Duaa R. , Chloe B. Smith , Brittney F. Anderson , Ifeoluwa T. Oyeyemi , Stephen J. Beebe , and Alvin A. Holder . 2024. “A Comparison of In Vitro Studies between Cobalt(III) and Copper(II) Complexes With Thiosemicarbazone Ligands to Treat Triple Negative Breast Cancer.” Inorganica Chimica Acta 562: 121898. 10.1016/j.ica.2023.121898 38282819 PMC10810091

[391] Montesdeoca, Nicolás , Lukas Johannknecht , Elizaveta Efanova , Jacqueline Heinen‐Weiler , and Johannes Karges . 2024. “Ferroptosis Inducing Co(III) Polypyridine Sulfasalazine Complex for Therapeutically Enhanced Anticancer Therapy.” Angewandte Chemie International Edition 63: e202412585. 10.1002/anie.202412585 39136323

[392] Lu, Xiuxin , Yang Zheng , Yan Liu , Dan Li , Jiaxin Lin , Lineng Wei , Song Gao , et al. 2024. “Orchestrating Apoptosis and Ferroptosis Through Enhanced Sonodynamic Therapy Using Amorphous UIO‐66‐CoO(x).” Journal of Colloid and Interface Science 667: 91–100. 10.1016/j.jcis.2024.04.064 38621335

[393] Zhao, Jianqi , Yin Chen , Tainong Xiong , Songling Han , Chenwenya Li , Yingjuan He , Yongwu He , et al. 2023. “Clustered Cobalt Nanodots Initiate Ferroptosis by Upregulating Heme Oxygenase 1 for Radiotherapy Sensitization.” Small 19: e2206415. 10.1002/smll.202206415 36627264

[394] Park, Chan Ho , Jun Young Park , and Won Gil Cho . 2024. “Chemical Hypoxia Induces Pyroptosis in Neuronal Cells by Caspase‐Dependent Gasdermin Activation.” International Journal of Molecular Sciences 25: 2185. 10.3390/ijms25042185 38396860 PMC10889762

[395] Gu, Chenglu , Dongmei Wang , Shuang Zhu , Xue Wang , Xinyi Tian , You Liao , and Zhanjun Gu . 2025. “A Pyroptosis Radiosensitizer Facilitates Hypoxic Tumor Necrosis.” Small 21: e2409594. 10.1002/smll.202409594 39989228

[396] Liu, Wenna , Yujin Gan , Yun Ding , Lina Zhang , Xiaojing Jiao , Lu Liu , Huixia Cao , et al. 2022. “Autophagy Promotes GSDME‐mediated Pyroptosis Via Intrinsic and Extrinsic Apoptotic Pathways in Cobalt Chloride‐Induced Hypoxia Reoxygenation‐Acute Kidney Injury.” Ecotoxicology and Environmental Safety 242: 113881. 10.1016/j.ecoenv.2022.113881 35863214

[397] Aschner, Michael , Anatoly V. Skalny , Airton C. Martins , Yousef Tizabi , Irina P. Zaitseva , Abel Santamaria , Rongzhu Lu , Yordanka Y. Gluhcheva , and Alexey A. Tinkov . 2025. “The Role of NLRP3 Inflammasome Activation in Proinflammatory and Cytotoxic Effects of Metal Nanoparticles.” Archives of Toxicology 99: 1287–1314. 10.1007/s00204-025-03972-x 39960653

[398] Du, Silin , Guangyu Ma , Xiang Li , Minghao Chao , Liying Fan , Zhiqiang Zhao , Rongze Tang , et al. 2026. “Targeting ROS‐metabolism Dual Pathways to Trigger PANoptosis by Cobalt–Vanadium Oxides Biomimetic Nanocakes for Tumor Immunotherapy.” Biomaterials 326: 123726. 10.1016/j.biomaterials.2025.123726 40987137

[399] Gupta, Govind , Anda Gliga , Jonas Hedberg , Angela Serra , Dario Greco , Inger Odnevall Wallinder , et al. 2020. “Cobalt Nanoparticles Trigger Ferroptosis‐Like Cell Death (Oxytosis) in Neuronal Cells: Potential Implications for Neurodegenerative Disease.” The FASEB Journal 34: 5262–5281. 10.1096/fj.201902191RR 32060981

[400] Liu, Yake , Wenfeng Zhu , Dalong Ni , Zihua Zhou , Jin‐hua Gu , Weinan Zhang , Huanjian Sun , and Fan Liu . 2020. “Alpha Lipoic Acid Antagonizes Cytotoxicity of Cobalt Nanoparticles by Inhibiting Ferroptosis‐Like Cell Death.” Journal of Nanobiotechnology 18: 141. 10.1186/s12951-020-00700-8 33008409 PMC7532644

[401] Khalil, Samah R. , Khlood M. El Bohi , Safaa Khater , Amir H. Abd El‐fattah , Fagr A. Mahmoud , and Mayada R. Farag . 2020. “Moringa Oleifera Leaves Ethanolic Extract Influences DNA Damage Signaling Pathways to Protect Liver Tissue from Cobalt ‐Triggered Apoptosis in Rats.” Ecotoxicology and Environmental Safety 200: 110716. 10.1016/j.ecoenv.2020.110716 32450433

[402] Yamamoto, Natsuho , Anna K. Renfrew , Byung J. Kim , Nicole S. Bryce , and Trevor W. Hambley . 2012. “Dual Targeting of Hypoxic and Acidic Tumor Environments with a Cobalt(III) Chaperone Complex.” Journal of Medicinal Chemistry 55: 11013–11021. 10.1021/jm3014713 23199008

[403] Long, Chuan , Han Peng , Wei Yang , Min Wang , Bo Luo , Jie Hao , Yan Dong , and Wenwei Zuo . 2024. “Targeted Delivery of Gemcitabine for Precision Therapy of Cholangiocarcinoma Using Hyaluronic Acid‐Modified Metal–Organic Framework Nanoparticles.” ACS Omega 9: 11998–12005. 10.1021/acsomega.3c09751 38496964 PMC10938583

[404] Shang, Kun , Nicolás Montesdeoca , Hanchen Zhang , Elizaveta Efanova , Ganghao Liang , Jasmine Ochs , Johannes Karges , Haiqin Song , and Lingpu Zhang . 2024. “Cobalt(III) Prodrug‐Based Nanomedicine for Inducing Immunogenic Cell Death and Enhancing Chemo‐Immunotherapy.” Journal of Controlled Release 373: 493–506. 10.1016/j.jconrel.2024.07.042 39033985

[405] Yu, Qiao , Shumin Sun , Nailin Yang , Zifan Pei , Youdong Chen , Jihu Nie , Huali Lei , et al. 2025. “Self‐Cascaded Pyroptosis‐STING Initiators for Catalytic Metalloimmunotherapy.” Journal of the American Chemical Society 147: 3161–3173. 10.1021/jacs.4c12552 39818788

[406] Yang, Fan , Guoxia Zhang , Na An , Qianqian Dai , William Cho , Hongcai Shang , and Yanwei Xing . 2024. “Interplay of Ferroptosis, Cuproptosis, and PANoptosis in Cancer Treatment‐Induced Cardiotoxicity: Mechanisms and Therapeutic Implications.” Seminars in Cancer Biology 106–107: 106–122. 10.1016/j.semcancer.2024.09.003 39299410

[407] Wang, Yaqiu , and Thirumala‐Devi Kanneganti . 2021. “From Pyroptosis, Apoptosis and Necroptosis to PANoptosis: A Mechanistic Compendium of Programmed Cell Death Pathways.” Computational and Structural Biotechnology Journal 19: 4641–4657. 10.1016/j.csbj.2021.07.038 34504660 PMC8405902

[408] Lee, SangJoon , Rajendra Karki , Yaqiu Wang , Lam Nhat Nguyen , Ravi C. Kalathur , and Thirumala‐Devi Kanneganti . 2021. “AIM2 Forms a Complex With Pyrin and ZBP1 to Drive PANoptosis and Host Defence.” Nature 597: 415–419. 10.1038/s41586-021-03875-8 34471287 PMC8603942

[409] Zhang, Xiaojie , Bufu Tang , Jinhua Luo , Yang Yang , Qiaoyou Weng , Shiji Fang , Zhongwei Zhao , et al. 2024. “Cuproptosis, Ferroptosis and PANoptosis in Tumor Immune Microenvironment Remodeling and Immunotherapy: Culprits or New Hope.” Molecular Cancer 23: 255. 10.1186/s12943-024-02130-8 39543600 PMC11566504

[410] Yun, Kaiqing , Xiaohong Yu , Shuang Liang , Qingling Wang , Ziyi Zhang , Yue Han , Yueyang Zhao , et al. 2025. “Tumor‐Responsive Cuproptosis Nanoinducer Realizing Efficient PANoptosis for Enhanced Cancer Immunotherapy.” Theranostics 15: 9294–9305. 10.7150/thno.115275 40963914 PMC12439466

[411] Dong, Shuohui , Haolin Cao , Ye Yuan , Shuo Liang , Zhendong Fu , Wei Shi , Qian Xu , et al. 2025. “A Novel ‘Three‐in‐One’ Copper‐Based Metal‐Organic Framework Nanozyme Eradicates Colorectal Cancer and Overcomes Chemoresistance for Tumor Therapy.” Advanced Science 12: e2413422. 10.1002/advs.202413422 39629925 PMC11809406

[412] Chen, Jinfeng , Ziqi Jin , Shuqing Zhang , Xiao Zhang , Peipei Li , Heng Yang , and Yuting Ma . 2023. “Arsenic Trioxide Elicits Prophylactic and Therapeutic Immune Responses Against Solid Tumors by Inducing Necroptosis and Ferroptosis.” Cellular & Molecular Immunology 20: 51–64. 10.1038/s41423-022-00956-0 36447031 PMC9794749

[413] Meng, Xiangqin , Dandan Li , Lei Chen , Helen He , Qian Wang , Chaoyi Hong , Jiuyang He , et al. 2021. “High‐Performance Self‐Cascade Pyrite Nanozymes for Apoptosis‐Ferroptosis Synergistic Tumor Therapy.” ACS Nano 15: 5735–5751. 10.1021/acsnano.1c01248 33705663

[414] Yan, Shuangqian , Panpan Xue , Ying Sun , Tingjie Bai , Sijie Shao , and Xuemei Zeng . 2025. “Cupric Doping Hollow Prussian Blue Nanoplatform for Enhanced Cholesterol Depletion: a Promising Strategy for Breast Cancer Therapy and Metastasis Inhibition.” Advanced Science 12: e2409967. 10.1002/advs.202409967 39606805 PMC11744725

[415] Hou, Guanghui , Youdong Chen , Huali Lei , Yujie Lu , Lin Liu , Zhihui Han , Shumin Sun , Jingrui Li , and Liang Cheng . 2024. “Bimetallic Peroxide Nanoparticles Induce PANoptosis by Disrupting Ion Homeostasis for Enhanced Immunotherapy.” Science Advances 10: eadp7160. 10.1126/sciadv.adp7160 39514658 PMC11546811

[416] Sundaram, Balamurugan , Nagakannan Pandian , Raghvendra Mall , Yaqiu Wang , Roman Sarkar , Hee Jin Kim , et al. 2023. “NLRP12‐PANoptosome Activates PANoptosis and Pathology in Response to Heme and PAMPs.” Cell 186: 2783–2801.e20. 10.1016/j.cell.2023.05.005 37267949 PMC10330523

[417] Meng, Xuan , Jian Deng , Fang Liu , Tao Guo , Mengying Liu , Peipei Dai , Aiping Fan , Zheng Wang , and Yanjun Zhao . 2019. “Triggered All‐Active Metal Organic Framework: Ferroptosis Machinery Contributes to the Apoptotic Photodynamic Antitumor Therapy.” Nano Letters 19: 7866–7876. 10.1021/acs.nanolett.9b02904 31594301

[418] Guo, Zhenhu , Xiaohan Gao , Jingsong Lu , Ying Li , Zeping Jin , Abdul Fahad , Neema Ufurahi Pambe , et al. 2024. “Apoptosis and Paraptosis Induced by Disulfiram‐Loaded Ca^2+^/Cu^2+^ Dual‐Ions Nano Trap for Breast Cancer Treatment.” ACS Nano 18: 6975–6989. 10.1021/acsnano.3c10173 38377439

[419] Bi, Xukun , Xiaotian Wu , Jiaqi Chen , Xiaoting Li , Yangjun Lin , Yingying Yu , Xuexian Fang , et al. 2024. “Characterization of Ferroptosis‐Triggered Pyroptotic Signaling in Heart Failure.” Signal Transduction and Targeted Therapy 9: 257. 10.1038/s41392-024-01962-6 39327446 PMC11427671

[420] Zhou, Bo , Jia‐yuan Zhang , Xian‐shuo Liu , Hang‐zi Chen , Yuan‐li Ai , Kang Cheng , Ru‐yue Sun , et al. 2018. “Tom20 Senses Iron‐Activated ROS Signaling to Promote Melanoma Cell Pyroptosis.” Cell Research 28: 1171–1185. 10.1038/s41422-018-0090-y 30287942 PMC6274649

[421] Zheng, Pan , Binbin Ding , Zhongyu Jiang , Weiguo Xu , Gao Li , Jianxun Ding , and Xuesi Chen . 2021. “Ultrasound‐Augmented Mitochondrial Calcium Ion Overload by Calcium Nanomodulator to Induce Immunogenic Cell Death.” Nano Letters 21: 2088–2093. 10.1021/acs.nanolett.0c04778 33596078

[422] Song, Ge , Minghui Li , Shumin Fan , Mengmeng Qin , Bin Shao , Wenbing Dai , Hua Zhang , et al. 2024. “Boosting Synergism of Chemo‐ and Immuno‐Therapies via Switching Paclitaxel‐Induced Apoptosis to Mevalonate Metabolism‐Triggered Ferroptosis by Bisphosphonate Coordination Lipid Nanogranules.” Acta Pharmaceutica Sinica B 14: 836–853. 10.1016/j.apsb.2023.08.029 38322346 PMC10840482

[423] Xu, Rong , Li‐sha Yuan , Ying‐qing Gan , Na Lu , Ya‐ping Li , Zhi‐ya Zhou , Qing‐bing Zha , et al. 2024. “Potassium Ion Efflux Induces Exaggerated Mitochondrial Damage and Non‐Pyroptotic Necrosis When Energy Metabolism Is Blocked.” Free Radical Biology and Medicine 212: 117–132. 10.1016/j.freeradbiomed.2023.12.029 38151213

[424] Zhen, Wenyao , Yingjie Fan , Tomas Germanas , Langston Tillman , Jinhong Li , Abigail L. Blenko , Ralph R. Weichselbaum , Ralph R. , Weichselbaum , and Wenbin Lin . 2025. “Digitonin‐Loaded Nanoscale Metal‐Organic Framework for Mitochondria‐Targeted Radiotherapy‐Radiodynamic Therapy and Disulfidptosis.” Advanced Materials 37: e2405494. 10.1002/adma.202405494 39252688 PMC11891090

[425] Yang, Minghua , Pan Chen , Jiao Liu , Shan Zhu , Guido Kroemer , Daniel J. Klionsky , Michael T. Lotze , et al. 2019. “Clockophagy Is a Novel Selective Autophagy Process Favoring Ferroptosis.” Science Advances 5: eaaw2238. 10.1126/sciadv.aaw2238 31355331 PMC6656546

[426] Salgado, Magdiel , Valeria Márquez‐Miranda , Luciano Ferrada , Maximiliano Rojas , Gonzalo Poblete‐Flores , Fernando D. González‐Nilo , Álvaro O. Ardiles , and Juan C. Sáez . 2024. “Ca^2+^ Permeation Through C‐Terminal Cleaved, but Not Full‐Length Human Pannexin1 Hemichannels, Mediates Cell Death.” Proceedings of the National Academy of Sciences 121: e2405468121. 10.1073/pnas.2405468121 PMC11194574 38861601

[427] Yoshida, Masahiro , Shunsuke Minagawa , Jun Araya , Taro Sakamoto , Hiromichi Hara , Kazuya Tsubouchi , Yusuke Hosaka , et al. 2019. “Involvement of Cigarette Smoke‐Induced Epithelial Cell Ferroptosis in COPD Pathogenesis.” Nature Communications 10: 3145. 10.1038/s41467-019-10991-7 PMC6637122 31316058

[428] Rapino, Francesca , Sylvain Delaunay , Florian Rambow , Zhaoli Zhou , Lars Tharun , Pascal De Tullio , Olga Sin , et al. 2018. “Codon‐Specific Translation Reprogramming Promotes Resistance to Targeted Therapy.” Nature 558: 605–609. 10.1038/s41586-018-0243-7 29925953

[429] Chen, Yu‐Ying , Yu‐Hsuan Lee , Bour‐Jr Wang , Rong‐Jane Chen , and Ying‐Jan Wang . 2022. “Skin Damage Induced by Zinc Oxide Nanoparticles Combined With UVB Is Mediated by Activating Cell Pyroptosis via the NLRP3 Inflammasome–Autophagy–Exosomal Pathway.” Particle and Fibre Toxicology 19: 2. 10.1186/s12989-021-00443-w 34983566 PMC8729117

[430] Letai, Anthony . 2017. “Functional Precision Cancer Medicine—Moving Beyond Pure Genomics.” Nature Medicine 23: 1028–1035. 10.1038/nm.4389 28886003

[431] Murphy, Michael P. , Hülya Bayir , Vsevolod Belousov , Christopher J. Chang , Kelvin J. A. Davies , Michael J. Davies , Tobias P. Dick , et al. 2022. “Guidelines for Measuring Reactive Oxygen Species and Oxidative Damage in Cells and In Vivo.” Nature Metabolism 4: 651–662. 10.1038/s42255-022-00591-z PMC9711940 35760871

[432] Qiao, Luying , Guoqing Zhu , Tengfei Jiang , Yanrong Qian , Qianqian Sun , Guanghui Zhao , Haidong Gao , and Chunxia Li . 2024. “Self‐Destructive Copper Carriers Induce Pyroptosis and Cuproptosis for Efficient Tumor Immunotherapy Against Dormant and Recurrent Tumors.” Advanced Materials 36: e2308241. 10.1002/adma.202308241 37820717

[433] Feng, Zhenzhen , Gui Chen , Min Zhong , Ling Lin , Ziyi Mai , Yan Tang , Guimei Chen , et al. 2023. “An Acid‐Responsive MOF Nanomedicine for Augmented Anti‐Tumor Immunotherapy via a Metal Ion Interference‐Mediated Pyroptotic Pathway.” Biomaterials 302: 122333. 10.1016/j.biomaterials.2023.122333 37738743

[434] Malireddi, R. K. Subbarao , Rajendra Karki , Balamurugan Sundaram , Balabhaskararao Kancharana , SangJoon Lee , Parimal Samir , and Thirumala‐Devi Kanneganti . 2021. “Inflammatory Cell Death, PANoptosis, Mediated by Cytokines in Diverse Cancer Lineages Inhibits Tumor Growth.” ImmunoHorizons 5: 568–580. 10.4049/immunohorizons.2100059 34290111 PMC8522052

[435] Sun, Xiaoqi , Yu Zhang , Jiaqian Li , Kyung Soo Park , Kai Han , Xingwu Zhou , Yao Xu , et al. 2021. “Amplifying STING Activation by Cyclic Dinucleotide‐Manganese Particles for Local and Systemic Cancer Metalloimmunotherapy.” Nature Nanotechnology 16: 1260–1270. 10.1038/s41565-021-00962-9 PMC8595610 34594005

[436] Skouta, Rachid , Scott J. Dixon , Jianlin Wang , Denise E. Dunn , Marina Orman , Kenichi Shimada , Paul A. Rosenberg , et al. 2014. “Ferrostatins Inhibit Oxidative Lipid Damage and Cell Death in Diverse Disease Models.” Journal of the American Chemical Society 136: 4551–4556. 10.1021/ja411006a 24592866 PMC3985476

[437] Freire Boullosa, Laurie , Jinthe Van Loenhout , Tal Flieswasser , Jorrit De Waele , Christophe Hermans , Hilde Lambrechts , Bart Cuypers , et al. 2021. “Auranofin Reveals Therapeutic Anticancer Potential by Triggering Distinct Molecular Cell Death Mechanisms and Innate Immunity in Mutant p53 Non‐Small Cell Lung Cancer.” Redox Biology 42: 101949. 10.1016/j.redox.2021.101949 33812801 PMC8113045

[438] Gao, Minghui , Prashant Monian , Nosirudeen Quadri , Ravichandran Ramasamy , and Xuejun Jiang . 2015. “Glutaminolysis and Transferrin Regulate Ferroptosis.” Molecular Cell 59: 298–308. 10.1016/j.molcel.2015.06.011 26166707 PMC4506736

[439] Trachootham, Dunyaporn , Jerome Alexandre , and Peng Huang . 2009. “Targeting Cancer Cells by ROS‐mediated Mechanisms: A Radical Therapeutic Approach?” Nature Reviews Drug Discovery 8: 579–591. 10.1038/nrd2803 19478820

[440] Brashears, Caitlyn B. , Bethany C. Prudner , Richa Rathore , Katharine E. Caldwell , Carina A. Dehner , Jane L. Buchanan , Sara E. S. Lange , et al. 2022. “Malic Enzyme 1 Absence in Synovial Sarcoma Shifts Antioxidant System Dependence and Increases Sensitivity to Ferroptosis Induction With ACXT‐3102.” Clinical Cancer Research 28: 3573–3589. 10.1158/1078-0432.CCR-22-0470 35421237 PMC9378556

[441] Dolma, Sonam , Stephen L. Lessnick , William C. Hahn , and Brent R. Stockwell . 2003. “Identification of Genotype‐Selective Antitumor Agents Using Synthetic Lethal Chemical Screening in Engineered Human Tumor Cells.” Cancer Cell 3: 285–296. 10.1016/S1535-6108(03)00050-3 12676586

[442] Yang, Wan Seok , and Brent R. Stockwell . 2008. “Synthetic Lethal Screening Identifies Compounds Activating Iron‐Dependent, Nonapoptotic Cell Death in Oncogenic‐RAS‐Harboring Cancer Cells.” Chemistry & Biology 15: 234–245. 10.1016/j.chembiol.2008.02.010 18355723 PMC2683762

[443] Yagoda, Nicholas , Moritz von Rechenberg , Elma Zaganjor , Andras J. Bauer , Wan Seok Yang , Daniel J. Fridman , Adam J. Wolpaw , et al. 2007. “RAS–RAF–MEK‐Dependent Oxidative Cell Death Involving Voltage‐Dependent Anion Channels.” Nature 447: 865–869. 10.1038/nature05859 PMC3047570 17568748

[444] Ou, Yang , Shang‐Jui Wang , Dawei Li , Bo Chu , and Wei Gu . 2016. “Activation of SAT1 Engages Polyamine Metabolism With p53‐Mediated Ferroptotic Responses.” Proceedings of the National Academy of Sciences 113: E6806–E6812. 10.1073/pnas.1607152113 PMC5098629 27698118

[445] Chu, Bo , Ning Kon , Delin Chen , Tongyuan Li , Tong Liu , Le Jiang , Shujuan Song , Omid Tavana , and Wei Gu . 2019. “ALOX12 Is Required for p53‐Mediated Tumour Suppression Through a Distinct Ferroptosis Pathway.” Nature Cell Biology 21: 579–591. 10.1038/s41556-019-0305-6 30962574 PMC6624840

[446] Kang, Yun Pyo , Andrea Mockabee‐Macias , Chang Jiang , Aimee Falzone , Nicolas Prieto‐Farigua , Everett Stone , Isaac S. Harris , and Gina M. DeNicola . 2021. “Non‐Canonical Glutamate‐Cysteine Ligase Activity Protects Against Ferroptosis.” Cell Metabolism 33: 174–189.e7. 10.1016/j.cmet.2020.12.007 33357455 PMC7839835

[447] Maeda, H. , S. Hori , H. Ohizumi , T. Segawa , Y. Kakehi , O. Ogawa , and A. Kakizuka . 2004. “Effective Treatment of Advanced Solid Tumors by the Combination of Arsenic Trioxide and L‐Buthionine‐Sulfoximine.” Cell Death & Differentiation 11: 737–746. 10.1038/sj.cdd.4401389 15002036

[448] Zhang, Yang , Yi Xu , Wenyun Lu , Jinyang Li , Sixiang Yu , Eric J. Brown , Ben Z. Stanger , Joshua D. Rabinowitz , and Yang Xiaolu . 2022. “G6PD‐mediated Increase in De Novo NADP(+) Biosynthesis Promotes Antioxidant Defense and Tumor Metastasis.” Science Advances 8: eabo0404. 10.1126/sciadv.abo0404 35857842 PMC9299539

[449] Luo, Ai‐Ling , Wen‐Ying Zheng , Qiong Zhang , Yan Yuan , Mei‐Qi Li , Kai Du , An‐Ran Gao , et al. 2025. “COPS5 Triggers Ferroptosis Defense by Stabilizing MK2 in Hepatocellular Carcinoma.” Advanced Science 12: e2416360. 10.1002/advs.202416360 40198582 PMC12165036

[450] Barbi de Moura, Michelle , Garret Vincent , Shelley L. Fayewicz , Nicholas W. Bateman , Brian L. Hood , Mai Sun , Joseph Suhan , et al. 2012. “Mitochondrial Respiration—An Important Therapeutic Target in Melanoma.” PLOS ONE 7: e40690. 10.1371/journal.pone.0040690 22912665 PMC3422349

[451] Dang, Derek , Akash Deogharkar , Akash Deogharkar , John McKolay , Kyle S. Smith , Pooja Panwalkar , Simon Hoffman , Wentao Tian , et al. 2025. “Isocitrate Dehydrogenase 1 Primes Group‐3 Medulloblastomas for Cuproptosis.” Cancer Cell 43: 1159–1174.e8. 10.1016/j.ccell.2025.04.013 40378837 PMC12151749

[452] Gallagher, Robert E . 1998. “Arsenic—New Life for an Old Potion.” New England Journal of Medicine 339: 1389–1391. 10.1056/NEJM199811053391909 9801402

[453] Chen, G. Q. , J. Zhu , X. G. Shi , J. H. Ni , H. J. Zhong , G. Y. Si , X. L. Jin , et al. 1996. “In Vitro Studies on Cellular and Molecular Mechanisms of Arsenic Trioxide (As2O3) in the Treatment of Acute Promyelocytic Leukemia: As2O3 Induces NB4 Cell Apoptosis With Downregulation of Bcl‐2 Expression and Modulation of PML‐RAR alpha/PML Proteins.” Blood 88: 1052–1061. 10.1182/blood.V88.3.1052.1052 https://www.ncbi.nlm.nih.gov/pubmed/8704214 8704214

[454] Soignet, Steven L. , Peter Maslak , Zhu‐Gang Wang , Suresh Jhanwar , Elizabeth Calleja , Laura J. Dardashti , Diane Corso , et al. 1998. “Complete Remission After Treatment of Acute Promyelocytic Leukemia With Arsenic Trioxide.” New England Journal of Medicine 339: 1341–1348. 10.1056/NEJM199811053391901 9801394

[455] Zhang, Chenliang , Tingting Huang , and Liping Li . 2024. “Targeting Cuproptosis for Cancer Therapy: Mechanistic Insights and Clinical Perspectives.” Journal of Hematology & Oncology 17: 68. 10.1186/s13045-024-01589-8 39152464 PMC11328505

[456] Chrzan, Natalia , and Mariusz L. Hartman . 2025. “Copper in Melanoma: At the Crossroad of Protumorigenic and Anticancer Roles.” Redox Biology 81: 103552. 10.1016/j.redox.2025.103552 39970778 PMC11880738

[457] Yim, Sun Young , Hayeon Kim , Tae Hyung Kim , Sang‐Hee Kang , Youngwoo Lee , Eunho Choi , Yang Jae Yoo , et al. 2026. “Identifying Sorafenib Benefit Among Patients With Hepatocellular Carcinoma: A Transcriptomic and Genomic Approach.” JHEP Reports 8: 101742. 10.1016/j.jhepr.2026.101742 41810428 PMC12969672

[458] He, Jiashuai , Yiran Zhang , Simin Luo , Zhan Zhao , Tianmu Mo , Hanyang Guan , Haoquan Li , et al. 2025. “Targeting SLC7A11 With Sorafenib Sensitizes Stereotactic Body Radiotherapy in Colorectal Cancer Liver Metastasis.” Drug Resistance Updates 81: 101250. 10.1016/j.drup.2025.101250 40381225

[459] Luo, Jing , Qiongjie Zhi , Dongxia Li , Yue Xu , Hui Zhu , Lujun Zhao , Guibing Ren , Jian Wang , and Ningbo Liu . 2025. “Low Dose Radiotherapy Combined With Immune Checkpoint Inhibitors Induces Ferroptosis in Lung Cancer via the Nrf2/HO‐1/GPX4 Axis.” Frontiers in Immunology 16: 1558814. 10.3389/fimmu.2025.1558814 40539046 PMC12178237

[460] Qiu, Liman , Hongbing Ji , Kai Wang , Wenhan Liu , Qizhen Huang , Xinting Pan , Honghao Ye , et al. 2024. “TLR3 Activation Enhances Abscopal Effect of Radiotherapy in HCC by Promoting Tumor Ferroptosis.” EMBO Molecular Medicine 16: 1193–1219. 10.1038/s44321-024-00068-4 38671318 PMC11098818

[461] Robe, Pierre A. , Didier H. Martin , Minh T. Nguyen‐Khac , Maria Artesi , Manuel Deprez , Adelin Albert , Sophie Vanbelle , et al. 2009. “Early Termination of ISRCTN45828668, a Phase 1/2 Prospective, Randomized Study of Sulfasalazine for the Treatment of Progressing Malignant Gliomas in Adults.” BMC Cancer 9: 372. 10.1186/1471-2407-9-372 19840379 PMC2771045

[462] Hsieh, Ming‐Shou , Hang Huong Ling , Syahru Agung Setiawan , Mardiah Suci Hardianti , Iat‐Hang Fong , Chi‐Tai Yeh , and Jia‐Hong Chen . 2024. “Therapeutic Targeting of Thioredoxin Reductase 1 Causes Ferroptosis While Potentiating Anti‐PD‐1 Efficacy in Head and Neck Cancer.” Chemico‐Biological Interactions 395: 111004. 10.1016/j.cbi.2024.111004 38636790

[463] Zhang, Chen , Xinyin Liu , Shidai Jin , Yi , Chen , and Renhua Guo . 2022. “Ferroptosis in Cancer Therapy: A Novel Approach to Reversing Drug Resistance.” Molecular Cancer 21: 47. 10.1186/s12943-022-01530-y 35151318 PMC8840702

[464] Wahida, Adam , and Marcus Conrad . 2025. “Decoding Ferroptosis for Cancer Therapy.” Nature Reviews Cancer 25: 910–924. 10.1038/s41568-025-00864-1 41073537

[465] Zheng, Peijie , Chuntao Zhou , Liuyi Lu , Bin Liu , and Yuemin Ding . 2022. “Elesclomol: A Copper Ionophore Targeting Mitochondrial Metabolism for Cancer Therapy.” Journal of Experimental & Clinical Cancer Research 41: 271. 10.1186/s13046-022-02485-0 36089608 PMC9465867

[466] O'Day, Steven J. , Alexander M. M. Eggermont , Vanna Chiarion‐Sileni , Richard Kefford , Jean Jacques Grob , Laurent Mortier , Caroline Robert , et al. 2013. “Final Results of Phase III SYMMETRY Study: Randomized, Double‐Blind Trial of Elesclomol Plus Paclitaxel Versus Paclitaxel Alone as Treatment for Chemotherapy‐Naive Patients With Advanced Melanoma.” Journal of Clinical Oncology 31: 1211–1218. 10.1200/JCO.2012.44.5585 23401447

[467] Nechushtan, Hovav , Yousef Hamamreh , Salim Nidal , Maya Gotfried , Amichai Baron , Yossi Israeli Shalev , et al. 2015. “A Phase IIb Trial Assessing the Addition of Disulfiram to Chemotherapy for the Treatment of Metastatic Non‐Small Cell Lung Cancer.” The Oncologist 20: 366–367. 10.1634/theoncologist.2014-0424 25777347 PMC4391770

[468] Werlenius, Katja , Sara Kinhult , Tora Skeidsvoll Solheim , Henriette Magelssen , David Löfgren , Munila Mudaisi , Sofia Hylin , et al. 2023. “Effect of Disulfiram and Copper Plus Chemotherapy vs Chemotherapy Alone on Survival in Patients With Recurrent Glioblastoma: A Randomized Clinical Trial.” JAMA Network Open 6: e234149. 10.1001/jamanetworkopen.2023.4149 37000452 PMC10066460

[469] Mahoney‐Sánchez, Laura , Hind Bouchaoui , Scott Ayton , David Devos , James A. Duce , and Jean‐Christophe Devedjian . 2021. “Ferroptosis and Its Potential Role in the Physiopathology of Parkinson's Disease.” Progress in Neurobiology 196: 101890. 10.1016/j.pneurobio.2020.101890 32726602

[470] Devos, David , Caroline Moreau , Jean Christophe Devedjian , Jérome Kluza , Maud Petrault , Charlotte Laloux , Aurélie Jonneaux , et al. 2014. “Targeting Chelatable Iron as a Therapeutic Modality in Parkinson's Disease.” Antioxidants & Redox Signaling 21: 195–210. 10.1089/ars.2013.5593 24251381 PMC4060813

[471] Devos, David , Julien Labreuche , Olivier Rascol , Jean‐Christophe Corvol , Alain Duhamel , Pauline Guyon Delannoy , Werner Poewe , et al. 2022. “Trial of Deferiprone in Parkinson's Disease.” New England Journal of Medicine 387: 2045–2055. 10.1056/NEJMoa2209254 36449420

[472] Millán, Mònica , Núria DeGregorio‐Rocasolano , Natàlia Pérez de la Ossa , Sílvia Reverté , Joan Costa , Pilar Giner , Yolanda Silva , et al. 2021. “Targeting Pro‐Oxidant Iron with Deferoxamine as a Treatment for Ischemic Stroke: Safety and Optimal Dose Selection in a Randomized Clinical Trial.” Antioxidants 10: 1270. 10.3390/antiox10081270 34439518 PMC8389327

[473] Aydinok, Yesim , Antonis Kattamis , M. Domenica Cappellini , Amal El‐Beshlawy , Raffaella Origa , Mohsen Elalfy , Yurdanur Kilinç , et al. 2015. “Effects of Deferasirox‐Deferoxamine on Myocardial and Liver Iron in Patients With Severe Transfusional Iron Overload.” Blood 125: 3868–3877. 10.1182/blood-2014-07-586677 25934475 PMC4490296

[474] Chen, Xu , Jiapan Shen , Xueqin Jiang , Min Pan , Shuang Chang , Juanjuan Li , Lei Wang , et al. 2024. “Characterization of Dipyridamole as a Novel Ferroptosis Inhibitor and Its Therapeutic Potential in Acute Respiratory Distress Syndrome Management.” Theranostics 14: 6947–6968. 10.7150/thno.102318 39629132 PMC11610143

[475] Chan, Nancy , Amy Willis , Naomi Kornhauser , Maureen M. Ward , Sharrell B. Lee , Eleni Nackos , Bo Ri Seo , et al. 2017. “Influencing the Tumor Microenvironment: A Phase II Study of Copper Depletion Using Tetrathiomolybdate in Patients With Breast Cancer at High Risk for Recurrence and in Preclinical Models of Lung Metastases.” Clinical Cancer Research 23: 666–676. 10.1158/1078-0432.CCR-16-1326 27769988

[476] Ramchandani, Divya , Mirela Berisa , Diamile A. Tavarez , Zhuoning Li , Matthew Miele , Yang Bai , Sharrell B. Lee , et al. 2021. “Copper Depletion Modulates Mitochondrial Oxidative Phosphorylation to Impair Triple Negative Breast Cancer Metastasis.” Nature Communications 12: 7311. 10.1038/s41467-021-27559-z PMC8674260 34911956

[477] Pilotto, Federica , Deepika M. Chellapandi , and Hélène Puccio . 2024. “Omaveloxolone: a Groundbreaking Milestone as the First FDA‐Approved Drug for Friedreich Ataxia.” Trends in Molecular Medicine 30: 117–125. 10.1016/j.molmed.2023.12.002 38272714

[478] Acute Liver Failure Study, Group , Hynan, Linda S. , William M. , Lorenzo Rossaro , Robert J. Fontana , R. Todd Stravitz , Anne M. Larson , Timothy J. Davern , Natalie G. Murray , et al. 2009. “Intravenous N‐Acetylcysteine Improves Transplant‐Free Survival in Early Stage Non‐Acetaminophen Acute Liver Failure.” Gastroenterology 137: 856–864.e1. 864 e851 10.1053/j.gastro.2009.06.006 19524577 PMC3189485

[479] Lee, Bor‐Jen , Yu‐Fen Tseng , Chi‐Hua Yen , and Ping‐Ting Lin . 2013. “Effects of Coenzyme Q10 Supplementation (300 mg/Day) on Antioxidation and Anti‐Inflammation in Coronary Artery Disease Patients During Statins Therapy: a Randomized, Placebo‐Controlled Trial.” Nutrition Journal 12: 142. 10.1186/1475-2891-12-142 24192015 PMC4176102

[480] O'Day, Steven , Rene Gonzalez , David Lawson , Robert Weber , Laura Hutchins , Clay Anderson , Jonathan Haddad , et al. 2009. “Phase II, Randomized, Controlled, Double‐Blinded Trial of Weekly Elesclomol Plus Paclitaxel Versus Paclitaxel Alone for Stage IV Metastatic Melanoma.” Journal of Clinical Oncology 27: 5452–5458. 10.1200/JCO.2008.17.1579 19826135

[481] Wiedau‐Pazos, Martina , Joy J. Goto , Shahrooz Rabizadeh , Edith B. Gralla , James A. Roe , Michael K. Lee , Joan S. Valentine , and Dale E. Bredesen . 1996. “Altered Reactivity of Superoxide Dismutase in Familial Amyotrophic Lateral Sclerosis.” Science 271: 515–518. 10.1126/science.271.5248.515 8560268

[482] Hunsaker, Elizabeth W. , and Katherine J. Franz . 2019. “Emerging Opportunities to Manipulate Metal Trafficking for Therapeutic Benefit.” Inorganic Chemistry 58: 13528–13545. 10.1021/acs.inorgchem.9b01029 31247859 PMC7272113

[483] Yang, Ying , Huanhuan Fan , and Zijian Guo . 2024. “Modulation of Metal Homeostasis for Cancer Therapy.” ChemPlusChem 89: e202300624. 10.1002/cplu.202300624 38315756

[484] Linkermann, Andreas , Rachid Skouta , Nina Himmerkus , Shrikant R. Mulay , Christin Dewitz , Federica De Zen , Agnes Prokai , et al. 2014. “Synchronized Renal Tubular Cell Death Involves Ferroptosis.” Proceedings of the National Academy of Sciences 111: 16836–16841. 10.1073/pnas.1415518111 PMC4250130 25385600

[485] Tuo, Qing‐zhang , Yu Liu , Zheng Xiang , Hong‐Fa Yan , Ting Zou , Yang Shu , Xu‐long Ding , et al. 2022. “Thrombin Induces ACSL4‐Dependent Ferroptosis During Cerebral Ischemia/Reperfusion.” Signal Transduction and Targeted Therapy 7: 59. 10.1038/s41392-022-00917-z 35197442 PMC8866433

[486] Sun, Shumin , Jie Shen , Jianwei Jiang , Fudi Wang , and Junxia Min . 2023. “Targeting Ferroptosis Opens New Avenues for the Development of Novel Therapeutics.” Signal Transduction and Targeted Therapy 8: 372. 10.1038/s41392-023-01606-1 37735472 PMC10514338

[487] Miao, Yu , Yiwei Chen , Feng Xue , Kexin Liu , Bin Zhu , Junjie Gao , Junhui Yin , Changqing Zhang , and Guangyi Li . 2022. “Contribution of Ferroptosis and GPX4′s Dual Functions to Osteoarthritis Progression.” eBioMedicine 76: 103847. 10.1016/j.ebiom.2022.103847 35101656 PMC8822178

[488] Hamza, Muhammad , Shuai Wang , Hao Wu , Jiayi Sun , Yang Du , Chuting Zeng , Yike Liu , et al. 2025. “Targeting Copper Homeostasis: Akkermansia‐Derived OMVs Co‐Deliver Atox1 siRNA and Elesclomol for Cancer Therapy.” Acta Pharmaceutica Sinica B 15: 2640–2654. 10.1016/j.apsb.2025.03.014 40487636 PMC12145039

[489] Kirk, Frederik Teicher , Ditte Emilie Munk , Eugene Scott Swenson , Adam Michael Quicquaro , Mikkel Holm Vendelbo , Agnete Larsen , Michael L. Schilsky , Peter Ott , and Thomas Damgaard Sandahl . 2024. “Effects of Tetrathiomolybdate on Copper Metabolism in Healthy Volunteers and in Patients With Wilson Disease.” Journal of Hepatology 80: 586–595. 10.1016/j.jhep.2023.11.023 38081365

[490] Sheline, ChristianT , EricH Choi , Jeong‐Sook Kim‐Han , LauraL Dugan , and DennisW Choi . 2002. “Cofactors of Mitochondrial Enzymes Attenuate Copper‐Induced Death In Vitro and In Vivo.” Annals of neurology 52: 195–204. 10.1002/ana.10276 12210790

[491] Hu, Jiayi , Xiaoyi Bao , Meihua Ting , Yecheng Tao , Ran Li , Guosheng Fu , Fuyu Qiu , Jing Zhao , and Wenbin Zhang . 2026. “Precision Targeting of FDX1‐Mediated Cuproptosis by a ROS‐Responsive Hydrogel for Myocardial Ischemia‐Reperfusion Injury Treatment.” Theranostics 16: 1281–1294. 10.7150/thno.120455 41355968 PMC12679365

[492] Koh, Jae‐Young , Sang W. Suh , Byoung J. Gwag , Yong Y. He , Chung Y. Hsu , and Dennis W. Choi . 1996. “The Role of Zinc in Selective Neuronal Death after Transient Global Cerebral Ischemia.” Science 272: 1013–1016. 10.1126/science.272.5264.1013 8638123

[493] Gong, Mengting , Yulin Fang , Kaijiang Yang , Fei Yuan , Rui Hu , Yajuan Su , Yiling Yang , et al. 2024. “The WFS1‐ZnT3‐Zn^2+^ Axis Regulates the Vicious Cycle of Obesity and Depression.” Advanced Science 11: e2403405. 10.1002/advs.202403405 39258564 PMC11538679

[494] Li, Yiqing , Lukas Andereggen , Kenya Yuki , Kumiko Omura , Yuqin Yin , Hui‐Ya Gilbert , Burcu Erdogan , et al. 2017. “Mobile Zinc Increases Rapidly in the Retina after Optic Nerve Injury and Regulates Ganglion Cell Survival and Optic Nerve Regeneration.” Proceedings of the National Academy of Sciences 114: E209–E218. 10.1073/pnas.1616811114 PMC5240690 28049831

[495] Nguyen, Huynh Quang Dieu , Mi‐hyun Nam , Jozsef Vigh , Joseph Brzezinski , Lucas Duncan , and Daewon Park . 2025. “Co‐Delivery of Neurotrophic Factors and a Zinc Chelator Substantially Increases Retinal Ganglion Cell Survival and Axon Protection in the Optic Nerve Crush Model.” Acta Biomaterialia 201: 297–308. 10.1016/j.actbio.2025.06.007 40482982 PMC12381791

[496] Ma, Qing , Yini Xiao , Wenjun Xu , Menghan Wang , Sheng Li , Zhihao Yang , Minglu Xu , et al. 2022. “ZnT8 Loss‐of‐Function Accelerates Functional Maturation of hESC‐derived β Cells and Resists Metabolic Stress in Diabetes.” Nature Communications 13: 4142. 10.1038/s41467-022-31829-9 PMC9288460 35842441

[497] Twyning, Madeleine J. , Roberta Tufi , Thomas P. Gleeson , Kinga M. Kolodziej , Susanna Campesan , Ana Terriente‐Felix , Lewis Collins , et al. 2024. “Partial Loss of MCU Mitigates Pathology In Vivo Across a Diverse Range of Neurodegenerative Disease Models.” Cell Reports 43: 113681. 10.1016/j.celrep.2024.113681 38236772

[498] Nashed, Mina G. , Robert G. Ungard , Kimberly Young , Natalie J. Zacal , Eric P. Seidlitz , Jennifer Fazzari , Benicio N. Frey , and Gurmit Singh . 2017. “Behavioural Effects of Using Sulfasalazine to Inhibit Glutamate Released by Cancer Cells: A Novel Target for Cancer‐Induced Depression.” Scientific Reports 7: 41382. 10.1038/srep41382 28120908 PMC5264609

[499] Brewer, George J. , Fred Askari , Matthew T. Lorincz , Martha Carlson , Michael Schilsky , Karen J. Kluin , Peter Hedera , et al. 2006. “Treatment of Wilson Disease With Ammonium Tetrathiomolybdate: IV. Comparison of Tetrathiomolybdate and Trientine in a Double‐Blind Study of Treatment of the Neurologic Presentation of Wilson Disease.” Archives of Neurology 63: 521–527. 10.1001/archneur.63.4.521 16606763

[500] Vidart, Josi , Simone Magagnin Wajner , Rogério Sarmento Leite , André Manica , Beatriz D. Schaan , P. Reed Larsen , and Ana Luiza Maia . 2014. “N‐Acetylcysteine Administration Prevents Nonthyroidal Illness Syndrome in Patients With Acute Myocardial Infarction: A Randomized Clinical Trial.” The Journal of Clinical Endocrinology & Metabolism 99: 4537–4545. 10.1210/jc.2014-2192 25148231 PMC4255112

[501] Li, Yang , Dongcheng Feng , Zhanyu Wang , Yan Zhao , Ruimin Sun , Donghai Tian , Deshun Liu , et al. 2019. “Ischemia‐Induced ACSL4 Activation Contributes to Ferroptosis‐Mediated Tissue Injury in Intestinal Ischemia/Reperfusion.” Cell Death & Differentiation 26: 2284–2299. 10.1038/s41418-019-0299-4 30737476 PMC6889315

[502] Lee, Jaewang , and Jong‐Lyel Roh . 2022. “SLC7A11 as a Gateway of Metabolic Perturbation and Ferroptosis Vulnerability in Cancer.” Antioxidants 11: 2444. 10.3390/antiox11122444 36552652 PMC9774303

[503] Chen, Zhen , Weilong Wang , Siti Razila , Abdul Razak , Tao Han , Nor Hazwani , et al. 2023. “Ferroptosis as a Potential Target for Cancer Therapy.” Cell Death & Disease 14: 460. 10.1038/s41419-023-05930-w 37488128 PMC10366218

[504] Dar, Nawab John , Urmilla John , Nargis Bano , Sameera Khan , and Shahnawaz Ali Bhat . 2024. “Oxytosis/Ferroptosis in Neurodegeneration: The Underlying Role of Master Regulator Glutathione Peroxidase 4 (GPX4).” Molecular Neurobiology 61: 1507–1526. 10.1007/s12035-023-03646-8 37725216

[505] Daugherty, Ana M. , and Naftali Raz . 2015. “Appraising the Role of Iron in Brain Aging and Cognition: Promises and Limitations of MRI Methods.” Neuropsychology Review 25: 272–287. 10.1007/s11065-015-9292-y 26248580 PMC4565753

[506] Silver, Brian B. , Carri Murphy , Erik J. Tokar , and Birandra K. Sinha . 2026. “Ferroptosis Suppressor Protein 1 (FSP1)‐CoQ10‐NADPH‐Axis Is Responsible for Erastin Resistance in MCF‐7 Breast Cancer Cells.” Antioxidants 15: 239. 10.3390/antiox15020239 41750619 PMC12938241

[507] Liu, Yanqing , and Wei Gu . 2022. “p53 in Ferroptosis Regulation: the New Weapon for the Old Guardian.” Cell Death & Differentiation 29: 895–910. 10.1038/s41418-022-00943-y 35087226 PMC9091200

[508] Tsvetkov, Peter , Alexandre Detappe , Kai Cai , Heather R. Keys , Zarina Brune , Weiwen Ying , Prathapan Thiru , et al. 2019. “Mitochondrial Metabolism Promotes Adaptation to Proteotoxic Stress.” Nature Chemical Biology 15: 681–689. 10.1038/s41589-019-0291-9 31133756 PMC8183600

[509] Zheng, Nan , Qiong Zhou , Zihao Chen , Lihua Xie , Xinyu Yang , Ziwen Chen , Fuwei Wang , et al. 2025. “Cuproptosis: Mechanisms and Links With Alzheimer's Disease.” Journal of Neurophysiology 134: 1853–1876. 10.1152/jn.00370.2025 41143863

[510] Mangala, Lingegowda S. , Vesna Zuzel , Rosemarie Schmandt , Erik S. Leshane , Jyotsna B. Halder , Guillermo N. Armaiz‐Pena , Whitney A. Spannuth , et al. 2009. “Therapeutic Targeting of ATP7B in Ovarian Carcinoma.” Clinical Cancer Research 15: 3770–3780. 10.1158/1078-0432.CCR-08-2306 19470734 PMC2752981

[511] Bandmann, Oliver , Karl Heinz Weiss , and Stephen G. Kaler . 2015. “Wilson's Disease and Other Neurological Copper Disorders.” The Lancet Neurology 14: 103–113. 10.1016/S1474-4422(14)70190-5 25496901 PMC4336199

[512] Wang, Yue , Menghan Zhang , Ran Bi , Yali Su , Fei Quan , Yanting Lin , Chongxiu Yue , et al. 2022. “ACSL4 Deficiency Confers Protection Against Ferroptosis‐Mediated Acute Kidney Injury.” Redox Biology 51: 102262. 10.1016/j.redox.2022.102262 35180475 PMC8857079

[513] Ye, Keng , Ruilong Lan , Zhimin Chen , Kunmei Lai , Yankun Song , Guoping Li , Huabin Ma , Hong Chen , and Yanfang Xu . 2025. “Roles of ACSL4/GPX4 and FSP1 in Oxalate‐Induced Acute Kidney Injury.” Cell Death Discovery 11: 279. 10.1038/s41420-025-02557-y 40527896 PMC12174353

[514] Zeng, Yingjie , Yuening Cao , Senmiao Ren , Chaozheng Zhang , Jianan Liu , Ke Liu , Yan , Wang , et al. 2025. “Responsive ROS‐Augmented Prodrug Hybridization Nanoassemblies for Multidimensionally Synergitic Treatment of Hepatocellular Carcinoma in Cascade Assaults.” Advanced Science 12: e2501420. 10.1002/advs.202501420 40323152 PMC12362818

[515] Członkowska, Anna , Tomasz Litwin , Petr Dusek , Peter Ferenci , Svetlana Lutsenko , Valentina Medici , Janusz K. Rybakowski , Karl Heinz Weiss , and Michael L. Schilsky . 2018. “Wilson Disease.” Nature Reviews Disease Primers 4: 21. 10.1038/s41572-018-0018-3 PMC6416051 30190489

[516] Yan, Ruihan , Bingyi Lin , Wenwei Jin , Ling Tang , Shuming Hu , and Rong Cai . 2023. “NRF2, a Superstar of Ferroptosis.” Antioxidants 12: 1739. 10.3390/antiox12091739 37760042 PMC10525540

[517] Ye, Yujun , Xuexin Xie , Yiming Bi , Qing Liu , Lingling Qiu , He Zhao , Chengyin Wang , Weifeng Zhu , and Ting Zeng . 2025. “Nrf2 Alleviates Acute Ischemic Stroke Induced Ferroptosis via Regulating xCT/GPX4 Pathway.” Free Radical Biology and Medicine 231: 153–162. 10.1016/j.freeradbiomed.2025.02.040 40020881

[518] Yang, Xi‐chen , Ya‐ju Jin , Rong Ning , Qiu‐yue Mao , Peng‐yue Zhang , Li Zhou , Cheng‐cai Zhang , Yi‐chen Peng , and Na Chen . 2025. “Electroacupuncture Attenuates Ferroptosis by Promoting Nrf2 Nuclear Translocation and Activating Nrf2/SLC7A11/GPX4 Pathway in Ischemic Stroke.” Chinese Medicine 20: 4. 10.1186/s13020-024-01047-0 39755657 PMC11699709

[519] Wei, Ya‐Li . 2025. “Ferroptosis in Ischemia‐ReperfuZhixuan Bian, Yixuan Xiao, Juan Ruansion Injury: Molecular Mechanisms and Therapeutic Strategies.” American Journal of Cardiovascular Disease 15: 405–441. 10.62347/OLGV6926 41567851 PMC12816776

[520] Feng, Lei , Kaikai Zhao , Liangchao Sun , Xiaoyang Yin , Junpeng Zhang , Conghe Liu , and Baosheng Li . 2021. “SLC7A11 Regulated by NRF2 Modulates Esophageal Squamous Cell Carcinoma Radiosensitivity by Inhibiting Ferroptosis.” Journal of Translational Medicine 19: 367. 10.1186/s12967-021-03042-7 34446045 PMC8393811

[521] Cole, Toby B. , H. Jürgen Wenzel , Kathy E. Kafer , Philip A. Schwartzkroin , and Richard D. Palmiter . 1999. “Elimination of Zinc From Synaptic Vesicles in the Intact Mouse Brain by Disruption of the ZnT3 Gene.” Proceedings of the National Academy of Sciences 96: 1716–1721. 10.1073/pnas.96.4.1716 PMC15571 9990090

[522] Paillard, Melanie , György Csordás , Gergö Szanda , Tünde Golenár , Valentina Debattisti , Adam Bartok , Nadan Wang , et al. 2017. “Tissue‐Specific Mitochondrial Decoding of Cytoplasmic Ca^2+^ Signals Is Controlled by the Stoichiometry of MICU1/2 and MCU.” Cell Reports 18: 2291–2300. 10.1016/j.celrep.2017.02.032 28273446 PMC5760244

[523] Debattisti, Valentina , Adam Horn , Raghavendra Singh , Erin L. Seifert , Marshall W. Hogarth , Davi A. Mazala , Kai Ting Huang , et al. 2019. “Dysregulation of Mitochondrial Ca2+ Uptake and Sarcolemma Repair Underlie Muscle Weakness and Wasting in Patients and Mice Lacking MICU1.” Cell Reports 29: 1274–1286.e6. 10.1016/j.celrep.2019.09.063 31665639 PMC7007691

[524] Zhang, Chen , Wenbo Bu , Dalong Ni , Shenjian Zhang , Qing Li , Zhenwei Yao , Jiawen Zhang , et al. 2016. “Synthesis of Iron Nanometallic Glasses and Their Application in Cancer Therapy by a Localized Fenton Reaction.” Angewandte Chemie International Edition 55: 2101–2106. 10.1002/anie.201510031 26836344

[525] Zhang, Shuaibing , Xuejiao J. Gao , Yuanjie Ma , Kexu Song , Mengyue Ge , Saiyu Ma , Lirong Zhang , et al. 2024. “A Bioinspired Sulfur–Fe–Heme Nanozyme With Selective Peroxidase‐Like Activity for Enhanced Tumor Chemotherapy.” Nature Communications 15: 10605. 10.1038/s41467-024-54868-w PMC11621791 39638998

[526] Estrella, Veronica , Tingan Chen , Mark Lloyd , Jonathan Wojtkowiak , Heather H. Cornnell , Arig Ibrahim‐Hashim , Kate Bailey , et al. 2013. “Acidity Generated by the Tumor Microenvironment Drives Local Invasion.” Cancer Research 73: 1524–1535. 10.1158/0008-5472.CAN-12-2796 23288510 PMC3594450

[527] Gao, Lizeng , Jie Zhuang , Leng Nie , Jinbin Zhang , Yu Zhang , Ning Gu , Taihong Wang , et al. 2007. “Intrinsic Peroxidase‐Like Activity of Ferromagnetic Nanoparticles.” Nature Nanotechnology 2: 577–583. 10.1038/nnano.2007.260 18654371

[528] Hu, Zhiyuan , Jie Shan , Xu Jin , Weijie Sun , Liang Cheng , Xu‐Linu Chen , Xianwen Wang . 2024. “Nanoarchitectonics of in Situ Antibiotic‐Releasing Acicular Nanozymes for Targeting and Inducing Cuproptosis‐Like Death to Eliminate Drug‐Resistant Bacteria.” ACS Nano 18: 24327–24349. 10.1021/acsnano.4c06565 39169538

[529] Jiang, Bing , Demin Duan , Lizeng Gao , Mengjie Zhou , Kelong Fan , Yan Tang , Juqun Xi , et al. 2018. “Standardized Assays for Determining the Catalytic Activity and Kinetics of Peroxidase‐Like Nanozymes.” Nature Protocols 13: 1506–1520. 10.1038/s41596-018-0001-1 29967547

[530] Wu, Luyan , Huihui Lin , Xiang Cao , Qiang Tong , Fangqi Yang , Yinxing Miao , Deju Ye , and Quli Fan . 2024. “Bioorthogonal Cu Single‐Atom Nanozyme for Synergistic Nanocatalytic Therapy, Photothermal Therapy, Cuproptosis and Immunotherapy.” Angewandte Chemie 136: e202405937. 10.1002/ange.202405937 38654446

[531] Xu, Wei , Guoqiang Guan , Renye Yue , Zhe Dong , Lingling Lei , Heemin Kang , and Guosheng Song . 2025. “Chemical Design of Magnetic Nanomaterials for Imaging and Ferroptosis‐Based Cancer Therapy.” Chemical Reviews 125: 1897–1961. 10.1021/acs.chemrev.4c00546 39951340

[532] Chen, Yuanjun , Shufang Ji , Chen Chen , Qing Peng , Dingsheng Wang , and Yadong Li . 2018. “Single‐Atom Catalysts: Synthetic Strategies and Electrochemical Applications.” Joule 2: 1242–1264. 10.1016/j.joule.2018.06.019

[533] Singh, Baljeet , Manoj B. Gawande , Arun D. Kute , Rajender S. Varma , Paolo Fornasiero , Peter McNeice , Rajenahally V. Jagadeesh , Matthias Beller , and Radek Zbořil . 2021. “Single‐Atom (Iron‐Based) Catalysts: Synthesis and Applications.” Chemical Reviews 121: 13620–13697. 10.1021/acs.chemrev.1c00158 34644065

[534] Yang, Meng‐Die , Chun‐Yan Zhu , Gang Yang , Xiao‐Yi Zhang , Yi Zhu , Miao Chen , Jia‐Jia Zhang , et al. 2025. “Camouflaged Membrane‐Bridged Radionuclide/Mn Single‐Atom Enzymes Target Lipid Metabolism Disruption to Evoke Antitumor Immunity.” Military Medical Research 12: 59. 10.1186/s40779-025-00647-7 40968370 PMC12447611

[535] Du, Yaqian , Xudong Zhao , Fei He , Haijiang Gong , Jiani Yang , Linzhi Wu , Xianchang Cui , et al. 2024. “A Vacancy‐Engineering Ferroelectric Nanomedicine for Cuproptosis/Apoptosis Co‐Activated Immunotherapy.” Advanced Materials 36: e2403253. 10.1002/adma.202403253 38703184

[536] Zhu, Guoqing , Yanrong Qian , Minghong Gao , Qianqian Sun , Ping'an Ma , Jun Lin , and Chunxia Li . 2026. “A Novel Nano‐Biocomposite Synergistically Activates PANoptosis and cGAS‐STING for Precise Cancer Immunotherapy.” Biomaterials 327: 123758. 10.1016/j.biomaterials.2025.123758 41061612

[537] Liu, Yang , Rui Niu , Huan Zhao , Yinghui Wang , Shuyan Song , Hongjie Zhang , and Yanli Zhao . 2024. “Single‐Site Nanozymes with a Highly Conjugated Coordination Structure for Antitumor Immunotherapy via Cuproptosis and Cascade‐Enhanced T Lymphocyte Activity.” Journal of the American Chemical Society 146: 3675–3688. 10.1021/jacs.3c08622 38305736

[538] Liu, Zhen , Junhong Ling , Nan Wang , and Xiao–kun Ouyang . 2025. “Redox Homeostasis Disruptors Enhanced Cuproptosis Effect for Synergistic Photothermal/Chemodynamic Therapy.” Journal of Colloid and Interface Science 678: 1060–1074. 10.1016/j.jcis.2024.08.234 39236435

[539] Tang, Wei , Jie Wu , Li Wang , Kailu Wei , Zifan Pei , Fei Gong , Linfu Chen , et al. 2024. “Bioactive Layered Double Hydroxides for Synergistic Sonodynamic/Cuproptosis Anticancer Therapy With Elicitation of the Immune Response.” ACS Nano 18: 10495–10508. 10.1021/acsnano.3c11818 38556991

[540] Yang, Zhuoran , Zhuo Li , Chunyu Yang , Li Meng , Wei Guo , and Liqiang Jing . 2025. “Synthesis of Closely‐Contacted Cu_2_O‐CoWO_4_ Nanosheet Composites for Cuproptosis Therapy to Tumors With Sonodynamic and Photothermal Assistance.” Advanced Science 12: e2410621. 10.1002/advs.202410621. 39573907 PMC11727377

[541] Liu, Tao , Wenlong Liu , Mingkang Zhang , Wuyang Yu , Fan Gao , Chuxin Li , Shi‐Boi Wang , et al. 2018. “Ferrous‐Supply‐Regeneration Nanoengineering for Cancer‐Cell‐Specific Ferroptosis in Combination With Imaging‐Guided Photodynamic Therapy.” ACS Nano 12: 12181–12192. 10.1021/acsnano.8b05860 30458111

[542] Liu, Yongbin , Dongfang Yu , Xueying Ge , Lingyi Huang , Ping‐Ying Pan , Haifa Shen , Roderic I. Pettigrew , Shu‐Hsia Chen , Junhua Mai . 2025. “Novel Platinum Therapeutics Induce Rapid Cancer Cell Death Through Triggering Intracellular ROS Storm.” Biomaterials 314: 122835. 10.1016/j.biomaterials.2024.122835 39276409 PMC11560510

[543] Guo, Junling , Yuan Ping , Hirotaka Ejima , Karen Alt , Mirko Meissner , Joseph J. Richardson , Yan Yan , et al. 2014. “Engineering Multifunctional Capsules Through the Assembly of Metal‐Phenolic Networks.” Angewandte Chemie International Edition 53: 5546–5551. 10.1002/anie.201311136 24700671

[544] Mi, Peng , Daisuke Kokuryo , Horacio Cabral , Hailiang Wu , Yasuko Terada , Tsuneo Saga , Ichio Aoki , Nobuhiro Nishiyama , and Kazunori Kataoka . 2016. “A pH‐Activatable Nanoparticle With Signal‐Amplification Capabilities for Non‐Invasive Imaging of Tumour Malignancy.” Nature Nanotechnology 11: 724–730. 10.1038/nnano.2016.72 27183055

[545] Zhou, Xinyuan , Anwei Zhou , Zihan Tian , Weiwei Chen , Yurui Xu , Xinghai Ning , and Kerong Chen . 2023. “A Responsive Nanorobot Modulates Intracellular Zinc Homeostasis to Amplify Mitochondria‐Targeted Phototherapy.” Small 19: e2302952. 10.1002/smll.202302952 37434337

[546] Lv, Chunxu , Wenyan Kang , Shuo Liu , Pishan Yang , Yuta Nishina , Shaohua Ge , Alberto Bianco , and Baojin Ma . 2022. “Growth of ZIF‐8 Nanoparticles In Situ on Graphene Oxide Nanosheets: A Multifunctional Nanoplatform for Combined Ion‐Interference and Photothermal Therapy.” ACS Nano 16: 11428–11443. 10.1021/acsnano.2c05532 35816172

[547] Zhou, Zaigang , Yu Liu , Wei Song , Xin Jiang , Zaian Deng , Wei Xiong , and Jianliang Shen . 2022. “Metabolic Reprogramming Mediated PD‐L1 Depression and Hypoxia Reversion to Reactivate Tumor Therapy.” Journal of Controlled Release 352: 793–812. 10.1016/j.jconrel.2022.11.004 36343761

[548] Fu, Liwen , Weiying Zhang , Xiaojun Zhou , Jingzhong Fu , and Chuanglong He . 2022. “Tumor Cell Membrane‐Camouflaged Responsive Nanoparticles Enable MRI‐Guided Immuno‐Chemodynamic Therapy of Orthotopic Osteosarcoma.” Bioactive Materials 17: 221–233. 10.1016/j.bioactmat.2022.01.035 35386464 PMC8965157

[549] Jin, Minghao , Muge Gu , Keyu Kong , Wenxuan Fan , Sonu Ng , Yuehao Hu , Zhe Wang , et al. 2026. “Chemical Gambit in Bone Microenvironment: pH‐Responsive Cu‐Mn Nanoplatform Breaks the Inflammation‐Osteoclast Vicious Cycle in Prosthesis‐Associated Osteolysis.” ACS Nano 20: 1826–1843. 10.1021/acsnano.5c09438 41480959

[550] Yang, Weihao , Chunjie Wang , Chao Yu , Nan Jiang , Bin Yu , Yicheng Ni , Caifang Ni , Liangzhu Feng , and Lei Zhang . 2026. “pH‐Regulating Lipiodol Pickering Emulsions Enhance Transarterial Embolization Therapy via Inducing Ferroptosis and Activating Antitumor Immunity.” Cell Reports Medicine 7: 102662. 10.1016/j.xcrm.2026.102662 41850231 PMC13006419

[551] Wang, Chunjie , Lei Zhang , Zhijuan Yang , Dongxu Zhao , Zheng Deng , Jialu Xu , Yumin Wu , et al. 2024. “Self‐Fueling Ferroptosis‐Inducing Microreactors Based on pH‐Responsive Lipiodol Pickering Emulsions Enable Transarterial Ferro‐Embolization Therapy.” National Science Review 11: nwad257. 10.1093/nsr/nwad257 38116090 PMC10727844

[552] Wang, Panfeng , Lijun Ren , Yifan Tang , Bo Yuan , Bijiang Geng , and Yin Zhao . 2024. “Tumor Microenvironment Activated MXene‐Protected Cu_2_O Heterojunctions Induce Tumor‐Specific Cuproptosis for Enhanced Sono‐Immunotherapy.” Chemical Engineering Journal 500: 156753. 10.1016/j.cej.2024.156753

[553] Li, Kun , Leilei Wu , Han Wang , Zi Fu , Jiani Gao , Xiucheng Liu , Yongfei Fan , et al. 2024. “Apoptosis and Cuproptosis Co‐Activated Copper‐Based Metal‐Organic Frameworks for Cancer Therapy.” Journal of Nanobiotechnology 22: 546. 10.1186/s12951-024-02828-3 39237931 PMC11378619

[554] Mei, Guanghui , Fei Lin , Liying Huang , Min Lin , Xinhua Lin , and Lingyi Huang . 2025. “A pH/H2O2 Dual‐Responsive Cobalt–Manganese‐Based Nano‐Delivery System for Chemo/Chemodynamic Therapy in Triple‐Negative Breast Cancer.” International Journal of Nanomedicine 20: 13359–13379. 10.2147/IJN.S546671 41216351 PMC12596851

[555] Bian, Yulong , Bin Liu , Binbin Ding , Meifang Wang , Meng Yuan , Ping'an Ma , and Jun Lin . 2023. “Tumor Microenvironment‐Activated Nanocomposite for Self‐Amplifying Chemodynamic/Starvation Therapy Enhanced IDO‐Blockade Tumor Immunotherapy.” Advanced Science 10: e2303580. 10.1002/advs.202303580 37807763 PMC10700178

[556] Wu, Hangyi , Xiaoyu Lu , Yuhan Hu , J. Baatarbolat , Zhihao Zhang , Yiping Liang , et al. 2025. “Biomimic Nanodrugs Overcome Tumor Immunosuppressive Microenvironment to Enhance Cuproptosis/Chemodynamic‐Induced Cancer Immunotherapy.” Advanced Science 12: e2411122. 10.1002/advs.202411122 39665263 PMC11791997

[557] Sun, Quanwei , Jinming Yang , Wei Shen , Huiyu Lu , Xiaohui Hou , Yang Liu , Yujing Xu , et al. 2022. “Engineering Mitochondrial Uncoupler Synergistic Photodynamic Nanoplatform to Harness Immunostimulatory Pro‐Death Autophagy/Mitophagy.” Biomaterials 289: 121796. 10.1016/j.biomaterials.2022.121796 36108581

[558] Ren, Hong , Zhihong Deng , Sheng Lu , Jing Zhang , Wenbin Liu , and Jia Tan . 2026. “Dual‐Targeting Cuproptosis and Mitophagy via a Flavopiridol‐Copper Nanoplatform Potentiates Immunotherapy Against Uveal Melanoma.” Advanced Science 13: e21183. 10.1002/advs.202521183 41891778 PMC13252610

[559] Deng, Kun , Wei Gao , Yu Wen , Jianliang Huang , Xuetong Li , Xiaoxin Yang , and Minghua Wu . 2026. “A Functional Nanocomposite Tri‐Activates Cuproptosis, Ferroptosis, and Mitophagy Death Pathway to Oppose Malignancies.” Advanced Healthcare Materials 15: e03530. 10.1002/adhm.202503530 41195893

[560] Liu, Molin , Jian Zheng , Mengqi Yu , Qirui Wang , Yi Yuan , Nannan Shao , Xiaoliang Yang , et al. 2026. “Stimuli‐Responsive CuFeTe_2_ Nanosheets for Amplified Cuproptosis/Ferroptosis in Triple‐Negative Breast Cancer Therapy.” Advanced Science 13: e05739. 10.1002/advs.202505739 41133938 PMC12806277

[561] Yue, Renye , Mengjie Zhou , Xu Li , Li Xu , Chang Lu , Zhe Dong , Lingling Lei , et al. 2023. “GSH/APE1 Cascade‐Activated Nanoplatform for Imaging Therapy Resistance Dynamics and Enzyme‐Mediated Adaptive Ferroptosis.” ACS Nano 17: 13792–13810. 10.1021/acsnano.3c03443 37458417

[562] Hu, Chuan , Xingli Cun , Shaobo Ruan , Rui Liu , Wei Xiao , Xiaotong Yang , Yuanyuan Yang , Chuanyao Yang , and Huile Gao . 2018. “Enzyme‐Triggered Size Shrink and Laser‐Enhanced NO Release Nanoparticles for Deep Tumor Penetration and Combination Therapy.” Biomaterials 168: 64–75. 10.1016/j.biomaterials.2018.03.046 29626787

[563] Iaccarino, Giulia , Martina Profeta , Raffaele Vecchione , Paolo A. Netti . 2019. “Matrix Metalloproteinase‐Cleavable Nanocapsules for Tumor‐Activated Drug Release.” Acta Biomaterialia 89: 265–278. 10.1016/j.actbio.2019.02.043 30851453

[564] Qin, Wen , Jinzhao Huang , Chunsheng Yang , Quer Yue , Shizhen Chen , Mengdie Wang , Shangbang Gao , et al. 2023. “Protease‐Activatable Nanozyme With Photoacoustic and Tumor‐Enhanced Magnetic Resonance Imaging for Photothermal Ferroptosis Cancer Therapy.” Advanced Functional Materials 33: 2209748. 10.1002/adfm.202209748

[565] Wang, Yifan , Yanqiu Zhang , Zhengxing Ru , Wei Song , Lin Chen , Hao Ma , and Lizhu Sun . 2019. “A ROS‐responsive Polymeric Prodrug Nanosystem With Self‐Amplified Drug Release for PSMA (−) Prostate Cancer Specific Therapy.” Journal of Nanobiotechnology 17: 91. 10.1186/s12951-019-0521-z 31451114 PMC6709549

[566] Li, Xiaoye , Qiang Li , Ao He , Meng Dang , Yu Zhang , Minjin Wang , Qinhong Sun , et al. 2025. “Disrupting Biofilm Tolerance by Ionic Microbubble‐Mediated Copper Ion Surge for Infection Clearance.” ACS Nano 19: 28624–28643. 10.1021/acsnano.5c08035 40743484 PMC12356198

[567] Xiang, Qi , Yan Zhang , Yuhong Ren , Fuxue Luo , Yunfang Wu , Haitao Ran , and Yang Cao . 2026. “Unleashing the Potent Antitumor Force: A Reactive Oxygen Species (ROS) Storm Formation by Ultrasound‐Activatable Metal Nanosonosensitizers.” Small 22: e13649. 10.1002/smll.202513649 41504027

[568] Huang, Yanli , Xufeng Wan , Qiang Su , Chunlin Zhao , Jian Cao , Yan Yue , Shuoyuan Li , et al. 2024. “Ultrasound‐Activated Piezo‐Hot Carriers Trigger Tandem Catalysis Coordinating Cuproptosis‐Like Bacterial Death Against Implant Infections.” Nature Communications 15: 1643. 10.1038/s41467-024-45619-y PMC10884398 38388555

[569] Cheng, Shuangshuang , Ting Zhou , Yue Luo , Jun Zhang , Kejun Dong , Qi Zhang , Wan Shu , et al. 2024. “Ultrasound‐Responsive Bi_2_MoO_6_‐MXene Heterojunction as Ferroptosis Inducers for Stimulating Immunogenic Cell Death Against Ovarian Cancer.” Journal of Nanobiotechnology 22: 408. 10.1186/s12951-024-02658-3 38992664 PMC11238442

[570] Liu, Zihui , Fei Li , Jialong Sun , Zhen Li , Keyang Li , Maochao Zheng , Jun Zhu , Shasha He , and Huayu Tian . 2026. “Reversible Metal Coordination Switch Enables Activatable Sonodynamic Therapy via Dual‐Death and Immune Synergy.” Advanced Materials 38: e21565. 10.1002/adma.202521565 41580916

[571] Cheng, Runzi , Zhenhao Li , Weican Luo , Hongwu Chen , Tingting Deng , Zhenqi Gong , Qing Zheng , et al. 2025. “A Copper‐Based Photothermal‐Responsive Nanoplatform Reprograms Tumor Immunogenicity via Self‐Amplified Cuproptosis for Synergistic Cancer Therapy.” Advanced Science 12: e2500652. 10.1002/advs.202500652 40125789 PMC12097029

[572] Yuan, Ying , Bo Chen , Xingxing An , Zhanhang Guo , Xin Liu , Hao Lu , Fangxin Hu , et al. 2024. “MOFs‐Based Magnetic Nanozyme to Boost Cascade ROS Accumulation for Augmented Tumor Ferroptosis.” Advanced Healthcare Materials 13: e2304591. 10.1002/adhm.202304591 38528711

[573] Jiang, Yuyan , Xuhui Zhao , Jiaguo Huang , Jingchao Li , Paul Kumar Upputuri , He Sun , Xiao Han , et al. 2020. “Transformable Hybrid Semiconducting Polymer Nanozyme for Second Near‐Infrared Photothermal Ferrotherapy.” Nature Communications 11: 1857. 10.1038/s41467-020-15730-x PMC7170847 32312987

[574] Dai, Yeneng , Lipeng Zhu , Xue Li , Fengjuan Zhang , Kai Chen , Guanda Jiao , Yu Liu , et al. 2024. “A Biomimetic Cuproptosis Amplifier for Targeted NIR‐II Fluorescence/Photoacoustic Imaging‐Guided Synergistic NIR‐II Photothermal Immunotherapy.” Biomaterials 305: 122455. 10.1016/j.biomaterials.2023.122455 38160626

[575] Zhao, Huan , Shujuan Jin , Yang Liu , Qian Wang , Brynne Shu Ni Tan , T Wang , Wang‐Kang Han , et al. 2025. “A Second Near‐Infrared Window‐Responsive Metal‐Organic‐Framework‐Based Photosensitizer for Tumor Immunotherapy via Synergistic Ferroptosis and STING Activation.” Journal of the American Chemical Society 147: 4871–4885. 10.1021/jacs.4c13241 39854684

[576] Yuan, Xue , Yong Kang , Ruiyan Li , Gaoli Niu , Jiacheng Shi , Yiwen Yang , Yueyue Fan , et al. 2025. “Magnetically Triggered Thermoelectric Heterojunctions With an Efficient Magnetic‐Thermo‐Electric Energy Cascade Conversion for Synergistic Cancer Therapy.” Nature Communications 16: 2369. 10.1038/s41467-025-57672-2 PMC11894112 40064895

[577] Qiao, Lihong , Yijing Ou , Lin Li , Shuzhen Wu , Yanxian Guo , Mu Liu , Dongsheng Yu , et al. 2024. “H(2)S‐Driven Chemotherapy and Mild Photothermal Therapy Induced Mitochondrial Reprogramming to Promote Cuproptosis.” Journal of Nanobiotechnology 22: 205. 10.1186/s12951-024-02480-x 38658965 PMC11044430

[578] Ling, Junhong , Zhen Liu , Hang Wu , Yuanjie Jin , Yani He , Nan Wang , and Xiao‐kun Ouyang . 2026. “Cascade‐Responsive Hydrogen Sulfide‐Releasing Nanoplatform for Synergistic Tumor Photothermal‐Immunotherapy.” Journal of Colloid and Interface Science 709: 139971. 10.1016/j.jcis.2026.139971 41605106

[579] Li, Binyi , Zheng Li , Ying Qian , Nan Xiao , Chunyun Fan , Yong Huang , Anwei Zhou , and Xinghai Ning . 2024. “The Convergence of Sonodynamic Therapy and Cuproptosis in the Dual‐Responsive Biomimetic CytoNano for Precision Mitochondrial Intervention in Cancer Treatment.” Nano Letters 24: 8107–8116. 10.1021/acs.nanolett.4c01864 38888223

[580] Xu, Luyao , Jing Feng , Shihao Xu , Mingli Jin , Xianyue Bai , Hui Zhang , Minghui Zhu , et al. 2026. “GSH‐responsive Triple‐Action Photosensitizer Nanoplatforms Orchestrate Cuproptosis‐Ferroptosis Synergy to Potentiate Antitumor PDT Efficacy.” Journal of Nanobiotechnology 24: 427. 10.1186/s12951-026-04331-3 41923244 PMC13169895

[581] Xu, Mengshu , Jingwei Liu , Lili Feng , Jiahe Hu , Wei Guo , Huiming Lin , Bin Liu , et al. 2025. “Designing a Sulfur Vacancy Redox Disruptor for Photothermoelectric and Cascade‐Catalytic‐Driven Cuproptosis‐Ferroptosis‐Apoptosis Therapy.” Nano‐Micro Letters 17: 321. 10.1007/s40820-025-01828-8 40613923 PMC12227396

[582] Chen, Mengyao , Chang Xu , Chunhui Wang , Nan Huang , Zhixuan Bian , Yixuan Xiao , Juan Ruan , Fenyong Sun , and Shuo Shi . 2024. “Three Birds With One Stone: Copper Ions Assisted Synergistic Cuproptosis/Chemodynamic/Photothermal Therapy by a Three‐Pronged Approach.” Advanced Healthcare Materials 13: e2401567. 10.1002/adhm.202401567 38962848

[583] Chin, Yu‐Cheng , Li‐Xing Yang , Fei‐Ting Hsu , Che‐Wei Hsu , Te‐Wei Chang , Hsi‐Ying Chen , Linda Yen‐Chien Chen , et al. 2022. “Iron Oxide@Chlorophyll Clustered Nanoparticles Eliminate Bladder Cancer by Photodynamic Immunotherapy‐Initiated Ferroptosis and Immunostimulation.” Journal of Nanobiotechnology 20: 373. 10.1186/s12951-022-01575-7 35953837 PMC9367122

[584] Cai, Jinming , Sheng Shi , Jinyan Hu , Zhenlin Zhang , Bijiang Geng , Dengyu Pan , and Longxiang Shen . 2026. “A Stimuli‐Responsive Cuproptosis Switch Boosts Persistent Immunotherapy for Tumor Eradication.” Biomaterials 329: 123930. 10.1016/j.biomaterials.2025.123930 41420963

[585] Fan, Zhijin , Sicheng Wu , Huaping Deng , Guanlin Li , Linghong Huang , and Hongxing Liu . 2024. “Light‐Triggered Nanozymes Remodel the Tumor Hypoxic and Immunosuppressive Microenvironment for Ferroptosis‐Enhanced Antitumor Immunity.” ACS Nano 18: 12261–12275. 10.1021/acsnano.4c00844 38683132

[586] Su, Tong , Guang Li , Yudong Guan , Qi Tian , Youzhi Qi , Yanqiu Zhang , Wenqian Wei , et al. 2026. “PROTAC‑Mediated HMGCR Depletion Reprograms Lipid Metabolism in Breast Cancer to Potentiate Photoimmunotherapy via Ferroptosis.” Advanced Science 13: e21525. 10.1002/advs.202521525 41486701 PMC12955944

[587] Gan, Yuehao , Wenteng Xie , Miaomiao Wang , Peng Wang , Qingdong Li , Junjie Cheng , Miao Yan , et al. 2024. “Cancer Cell Membrane‐Camouflaged CuPt Nanoalloy Boosts Chemotherapy of Cisplatin Prodrug to Enhance Anticancer Effect and Reverse Cisplatin Resistance of Tumor.” Materials Today Bio 24: 100941. 10.1016/j.mtbio.2023.100941 PMC10805937 38269055

[588] Sun, Mengqi , Yuchen Duan , Yumeng Ma , and Qingyuan Zhang . 2020. “ancer Cell‐Erythrocyte Hybrid Membrane Coated Gold Nanocages for Near Infrared Light‐Activated Photothermal/Radio/Chemotherapy of Breast Cancer.” International Journal of Nanomedicine 15: 6749–6760. 10.2147/IJN.S266405 32982231 PMC7494427

[589] Wan, Xiuyan , Liqun Song , Wei Pan , Hui Zhong , Na Li , and Bo Tang . 2020. “Tumor‐Targeted Cascade Nanoreactor Based on Metal‐Organic Frameworks for Synergistic Ferroptosis‐Starvation Anticancer Therapy.” ACS Nano 14: 11017–11028. 10.1021/acsnano.9b07789 32786253

[590] Zhang, Qingfei , Gaizhen Kuang , Hanbing Wang , Yuanjin Zhao , Jia Wei , and Luoran Shang . 2023. “Multi‐Bioinspired MOF Delivery Systems From Microfluidics for Tumor Multimodal Therapy.” Advanced Science 10: e2303818. 10.1002/advs.202303818 37852943 PMC10667824

[591] Zhao, Yuyue , Yuanwei Pan , Kelong Zou , Zhou Lan , Guowang Cheng , Qiuying Mai , Hao Cui , et al. 2023. “Biomimetic Manganese‐Based Theranostic Nanoplatform for Cancer Multimodal Imaging and Twofold Immunotherapy.” Bioactive Materials 19: 237–250. 10.1016/j.bioactmat.2022.04.011 35510176 PMC9048124

[592] Tian, Yu , Feng Wang , Jing Ma , Wei Huang , Xiaodan Zhang , Chengzhong Du , Penghui Wei , et al. 2025. “Targeting Ferroptosis and Mitophagy With Neutrophil‐Inspired Nanozyme for Parkinson's Disease Therapy.” Journal of Controlled Release 384: 113950. 10.1016/j.jconrel.2025.113950 40505893

[593] Ma, Guangyu , Silin Du , Xiang Li , Dehong Yu , Minghao Chao , Rongze Tang , Chaonan Jing , et al. 2026. “pH‐Responsive Neutrophil Membrane Camouflage Ga‐Mn Bimetallic Nanodecoy Triggers Apoptosis‐Immunity‐Metastasis Suppression for Tumor Therapy.” Biomaterials 327: 123794. 10.1016/j.biomaterials.2025.123794 41151369

[594] Chen, Kerong , Anwei Zhou , Xinyuan Zhou , Yuhang Liu , Yurui Xu , and Xinghai Ning . 2023. “An Intelligent Cell‐Derived Nanorobot Bridges Synergistic Crosstalk Between Sonodynamic Therapy and Cuproptosis to Promote Cancer Treatment.” Nano Letters 23: 3038–3047. 10.1021/acs.nanolett.3c00434 36951267

[595] Chen, Heying , Dongqing Wang , Jiahe Liu , Jun Chen , Yi Hu , and Yilu Ni . 2025. “Augmenting Antitumor Immune Effects Through the Coactivation of cGAS‐STING and NF‐kappaB Crosstalk in Dendritic Cells and Macrophages by Engineered Manganese Ferrite Nanohybrids.” ACS Applied Materials & Interfaces 17: 13375–13390. 10.1021/acsami.4c18570 39964151

[596] Wang, Zhongkai , Cheng Feng , Yong Wang , Enqi Qiao , Tian Huang , Junhao Mei , Tong Sun , et al. 2026. “A Manganese‐Based Biomimetic Theranostic Platform for ‘Root‐Eradicating’ Strategy via Pro‐Survival Autophagy Inhibition‐Enhanced Synergistic Antitumor Therapy.” Biomaterials 331: 124100. 10.1016/j.biomaterials.2026.124100 41812546

[597] Zhang, Yan , Weiting Sun , Xin Meng , Xiaohan Yang , Lina Su , Pengyu Huang , Dunwan Zhu , et al. 2026. “Efficient Copper Ion Transport Triggers In Situ Photothermia and Cuproptosis for Boosting Colon Cancer Immunotherapy.” Biomaterials 327: 123759. 10.1016/j.biomaterials.2025.123759 41045759

[598] Zhang, Ni , Wei Ping , Kexiang Rao , Zhenlin Zhang , Rong Huang , Daoming Zhu , Guoxin Li , and Shipeng Ning . 2024. “Biomimetic Copper‐Doped Polypyrrole Nanoparticles Induce Glutamine Metabolism Inhibition to Enhance Breast Cancer Cuproptosis and Immunotherapy.” Journal of Controlled Release 371: 204–215. 10.1016/j.jconrel.2024.05.045 38810704

[599] Han, Yu , WenWen Shen , YuYi Zhang , ZeQiang Jin , Jie Li , ZhongQuan Qi , and ShiFeng Zhang . 2026. “Nanodelivery Vectors Veiled in Natural Killer Cell Membranes: Enhancing Colorectal Cancer Therapy Through Synergistic Starvation and Chemotherapy.” ACS Applied Materials & Interfaces 18: 758–768. 10.1021/acsami.5c20905 41489079

[600] Li, Hao , Cheng Zhang , Yue Chen , Yingjie Xu , Wenjing Yao , and Wenpei Fan . 2024. “Biodegradable Long‐Circulating Nanoagonists Optimize Tumor‐Tropism Chemo‐Metalloimmunotherapy for Boosted Antitumor Immunity by Cascade cGAS‐STING Pathway Activation.” ACS Nano 18: 23711–23726. 10.1021/acsnano.4c08463 39148423

[601] Wu, Yanjie , Zhiyu Zhao , Mengli Ma , Weijin Zhang , Wei Liu , Xiaochen Liang , Ting Zhao , et al. 2025. “Ultrasound‐Activated Erythrocyte Membrane‐Camouflaged Pt (II) Layered Double Hydroxide Enhances PD‐1 Inhibitor Efficacy in Triple‐Negative Breast Cancer Through cGAS‐STING Pathway‐Mediated Immunogenic Cell Death.” Theranostics 15: 1456–1477. 10.7150/thno.102284 39816689 PMC11729553

[602] Long, Ying , Jialong Fan , Naduo Zhou , Jiahao Liang , Chang Xiao , Chunyi Tong , Wei Wang , and Bin Liu . 2023. “Biomimetic Prussian Blue Nanocomplexes for Chemo‐Photothermal Treatment of Triple‐Negative Breast Cancer by Enhancing ICD.” Biomaterials 303: 122369. 10.1016/j.biomaterials.2023.122369 37922746

[603] Li, Qiang , Meng Dang , Ao He , Xiaoye Li , Meng Ding , Zhuo Dai , Yu Zhang , et al. 2025. “Hybrid Cell Membrane‐Functionalized Nanoagents Synergistically Enhance Cuproptosis‐Mediated Immunotherapy by Dual Modulation of Glycolytic Metabolism and Tumor Microenvironments.” ACS Nano 19: 28913–28932. 10.1021/acsnano.5c10671 40755199 PMC12356196

[604] Cheng, Xiao , Junming Dong , Pramath Jain , Shuheng Qin , Yufei Miao , Kai Liu , Maria Lucia Veronica Theja , et al. 2026. “Ultrasound Activated Hybrid‐Biomimetic Nanocarriers That Combine Tumor‐Confined CRISPR/Cas9 Metabolic Reprogramming and Cuproptosis With Anticancer Macrophage Polarization.” Small 22: e10436. 10.1002/smll.202510436 41474015

[605] Guo, Shuai , Tianwang Guan , Yushen Ke , Yuping Lin , Rundong Tai , Jujian Ye , Zhilin Deng , Shaohui Deng , and Caiwen Ou . 2025. “Biologically Logic‐Gated Trojan‐Horse Strategy for Personalized Triple‐Negative Breast Cancer Precise Therapy by Selective Ferroptosis and STING Pathway Provoking.” Biomaterials 315: 122905. 10.1016/j.biomaterials.2024.122905 39471713

[606] Zhang, Fangming , Ziyao Zhang , Wanting Yang , Zhuyuan Peng , Juntao Sun , Guofeng Li , Yen Wei , et al. 2025. “Engineering Autologous Cell‐Derived Exosomes to Boost Melanoma‐Targeted Radio‐Immunotherapy by Cascade cGAS‐STING Pathway Activation.” Small 21: e2408769. 10.1002/smll.202408769 39604223

[607] Wei, Xiaoqing , Yongxin Zhang , Yuxin Chen , Zhongzhu Mo , Bin Jiang , Jingya Zhao , and Shaobing Zhou . 2026. “Multifunctional Exosome‐Driven Tumor Immunotherapy Sensitization: Converting Intratumoral Bacteria Into Antitumor Fighters.” Biomaterials 327: 123729. 10.1016/j.biomaterials.2025.123729 41022013

[608] Jia, Wenhui , Hailong Tian , Jingwen Jiang , Li Zhou , Lei Li , Maochao Luo , Ning Ding , et al. 2023. “Brain‐Targeted HFn‐Cu‐REGO Nanoplatform for Site‐Specific Delivery and Manipulation of Autophagy and Cuproptosis in Glioblastoma.” Small 19: e2205354. 10.1002/smll.202205354 36399643

[609] Liang, Minmin , Kelong Fan , Meng Zhou , Demin Duan , Jiyan Zheng , Dongling Yang , Jing Feng , and Xiyun Yan . 2014. “H‐Ferritin–Nanocaged Doxorubicin Nanoparticles Specifically Target and Kill Tumors With a Single‐Dose Injection.” Proceedings of the National Academy of Sciences 111: 14900–14905. 10.1073/pnas.1407808111 PMC4205604 25267615

[610] Qu, Chang , Xinyue Shao , Ran Jia , Guoqiang Song , Donghong Shi , Hui Wang , Jinping Wang , and Hailong An . 2024. “Hypoxia Reversion and STING Pathway Activation Through Large Mesoporous Nanozyme for Near‐Infrared‐II Light Amplified Tumor Polymetallic‐Immunotherapy.” ACS Nano 18: 22153–22171. 10.1021/acsnano.4c05483 39118372

[611] Miao, Yao , Tao Yang , Shuxu Yang , Mingying Yang , and Chuanbin Mao . 2022. “Protein Nanoparticles Directed Cancer Imaging and Therapy.” Nano Convergence 9: 2. 10.1186/s40580-021-00293-4 34997888 PMC8742799

[612] Ji, Qingzhi , Huimin Zhu , Yuting Qin , Ruiya Zhang , Lei Wang , Erhao Zhang , Xiaorong Zhou , and Run Meng . 2024. “GP60 and SPARC as Albumin Receptors: Key Targeted Sites for the Delivery of Antitumor Drugs.” Frontiers in Pharmacology 15: 1329636. 10.3389/fphar.2024.1329636 38323081 PMC10844528

[613] Yang, Shangqin , Jingxuan Wang , Kerong Tu , Xiaobing Huang , Liangliang Lv , Mingjie Peng , Qiqi Xu , et al. 2026. “AML‐Targeted Metal‐Polyphenol Nanoplatform Induces Ferroptosis‐ICD Cascade for Antitumor Immunity Boosting.” Advanced Science 13: e20544. 10.1002/advs.202520544 41773746 PMC13170192

[614] Chen, Yuan , Shan Wang , Congxiu Mao , Qinyi Lu , Xingyu Zhu , Dongqi Fan , Yiping Liu , et al. 2025. “5‐ALA Photodynamic Metabolite‐Powered Zero‐Waste Ferroptosis Amplifier for Enhanced Hypertrophic Scar Therapy.” Nature Communications 16: 8321. 10.1038/s41467-025-63438-7 PMC12446439 40968167

[615] Lin, Xin , Jin Xie , Gang Niu , Fan Zhang , Haokao Gao , Min Yang , Qimeng Quan , et al. 2011. “Chimeric Ferritin Nanocages for Multiple Function Loading and Multimodal Imaging.” Nano Letters 11: 814–819. 10.1021/nl104141g 21210706 PMC3036786

[616] Luo, Siyuan , Yueyan Yang , Liuting Chen , Perumal Ramesh Kannan , Weilin Yang , Yongjia Zhang , Ruibo Zhao , et al. 2024. “Outer Membrane Vesicle‐Wrapped Manganese Nanoreactor for Augmenting Cancer Metalloimmunotherapy Through Hypoxia Attenuation and Immune Stimulation.” Acta Biomaterialia 181: 402–414. 10.1016/j.actbio.2024.05.010 38734282

[617] Ma, Xiaotu , Xiaolong Liang , Yao Li , Qingqing Feng , Keman Cheng , Nana Ma , Fei Zhu , et al. 2023. “Modular‐Designed Engineered Bacteria for Precision Tumor Immunotherapy via Spatiotemporal Manipulation by Magnetic Field.” Nature Communications 14: 1606. 10.1038/s41467-023-37225-1 PMC10036336 36959204

[618] Kang, Ruixin , Xiuhua Pan , Xiawei Zhou , Yueru Pang , Ziqi Shen , Lin Luo , Feiyang Liu , Siyuan Yu , and Qi Shen . 2026. “Programmable Bacteria‐Driven Biohybrid Triggers Spatiotemporal‐Controlled STING Activation to Potentiate Cuproptosis‐Based Cancer Therapy.” Biomaterials 328: 123893. 10.1016/j.biomaterials.2025.123893 41401569

[619] Xie, Beibei , Huichao Zhao , Yuan‐Fu Ding , Ziyi Wang , Cheng Gao , Shengke Li , Kehan Zhang , et al. 2023. “Supramolecularly Engineered Conjugate of Bacteria and Cell Membrane‐Coated Magnetic Nanoparticles for Enhanced Ferroptosis and Immunotherapy of Tumors.” Advanced Science 10: e2304407. 10.1002/advs.202304407 37850572 PMC10700203

[620] Zhang, Fan , Qianqian Li , Haibing Dai , Weiqun Li , Xiang Chen , Huibin Wu , Shanming Lu , et al. 2025. “Chimeric Nanozyme Bacterial Outer Membrane Vesicles Reprograming Tumor Microenvironment for Safe and Efficient Anticancer Therapy.” Advanced Science 12: e2417712. 10.1002/advs.202417712 40278503 PMC12165108

[621] Shen, Ziqi , Yueru Pang , Ruixin Kang , Xiawei Zhou , Chao Li , Siyuan Yu , Lin Luo , et al. 2025. “Outer Membrane Vesicle‐Coated Ferrocene Nanoparticles Induce Dual Ferroptosis for Cancer Immunotherapy.” Journal of Controlled Release 387: 114203. 10.1016/j.jconrel.2025.114203 40921256

[622] Wu, Yalong , Yuhan Zhang , Jiansong Han , Yongnan Jiang , Maolong Chen , Xinquan Gu , Wei Jiang , Kelong Fan , and Bin Liu . 2025. “Nanozyme‐Based Biomimetic Intelligent Immune Organelles for the Treatment of Bladder‐Metastasized Tumors.” Advanced Materials 37: e11181. 10.1002/adma.202511181 40810617

[623] Yu, Yangyang , Ying Wang , Jin Zhang , Qingyue Bu , Dan Jiang , Yalin Jiang , Ligeng Xu , Zhenyu Ju , and Tianfeng Chen . 2025. “Anaerobic Probiotics‐In Situ Se Nanoradiosensitizers Selectively Anchor to Tumor With Immuno‐Regulations for Robust Cancer Radio‐Immunotherapy.” Biomaterials 318: 123117. 10.1016/j.biomaterials.2025.123117 39864125

[624] Wang, Wenqi , Yuyu Cui , Xiaolong Wei , Ying Zang , Xulin Chen , Liang Cheng , and Xianwen Wang . 2024. “CuCo2O4 Nanoflowers with Multiple Enzyme Activities for Treating Bacterium‐Infected Wounds via Cuproptosis‐Like Death.” ACS Nano 18: 15845–15863. 10.1021/acsnano.4c02825 38832685

[625] Mei, Jiawei , Dongdong Xu , Lingtian Wang , Lingtong Kong , Quan Liu , Qianming Li , Xianzuo Zhang , et al. 2023. “Biofilm Microenvironment‐Responsive Self‐Assembly Nanoreactors for All‐Stage Biofilm Associated Infection Through Bacterial Cuproptosis‐Like Death and Macrophage Re‐Rousing.” Advanced Materials 35: e2303432. 10.1002/adma.202303432 37262064

[626] Chu, Zhaoyou , Wang Zheng , Wanyue Fu , Jun Liang , Wanni Wang , Lingling Xu , Xiaohua Jiang , Zhengbao Zha , and Haisheng Qian . 2025. “Implanted Microneedles Loaded With Sparfloxacin and Zinc‐Manganese Sulfide Nanoparticles Activates Immunity for Postoperative Triple‐Negative Breast Cancer to Prevent Recurrence and Metastasis.” Advanced Science 12: e2416270. 10.1002/advs.202416270 40042034 PMC12021102

[627] Amaral, Eduardo P. , Diego L. Costa , Sivaranjani Namasivayam , Nicolas Riteau , Olena Kamenyeva , Lara Mittereder , Katrin D. Mayer‐Barber , Bruno B. Andrade , and Alan Sher . 2019. “A Major Role for Ferroptosis in *Mycobacterium tuberculosis*‐Induced Cell Death and Tissue Necrosis.” Journal of Experimental Medicine 216: 556–570. 10.1084/jem.20181776 30787033 PMC6400546

[628] Tang, Zhongmin , Yanyan Liu , Mingyuan He , and Wenbo Bu . 2019. “Chemodynamic Therapy: Tumour Microenvironment‐Mediated Fenton and Fenton‐Like Reactions.” Angewandte Chemie International Edition 58: 946–956. 10.1002/anie.201805664 30048028

[629] Lin, Li‐Sen , Jibin Song , Liang Song , Kaimei Ke , Yijing Liu , Zijian Zhou , Zheyu Shen , et al. 2018. “Simultaneous Fenton‐Like Ion Delivery and Glutathione Depletion by MnO(2) ‐Based Nanoagent to Enhance Chemodynamic Therapy.” Angewandte Chemie International Edition 57: 4902–4906. 10.1002/anie.201712027 29488312

[630] Efimova, Iuliia , Elena Catanzaro , Louis Van der Meeren , Victoria D. Turubanova , Hamida Hammad , Tatiana A. Mishchenko , Maria V. Vedunova , et al. 2020. “Vaccination With Early Ferroptotic Cancer Cells Induces Efficient Antitumor Immunity.” Journal for ImmunoTherapy of Cancer 8: e001369. 10.1136/jitc-2020-001369 33188036 PMC7668384

[631] Wen, Qirong , Jiao Liu , Rui Kang , Borong Zhou , and Daolin Tang . 2019. “The Release and Activity of HMGB1 in Ferroptosis.” Biochemical and Biophysical Research Communications 510: 278–283. 10.1016/j.bbrc.2019.01.090 30686534

[632] Chen, Fangquan , Rui Kang , Daolin Tang , and Jiao Liu . 2024. “Ferroptosis: Principles and Significance in Health and Disease.” Journal of Hematology & Oncology 17: 41. 10.1186/s13045-024-01564-3 38844964 PMC11157757

[633] Lv, Lijie , Yue Wang , Xuan Lv , and Qiuli Miao . 2025. “Involvement of HMGB1‐Mediated Ferroptosis in Systemic Diseases.” Frontiers in Cell and Developmental Biology 13: 1676941. 10.3389/fcell.2025.1676941 41113461 PMC12531243

[634] Green, Douglas R. , Thomas Ferguson , Laurence Zitvogel , and Guido Kroemer . 2009. “Immunogenic and Tolerogenic Cell Death.” Nature Reviews Immunology 9: 353–363. 10.1038/nri2545 PMC2818721 19365408

[635] Wu, Hangyi , Zhenhai Zhang , Yanni Cao , Yuhan Hu , Yi Li , Lanyi Zhang , Xinyi Cao , et al. 2024. “A Self‐Amplifying ROS‐Responsive Nanoplatform for Simultaneous Cuproptosis and Cancer Immunotherapy.” Advanced Science 11: e2401047. 10.1002/advs.202401047 38569217 PMC11187900

[636] Krysko, Dmitri V. , Abhishek D. Garg , Agnieszka Kaczmarek , Olga Krysko , Patrizia Agostinis , and Peter Vandenabeele . 2012. “Immunogenic Cell Death and DAMPs in Cancer Therapy.” Nature Reviews Cancer 12: 860–875. 10.1038/nrc3380 23151605

[637] Martins, I. , O. Kepp , F. Schlemmer , S. Adjemian , M. Tailler , S. Shen , M. Michaud , et al. 2011. “Restoration of the Immunogenicity of Cisplatin‐Induced Cancer Cell Death by Endoplasmic Reticulum Stress.” Oncogene 30: 1147–1158. 10.1038/onc.2010.500 21151176

[638] Dondelinger, Yves , Dario Priem , Jon Huyghe , Tom Delanghe , Peter Vandenabeele , Mathieu J , and M., Bertrand . 2023. “NINJ1 Is Activated by Cell Swelling to Regulate Plasma Membrane Permeabilization During Regulated Necrosis.” Cell Death & Disease 14: 755. 10.1038/s41419-023-06284-z 37980412 PMC10657445

[639] Zeng, Guicheng , Jinning Mao , Haiyan Xing , Zhigang Xu , Zhong Cao , Yuejun Kang , Guodong Liu , and Peng Xue . 2024. “Gold Nanodots‐Anchored Cobalt Ferrite Nanoflowers as Versatile Tumor Microenvironment Modulators for Reinforced Redox Dyshomeostasis.” Advanced Science 11: e2406683. 10.1002/advs.202406683 38984397 PMC11529044

[640] Zhang, Xuan , Jinwei Bai , Shihao Sun , Yu Li , Xinxin Li , Genping Meng , Wenyuan Cheng , et al. 2025. “Chiral Nanoassembly Remodels Tumor Microenvironment Through Non‐Oxygen‐Dependent Depletion Lactate for Eﬀective Photodynamic Immunotherapy.” Biomaterials 319: 123203. 10.1016/j.biomaterials.2025.123203 40009900

[641] Liu, Jiao , Yang Liu , Yuan Wang , Rui Kang , and Daolin Tang . 2022. “HMGB1 Is a Mediator of Cuproptosis‐Related Sterile Inflammation.” Frontiers in Cell and Developmental Biology 10: 996307. 10.3389/fcell.2022.996307 36211458 PMC9534480

[642] Zhang, Fan , Feng Li , Gui‐Hong Lu , Weidong Nie , Lijun Zhang , Yanlin Lv , Weier Bao , et al. 2019. “Engineering Magnetosomes for Ferroptosis/Immunomodulation Synergism in Cancer.” ACS Nano 13: 5662–5673. 10.1021/acsnano.9b00892 31046234

[643] Wiernicki, Bartosz , Sophia Maschalidi , Jonathan Pinney , Sandy Adjemian , Tom Vanden Berghe , Kodi S. Ravichandran , and Peter Vandenabeele . 2022. “Cancer Cells Dying From Ferroptosis Impede Dendritic Cell‐Mediated Anti‐Tumor Immunity.” Nature Communications 13: 3676. 10.1038/s41467-022-31218-2 PMC9237053 35760796

[644] Li, Pengchong , Mengdi Jiang , Ketian Li , Hao Li , Yangzhong Zhou , Xinyue Xiao , Yue Xu , et al. 2021. “Glutathione Peroxidase 4‐Regulated Neutrophil Ferroptosis Induces Systemic Autoimmunity.” Nature Immunology 22: 1107–1117. 10.1038/s41590-021-00993-3 34385713 PMC8609402

[645] Tang, Cong , Kairui Liu , Xiaoning Gao , Hanmeixuan Kang , Weijie Xie , Jin Chang , Linling Yin , and Jun Kang . 2025. “A Metal‐Organic Framework Functionalized CaO(2)‐Based Cascade Nanoreactor Induces Synergistic Cuproptosis/Ferroptosis and Ca^2+^ Overload‐Mediated Mitochondrial Damage for Enhanced Sono‐Chemodynamic Immunotherapy.” Acta Biomaterialia 193: 455–473. 10.1016/j.actbio.2024.12.010 39637958

[646] Shen, Yinjing , Nuo Yu , Wenjing Zhao , Shining Niu , Pu Qiu , Haiyan Zeng , Zhigang Chen , Wei Men , and Dong Xie . 2025. “M1‐macrophage Membrane‐Camouflaged Nanoframeworks Activate Multiple Immunity via Calcium Overload and Photo‐Sonosensitization.” Biomaterials 320: 123287. 10.1016/j.biomaterials.2025.123287 40147112

[647] Ding, Jingjin , Kun Wang , Wang Liu , Yang She , Qi Sun , Jianjin Shi , Hanzi Sun , Da‐Cheng Wang , and Feng Shao . 2016. “Pore‐Forming Activity and Structural Autoinhibition of the Gasdermin Family.” Nature 535: 111–116. 10.1038/nature18590 27281216

[648] Chen, Xin , Andrey S. Tsvetkov , Han‐Ming Shen , Ciro Isidoro , Nicholas T. Ktistakis , Andreas Linkermann , Werner J. H. Koopman , et al. 2024. “International Consensus Guidelines for the Definition, Detection, and Interpretation of Autophagy‐Dependent Ferroptosis.” Autophagy 20: 1213–1246. 10.1080/15548627.2024.2319901 38442890 PMC11210914

[649] Cho, Sungji , Eddie Tam , Khang Nguyen , Yubin Lei , Carine Fillebeen , Kostas Pantopoulos , Hye Kyoung Sung , and Gary Sweeney . 2025. “ω−6 PUFA‐Enriched Membrane Phospholipid Composition of Cardiomyocytes Increases the Susceptibility to Iron‐Induced Ferroptosis and Inflammation.” Apoptosis 30: 1614–1627. 10.1007/s10495-025-02121-0 40381101

[650] Ichihara, Genki , Yoshinori Katsumata , Yuki Sugiura , Yuta Matsuoka , Rae Maeda , Jin Endo , Atsushi Anzai , et al. 2023. “MRP1‐Dependent Extracellular Release of Glutathione Induces Cardiomyocyte Ferroptosis After Ischemia‐Reperfusion.” Circulation Research 133: 861–876. 10.1161/CIRCRESAHA.123.323517 37818671

[651] Li, Wenjun , Guoshuai Feng , Jason M. Gauthier , Inessa Lokshina , Ryuji Higashikubo , Sarah Evans , Xinping Liu , et al. 2019. “Ferroptotic Cell Death and TLR4/Trif Signaling Initiate Neutrophil Recruitment After Heart Transplantation.” The Journal of Clinical Investigation 129: 2293–2304. 10.1172/JCI126428 30830879 PMC6546457

[652] Zhang, Jiong , Qing Li , Yu‐Rong Zou , Shu‐kun Wu , Xiang‐heng Lu , Gui‐sen Li , and Jia Wang . 2021. “HMGB1‐TLR4‐IL‐23‐IL‐17A Axis Accelerates Renal Ischemia‐Reperfusion Injury via the Recruitment and Migration of Neutrophils.” International Immunopharmacology 94: 107433. 10.1016/j.intimp.2021.107433 33592404

[653] Guo, Lili , Dong Zhang , Xiaoyan Ren , and Dingsheng Liu . 2023. “SYVN1 Attenuates Ferroptosis and Alleviates Spinal Cord Ischemia–Reperfusion Injury in Rats by Regulating the HMGB1/NRF2/HO‐1 Axis.” International Immunopharmacology 123: 110802. 10.1016/j.intimp.2023.110802 37591122

[654] Liu, Zan , Yuxiang Zhou , Ming Li , Zhenghui Xiao , Zitong Zhao , and Yong Li . 2025. “Dimeric PKM2 Induces Ferroptosis from Intestinal Ischemia/Reperfusion in Mice by Histone H4 Lysine 12 Lactylation‐Mediated HMGB1 Transcription Activation Through the Lactic Acid/p300 Axis.” Biochimica et Biophysica Acta (BBA) ‐ Molecular Basis of Disease 1871: 167998. 10.1016/j.bbadis.2025.167998 40738462

[655] Fu, Biqi , Zhihui Fu , Zhongzhong Liu , Qin Deng , Fuping Cao , Jiansheng Xiao , Xingjian Zhang , and Qi Xiao . 2026. “Mild Hypothermia Alleviates Ferroptosis in Kidney Ischemia‐Reperfusion Injury via the Glycolysis‐Lactate‐HMGB1 Lactylation Axis.” Cellular Signalling 143: 112475. 10.1016/j.cellsig.2026.112475 41806899

[656] Ruan, Yongle , Lu Wang , Yue Zhao , Ying Yao , Song Chen , Junhua Li , Hui Guo , et al. 2014. “Carbon Monoxide Potently Prevents Ischemia‐Induced High‐Mobility Group Box 1 Translocation and Release and Protects Against Lethal Renal Ischemia–Reperfusion Injury.” Kidney International 86: 525–537. 10.1038/ki.2014.80 24694987

[657] Wei, Qi , Huanyu Zhou , Jie Sun , and Ling Qin . 2026. “SLC31A1 Knockdown Mitigates post‐MI Heart Failure via Regulation of Copper Metabolism.” Frontiers in Immunology 17: 1707203. 10.3389/fimmu.2026.1707203 41808820 PMC12968310

[658] Tang, Yun , Haojun Luo , Qiong Xiao , Li Li , Xiang Zhong , Jiong Zhang , Fang Wang , et al. 2021. “Isoliquiritigenin Attenuates Septic Acute Kidney Injury by Regulating Ferritinophagy‐Mediated Ferroptosis.” Renal Failure 43: 1551–1560. 10.1080/0886022X.2021.2003208 34791966 PMC8604484

[659] Jin, Xiaoming , Riming He , Yunxin Lin , Jiahui Liu , Yuzhi Wang , Zhongtang Li , Yijiao Liao , and Shudong Yang . 2023. “Shenshuaifu Granule Attenuates Acute Kidney Injury by Inhibiting Ferroptosis Mediated by p53/SLC7A11/GPX4 Pathway.” Drug Design, Development and Therapy 17: 3363–3383. 10.2147/DDDT.S433994 38024532 PMC10656853

[660] Zhu, Kaiyi , Rong Fan , Yuchen Cao , Wei Yang , Zhe Zhang , Qiang Zhou , Jie Ren , et al. 2024. “Glycyrrhizin Attenuates Myocardial Ischemia Reperfusion Injury by Suppressing Inflammation, Oxidative Stress, and Ferroptosis via the HMGB1‐TLR4‐GPX4 Pathway.” Experimental Cell Research 435: 113912. 10.1016/j.yexcr.2024.113912 38176464

[661] Wang, Bo , Li‐na Yang , Le‐tian Yang , Yan Liang , Fan Guo , Ping Fu , Liang Ma , and Liang Ma . 2024. “Fisetin Ameliorates Fibrotic Kidney Disease in Mice via Inhibiting ACSL4‐Mediated Tubular Ferroptosis.” Acta Pharmacologica Sinica 45: 150–165. 10.1038/s41401-023-01156-w 37696989 PMC10770410

[662] Jhelum, Priya , Stephanie Zandee , Fari Ryan , Juan G. Zarruk , Bernhard Michalke , Vivek Venkataramani , Laura Curran , et al. 2023. “Ferroptosis Induces Detrimental Effects in Chronic EAE and Its Implications for Progressive MS.” Acta Neuropathologica Communications 11: 121. 10.1186/s40478-023-01617-7 37491291 PMC10369714

[663] Liu, Yan , Xiqing Luo , Ye Chen , Junlong Dang , Donglan Zeng , Xinghua Guo , Weizhen Weng , et al. 2024. “Heterogeneous Ferroptosis Susceptibility of Macrophages Caused by Focal Iron Overload Exacerbates Rheumatoid Arthritis.” Redox Biology 69: 103008. 10.1016/j.redox.2023.103008 38142586 PMC10788633

[664] Rousselle, Anthony , Dörte Lodka , Janis Sonnemann , Lovis Kling , Ralph Kettritz , and Adrian Schreiber . 2025. “Endothelial but Not Systemic Ferroptosis Inhibition Protects From Antineutrophil Cytoplasmic Antibody‐Induced Crescentic Glomerulonephritis.” Kidney International 107: 1037–1050. 10.1016/j.kint.2025.02.023 40122342

[665] Galluzzi, Lorenzo , Aitziber Buqué , Oliver Kepp , Laurence Zitvogel , and Guido Kroemer . 2017. “Immunogenic Cell Death in Cancer and Infectious Disease.” Nature Reviews Immunology 17: 97–111. 10.1038/nri.2016.107 27748397

[666] Ackerman, Shelley E. , Cecelia I. Pearson , Joshua D. Gregorio , Joseph C. Gonzalez , Justin A. Kenkel , Felix J. Hartmann , Angela Luo , et al. 2021. “Immune‐Stimulating Antibody Conjugates Elicit Robust Myeloid Activation and Durable Antitumor Immunity.” Nature Cancer 2: 18–33. 10.1038/s43018-020-00136-x 35121890 PMC9012298

[667] Ma, Guiqi , Xinyu Zhang , Kunlong Zhao , Shuxuan Zhang , Ke Ren , Mengyao Mu , Chenyu Wang , et al. 2024. “Polydopamine Nanostructure‐Enhanced Water Interaction With pH‐Responsive Manganese Sulfide Nanoclusters for Tumor Magnetic Resonance Contrast Enhancement and Synergistic Ferroptosis‐Photothermal Therapy.” ACS Nano 18: 3369–3381. 10.1021/acsnano.3c10249 38251846

[668] Zhang, Ke , Chengyao Huang , Yu Ren , Mingyue Zhang , Xiaotong Lu , Bangliu Yang , Peiran Chen , et al. 2025. “Manganese‐Based Nanoadjuvants for the Synergistic Enhancement of Immune Responses in Breast Cancer Therapy via Disulfidptosis‐Induced ICD and cGAS‐STING Activation.” Biomaterials 322: 123359. 10.1016/j.biomaterials.2025.123359 40288315

[669] Zhu, Lingfang , Lei Xu , Chenguang Wang , Changfu Li , Mengyuan Li , Qinmeng Liu , Xiao Wang , et al. 2021. “T6SS Translocates a Micropeptide to Suppress STING‐Mediated Innate Immunity by Sequestering Manganese.” Proceedings of the National Academy of Sciences 118: e2103526118. 10.1073/pnas.2103526118 PMC8545469 34625471

[670] Qian, Kaiqiang , Lidong Shan , Shengwen Shang , Tianyue Li , Shuxin Wang , Meili Wei , Bikui Tang , and Jun Xi . 2022. “Manganese Enhances Macrophage Defense Against Mycobacterium Tuberculosis via the STING‐TNF Signaling Pathway.” International Immunopharmacology 113: 109471. 10.1016/j.intimp.2022.109471 36435065

[671] Liu, Jianping , Jiezhao Zhan , Ye Zhang , Lin Huang , Jing Yang , Jie Feng , Lingwen Ding , Zheyu Shen , and Xiaoyuan Chen . 2024. “Ultrathin Clay Nanoparticles‐Mediated Mutual Reinforcement of Ferroptosis and Cancer Immunotherapy.” Advanced Materials 36: e2309562. 10.1002/adma.202309562 37939375

[672] Qiu, Chong , Fei Xia , Qingchao Tu , Huan Tang , Yinan Liu , Hongda Liu , Chen Wang , et al. 2025. “Multimodal Lung Cancer Theranostics via Manganese Phosphate/Quercetin Particle.” Molecular Cancer 24: 43. 10.1186/s12943-025-02242-9 39905491 PMC11796208

[673] Luo, Guanghong , Xing Li , Jihui Lin , Gao Ge , Jiangli Fang , Wangze Song , Gary Guishan Xiao , et al. 2023. “Multifunctional Calcium‐Manganese Nanomodulator Provides Antitumor Treatment and Improved Immunotherapy via Reprogramming of the Tumor Microenvironment.” ACS Nano 17: 15449–15465. 10.1021/acsnano.3c01215 37530575 PMC10448754

[674] Mendez‐Vazquez, Hadassah, Avanti Gokhale, Maureen M. Sampson, Felix G. Rivera Moctezuma, Adriana Harbuzariu, Anson Sing, Stephanie A. Zlatic, et al. 2026. “Mitochondrially Transcribed DsRNA Mediates Manganese‐Induced Neuroinflammation.” The Journal of Neuroscience 46: e1936252026. 10.1523/JNEUROSCI.1936-25.2026 42167917 PMC13255918

[675] Lu, Yang , Liang Gao , Yuqing Yang , Dihang Shi , Zhipeng Zhang , Xiaobai Wang , Ying Huang , et al. 2025. “Protective Role of Mitophagy on Microglia‐Mediated Neuroinflammatory Injury Through mtDNA‐STING Signaling in Manganese‐Induced Parkinsonism.” Journal of Neuroinflammation 22: 55. 10.1186/s12974-025-03396-5 40022162 PMC11869743

[676] Wu, Jinxia , Honggang Chen , Tingting Guo , Ming Li , Changhao Yang , Michael Aschner , Jingyuan Chen , Peng Su , and Wenjing Luo . 2023. “Sesamol Alleviates Manganese‐Induced Neuroinflammation and Cognitive Impairment via Regulating the Microglial cGAS‐STING/NF‐κB Pathway.” Environmental Pollution 319: 120988. 10.1016/j.envpol.2022.120988 36596376

[677] Sharma, Padmanee , Siwen Hu‐Lieskovan , Jennifer A. Wargo , and Antoni Ribas . 2017. “Primary, Adaptive, and Acquired Resistance to Cancer Immunotherapy.” Cell 168: 707–723. 10.1016/j.cell.2017.01.017 28187290 PMC5391692

[678] Herbst, Roy S. , Jean‐Charles Soria , Marcin Kowanetz , Gregg D. Fine , Omid Hamid , Michael S. Gordon , Jeffery A. Sosman , et al. 2014. “Predictive Correlates of Response to the Anti‐PD‐L1 Antibody MPDL3280A in Cancer Patients.” Nature 515: 563–567. 10.1038/nature14011 25428504 PMC4836193

[679] De Henau, Olivier , Matthew Rausch , David Winkler , Luis Felipe Campesato , Cailian Liu , Daniel Hirschhorn Cymerman , Sadna Budhu , et al. 2016. “Overcoming Resistance to Checkpoint Blockade Therapy by Targeting PI3Kγ in Myeloid Cells.” Nature 539: 443–447. 10.1038/nature20554 27828943 PMC5634331

[680] Yang, Fan , Yi Xiao , Jia‐Han Ding , Xi Jin , Ding Ma , Da‐Qiang Li , Jin‐Xiu Shi , et al. 2023. “Ferroptosis Heterogeneity in Triple‐Negative Breast Cancer Reveals an Innovative Immunotherapy Combination Strategy.” Cell Metabolism 35: 84–100.e8. 10.1016/j.cmet.2022.09.021 36257316

[681] Zheng, Yuzhao , Fanxue Bu , Chenfeng Xu , Tongyu Wu , Jianping Zhou , Weiyang Shen , and Tingjie Yin . 2024. “A Coordinative Modular Assembly‐Constructed Self‐Reinforced Nano‐Therapeutic Agent for Effective Antitumor‐Immune Activation.” Journal of Controlled Release 371: 588–602. 10.1016/j.jconrel.2024.06.020 38866245

[682] Kapralov, Alexandr A. , Qin Yang , Haider H. Dar , Yulia Y. Tyurina , Tamil S. Anthonymuthu , Rina Kim , Claudette M. St. Croix , et al. 2020. “Redox Lipid Reprogramming Commands Susceptibility of Macrophages and Microglia to Ferroptotic Death.” Nature Chemical Biology 16: 278–290. 10.1038/s41589-019-0462-8 32080625 PMC7233108

[683] Li, Ke , Kun Xu , Ye He , Yulu Yang , Meijun Tan , Yulan Mao , Yanan Zou , et al. 2023. “Oxygen Self‐Generating Nanoreactor Mediated Ferroptosis Activation and Immunotherapy in Triple‐Negative Breast Cancer.” ACS Nano 17: 4667–4687. 10.1021/acsnano.2c10893 36861638

[684] Xie, Luoyingzi , Jie Gong , Zhiqiang He , Weinan Zhang , Haoyu Wang , Shitao Wu , Xianxing Wang , et al. 2025. “A Copper‐Manganese Based Nanocomposite Induces Cuproptosis and Potentiates Anti‐Tumor Immune Responses.” Small 21: e2412174. 10.1002/smll.202412174 39955646

[685] Wu, Linying , Wenmin Pi , Xuemei Huang , Luping Yang , Xiang Zhang , Jihui Lu , Shuchang Yao , et al. 2025. “Orchestrated Metal‐Coordinated Carrier‐Free Celastrol Hydrogel Intensifies T Cell Activation and Regulates Response to Immune Checkpoint Blockade for Synergistic Chemo‐Immunotherapy.” Biomaterials 312: 122723. 10.1016/j.biomaterials.2024.122723 39121732

[686] Liu, Tiantian , Zehang Zhou , Mengxing Zhang , Puxin Lang , Jing Li , Zhenmi Liu , Zhirong Zhang , Lin Li , and Ling Zhang . 2023. “Cuproptosis‐Immunotherapy Using PD‐1 Overexpressing T Cell Membrane‐Coated Nanosheets Efficiently Treats Tumor.” Journal of Controlled Release 362: 502–512. 10.1016/j.jconrel.2023.08.055 37652367

[687] Gao, Si , Haodong Ge , Lili Gao , Ying Gao , Shuibin Tang , Yiming Li , Zhiqing Yuan , and Wei Chen . 2025. “Silk Fibroin Nanoparticles for Enhanced Cuproptosis and Immunotherapy in Pancreatic Cancer Treatment.” Advanced Science 12: e2417676. 10.1002/advs.202417676 40091480 PMC12079484

[688] Wang, Zhihua , Mingda Han , Yiqiao Wang , Ning Wang , Yilin Yang , Bingru Shao , Qiannan Miao , et al. 2025. “UiO‐66 MOFs‐Based ‘Epi‐Nano‐Sonosensitizer’ for Ultrasound‐Driven Cascade Immunotherapy against B‐Cell Lymphoma.” ACS Nano 19: 6282–6298. 10.1021/acsnano.4c15761 39920081

[689] Wawrzyniak, Piotr , and Mariusz L. Hartman . 2025. “Dual Role of Interferon‐Gamma in the Response of Melanoma Patients to Immunotherapy With Immune Checkpoint Inhibitors.” Molecular Cancer 24: 89. 10.1186/s12943-025-02294-x 40108693 PMC11924818

[690] Duan, Xiaopin , Christina Chan , and Wenbin Lin . 2019. “Nanoparticle‐Mediated Immunogenic Cell Death Enables and Potentiates Cancer Immunotherapy.” Angewandte Chemie International Edition 58: 670–680. 10.1002/anie.201804882 30016571 PMC7837455

[691] Chen, Chao , Zaiyu Wang , Shaorui Jia , Yuan Zhang , Shenglu Ji , Zheng Zhao , Ryan T. K. Kwok , et al. 2022. “Evoking Highly Immunogenic Ferroptosis Aided by Intramolecular Motion‐Induced Photo‐Hyperthermia for Cancer Therapy.” Advanced Science 9: 2104885. 10.1002/advs.202104885 35132824 PMC8981454

[692] Wang, Run , Yuyang Tian , Xuliang Lu , Leyi Fang , Yinxing Miao , Daqing Fang , Yingxia Li , Hong Liu , and Deju Ye . 2025. “Pretargeted Mitochondrial Delivery of Organoarsenicals for Cancer Immunotherapy.” Journal of the American Chemical Society 147: 38534–38548. 10.1021/jacs.5c12201 41077966

[693] Yee, Patricia P. , Yiju Wei , Soo‐Yeon Kim , Tong Lu , Stephen Y. Chih , Cynthia Lawson , Miaolu Tang , et al. 2020. “Neutrophil‐Induced Ferroptosis Promotes Tumor Necrosis in Glioblastoma Progression.” Nature Communications 11: 5424. 10.1038/s41467-020-19193-y PMC7591536 33110073

[694] Veglia, Filippo , Vladimir A. Tyurin , Dariush Mohammadyani , Maria Blasi , Elizabeth K. Duperret , Laxminarasimha Donthireddy , Ayumi Hashimoto , et al. 2017. “Lipid Bodies Containing Oxidatively Truncated Lipids Block Antigen Cross‐Presentation by Dendritic Cells in Cancer.” Nature Communications 8: 2122. 10.1038/s41467-017-02186-9 PMC5730553 29242535

[695] Cao, Wei , Rupal Ramakrishnan , Vladimir A. Tuyrin , Filippo Veglia , Thomas Condamine , Andrew Amoscato , Dariush Mohammadyani , et al. 2014. “Oxidized Lipids Block Antigen Cross‐Presentation by Dendritic Cells in Cancer.” The Journal of Immunology 192: 2920–2931. 10.4049/jimmunol.1302801 24554775 PMC3998104

[696] Tuerxun, Halahati , Yixin Zhao , Yawen Li , Xingyu Liu , Shuhui Wen , and Yuguang Zhao . 2025. “Resveratrol Alleviates Testicular Toxicity Induced by Anti‐PD‐1 Through Regulating the NRF2‐SLC7A11‐GPX4 Pathway.” Frontiers in Immunology 16: 1529991. 10.3389/fimmu.2025.1529991 40145083 PMC11937136

[697] Cai, Yang , Lingchuan Deng , and Jun Yao . 2024. “Analysis and Identification of Ferroptosis‐Related Diagnostic Markers in Rheumatoid Arthritis.” Annals of Medicine 56: 2397572. 10.1080/07853890.2024.2397572 39221753 PMC11370691

[698] Feng, Zhuan , Feiyang Meng , Fei Huo , Yumeng Zhu , Yifei Qin , Yu Gui , Hai Zhang , et al. 2024. “Inhibition of Ferroptosis Rescues M2 Macrophages and Alleviates Arthritis by Suppressing the HMGB1/TLR4/STAT3 Axis in M1 Macrophages.” Redox Biology 75: 103255. 10.1016/j.redox.2024.103255 39029270 PMC11304870

[699] Liang, Bingxue , Long Li , Xiang Ma , Hua Xian , Chenglin Tang , Xuezhi Li , and Dan Wang . 2025. “The Nrf2‐GPX4 Pathway Provides Protection Against Rheumatoid Arthritis via Inhibition of Macrophage Ferroptosis‐Mediated Inflammation and Synovial Fibroblast Proliferation.” Journal of Biochemical and Molecular Toxicology 39: e70539. 10.1002/jbt.70539 41147649

[700] Liang, Lifang , Huaguo Liang , Min He , Huiling Zhang , and Peifeng Ke . 2025. “Integrative Multi‐Omics Analysis Reveals the Interaction Mechanisms between Gut Microbiota Metabolites and Ferroptosis in Rheumatoid Arthritis.” Frontiers in Immunology 16: 1608262. 10.3389/fimmu.2025.1608262 40703513 PMC12283288

[701] Wang, Xuefei , Xiaoxiao Han , Ruizhi Feng , Wenlin Qiu , Lijuan Jiang , Xiaoru Duan , and Guo‐Min Deng . 2025. “Pumilio2 Deficiency Promotes Iron‐Dependent Macrophage Inflammation via TfR1 Upregulation in Lupus.” Free Radical Biology and Medicine 239: 513–527. 10.1016/j.freeradbiomed.2025.08.012 40782976

[702] Cheng, Qi , Lijun Mou , Wenjing Su , Xin Chen , Ting Zhang , Yifan Xie , Jing Xue , et al. 2023. “Ferroptosis of CD163^+^ Tissue‐Infiltrating Macrophages and CD10^+^ PC^+^ Epithelial Cells in Lupus Nephritis.” Frontiers in Immunology 14: 1171318. 10.3389/fimmu.2023.1171318 37583695 PMC10423811

[703] Jia, Na , Jia Cui , Lei Wang , Zifeng Huang , Xingru Tao , Qianqian Dong , and Jingwen Wang . 2025. “Restoring Macrophage Iron Homeostasis by Natural Carrier‐Free Nanoplatform for the Therapy of Rheumatoid Arthritis Syndromes.” Materials Today Bio 35: 102544. 10.1016/j.mtbio.2025.102544 PMC12664438 41322152

[704] Zhong, Xiaoyan , Yang Yang , Limei Jian , Zhenfeng Cheng , Jun Liu , Yukai Huang , Qidang Huang , et al. 2026. “Selenium Nanoparticles Efficiently Inhibit M1 Macrophage Polarization by Regulating Selenoprotein to Scavenge ROS in Alleviating Rheumatoid Arthritis.” Colloids and Surfaces B: Biointerfaces 258: 115236. 10.1016/j.colsurfb.2025.115236 41205348

[705] Ling, Yi , Yuzheng Yang , Nina Ren , Hui Xu , Changming Cheng , Daomin Lu , Qiuyi Wang , Xueming Yao , and Wukai Ma . 2024. “Jinwu Jiangu Capsule Attenuates Rheumatoid Arthritis via the SLC7A11/GSH/GPX4 Pathway in M1 Macrophages.” Phytomedicine 135: 156232. 10.1016/j.phymed.2024.156232 39547097

[706] Eltyar, Fatma S. , Dalia M. El‐Tanbouly , Hala F. Zaki , and Rehab M. El‐Sayed . 2025. “Crosstalk between Ferroptosis and NLRP3, a Possible Therapeutic Target in Experimentally‐Induced Rheumatoid Arthritis: Role of P2Y12R Inhibition in Modulating P53/SLC7A11/ALOX15 Signaling.” Inflammopharmacology 33: 3947–3967. 10.1007/s10787-025-01841-8 40622464 PMC12354577

[707] Liu, Hui , Haoyu Wan , Anbiao Zhang , Yi Ouyang , Xinya Lu , Mengyuan Wu , Ning Hu , et al. 2025. “Polypyrrole‐Ferric Phosphate‐Methotrexate Nanoparticles Enhance Apoptosis/Ferroptosis of M1 Macrophages Via Autophagy Blockage for Rheumatoid Arthritis Treatment.” Journal of Nanobiotechnology 23: 428. 10.1186/s12951-025-03501-z 40481493 PMC12144751

[708] Wang, Xiaohua , Tingting Gao , Jingjing Wu , Xu Zhou , Yuanyuan Chu , Lei Zhang , and Xi Cao . 2025. “A Dual‐Targeted Nano‐System Co‐Regulating Macrophage ROS and Fibroblast Ferroptosis for Rheumatoid Arthritis Treatment.” Biomaterials Science 13: 4524–4537. 10.1039/d5bm00618j 40635583

[709] Su, Haibo , Baoying Zhang , Qiudi Deng , Jiaxin Huang , Jinyu Feng , Yuan Fu , Yuejun Huang , et al. 2025. “Trained Immunity Exacerbates Inflammatory Arthritis Progression via Promoting Synovial Fibroblast Ferroptotic Resistance.” Advanced Science 12: e04245. 10.1002/advs.202504245 41255239 PMC12697851

[710] Cheng, Qi , Mo Chen , Mengdan Liu , Xin Chen , Lingjiang Zhu , Jieying Xu , Jing Xue , Huaxiang Wu , and Yan Du . 2022. “Semaphorin 5A Suppresses Ferroptosis Through Activation of PI3K‐AKT‐mTOR Signaling in Rheumatoid Arthritis.” Cell Death & Disease 13: 608. 10.1038/s41419-022-05065-4 35835748 PMC9283415

[711] Zhang, Yage , Fan Shi , Haoyu Tang , Tianyi Wang , Hui Zhao , Jie Chang , Chengyao Zhu , et al. 2026. “Copper Supports Regulatory T Cell Energetic State to Sustain Peripheral Immune Tolerance.” Science Immunology 11: eaec2573. 10.1126/sciimmunol.aec2573 41931599

[712] Guo, Boda , Feiya Yang , Lingpu Zhang , Qinxin Zhao , Wenkuan Wang , Lu Yin , Dong Chen , et al. 2023. “Cuproptosis Induced by ROS Responsive Nanoparticles With Elesclomol and Copper Combined With αPD‐L1 for Enhanced Cancer Immunotherapy (Adv. Mater. 22/2023).” Advanced Materials 35: e2212267. 10.1002/adma.202370152 36916030

[713] Lin, Xinyue , Jieyi Ping , Yi Wen , and Yan Wu . 2020. “The Mechanism of Ferroptosis and Applications in Tumor Treatment.” Frontiers in Pharmacology 11: 1061. 10.3389/fphar.2020.01061 32774303 PMC7388725

[714] Hadian, Kamyar , and Brent R. Stockwell . 2021. “A Roadmap to Creating Ferroptosis‐Based Medicines.” Nature Chemical Biology 17: 1113–1116. 10.1038/s41589-021-00853-z 34675413 PMC8990224

[715] Bhatia, Sangeeta N. , Xiaoyuan Chen , Marina A. Dobrovolskaia , and Twan Lammers . 2022. “Cancer Nanomedicine.” Nature Reviews Cancer 22: 550–556. 10.1038/s41568-022-00496-9 PMC9358926 35941223

[716] Yan, Hong‐fa , Ting Zou , Qing‐zhang Tuo , Shuo Xu , Hua Li , Abdel Ali Belaidi , and Peng Lei . 2021. “Ferroptosis: Mechanisms and Links With Diseases.” Signal Transduction and Targeted Therapy 6: 49. 10.1038/s41392-020-00428-9 33536413 PMC7858612

[717] Yao, Yin , Zhian Chen , Hao Zhang , Cailing Chen , Ming Zeng , Joseph Yunis , Yunbo Wei , et al. 2021. “Selenium‐GPX4 Axis Protects Follicular Helper T Cells From Ferroptosis.” Nature Immunology 22: 1127–1139. 10.1038/s41590-021-00996-0 34413521

[718] Xie, Y. , W. Hou , X. Song , Y. Yu , J. Huang , X. Sun , R. Kang , and D. Tang . 2016. “Ferroptosis: Process and Function.” Cell Death & Differentiation 23: 369–379. 10.1038/cdd.2015.158 26794443 PMC5072448

[719] Ichikawa, Yoshihiko , Mohsen Ghanefar , Marina Bayeva , Rongxue Wu , Arineh Khechaduri , Sathyamangla V. Naga Prasad , R. Kannan Mutharasan , Tejaswitha Jairaj Naik , and Hossein Ardehali . 2014. “Cardiotoxicity of Doxorubicin Is Mediated Through Mitochondrial Iron Accumulation.” Journal of Clinical Investigation 124: 617–630. 10.1172/JCI72931 24382354 PMC3904631

[720] Lei, Peng , Scott Ayton , David I. Finkelstein , Loredana Spoerri , Giuseppe D. Ciccotosto , David K. Wright , Bruce X. W. Wong , et al. 2012. “Tau Deficiency Induces Parkinsonism With Dementia by Impairing APP‐Mediated Iron Export.” Nature Medicine 18: 291–295. 10.1038/nm.2613 22286308

[721] Harischandra, Dilshan S. , Shivani Ghaisas , Gary Zenitsky , Huajun Jin , Arthi Kanthasamy , Vellareddy Anantharam , and Anumantha G. Kanthasamy . 2019. “Manganese‐Induced Neurotoxicity: New Insights Into the Triad of Protein Misfolding, Mitochondrial Impairment, and Neuroinflammation.” Frontiers in Neuroscience 13: 654. 10.3389/fnins.2019.00654 31293375 PMC6606738

[722] Ryan, Sean K. , Matija Zelic , Yingnan Han , Erin Teeple , Luoman Chen , Mahdiar Sadeghi , Srinivas Shankara , et al. 2023. “Microglia Ferroptosis Is Regulated by SEC. 24B and Contributes to Neurodegeneration.” Nature Neuroscience 26: 12–26. 10.1038/s41593-022-01221-3 36536241 PMC9829540

[723] Fang, Jun , Tomohiro Sawa , Takaaki Akaike , Teruo Akuta , Sanjeeb K. Sahoo , Greish Khaled , Akinobu Hamada , and Hiroshi Maeda . 2003. “In Vivo Antitumor Activity of Pegylated Zinc Protoporphyrin: Targeted Inhibition of Heme Oxygenase in Solid Tumor.” Cancer Research 63: 3567–3574 https://www.ncbi.nlm.nih.gov/pubmed/12839943 12839943

[724] Wang, Junrong , Yulin Xie , Guoqing Zhu , Yanrong Qian , Qianqian Sun , Haoze Li , and Chunxia Li . 2025. “Acidity‐Unlocked Glucose Oxidase as Drug Vector to Boost Intratumor Copper Homeostatic Imbalance‐Enhanced Cuproptosis for Metastasis Inhibition and Anti‐Tumor Immunity.” Biomaterials 319: 123207. 10.1016/j.biomaterials.2025.123207 40037207

[725] Song, Rundi , Tianliang Li , Jiayi Ye , Fang Sun , Bo Hou , Madiha Saeed , Jing Gao , et al. 2021. “Acidity‐Activatable Dynamic Nanoparticles Boosting Ferroptotic Cell Death for Immunotherapy of Cancer.” Advanced Materials 33: e2101155. 10.1002/adma.202101155 34170581

[726] Hangen, Emilie , Olivier Féraud , Sylvie Lachkar , Haiwei Mou , Nunzianna Doti , Gian Maria Fimia , Ngoc‐vy Lam , et al. 2015. “Interaction Between AIF and CHCHD4 Regulates Respiratory Chain Biogenesis.” Molecular Cell 58: 1001–1014. 10.1016/j.molcel.2015.04.020 26004228

[727] Dong, Zhe , Peng Liang , Guoqiang Guan , Baoli Yin , Youjuan Wang , Renye Yue , Xiaobing Zhang , and Guosheng Song . 2022. “Overcoming Hypoxia‐Induced Ferroptosis Resistance via a (19) F/(1) H‐MRI Traceable Core‐Shell Nanostructure.” Angewandte Chemie International Edition 61: e202206074. 10.1002/anie.202206074 36222012

[728] Chen, Liyun , Qian Wu , Chaohui Lin , Zijun Song , Yunxing Su , Chaodong Ge , Xue Wang , et al. 2025. “Integrative Analysis of Copper Dysregulation and Cuproptosis in Postnatal Hematopoiesis.” Science Bulletin 70: 2658–2675. 10.1016/j.scib.2025.05.019 40473539

[729] Hofmans, Sam , Tom Vanden Berghe , Lars Devisscher , Behrouz Hassannia , Sophie Lyssens , Jurgen Joossens , Pieter Van Der Veken , Peter Vandenabeele , and Koen Augustyns . 2016. “Novel Ferroptosis Inhibitors With Improved Potency and ADME Properties.” Journal of Medicinal Chemistry 59: 2041–2053. 10.1021/acs.jmedchem.5b01641 26696014

[730] Van Coillie, Samya , Emily Van San , Ines Goetschalckx , Bartosz Wiernicki , Banibrata Mukhopadhyay , Wulf Tonnus , Sze Men Choi , et al. 2022. “Targeting Ferroptosis Protects Against Experimental (Multi)Organ Dysfunction and Death.” Nature Communications 13: 1046. 10.1038/s41467-022-28718-6 PMC8873468 35210435

[731] Devisscher, Lars , Samya Van Coillie , Sam Hofmans , Dries Van Rompaey , Kenneth Goossens , Eline Meul , Louis Maes , et al. 2018. “Discovery of Novel, Drug‐Like Ferroptosis Inhibitors With In Vivo Efficacy.” Journal of Medicinal Chemistry 61: 10126–10140. 10.1021/acs.jmedchem.8b01299 30354101

[732] Gaschler, Michael M. , Alexander A. Andia , Hengrui Liu , Joleen M. Csuka , Brisa Hurlocker , Christopher A. Vaiana , Daniel W. Heindel , et al. 2018. “FINO(2) Initiates Ferroptosis Through GPX4 Inactivation and Iron Oxidation.” Nature Chemical Biology 14: 507–515. 10.1038/s41589-018-0031-6 29610484 PMC5899674

[733] Desai, Neil . 2012. “Challenges in Development of Nanoparticle‐Based Therapeutics.” The AAPS Journal 14: 282–295. 10.1208/s12248-012-9339-4 22407288 PMC3326161

[734] Shi, Jinjun , Philip W. Kantoff , Richard Wooster , and Omid C. Farokhzad . 2017. “Cancer Nanomedicine: Progress, Challenges and Opportunities.” Nature Reviews Cancer 17: 20–37. 10.1038/nrc.2016.108 27834398 PMC5575742

[735] Liu, Xiangsheng , and Huan Meng . 2021. “Consideration for the Scale‐Up Manufacture of Nanotherapeutics—A Critical Step for Technology Transfer.” VIEW 2: 20200190. 10.1002/VIW.20200190

[736] Liu, Wen , Jinpei Wu , Xin Ji , Yandong Ma , Lamei Liu , Xiaoqing Zong , Haiyuan Yang , et al. 2020. “Advanced Biomimetic Nanoreactor for Specifically Killing Tumor Cells Through Multi‐Enzyme Cascade.” Theranostics 10: 6245–6260. 10.7150/thno.45456 32483451 PMC7255035

[737] Duskey, Jason Thomas , Federica da Ros , Ilaria Ottonelli , Barbara Zambelli , Maria Angela Vandelli , Giovanni Tosi , and Barbara Ruozi . 2020. “Enzyme Stability in Nanoparticle Preparations Part 1: Bovine Serum Albumin Improves Enzyme Function.” Molecules 25: 4593. 10.3390/molecules25204593 33050145 PMC7587188

[738] Torres‐Herrero, Beatriz , Ilaria Armenia , Cecilia Ortiz , Jesús Martinez de la Fuente , Lorena Betancor , Valeria Grazú . 2024. “Opportunities for Nanomaterials in Enzyme Therapy.” Journal of Controlled Release 372: 619–647. 10.1016/j.jconrel.2024.06.035 38909702

[739] Shen, Zheyu , Ting Liu , Yan Li , Joseph Lau , Zhen Yang , Wenpei Fan , Zijian Zhou , et al. 2018. “Fenton‐Reaction‐Acceleratable Magnetic Nanoparticles for Ferroptosis Therapy of Orthotopic Brain Tumors.” ACS Nano 12: 11355–11365. 10.1021/acsnano.8b06201 30375848

[740] Li, Jie , Feng Cao , He‐liang Yin , Zi‐jian Huang , Zhi‐tao Lin , Ning Mao , Bei Sun , and Gang Wang . 2020. “Ferroptosis: Past, Present and Future.” Cell Death & Disease 11: 88. 10.1038/s41419-020-2298-2 32015325 PMC6997353

[741] Galluzzi, Lorenzo , Ilio Vitale , Sarah Warren , Sandy Adjemian , Patrizia Agostinis , Aitziber Buqué Martinez , Timothy A. Chan , et al. 2020. “Consensus Guidelines for the Definition, Detection and Interpretation of Immunogenic Cell Death.” Journal for ImmunoTherapy of Cancer 8: e000337. 10.1136/jitc-2019-000337 32209603 PMC7064135

[742] Sperandio, Sabina , Ian de Belle , and Dale E. Bredesen . 2000. “An Alternative, Nonapoptotic Form of Programmed Cell Death.” Proceedings of the National Academy of Sciences 97: 14376–14381. 10.1073/pnas.97.26.14376 PMC18926 11121041

[743] Flannick, Jason , Gudmar Thorleifsson , Nicola L. Beer , Suzanne B. R. Jacobs , Niels Grarup , Noël P. Burtt , Anubha Mahajan , et al. 2014. “Loss‐Of‐Function Mutations in SLC30A8 Protect Against Type 2 Diabetes.” Nature Genetics 46: 357–363. 10.1038/ng.2915 24584071 PMC4051628

[744] Chin, Emily N. , Chenguang Yu , Vincent F. Vartabedian , Ying Jia , Manoj Kumar , Ana M. Gamo , William Vernier , et al. 2020. “Antitumor Activity of a Systemic STING‐Activating Non‐Nucleotide cGAMP Mimetic.” Science 369: 993–999. 10.1126/science.abb4255 32820126

[745] Sugahara, Kazuki N. , Tambet Teesalu , Priya Prakash Karmali , Venkata Ramana Kotamraju , Lilach Agemy , Olivier M. Girard , Douglas Hanahan , Robert F. Mattrey , and Erkki Ruoslahti . 2009. “Tissue‐Penetrating Delivery of Compounds and Nanoparticles Into Tumors.” Cancer Cell 16: 510–520. 10.1016/j.ccr.2009.10.013 19962669 PMC2791543

[746] Wu, Jiao , Zhuan Feng , Liang Chen , Yong Li , Huijie Bian , Jiejie Geng , Zhao‐Hui Zheng , et al. 2022. “TNF Antagonist Sensitizes Synovial Fibroblasts to Ferroptotic Cell Death in Collagen‐Induced Arthritis Mouse Models.” Nature Communications 13: 676. 10.1038/s41467-021-27948-4 PMC8813949 35115492

[747] Liu, Jun , Peng Qu , Jiao Shi , Tingting Liang , Yao Gu , Xue Li , Cui Ma , et al. 2026. “Engineered Multifunctional Hydrogel Delivering Novel CBX7 Inhibitor Modulates Cuproptosis via Liquid‐Liquid Phase Separation to Restore Cardiac Function in Aged Myocardial Infarction.” Advanced Science 13: e11630. 10.1002/advs.202511630 41117088 PMC12767114

